# A firm-push-to-open and light-push-to-lock strategy for a general chemical platform to develop activatable dual-modality NIR-II probes

**DOI:** 10.1126/sciadv.ado2037

**Published:** 2024-06-14

**Authors:** Lili Shen, Jian Li, Chenglong Wen, Hao Wang, Nian Liu, Xinhui Su, Jianzhong Chen, Xin Li

**Affiliations:** ^1^ College of Pharmaceutical Sciences, Zhejiang University, 866 Yuhangtang Street, Hangzhou 310058, China.; ^2^ Department of Nuclear Medicine, The First Affiliated Hospital, Zhejiang University School of Medicine, Hangzhou, China.; ^3^ National Key Laboratory of Chinese Medicine Modernization, Innovation Center of Yangtze River Delta, Zhejiang University, Jiashan 314100, China.; ^4^ State Key Laboratory of Advanced Drug Delivery and Release Systems, Zhejiang University, Hangzhou 310058, China.

## Abstract

Activatable near-infrared (NIR) imaging in the NIR-II range is crucial for deep tissue bioanalyte tracking. However, designing such probes remains challenging due to the limited availability of general chemical strategies. Here, we introduced a foundational platform for activatable probes, using analyte-triggered smart modulation of the π-conjugation system of a NIR-II–emitting rhodamine hybrid. By tuning the nucleophilicity of the ortho-carboxy moiety, we achieved an electronic effect termed “firm-push-to-open and light-push-to-lock,” which enables complete spirocyclization of the probe before sensing and allows for efficient zwitterion formation when the light-pushing aniline carbamate trigger is transformed into a firm-pushing aniline. This platform produces dual-modality NIR-II imaging probes with ~50-fold fluorogenic and activatable photoacoustic signals in live mice, surpassing reported probes with generally below 10-fold activatable signals. Demonstrating generality, we successfully designed probes for hydrogen peroxide (H_2_O_2_) and hydrogen sulfide (H_2_S). We envision a widespread adoption of the chemical platform for designing activatable NIR-II probes across diverse applications.

## Teaser

A breakthrough chemical platform delivers ultrasensitive activatable NIR-II imaging probes to unlock deep tissue secrets.

## INTRODUCTION

Near-infrared II (NIR-II) optical imaging, operating within the wavelength range of 900 to 1700 nm, has emerged as a valuable modality offering reduced autofluorescence and scattering, leading to increased penetration depth and revolutionizing the study of biology (*1*–*4*). In vivo NIR-II imaging not only enhances our ability to capture spatiotemporal-resolved biological data but also improves its relevance in understanding pathophysiological processes, surpassing the limitations of in vitro and ex vivo imaging conducted at cellular and tissue levels. Apart from the instrumental aspect, the success of NIR-II imaging heavily relies on appropriate optical probes. Over the past decade, various inorganic and organic agents have been developed as NIR-II dyes, serving multiple biomedical imaging purposes (*5*–*8*). These dyes can be used directly by themselves to image the blood and lymphatic vasculature networks (*9*–*11*), offering valuable phenotypic insights on disease progression. Alternatively, they can be chemically conjugated with antibodies or affibodies, enabling molecular imaging of target proteins (*12*, *13*). However, both applications face a substantial challenge stemming from background signals induced by unspecific probe absorption or binding, given that these probes are inherently signal active (*14*). Typically, the signal-to-background ratios (SBRs) generally fall into the range of 5 to 10 for these always-on NIR-II fluorescent probes (*12*, *15*, *16*). In addressing this issue, activatable NIR-II probes hold the promise. However, although activatable probes in the visible or NIR-I window have gained great success, the design of activatable NIR-II probes remains challenging, largely due to the limited availability of general chemical strategies to modulate NIR-II emission.

**Fig. 1.**
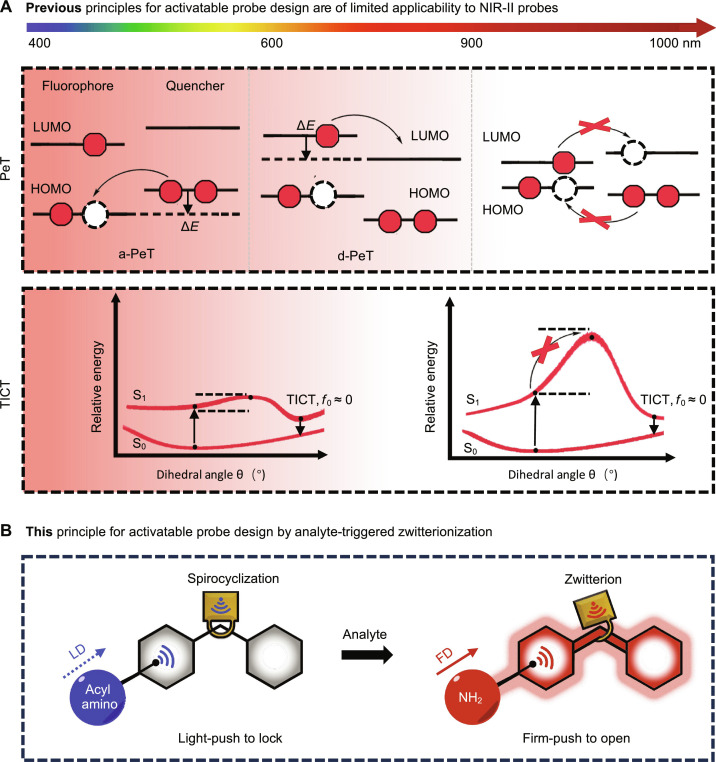
Strategies for fluorescence tuning. (**A**) Schematic illustration of acceptor-excited PeT (a-PeT) or donor-excited PeT (d-PeT), and TICT principles for fluorescence quenching, which are inherently of limited applicability for NIR-II probes. (**B**) Our strategy of analyte-triggered zwitterionization for designing NIR-II probes with ultra–turn-on signals. The light electron-donating (LD) acyl amino group tends to keep the probe in the nonfluorescent spirocyclic form. In contrast, analyte-triggered conversion to the firm electron-donating (FD) amino group would shift the equilibrium to the fluorescent zwitterionic form.

Now, activatable probes in the visible or NIR-I window are usually designed leveraging the following photophysical principles: Förster resonance energy transfer (FRET) (*17*, *18*), intramolecular photoinduced electron transfer (PeT) (*19*, *20*), twisted intramolecular charge transfer (TICT) (*21*), etc. Although these principles were also used to design NIR-II probes, they exhibited limited compatibility with NIR-II dyes. This limitation arises from the inherently low highest occupied molecular orbital (HOMO)/lowest unoccupied molecular orbital (LUMO) energy gaps of these dyes, which pose challenges in constructing counterpart dyes for efficient energy transfer, as well as hindering essential electron transfer processes for PeT quenching and the twisted charge transfer processes for TICT quenching (Fig. 1A) (*22*). Although several FRET-based activatable NIR-II probes have been developed, they are largely by nature formulated composites beyond the scope of small molecules (*10*, *23*, *24*). Although several activatable NIR-II probes based on the PeT or TICT mechanism were reported, they demonstrated only limited SBRs of 3 to 7 in live mouse imaging applications (*25*–*28*). In this context, there is a pressing need for innovative and general strategies in the design of activatable NIR-II probes with ultrahigh SBRs.

**Fig. 2.**
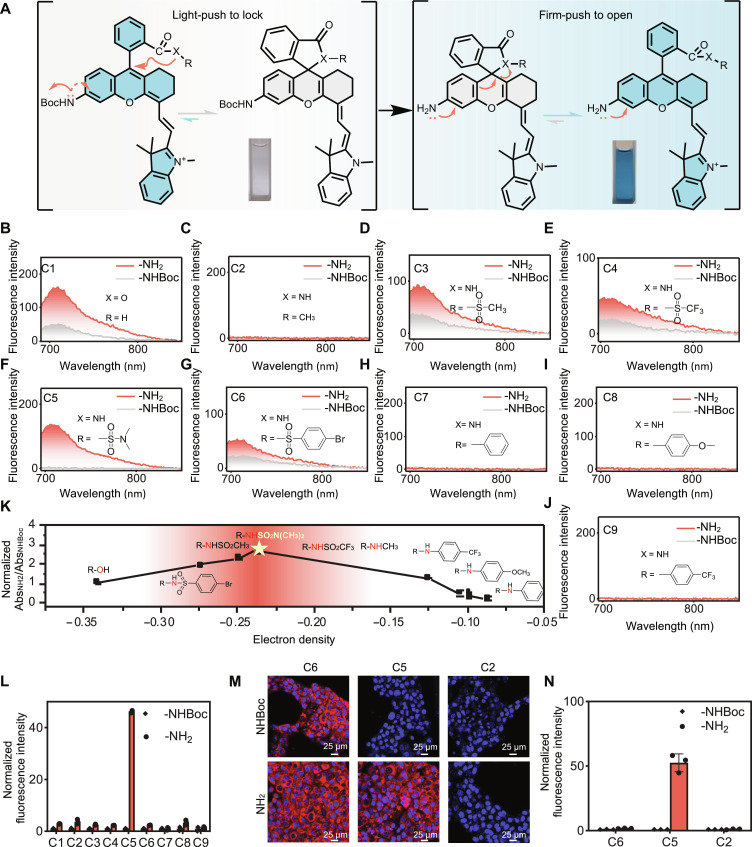
Tuning the *ortho*-carboxy moiety for efficient FOLL effect. (**A**) Structures of the model compounds. (**B** to **J**) Fluorescence spectra of C1 to C9 in PBS buffer (pH 7.4), λ_ex_ = 680 nm. (**K**) Plotting Abs_NH2_/Abs_NHBoc_ at 680 nm as a rough estimation of the zwitterionic form proportion ratio between the NH_2_ and the NHBoc compound versus the electron density of the *meso*-N (or O) atom (R was the benzoyl group). Data were normalized to the ratio of C1 pair compounds and were presented as mean ± SD (*n* = 3 independent samples). (**L**) Normalized fluorescence intensity of C1 to C9 at 720 nm in PBS buffer (pH 7.4). The data of the NH_2_ derivative was normalized to that of the NHBoc counterpart and presented as mean values ± SD (*n* = 3 independent samples). (**M**) Representative images of 4T1 cells after being stained with C2-NH_2_/-NHBoc, C5-NH_2_/-NHBoc, and C6-NH_2_/-NHBoc for 90 min at 37°C. (**N**) Quantified cellular probe fluorescence intensity. Data were normalized to the respective NHBoc group and were presented as mean ± SD (*n* = 3 biologically independent samples).

The equilibrium between the nonfluorescent spirocyclic form and the fluorescent zwitterionic form is a distinctive characteristic of rhodamines (*29*, *30*), offering a rapid pathway for constructing highly efficient fluorogenic probes (fig. S1). The equilibrium could be manipulated by ultraviolet irradiation, protein binding, solvent pH or polarity change, or analyte-triggered structure conversion (*31*) and has been extensively harnessed to develop probes for super-resolution imaging of cellular proteins (*32*–*36*), as well as for sensing cellular microenvironment, enzymes or metabolites (fig. S2) (*37*–*40*). However, one limitation is that the emission of rhodamines remains within the visible window. To extend the benefits of the spirocyclization-zwitterion equilibrium into the NIR spectrum, researchers developed the hybrid molecule Changsha (CS) NIR, which combines merocyanine A and benzoic acid (fig. S3) (*41*). CS NIR exhibits both NIR emission and switchable fluorescence due to the carboxylic group–induced spirocyclization-zwitterion equilibrium. Inspired by this development, a range of NIR-emitting rhodamine hybrid polymethine dyes were created over the past decade (fig. S4) (*42*, *43*). However, these dyes often featured an *ortho*-carboxylic group with limited nucleophilicity, leading to insufficient spirocyclization and, consequently, notable background fluorescence (as exemplified by the data in Fig. 2B). Although transforming the carboxylic group into a spirolactam can markedly inhibit the background signal (*44*), this has limited applicability for sensing probe design because few sensing reactions can transform the spirolactam into a fluorescent zwitterion.

Johnsson *et al.* (*45*–*47*) pioneered to tune the spirocyclization-zwitterion equilibrium of rhodamines by modulating the *ortho*-carboxy moiety, and the resulting fluorophore could be conjugated with a protein ligand to realize the no-wash super-resolution imaging of the target protein. This strategy used the environment-sensitive nature of the equilibrium. However, since even a low zwitterionic proportion is sufficient to afford the desirable signal for super-resolution imaging, whether this strategy could be used to develop activatable NIR-II probes for conventional microscopy or in vivo imaging which is inherently much less sensitive remained unknown, which necessitated not only the complete spirocyclization of the intact probe but also the sufficient zwitterion of the product after sensing.

Here, we explored how various *ortho*-carboxy moieties influenced the equilibrium in a pair of CS NIR derivatives: one with an NHBoc and the other with an NH_2_ substituent at the C5 position of the fluorophore, mimicking the behavior of reaction-based probes before and after they sense their biological targets (Fig. 1B). Our investigation revealed a synergistic effect between the electron-donating ability of the C5 substituent and the nucleophilicity of the *ortho*-carboxy moiety. This led to the identification of an ideally matched electronic effect, which we term “firm-push-to-open and light-push-to-lock” (FOLL), signifying NHBoc substituent–induced spirocyclization before sensing and NH_2_ substituent–induced zwitterion after sensing, assuming the ideally matched *ortho*-carboxy moiety is in place. By FOLL, ~50-fold fluorogenic response was observed in both solution- and cell-based imaging experiments.

Translating the FOLL effect into an optimized rhodamine hybrid whose maximum emission (920 nm) falls into the NIR-II window, we established a general chemical platform for designing NIR-II probes with minimal background and ultrafluorogenic signals in live mice (SBR ~ 50). Furthermore, this spirocyclization-zwitterion equilibrium mechanism in our chemical platform facilitated its application in activatable photoacoustic imaging. As a proof of concept, we successfully developed an H_2_O_2_ probe using this platform. This enabled fluorogenic and activatable photoacoustic imaging of endogenous H_2_O_2_ with nearly zero background signal in live cells and live mice, demonstrating the platform’s reliability. The potential generality of the platform was also exemplified by the successful construction of an H_2_S probe.

## RESULTS

### Tuning the *ortho*-carboxy moiety for efficient FOLL effect

We initiated our study using the CS NIR fluorophore as a model due to its ease of synthesis. Given the broad applicability of analyte-triggered transformations from phenyl carbamates to anilines for sensing enzymatic activities, reactive metabolites, or metal ions, we strategically introduced two substituents to the C5 position of the fluorophore: an electron less donating NHBoc group and an electron firm donating NH_2_ group (Fig. 2A). Our objectives were twofold: (i) to mimic the structural changes of a probe before and after sensing its target and (ii) to emulate the different electrophilicity of the fluorophore core before and after sensing. Our goal was to identify an *ortho*-carboxy moiety that would favor maximum spirocyclization for the NHBoc-substituted structure (the “light-push-to-lock” effect) and maximum zwitterion formation for the NH_2_-substituted counterpart (the “firm-push-to-open” effect).

We initially synthesized C1 compounds with carboxyl acid as the *ortho*-carboxy moiety (Fig. 2B). Fluorescence spectra measurements revealed moderate background signals in the NIR range for NHBoc-C1, indicating inefficient spirocyclization which was further supported by its marked absorption in this range (fig. S5). This is consistent with the limited nucleophilicity of the carboxyl acid. To enhance spirocyclization propensity, we converted the carboxyl acid group into the more nucleophilic *N*-methyl amide. While this transformation completely eliminated background fluorescence in the NIR range of NHBoc-C2, it hindered zwitterion formation in the NH_2_-substituted counterpart due to the high nucleophilicity of the *N*-methyl amide (Fig. 2C and fig. S6).

We then fine-tuned the nucleophilicity of the amide group by either reducing the electronic density of the nitrogen atom or increasing its surrounding steric effects. This led to the synthesis of seven additional pairs of compounds (C3 to C9), and their fluorescence spectra were recorded (Fig. 2, D to J), together with the quantum yields in the NIR range measured (table S1). As the spirocyclization-zwitterion equilibrium of traditional rhodamines is sensitive to pH changes, we then measured the absorption spectra of NHBoc- and NH_2_-substituted compounds in solutions of various pH (figs. S7 to S14). It turned out that their absorption intensity was inert toward pH change in the range of 4 to 9, implying that the equilibriums were relatively stable in this pH range. We used the ratios of NIR absorption intensity of the NH_2_/NHBoc couple at pH 7.4 as a rough estimation of the change in probe equilibrium between zwitteric and spirocyclic forms (Fig. 2K and fig. S15) and correlated these data with the calculated electron density of the amide nitrogen atoms via Gaussian computation (fig. S16 and table S2) (*48*), which indirectly measured nucleophilicity. As depicted in Fig. 2K, both carboxy moieties with the lowest and highest nucleophilicity resulted in low signal-to-noise contrasts. Poor nucleophiles could not effectively inhibit background signals in the NHBoc-substituted structures due to limited spirocyclization, while strong nucleophiles could not achieve sensitive fluorogenic signals in the NH_2_-substituted structures due to limited zwitterion formation. Among the tested compounds, the C5 pair, featuring an *ortho*-carboxy moiety as an acyl sulfamide, exhibited the most marked activatable fluorescence (Fig. 2L), with NH_2_-C5 showing a sevenfold quantum yield increase compared to NHBoc-C5 (table S1).

To further validate the efficacy of the FOLL effect–induced high SBRs, we stained live cells with three pairs of compounds (C2, C5, and C6), where the NHBoc-substituted compounds represented probes before sensing, and the NH_2_-substituted compounds represented probes after sensing. Cells stained with the C6 pair structures exhibited uniformly strong fluorescence, with only a modest 1.5-fold signal increase observed for NH_2_-C6 compared to NHBoc-C6. Conversely, cells stained with the C2 pair of compounds remained nonfluorescent, aligning with the spectral measurements. As anticipated, cells stained with the C5 pair of compounds demonstrated an exceptionally fluorogenic response, with NHBoc-C5 showing nearly zero background signal and NH_2_-C5 exhibiting marked fluorescence (~50-fold) (Fig. 2, M and N, and fig. S17). These findings further validate the success of our FOLL strategy in designing highly fluorogenic probes.

### Tuning the rhodamine hybrid polymethine dyes for NIR-II emission

**Fig. 3.**
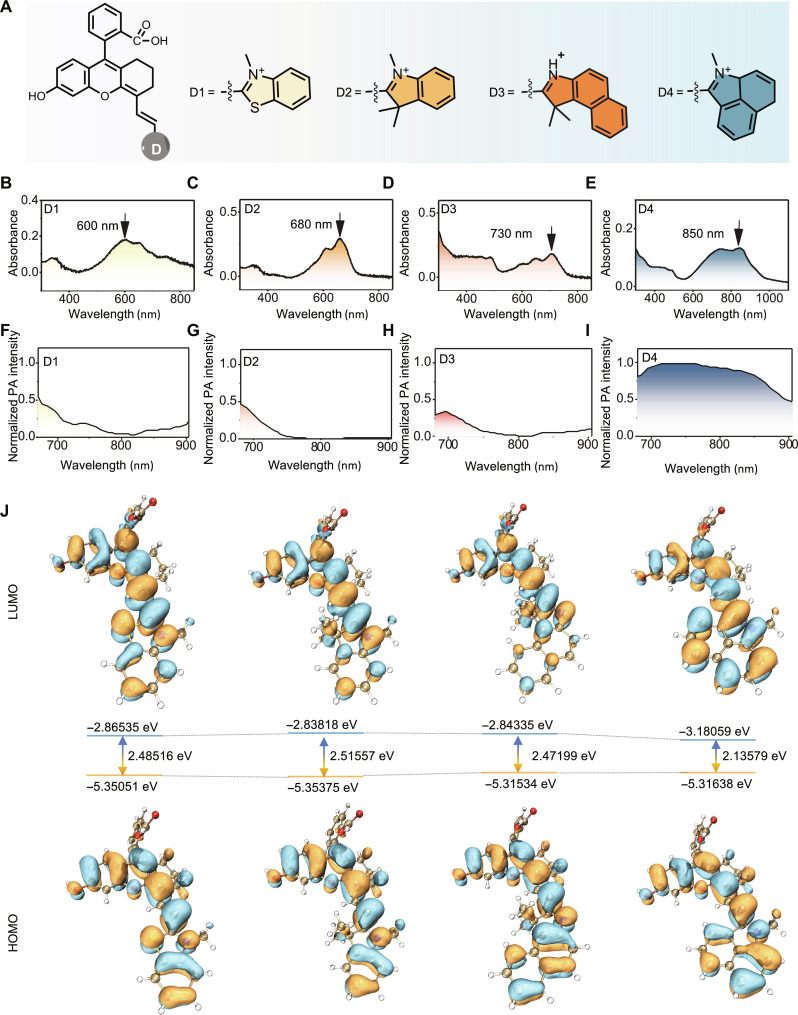
Tuning the rhodamine hybrid polymethine dyes for NIR-II emission. (**A**) Fluorophore structures. (**B** to **E**) Absorbance spectra of D1 to D4 in PBS buffer (pH 7.4). (**F** to **I**) Photoacoustic (PA) spectra of D1 to D4 in PBS buffer (pH 7.4). (**J**) The calculated frontier molecular orbitals of D1 to D4.

Having identified the suitable *ortho*-carboxy moiety, our next objective was to optimize the fluorescence properties of the rhodamine hybrid polymethine dyes. Considering the enhanced appeal of NIR-II imaging, we decided to fine-tune the emission of these hybrids into the NIR-II range by modulating the indolium moiety. Our strategy involved exploring both sulfur substitution and conjugation extension to design fluorophores with NIR-II emissions. We designed four structures and synthesized them through the Knoevenagel reaction (Fig. 3A). Upon examination of their absorption, fluorescence, and photoacoustic spectra (Fig. 3, B to I, and figs. S18 and S19), it became evident that D4 exhibited the most red-shifted absorptive, emissive, and photoacoustic signals, consistent with its possession of the lowest LUMO/HOMO energy gap calculated by the Multiwfn software based on the check files from density functional theory (DFT) at the B3LYP-D3(BJ)/6-311g(d,p) level after geometry optimization at the M06-2X/def-TZVP level (Fig. 3J) (*49*). Notably, D4 demonstrated a tail-like absorption extending beyond the 900-nm range and tremendous photoacoustic intensity. Given that D4 featured a hydroxyl substituent in the C5 position, we hypothesized that its derivatization to the aniline counterpart could further red-shift its emission, owing to the improved electron-donating ability of the nitrogen atom. Therefore, we selected D4 as the candidate fluorophore for the construction of our general chemical platform for designing fluorogenic probes.

### Development of the chemical platform for fluorogenic NIR-II imaging

**Fig. 4.**
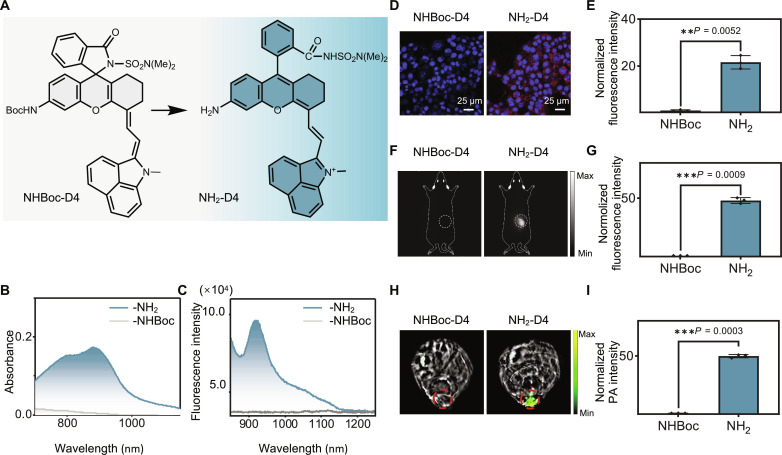
Development of the chemical platform for fluorogenic NIR-II imaging. (**A**) Structures of the model compounds NHBoc-D4 and NH_2_-D4. (**B**) Absorbance spectra of NHBoc-D4 and NH_2_-D4 in PBS buffer (pH 7.4). (**C**) Fluorescence spectra of NHBoc-D4 and NH_2_-D4 in PBS buffer (pH 7.4), λ_ex_ = 850 nm. (**D**) Representative fluorescence images of 4T1 cells stained with NHBoc-D4 and NH_2_-D4 for 90 min at 37°C and (**E**) the normalized fluorescence intensity quantification data. Data were presented as mean ± SD (*n* = 3 biologically independent samples). (**F**) Fluorescence imaging of mice bearing 4T1 tumors that were intratumor injected with either NHBoc-D4 or NH_2_-D4 and imaged after 6 hours and (**G**) the normalized fluorescence intensity quantification data. Data were normalized to the NHBoc group and were presented as mean ± SD (*n* = 3 biologically independent samples). (**H**) Cross-sectional photoacoustic imaging of the mice after intratumor administration of NHBoc-D4 and NH_2_-D4 for 8 hours and (**I**) the normalized photoacoustic intensity quantification data. Data were normalized to the NHBoc group.

Our next step was to assess the compatibility of the nucleophilicity of the acyl sulfamide in C5 with the aniline-derived D4 fluorophore. To investigate this, we synthesized compounds NHBoc-D4 and NH_2_-D4, incorporating the *ortho*-acyl sulfamide moiety (Fig. 4A). By comparing the absorption and fluorescence spectra of this pair of compounds, we discovered that the FOLL effect exhibited even greater efficacy in this fluorophore than in the CS NIR fluorophore. As illustrated in Fig. 4 (B and C), NHBoc-D4 displayed nearly zero absorption and emission within the NIR range, while NH_2_-D4 exhibited intensive absorption and fluorescence in this range. This trend was almost inert toward pH change in the range of 5 to 9 (figs. S20 and S21), implying that the equilibrium was constant in this pH range. Consistent with the absorption spectra, NH_2_-D4 showed a marked photoacoustic signal in the NIR range, while NHBoc-D4 was silent (fig. S22). These findings indicated complete spirocyclization of NHBoc-D4 (representing the probes before sensing) and efficient zwitterion of NH_2_-D4 (representing the probes after sensing).

We further validated the remarkable imaging contrast of this pair of structures in both live cells and live mice. Cells stained with NHBoc-D4 showed no visible fluorescence, while those stained with NH_2_-D4 displayed marked fluorescence; and a ~28-fold fluorogenic signal was observed (Fig. 4, D and E, and fig. S23). Similarly, following the injection of the probes into live mouse tumors, we observed no signal in the NHBoc-D4 group, while the NH_2_-D4 group exhibited marked signals (~50-fold) (Fig. 4, F and G). We observed that upon intravenous administration, NH_2_-D4 showed a whole-body distribution profile. Upon sacrificing mice and examining various organs using a NIR-II imaging system, no discernible fluorescence signal was detected in organs from the NHBoc-D4 group. In contrast, all organs from the NH_2_-D4 group exhibited distinct signals, with the liver displaying the most prominent signal (fig. S24). The contrasts between the two groups varied in different organs, attributed to the varying local concentrations of the probes.

Given the sharp contrast in NIR absorption between NHBoc-D4 and NH_2_-D4, we hypothesized that this structure held promise for activatable photoacoustic imaging. To test this hypothesis, we conducted photoacoustic scans on mice upon intratumor administration of the probes. The cross-sectional images showed no signal in the NHBoc-D4 group, while the NH_2_-D4 group exhibited marked signals (~50-fold) (Fig. 4, H and I), aligning with the results observed by fluorescence imaging.

Together, these data strongly indicate that NH_2_-D4 should serve as an ideal chemical platform for constructing highly fluorogenic NIR-II probes by being transformed into the corresponding carbamates. Moreover, activatable photoacoustic imaging can be realized simultaneously. Noteworthy, NH_2_-D4 at 100 μM in phosphate-buffered saline (PBS) is stable toward the treatment of 20 equivalents of highly oxidative species such as ONOO^−^ and NaClO (fig. S25). These findings underscore the versatility and potential of this chemical platform for a wide range of biological applications.

### Testing the chemical platform for activatable probe design

Our next objective was to evaluate the practicality of this chemical platform for constructing activatable probes for bioanalyte imaging. H_2_O_2_, a prominent member of reactive oxygen species (ROS), plays a pivotal role in regulating various physiological processes, including cell proliferation, differentiation, and migration (*50*–*54*). Aberrant H_2_O_2_ production is associated with the pathophysiology of conditions such as cancer, neurodegenerative diseases, cardiovascular disease, and more (*53*, *55*–*57*). In this study, we aimed to design a NIR-II probe for fluorogenic imaging of H_2_O_2_.

**Fig. 5.**
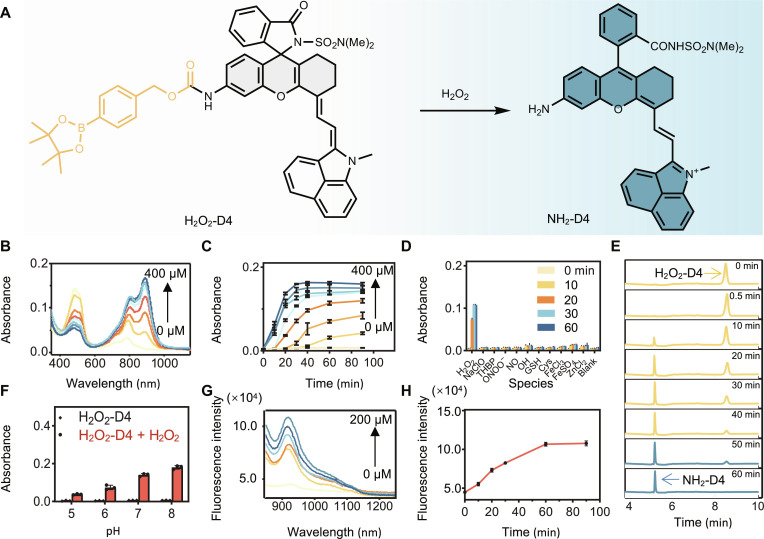
Design of an activatable probe for H_2_O_2_ sensing as a proof-of-concept study. (**A**) Probe structure and the sensing mechanism. (**B**) The absorbance spectra of probe H_2_O_2_-D4 (20 μM) after engaging with H_2_O_2_ (0 to 400 μM) in PBS buffer [pH 7.4, with 20% *N*,*N*′-dimethylformamide (DMF)] for 90 min. (**C**) The time-dependent response of H_2_O_2_-D4 (20 μM) absorbance at 900 nm toward H_2_O_2_ (0 to 400 μM). Data were mean ± SD (*n* = 3 independent samples). (**D**) The absorbance intensity of H_2_O_2_-D4 at 900 nm after the treatment of indicated analytes for 1 hour. The final concentrations of NaClO, TBHP, ONOO^−^, and ·NO were 50 μM, and others were 100 μM. Data were mean ± SD (*n* = 3 independent samples). (**E**) LC traces of probe H_2_O_2_-D4 before and after sensing H_2_O_2_. (**F**) The absorbance intensity of H_2_O_2_-D4 at 900 nm before and after interacting with H_2_O_2_ (400 μM) for 1 hour in PBS buffer with pH ranging from 5 to 8. Data were mean ± SD (*n* = 3 independent samples). (**G**) Fluorescence spectra of H_2_O_2_-D4 (10 μM) after interacting with H_2_O_2_ (0 to 200 μM) for 1 hour in PBS buffer, λ_ex_ = 850 nm. (**H**) Time-lapse fluorescence intensity of H_2_O_2_-D4 (10 μM) at 920 nm after interacting with H_2_O_2_ (200 μM) in PBS buffer (pH 7.4, with 20% DMF), λ_ex_ = 850 nm. Data were mean ± SD (*n* = 3 independent samples).

The phenylborate moiety has a well-established history as a chemical trigger for sensing H_2_O_2_ (*58*, *59*). Consequently, we transformed the NH_2_-D4 fluorophore into a boronophenoxy carbamate. This resulting probe, denoted as H_2_O_2_-D4 (Fig. 5A), was first confirmed for its stability in PBS buffer solution (fig. S26). Then, its response to H_2_O_2_ was tested. H_2_O_2_-D4 exhibited a maximum absorption band centered at 500 nm, indicating its existence in the spirocyclic form. Upon interaction with H_2_O_2_, the 500-nm-centered absorbance decreased, accompanied by the emergence of two neighboring peaks centered at 790 and 900 nm (Fig. 5B). This shift suggested H_2_O_2_-triggered zwitterion formation. Notably, both the decrease and increase in absorbance were proportionate to the doses of H_2_O_2_ applied (Fig. 5C). Our dynamic recording demonstrated that the sensing of H_2_O_2_ with H_2_O_2_-D4 reached completion within 1 hour (Fig. 5C). This observation was further corroborated by liquid chromatography–mass spectrometry (LC-MS) analysis (Fig. 5E and fig. S27), which revealed the full conversion of H_2_O_2_-D4 into NH_2_-D4 within 1 hour of interaction with H_2_O_2_. Notably, we investigated how pH levels would affect the performance of H_2_O_2_-D4 for sensing H_2_O_2_. For this purpose, the sensing reaction was carried out at various biologically relevant pH levels (Fig. 5F). We observed that the probe remained dark in the tested pH levels. However, the sensing signals after H_2_O_2_ interaction intensified as the pH increased. Since we have confirmed that the spirocyclization-zwitterion equilibrium of NH_2_-D4 was insensitive toward pH, this observation was attributed to the more efficient transformation of the probe to NH_2_-D4 under more basic conditions, driven by both the enhanced reactivity of H_2_O_2_ and the accelerated cleavage of the self-immolative linker. Furthermore, we confirmed the high selectivity of the probe toward H_2_O_2_, as other reactive species caused little change in its absorption (Fig. 5D). Similarly, a highly fluorogenic response of the probe was observed upon interaction with H_2_O_2_ (Fig. 5, G and H). Noteworthy, because of the spirocyclization-induced efficient fluorescence quenching in the NIR-II region, H_2_O_2_-D4 could achieve a limit of detection of 7.0 nM for H_2_O_2_ by the 3*S_B_/K* method detailed in the Supplementary Materials.

**Fig. 6.**
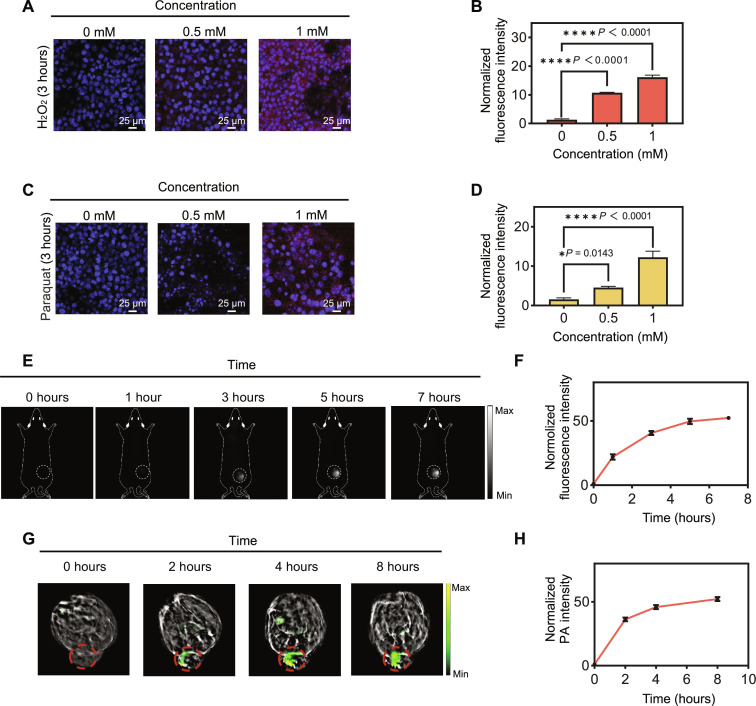
Activatable imaging of H_2_O_2_ in live cells and mice. (**A**) Representative fluorescence images and (**B**) the quantified cellular probe fluorescence intensity data of 4T1 cells. Cells were pretreated with H_2_O_2_ (0 to 1 mM) for 3 hours, washed, and then incubated with H_2_O_2_-D4 (5 μM) for 90 min. Data were normalized to that of the 0 mM H_2_O_2_ group and presented as mean ± SD (*n* = 3 biologically independent samples). (**C**) Representative fluorescence images and (**D**) the quantified fluorescence intensity data of 4T1 cells pretreated with paraquat. After an incubation time of 3 hours with paraquat (0 to 1 mM), cells were washed and stained with H_2_O_2_-D4 (5 μM) for 90 min. Data were normalized to that of the 0 mM paraquat group and presented as mean values ± SD (*n* = 3 biologically independent samples). (**E**) Fluorescence imaging of 4T1 mice before (0 hours) and after intratumor treatment with H_2_O_2_-D4 for 1, 3, 5, and 7 hours. (**F**) Normalized intratumoral probe fluorescence intensity. Data were normalized to the 0-hour group and presented as mean ± SD (*n* = 3 biologically independent samples). (**G**) Cross-sectional photoacoustic imaging of the 4T1 mice before (0 hours) and after intratumor administration of H_2_O_2_-D4 for 2, 4, and 8 hours. (**H**) Normalized intratumoral photoacoustic intensity. Data were normalized to the 0-hour group.

Following the successful assessment of the probe’s performance toward H_2_O_2_ in an aqueous solution–based assay, coupled with confirmation of its safety in both cultured cells (fig. S28) and mice (fig. S29), our next step involved using the probe for the imaging of H_2_O_2_ in biological samples. We initiated this by stimulating cultured cells with H_2_O_2_ at varying concentrations for 3 hours to induce acute oxidative stress. Subsequently, the cells were washed, stained with H_2_O_2_-D4, and subjected to imaging. A dose-dependent increase in cellular fluorescence was observed in response to varying concentrations of H_2_O_2_ (Fig. 6, A and B, and fig. S30). Notably, the cellular probe fluorescence increased approximately 15-fold in the presence of H_2_O_2_ (1.0 mM) compared to the negative control group. Intriguingly, when the cells were pretreated with a ROS scavenging agent, *N*-acetyl-cysteine (NAC), for 1 hour before H_2_O_2_ stimulation, a distinct compromisation in cellular probe fluorescence intensity was observed (fig. S30). We extended our investigation to another model involving paraquat-induced acute oxidative stress. In this setup, cells were initially treated with varying concentrations of paraquat for 3 hours, followed by washing, staining with the probe, and subsequent imaging. Similar to the H_2_O_2_-treated group, we observed a dose-dependent increase in cellular probe fluorescence in response to varying paraquat concentrations (Fig. 6, C and D, and fig. S31). Collectively, these results indicated that H_2_O_2_-D4 had the capability to assess the degree of cellular oxidative stress levels through its fluorogenic signals, thereby providing a valuable tool for monitoring oxidative stress dynamics in live cells.

Building on the promising results obtained in cellular studies, we proceeded to evaluate the feasibility of H_2_O_2_-D4 for imaging H_2_O_2_ in a xenograft 4T1 tumor mouse model. We first confirmed that the fluorescence intensity of NH_2_-D4 recorded by the BLT NIR-30F system was proportional to its concentrations (fig. S32). Then, real-time NIR-II fluorescence imaging was conducted to monitor and quantify the intensification of H_2_O_2_-activated probe fluorescence signals within the tumor of living mice after intratumor administration of the probe (Fig. 6E). After a 7-hour recording, the NIR-II fluorescence at the tumor site was approximately 50 times higher than the background (Fig. 6F), which represents the highest SBR ever reported. Furthermore, we explored the probe’s applicability for activatable photoacoustic imaging of H_2_O_2_. Similar to the NIR-II fluorescence imaging results, we observed a time-dependent increase in photoacoustic signals at the tumor sites of the mice, with virtually no background signal interference, and an SBR of 50 was achieved after 8 hours (Fig. 6, G and H).

The success of H_2_O_2_-D4 in imaging H_2_O_2_ in mouse tumors inspired us to further investigate its performance in imaging H_2_O_2_ up-regulation in drug-induced liver injury, a big concern in both clinical practice and drug development. Trazodone has been associated with rare cases of liver injury, but the mechanism remains largely unknown (*60*). There are reports that oxidative stress may play a role in this process (*61*). To investigate whether trazodone up-regulates H_2_O_2_ levels in mouse livers, the mice after treatment with trazodone were intravenously administrated with H_2_O_2_-D4, and both the NIR-II fluorescence and photoacoustic images were taken before and after the probe administration. As shown by the data in fig. S33, both groups showed gradual signal enhancement in mouse livers under either imaging modalitiy, with the trazodone group demonstrating a more marked intensifying tendency. Noteworthy, the imaging contrast between the trazodone group and the vehicle group was especially distinct under the photoacoustic modality. This result implied that H_2_O_2_-D4 was sensitive enough to capture physiological H_2_O_2_ in mouse livers and that trazodone led to the up-regulation of H_2_O_2_ in mouse livers. It also highlighted the advantage of our strategy for dual-modality activatable imaging.

All the above cell and mouse imaging results underscore the applicability of our chemical platform for designing ultrafluorogenic and activatable photoacoustic probes. This opens up possibilities for the realization of multimodality imaging of various bioanalytes, further enhancing our capabilities for precise and versatile biological imaging applications.

### Generality of the chemical platform for probe design

**Fig. 7.**
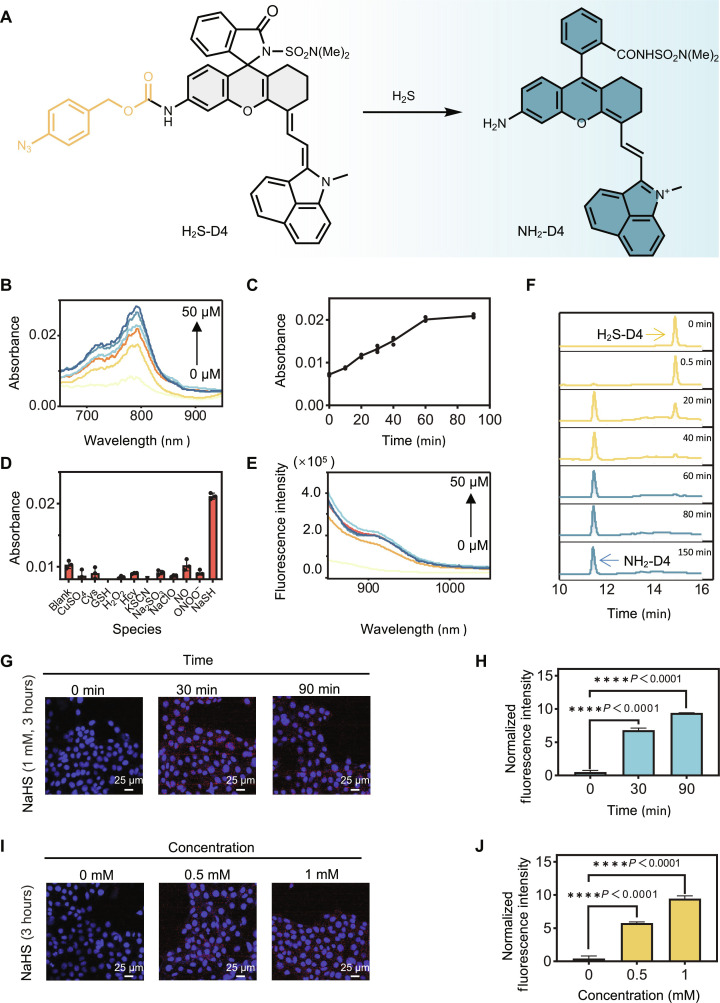
Activatable probe for imaging H_2_S. (**A**) Structure of H_2_S-D4 and its mechanism for detecting H_2_S. (**B**) Absorbance spectra of H_2_S-D4 (20 μM) after interacting with NaHS (0 to 50 μM) in PBS buffer (pH 7.4, 20% DMF) for 90 min. (**C**) Absorbance intensity of H_2_S-D4 (20 μM) at 800 nm after being treated with NaHS (30 μM) for the indicated time. Data were presented as mean ± SD (*n* = 3 independent samples). (**D**) Absorbance intensity of H_2_S-D4 (20 μM) at 800 nm after being treated with the indicated analyte for 90 min. NaHS, NaClO, and ·NO were used at 20 μM. ONOO^−^ was used at 10 μM. All other species were used at 100 μM. Data were presented as mean values ± SD (*n* = 3 independent samples). (**E**) Fluorescence spectra of H_2_S-D4 (10 μM) after being treated with NaHS (0 to 50 μM) in PBS buffer (pH 7.4, 20% DMF) for 90 min (λ_ex_ = 820 nm). (**F**) HPLC traces of H_2_S-D4 before and after being treated with NaHS. (**G**) Representative fluorescence images and (**H**) the quantified fluorescence intensity of 4T1 cells. Cells were pretreated with NaHS (1 mM) for 3 hours, washed, incubated with H_2_S-D4 (5 μM) for the indicated time, and then imaged. Data were normalized to that of the 0-min group and presented as mean values ± SD (*n* = 3 biologically independent samples). (**I**) Representative fluorescence images and (**J**) the quantified fluorescence intensity of 4T1 cells. Cells were pretreated with NaHS (0 to 1 mM) for 3 hours, washed, and then incubated with H_2_S-D4 (5 μM) for 90 min. Data were normalized to that of the 0 mM group and presented as mean ± SD (*n* = 3 biologically independent samples).

Encouraged by the successful application of H_2_O_2_-D4, we embarked on an exploration of the general applicability of our chemical platform for designing activatable NIR-II probes. One such important target molecule is H_2_S, an endogenous gas signal molecule in living systems that plays a pivotal role in a variety of physiological processes (*62*–*64*). The prospect of highly fluorogenic imaging of H_2_S holds promise for gaining valuable insights into its pathophysiology (*65*–*67*). In this context, we developed a H_2_S-sensing probe, denoted as H_2_S-D4 (Fig. 7A), by introducing an H_2_S-responsive trigger to the NH_2_-D4 fluorophore (*68*, *69*). This resulting probe was first confirmed for its stability in a PBS solution (fig. S34).

Our initial investigations aimed to assess the sensing performance of the probe toward H_2_S in an aqueous solution. Absorption spectra revealed that H_2_S-D4 did not exhibit notable absorption in the NIR range (Fig. 7B), suggesting a preference for the spirocyclic form. However, upon exposure to H_2_S, we observed a dose-dependent intensification of NIR absorption, indicating a transition to the zwitterionic form. This transformation was also found to be incubation time dependent (Fig. 7C and fig. S35). The reaction of H_2_S-D4 toward H_2_S was insensitive to pH change ranging from 5 to 8 (fig. S36). Furthermore, we confirmed the high selectivity of the probe toward H_2_S (Fig. 7D), aligning with the reported selectivity of the phenylazide moiety toward H_2_S. Parallel to the absorption analysis, fluorescence spectra measurements demonstrated a remarkable H_2_S-induced fluorogenic response of the probe (Fig. 7E), and the limit of detection was determined to be 23 nM. To gain a deeper insight into the sensing process of the probe toward H_2_S, we used high-performance liquid chromatography (HPLC) analysis, providing additional support for the H_2_S-triggered transformation of the carbamate probe into the NH_2_-D4 fluorophore (Fig. 7F and fig. S37).

H_2_S-D4 exhibited no observable toxicity in either cultured cells (fig. S38) or mice (fig. S39). Subsequently, we evaluated its feasibility for fluorogenically imaging H_2_S in live cells. Cells were first treated with NaHS (1 mM) as an H_2_S donor for 3 hours, subjected to H_2_S-D4 staining (5 μM) for various durations, and then imaged. The results showed a staining time-dependent cellular probe fluorescence intensification (Fig. 7, G and H, and fig. S40), suggesting the continuous transformation of cellular H_2_S-D4 to NH_2_-D4. In another experiment, cells were treated with varying doses of NaHS (0, 0.5, and 1 mM) for 3 hours, followed by a 90-min incubation with H_2_S-D4 and then imaged. The cellular probe fluorescence signal exhibited enhancement in a NaHS dose-dependent manner, suggesting its capability to respond to varying H_2_S concentrations in live cells (Fig. 7, I and J, and fig. S41). We then tested its performance to image H_2_S in an endotoxin-induced acute inflammation process. Lipopolysaccharide (LPS) is reported to produce a dose and time-dependent up-regulation of H_2_S in mice (*70*). To check whether H_2_S-D4 would track this process, mice intraperitoneally treated with LPS and *L*-Cysteine (*L*-Cys) were intravenously administrated with H_2_S-D4, and both the NIR-II fluorescence and photoacoustic signals were recorded (fig. S42). The LPS group demonstrated more marked time-dependent signal intensification, with the signals under the photoacoustic modality showing better contrast between the two groups. This result implied that probe H_2_S-D4 was sensitive enough to image endogenous H_2_S generation.

These results collectively indicate that H_2_S-D4 can effectively assess biological H_2_S through its sensitive fluorogenic signals. The success of this probe not only underscores its utility but also implies the generality and versatility of our chemical platform for designing NIR-II probes. Such probes hold the potential for realizing fluorogenic imaging of various enzymes, reactive metabolites, and other pathological targets, further expanding the horizons of biomedical research and diagnostics.

## DISCUSSION

In recent years, the limitations of optical imaging in the visible spectrum, such as absorption, scattering, and tissue autofluorescence (*71*, *72*), have driven the development of imaging in the NIR, particularly in the NIR-II region (*73*, *74*). This advancement allows for deeper tissue penetration with reduced scattering and autofluorescence, resulting in high-resolution in vivo imaging. NIR-II imaging has rapidly gained prominence in medical and biological research. However, the issue of self-background signals from probes, due to their always-on signals, remains a challenge.

Activatable probes have emerged as a promising solution to address this challenge. Noteworthy, Yuan *et al.* (*75*) developed a versatile chemical platform for the development of NIR-II fluorogenic probes using analyte-triggered transformation of phenol ethers to phenols, and desirable SBRs were achieved. This should inspire the exploration of more tuning strategies to develop activatable NIR-II imaging probes. Nevertheless, NIR-II dyes inherently have low LUMO/HOMO energy gaps, making the application of principles commonly used for designing activatable probes with visible emissions, such as FRET, TICT, and PeT, less practical for developing activatable NIR-II probes. In light of this challenge, an ideal photophysical property tuning strategy tailored to NIR-II dyes has become a pressing need. Tuning the conjugation system through a pure chemical mechanism offers a straightforward approach. NIR-II dyes typically feature an extensive conjugation system for efficient electron delocalization, reducing their LUMO/HOMO energy gaps to enable efficient NIR absorption. Activatable signals could be achieved if this conjugation system can be reversibly disrupted and restored. The reversible fluorescent signals exhibited by rhodamines, transitioning between the nonfluorescent spirocyclic form and the fluorescent zwitterionic form, serve as a prime example of this mechanism.

To harness the potential of *ortho*-carboxy moiety-modulated spirocyclization-zwitterion equilibrium as an efficient design principle for activatable NIR probes, we conducted a systematic study by combining analyte-triggered electronic effects with the nucleophilicity of the *ortho*-carboxy moiety. Using a rhodamine hybrid as our model compound, we used the 5-NHBoc–substituted derivative to mimic the probe before sensing the biotarget, with the 5-NH_2_–substituted counterpart representing the product after sensing. By meticulously adjusting the *ortho*-carboxy moiety, we identified an ideally matched electronic effect—firm-push to open and light-push to lock. This effect ensured optimal spirocyclization for the probe before sensing and maximal zwitterionic character after sensing.

Furthermore, by refining the emission properties of the hybrid through structural modifications, we identified NH_2_-D4 as a fluorophore with NIR-II region emission capabilities. The selected *ortho*-carboxy moiety perfectly complemented this fluorophore. While NHBoc-D4 exhibited negligible NIR absorption, implying complete spirocyclization, NH_2_-D4 displayed substantial NIR absorptivity. Live mouse imaging confirmed the highly fluorogenic and activatable photoacoustic signals of NH_2_-D4 compared to NHBoc-D4.

Last, the NH_2_-D4 fluorophore’s applicability and generality as a chemical platform for the straightforward design of activatable optical probes in the NIR-II range were validated. We successfully developed ultrasensitive fluorogenic NIR-II probes by simply derivatizing the amine group in NH_2_-D4 to a corresponding H_2_O_2_ or H_2_S-responsive carbamate. These probes were also found to be compatible with activatable photoacoustic imaging. In addition, live mouse imaging of either its tumor tissue or injured liver was achieved. Thus, this platform represents a straightforward approach for designing activatable NIR-II probes, enabling dual-modality imaging in the NIR-II spectrum.

It is important to acknowledge that although our study has achieved promising results in designing activatable NIR-II probes for bioanalytes, certain limitations remain for future improvement. The scope of analytes we have explored remains limited. We primarily focused on H_2_O_2_ and H_2_S probes as proof-of-concept examples. Future research should expand upon this platform to encompass a broader range of targets and analytes, enhancing its versatility and practicality.

## MATERIALS AND METHODS

### Synthesis and structure characterization

General chemistry methods, probe synthesis and characterization, HPLC, and LC-MS methods were detailed in the Supplementary Materials.

### Optical spectra measurements

Absorption, fluorescent, and photoacoustic spectra recording methods and the fluorescence quantum yield determination methods were described in the Supplementary Materials.

### Computational calculation methods

All the DFT calculations were performed with the Gaussian16 C.01 program (*48*). Structure optimization was performed using the M06-2X functional and the def-TZVP basis set for all atoms (denoted M06-2X/def-TZVP). Electrostatic potential (ESP), atomic dipole moment corrected Hirshfeld (ADCH) atomic charges, and HOMO and LUMO analyses were carried out using the Multiwfn software (*49*) based on the check files from DFT calculations. To improve the calculation accuracy, single-point energy calculations were performed by the M06-2X functional and the def2-TZVPP basis set (denoted M06-2X/def2-TZVPP) for ADCH charges and ESP analyses, while the B3LYP functional with Grimme’s dispersion correction DFT-D3 (BJ) and the 6-311g(d,p) basis set [denoted B3LYP-D3(BJ)/6-311g(d,p)] for HOMO and LUMO analyses. Furthermore, we have also considered the solvent effects of molecules in water using the solvation model based on density for all the DFT calculations and then presented all the figures by Visual Molecular Dynamics (*76*).

### Ethical statement

All animal experiments were performed according to the guidelines of the Animal Care and Use Committee of Zhejiang University and approved by the Ethics Committee for Animal Experiments of Zhejiang University in China.

### Cell culture

4T1 cells were provided by Stem Cell Bank, Chinese Academy of Sciences. All of them were proved by short tandem repeat analysis by Stem Cell Bank, Chinese Academy of Sciences. 4T1 cells were cultured in high-glucose Dulbecco’s modified Eagle’s medium (DMEM; Gibico) supplemented with 10% fetal bovine serum (Pan) with 1% antibiotics [penicillin (100 U/ml) and streptomycin (100 μg/ml)] at 37°C and 5% CO_2_. Cells were carefully harvested and split when they reached 80% confluence to maintain exponential growth.

### CCK-8 assay

Cells of the logarithmic growth phase were taken and inoculated in 96-well culture plates with edge-well PBS replenishment and incubated in a 37°C incubator with 5% CO_2_. After cell apposition, the cells were administrated with a complete medium containing the tested compounds and incubated for 24 hours. Then, the liquid was replaced in all wells with Cell Counting Kit-8 (CCK-8) solution. The cells were put back into the incubator at 37°C for 1 hour. The absorbance at 450 nm was measured on a TECAN infinite M1000 multifunction microplate reader. The absorbance of each well was compared with the absorbance of normal wells, and the ratio obtained was calculated as the cell survival rate. Cell survival (%) = (absorbance intensity of test sample/absorbance intensity of control) × 100%.

### Confocal fluorescence microscopy

For imaging cells stained with compounds C2, C5, and C6, 4T1 cells were seeded in 15-mm glass-bottomed dishes and cultured for 24 hours. Then, these cells were incubated with compounds C2, C5, and C6 (5 μM) in a serum-free medium for 90 min. After three times washing with PBS, fresh medium without serum was added into the wells, and fluorescence images were recorded on a Leica STELLARIS 5 confocal microscope (channel: λ_ex_ = 680 nm, λ_em_ = 700 to 730 nm). Each experiment was performed three times. Three frames were taken each time for a single focal plane without a Z-stack by random selection. For various conditions in one experiment, the same microscope parameters were used to keep the background signal constant. Images were analyzed via the software “ImageJ” to quantify the fluorescence intensity by densitometry. The mean gray value was used for measuring the fluorescence intensity. No background subtraction was used. No Gaussian blur filter was applied. The measurements of the raw data were pooled across various conditions and the three parallels in one experiment. For imaging cells stained with BocNH-D4 and NH_2_-D4, similar procedures were carried out except that the images were recorded with λ_ex_ = 790 nm, λ_em_ = 800 to 850 nm.

To test the response of H_2_O_2_-D4 to cellular H_2_O_2_, 4T1 cells were seeded in 24-well culture plates with coverslips and cultured for 24 hours. The medium was then changed into a serum-free medium containing various concentrations of H_2_O_2_ (0, 0.5, or 1 mM). After a further incubation time of 3 hours, cells were washed with PBS three times. Then, these cells were incubated with H_2_O_2_-D4 (5 μM) in a serum-free medium for 90 min. After three times washing with PBS, the cells were stained with Hoechst for 15 min. After three times washing with PBS, 4% paraformaldehyde (200 μl) was added to the plate to fix the cells. After three times washing with PBS, coverslips were removed and secured to the slide, and fluorescence images were recorded on a Leica STELLARIS 5 confocal microscope (channel: λ_ex_ = 790 nm, λ_em_ = 800 to 850 nm). Each experiment was performed three times. In addition, images were taken in a similar way as above described.

For the NAC pretreatment group, 4T1 cells were first treated with NAC (100 μM) in serum-free DMEM for 1 hour. Then, cells were treated with H_2_O_2_ and stained with H_2_O_2_-D4 as above described.

To image paraquat-induced cellular H_2_O_2_, 4T1 cells were treated with paraquat (0, 0.5, or 1 mM) in a serum-free medium for 3 hours. Cells were then stained with H_2_O_2_-D4 and imaged as above described.

To test the response of H_2_S-D4 to cellular H_2_S, 4T1 cells were first incubated with NaHS (0, 0.5, or 1 mM) in a serum-free medium for 3 hours, and cells were washed with PBS three times. Then, the cells were incubated with H_2_S-D4 (5 μM) in a serum-free medium for the indicated time. After three times washing with PBS, cells were incubated with Hoechst for 15 min. The cells were then fixed and imaged as above described.

### In vivo imaging

The in vivo imaging experiments followed the Animal Research: Reporting of In Vivo Experiments Essential 10 guidelines. 4T1 mouse breast cancer cells (1 × 10^6^) were injected subcutaneously into the back of Balb/c nude mice (6 weeks old). Upon reaching a tumor volume of 100 mm^3^, 50 μl of NHBoc-D4 or NH_2_-D4 [1.0 mg/ml in deionized water containing 5% dimethyl sulfoxide (DMSO)] was intratumorally injected into the mouse. In vivo fluorescence images were taken before (0 hours) or after (6 hours) the administration of the probe on a NIR-II fluorescence imaging system with excitation at 808 nm and emission collected at 900 nm. Three mice were used for each group. The tumors from the three individuals in each group were taken to measure the average fluorescence signals. The averaged fluorescence signals of tumor regions were analyzed via the software ImageJ to quantify the fluorescence intensity by densitometry. The mean gray value was used for measuring the fluorescence intensity. No background subtraction was used. No Gaussian blur filter was applied. For intravenously (via tail vein) administration, 50 μl of NHBoc-D4 or NH_2_-D4 (1.0 mg/ml in deionized water containing 5% DMSO) was dosed. One mouse was used for each group. After 6 hours, mice were euthanized. The tumors and representative organs were dissected and examined ex vivo.

In vivo photoacoustic imaging of the mice after intratumor administration of NHBoc-D4 and NH_2_-D4 was performed with similar procedures, except that the signals were recorded on an MSOT inVision 256-TF instrument before (0 hours) and after (8 hours) the administration of the probes. One mouse was used for each group. The averaged photoacoustic signals of tumor regions were analyzed using View MSOT 4.0 software (iThera Medical, Munich). Three different areas of the tumor were taken to measure the average photoacoustic signals, and the averaged photoacoustic signals of tumor regions were analyzed via the software ImageJ to quantify the photoacoustic signal intensity by densitometry. The mean gray value was used for measuring the fluorescence intensity. No background subtraction was used. No Gaussian blur filter was applied.

Intratumor administration of H_2_O_2_-D4 (50 μl, 1.0 mg/ml in deionized water containing 5% DMSO) was carried out as above described. In vivo fluorescence images were taken before (0 hours) and after (1, 3, 5, and 7 hours) the administration of the probe using a NIR-II fluorescence imaging system with excitation at 808 nm and emission collected at 900 nm. Three mice were used for each group. The tumors from the three individuals in each group were taken to measure the average fluorescence signals, and the averaged fluorescence signals of tumor regions were analyzed via the software ImageJ to quantify the fluorescence intensity by densitometry. The in vivo photoacoustic imaging after administration of H_2_O_2_-D4 was carried out similarly to the photoacoustic imaging after administration of NH_2_-D4. One mouse was used for each group.

To establish the trazodone-induced liver injury mouse model, ICR male mice (7 to 8 weeks old) were randomly divided into two groups with two mice per group, balancing sufficient replication of results with a reduction in animal number. Mice were treated with trazodone hydrochloride (200 mg/kg per day) or isovolumic saline (control group) via intraperitoneal administration for three consecutive days. Six hours after the last administration, mice were intravenously injected with H_2_O_2_-D4 (1.0 mg/ml in deionized water containing 5% DMSO, 100 μl). In vivo fluorescence images were taken before (0 hours) and after (1, 2, 3, and 4 hours) the administration of the probe using NIR-II fluorescence imaging system with excitation at 808 nm and emission collected at 900 nm. In vivo optoacoustic imaging was carried out on an MSOT inVision 256-TF instrument before (0 hours) and after (1, 2, and 3 hours) the administration of the probe. All mice had hair removed from the imaging area before the experiment.

To develop an LPS-induced liver injury mouse model, ICR male mice (7 to 8 weeks old) were randomly divided into two groups with two mice per group. Mice were intraperitoneally injected with LPS (20 mg/kg) or isovolumic saline (control group). Two hours later, the mice in the LPS group were further intraperitoneally administered with *L*-Cys (1.0 mM, 100 μl). After 30 min, the mice were intravenously injected with H_2_S-D4 (1.0 mg/ml in deionized water containing 5% DMSO, 100 μl). In vivo fluorescence images were taken before (0 hours) and after (1, 2, 3, 4 hours) the administration of the probe using NIR-II fluorescence imaging system with excitation at 808 nm and emission collected at 900 nm. In vivo fluorescence and photoacoustic images were taken as above described.

### In vivo biosafety assessment

C57BL/6J mice (male, 7 to 8 weeks old) were intravenously injected with either H_2_O_2_-D4 or H_2_S-D4 (1.0 mg/ml in deionized water containing 5% DMSO, 100 μl) or with saline (100 μl). Blood samples were taken 1, 8, and 15 days postprobe administration. EDTA-anticoagulated blood was used to test white blood cells, lymphocytes, monocytes, red blood cells, hemoglobin, mean corpuscular hemoglobin, hematocrit, mean corpuscular volume, mean corpuscular hemoglobin concentration, platelets, platelet distribution width, and mean platelet volume. The plasma was used to test creatinine, urea, alanine aminotransferase, aspartate aminotransferase, total protein, and albumin.

### Statistics and reproducibility

All the statistical analysis was performed with Graphpad Prism 9.4.1 software. The *t* test (and nonparametric tests) was used for data statistical analysis. *P* value < 0.05 was considered as statistically significant. Cell and animal imaging experiments were performed in biologically independent replicates with the *n* noted in figure captions.

## Supplementary Materials

### This PDF file includes:

Supplementry Text Figs. S1 to S48 Tables S1 and S2
