# Supplementary material for: A firm-push-to-open and light-push-to-lock strategy for a general chemical platform to develop activatable dual-modality NIR-II probes
Source: Sci Adv. 2024 Jun 14;10(24):eado2037. doi: 10.1126/sciadv.ado2037 (PMC11177897; doi:10.1126/sciadv.ado2037)
Supplement: Supplementary file 1 — Supplementry Text Figs. S1 to S48 Tables S1 and S2 [file sciadv.ado2037_sm.pdf]

Science Advances
AAAS

Supplementary Materials for

A firm-push-to-open and light-push-to-lock strategy for a general chemical platform to develop activatable dual-modality NIR-II probes

Lili Shen *et al.*

Corresponding author: Xin Li, lixin81@zju.edu.cn

Sci. Adv. **10**, eado2037 (2024)
DOI: 10.1126/sciadv.ado2037

The PDF file includes:

Supplementry Text
Figs. S1 to S48
Tables S1 and S2

1. General chemistry experimental

All chemicals were from commercial supplies and used without further purification except otherwise indicated. Reactions were run under a nitrogen atmosphere and monitored by thin-layer chromatography (TLC) carried out on TLC Silica gel 60 F254 plates supplied by MERCK, and UV light was used as the visualizing agent. All the crude products were purified by flash chromatography on 200-300 mesh or 40-80 mesh silica gel supplied by Qingdao Marine Chemical Factory. Anhydrous dichloromethane (DCM) was distilled from CaH_2. Anhydrous N, N-dimethylformamide (DMF) and anhydrous 1,4-dioxane were from Aladdin, China.

All full optical spectra in this article were the represented ones from three independent replicates and measured in pH 7.4 PBS (10 mM) under ambient temperature except otherwise noted. All intensity date were the mean ± SD of three replicated experiments.

^1H NMR spectra were obtained on a Bruker 500 Fourier transform spectrometer (500 MHz). ^{13}C NMR spectra were recorded on a Bruker 500 Fourier transform spectrometer (126 MHz). The residual solvent signals were used as references and the chemical shifts were converted to the TMS scale ($CDCl_3$, 7.26 ppm for ^1H NMR and 77.16 ppm for ^{13}C NMR; CD_3OD, 3.31 ppm for ^1H NMR and 49.00 ppm for ^{13}C NMR; $(CD_3)_2SO$, 2.50 ppm for ^1H NMR and 39.52 ppm for ^{13}C NMR). All chemical shifts were reported in parts per million (ppm) and coupling constants (*J*) in Hz. The following abbreviations were used to explain the multiplicities: d = doublet, t = triplet, m = multiplet, dd = doublet of doublets. High-resolution mass spectra (HRMS) for new compounds were measured on an Agilent 6224 TOF LC/MS spectrometer using ESI-TOF (electrospray ionization-time of flight) or a Waters TOFMS GCT Premier using ESI ionization. Liquid chromatography equipped with a low-resolution mass detector (LC-MS) was conducted on a SHIMADZU LCMS-2020 spectrometer. High Performance Liquid Chromatography (HPLC) was measured on an Agilent Technologies 1260 Infinity system. Absorption spectra were collected using a Hitachi U-3010 spectrophotometer or a Shimadzu UV-VIS-NIR (UV3600Plus+UV2700). Fluorescence spectra were measured on an Agilent Cary Eclipse Fluorescence Spectrophotometer or a steady-state, time-resolved photoluminescence spectrometer of FLS1000+FS5+Pico1000 (Edinburgh), or a C328 Odyssey CLx. Photoacoustic spectra were obtained with an iThera MosTiv256-TF. Confocal fluorescence imaging was obtained with a Leica STELLARIS 5 confocal microscope. *In vivo* fluorescence imaging was obtained with NIR-II fluorescence imaging system (Series II 808/900–1700, Suzhou NIR-Optics Technologies Co., Ltd.). *In vivo* Photoacoustic imaging was obtained with an iThera MosTiv256-TF.

2. Optical spectra analysis

2.1. Absorption spectra

Absorption spectra of compounds C1-C9 and D1-D3

To measure the absorption spectra of the compounds, their stock solutions (in DMSO, 5 mM) were individually diluted with PBS of indicated pH to 20 μM, shaked and then measured on a Hitachi U-3010 spectrophotometer.

Absorption spectra of compounds D4, BocNH-D4 and NH_2-D4

To measure the absorption spectra of the compounds, their stock solutions (in DMSO, 5 mM) were individually diluted with PBS of indicated pH to 20 μM, shaked, and then measured on a Shimadzu UV-VIS-NIR (UV3600Plus+UV2700).

Absorption spectra of H_2O_2-D4 and H_2S-D4 in response to H_2O_2 and H_2S

To measure the absorption spectra of probe **H_2O_2-D4** or **H_2S-D4** in response to analyte-treatment, the stock solution of probe **H_2O_2-D4** or **H_2S-D4** (in DMSO, 5 mM) was diluted with PBS of indicated pH (with 20% DMF) to 20 μM. Different concentrations of H_2O_2 or NaHS was added to this solution. After indicated incubation time at 37 °C, the spectra were measured on a Shimadzu UV-VIS-NIR (UV3600Plus+UV2700).

To test the selectivity of the probes, probe **H_2O_2-D4** or **H_2S-D4** (in DMSO, 5 mM) was diluted with PBS (with 20% DMF) to 20 μM. Aliquots of this solution were then treated with various analytes. The stock solutions of the analytes were prepared according to the below-described methods. After the indicated incubation time at 37 °C, the absorption spectra were collected on a Shimadzu UV-VIS-NIR (UV3600Plus+UV2700).

Preparation of various analytes for the selectivity experiment

H_2O_2 and NaClO: These solutions were prepared by diluting commercial H_2O_2 and NaClO solutions with PBS (10 mM, pH 7.4) to make 10 mM stock solutions.

***t*BHP:** *Tert*-Butylhydroperoxide (*t*BHP) was dissolved in PBS (10 mM, pH 7.4) 1 h before use to make a stock solution of 10 mM.

$ONOO^-$: To a vigorously stirred solution of $NaNO_2$ (0.6 M, 10 mL) and H_2O_2 (0.7 M, 10 mL) in deionized H_2O at 0 °C was added HCl (0.6 M, 10 mL), immediately followed by the rapid addition of NaOH (1.5 M, 20 mL). Excess hydrogen peroxide was removed by passing the solution through a short column of MnO_2. The concentration of $ONOO^-$ was determined by UV analysis with the extinction coefficient at 302 nm (ε= 1670 M^{-1} cm^{-1}). Aliquots of the solution were stored at -20 °C for use. Usually, a concentration around 12.5 mM could be obtained when the solution was prepared as described herein.

·NO: ·NO was administrated with DEA·NONOate as a donor. DEA·NONOate was prepared in 0.01 M NaOH to make a 10 mM stock solution.

·OH: ·OH was generated by Fenton reaction. To a solution of H_2O_2 (1.0 mM, 1.0 mL) in PBS (10 mM, pH 7.4) was added $FeSO_4$ solution (1.0 mM, 100 μL) at room temperature (stock solution 0.1 mM).

GSH, Cys, $FeSO_4$, $FeCl_3$, $ZnCl_2$, $CuSO_4$, KSCN, $NaSO_3$ NaHS and Hcy: These solutions were prepared by dissolving commercial GSH, Cys, $FeSO_4$, $FeCl_3$, $ZnCl_2$, $CuSO_4$, KSCN, $NaSO_3$ NaHS and Hcy powder with PBS to make 10 mM stock solutions.

2.2. Fluorescence spectra

Fluorescence spectra of compounds C1-C9 and D1-D3

To measure the fluorescence spectra of the compounds, their stock solutions (5 mM, in DMSO) were individually diluted with PBS of indicated pH to 10 μM, shaked and then measured on an Agilent Cary Eclipse Fluorescence Spectrophotometer.

Fluorescence spectra of compounds D4, BocNH- D4 and NH_2-D4

To measure the fluorescence spectra of the compounds, their stock solutions (5 mM, in DMSO) were individually diluted with PBS of indicated pH to 10 μM, shaked, and then measured on a steady-state and time-resolved photoluminescence spectrometer of FLS1000+FS5+Pico1000 (Edinburgh).

Fluorescence spectra of H_2O_2-D4 and H_2S-D4 in response to H_2O_2 and H_2S

To measure the fluorescence spectra of probe H_2O_2-D4 or H_2S-D4 to the treatment of H_2O_2 and H_2S, the stock solution of probe H_2O_2-D4 or H_2S-D4 (5 mM, in DMSO) was diluted with PBS (with 20% DMF) to 10 μM. Different concentrations of H_2O_2 or H_2S were added. After incubation for the indicated time at 37 °C, the spectra were measured on a steady-state and time-resolved photoluminescence spectrometer of FLS1000+FS5+Pico1000 (Edinburgh).

Limit of detection determination for H_2O_2-D4 and H_2S-D4

To measure the limit of detection for probe H_2O_2-D4 or H_2S-D4, the stock solution of probe H_2O_2-D4 or H_2S-D4 (5 mM, in DMSO) was diluted with PBS (with 20% DMF) to 10 μM. Different concentrations of H_2O_2 or H_2S were added. After 1 h of incubation at 37 °C, the spectra were measured on C328 Odyssey CLx. The linear regression between fluorescence intensity and analyte concentrations was plotted to get the slope K. Meanwhile, at least 20 replicates of the blank probe solutions were prepared in the same way and were measured to calculate the standard derivation of the fluorescence intensity S_B. The limit of detection was calculated by $3S_B/K$.

Quantum yield determination

To determine the quantum yields of C1-C9, silicon rhodamine (Φ_{standard} = 0.310 in PBS of pH 7.4) was used as a standard. For C1-C9 and silicon rhodamine, the absorption spectra were measured within an absorbance intensity range of 0.0100 to 0.0500. The quantum yields were calculated according to the equation:

$$\Phi u = \Phi s \cdot \frac{\Sigma F\ u}{\Sigma F\ s} \cdot \frac{As}{Au} \cdot \left(\frac{Gu}{Gs}\right)^2$$

Where Φ is the quantum yield, ΣF is the integrated fluorescence intensity, A is the absorbance at the excitation wavelength, and G represents the refractive index of the solvent.

2.3. Photoacoustic spectra

Photoacoustic spectra of compounds D1-D4

To measure the photoacoustic spectra of compounds D1-D4, the stock solutions of compounds D1-D4 (5 mM, in DMSO) were individually diluted with PBS of indicated pH to 20 μM, shaked, and then measured on an iThera MosTiv256-TF instrument.

Photoacoustic spectra of compound BocNH-D4 and NH_2-D4

To measure the photoacoustic spectra of BocNH-D4 and NH_2-D4, the stock solutions of BocNH-D4 and NH_2-D4 (5 mM, in DMSO) were individually diluted with PBS of indicated pH to 20 μM, shaked, and then measured on an iThera MosTiv256-TF instrument.

3. HPLC methods

Testing the stability of NH_2-D4 in PBS towards NaClO and $ONOO^-$ treatment

The stock solution of compound NH_2-D4 (5 mM, in DMSO) was diluted with PBS (pH 7.4) to 100 μM. Then NaClO or $ONOO^-$ (2 eq and 20 eq) was added. The mixture was stored at room temperature and monitored by HPLC in a time-lapse way. HPLC was performed using an Agilent 1260 Infinity II instrument equipped with a C18 reversed-phase column (GL Sciences ODS-3, 4.6 × 250 mm, 5 μm) with a flow rate of 1.0 mL/min; detected under UV absorbance at 480 nm, the

column temperature was 25 °C, eluted with water (0.1% formic acid) as the A phase and methanol as the B phase.

Testing the storage stability of H_2O_2-D4 or H_2S-D4 in PBS

The stock solution of H_2O_2-D4 or H_2S-D4 (5 mM, in DMSO) was diluted with PBS (pH 7.4) to 100 μM, shaked, measured by HPLC in a time-lapsed way for 16 h. HPLC analysis was performed as above described.

HPLC traces of probe H_2S-D4 before and after sensing H_2S

The probe stock solution (5 mM, in DMSO) was diluted in methanol to make a 100 μM solution. Then NaHS (2.5 eq) was added. The mixture was stored at room temperature and monitored by HPLC analysis every 15 min for 150 min. Analytical HPLC was performed using an Agilent 1260 Infinity II machine with a flow rate of 0.3 mL/min; detection under UV absorbance was performed at a wavelength of 365 nm, the column temperature was 25 °C, eluting with water (0.1% formic acid) as the A phase and methanol as the B phase.

4. LCMS methods

LCMS analysis of probe H_2O_2-D4 before and after sensing H_2O_2

The probe stock solution (5 mM, DMSO) was diluted with methanol to make a 100 μM solution. Then H_2O_2 (5 eq) was added. The mixture was stored at room temperature and analyzed by LCMS every 10 min for 1 h. Analytical LCMS was performed using a Shimadzu LC/MS-2020 instrument.

5. Supplementary tables and figures

Compound name	ε_{max} /M^{-1} cm^{-1} (at 680 nm)	λ_{em} /nm (in NIR)	Φ_f [a]
**BocNH-C1**	14 178	720	0.00365
**NH_2-C1**	16 892	720	0.00604
**BocNH-C2**	185	-	-
**NH_2-C2**	427	-	-
**BocNH-C3**	8 186	720	0.00148
**NH_2-C3**	18 761	720	0.00996
**BocNH-C4**	14 983	720	0.00077
**NH_2-C4**	18 409	720	0.00102
**BocNH-C5**	4 057	720	0.00089
**NH_2-C5**	10 555	720	0.00609
**BocNH-C6**	7 706	720	0.00100
**NH_2-C6**	19 468	720	0.00376
**BocNH-C7**	803	-	-
**NH_2-C7**	493	-	-
**BocNH-C8**	105	-	-
**NH_2-C8**	1 180	-	-
**BocNH-C9**	163	-	-
**NH_2-C9**	162	-	-

Table S1. Photophysical data of C1−9 in PBS. a) Φ_f is the relative fluorescence quantum yield estimated by using Si-rhodamine (Φ_f = 0.310 in PBS) as a standard.

Group	electron density
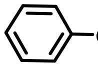 COOH	-0.341
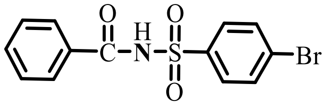	-0.274
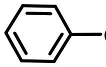 $CONHSO_2CH_3$	-0.249
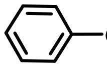 $CONHSO_2N(CH_3)_2$	-0.237
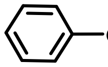 $CONHSO_2CF_3$	-0.125
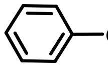 $CONHCH_3$	-0.105
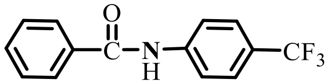	-0.099
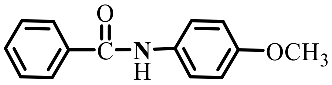	-0.099
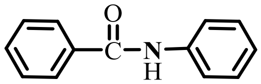	-0.087

Table S2. Electronic density of the oxygen or nitrogen atoms on the *ortho*-carboxy groups. Data were calculated *via* Gaussian computation.

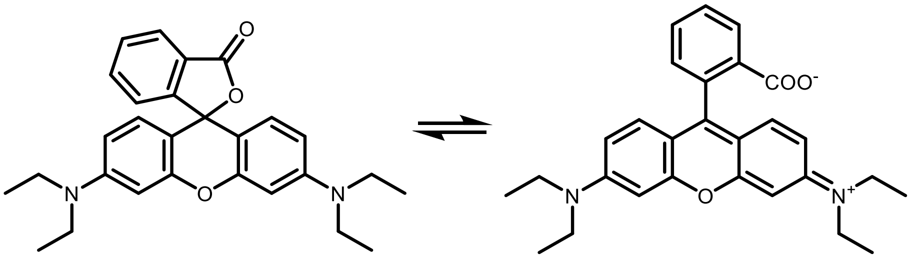

Fig. S1. Structures of rhodamine B in its spirocyclization-zwitterion equilibria.

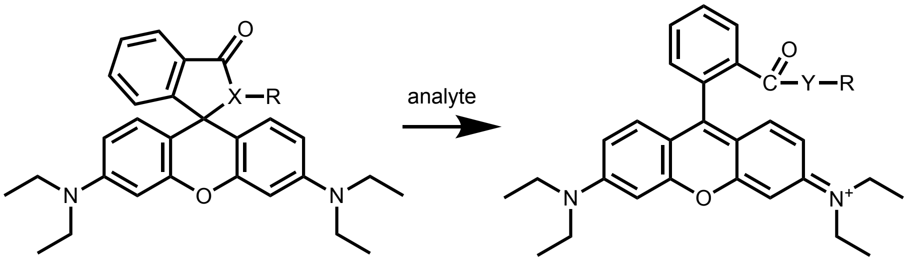

Fig. S2. No-wash probes for bio-sensing based on rhodamine spirocyclization-zwitterion equilibrium.

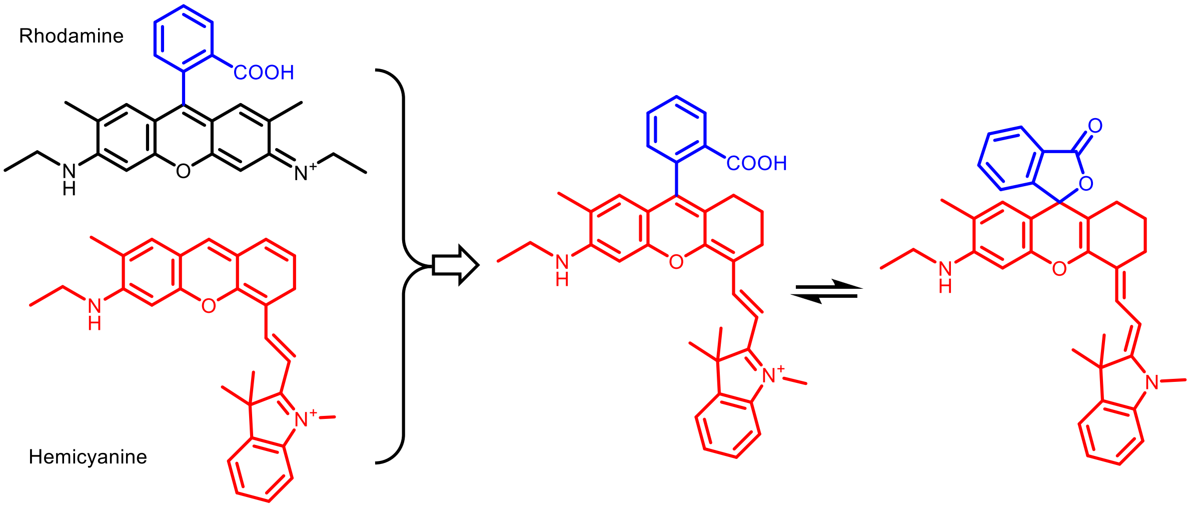

Fig. S3. Changsha (CS) NIR dyes and its equilibrium between the spirocyclic and zwitterionic form.

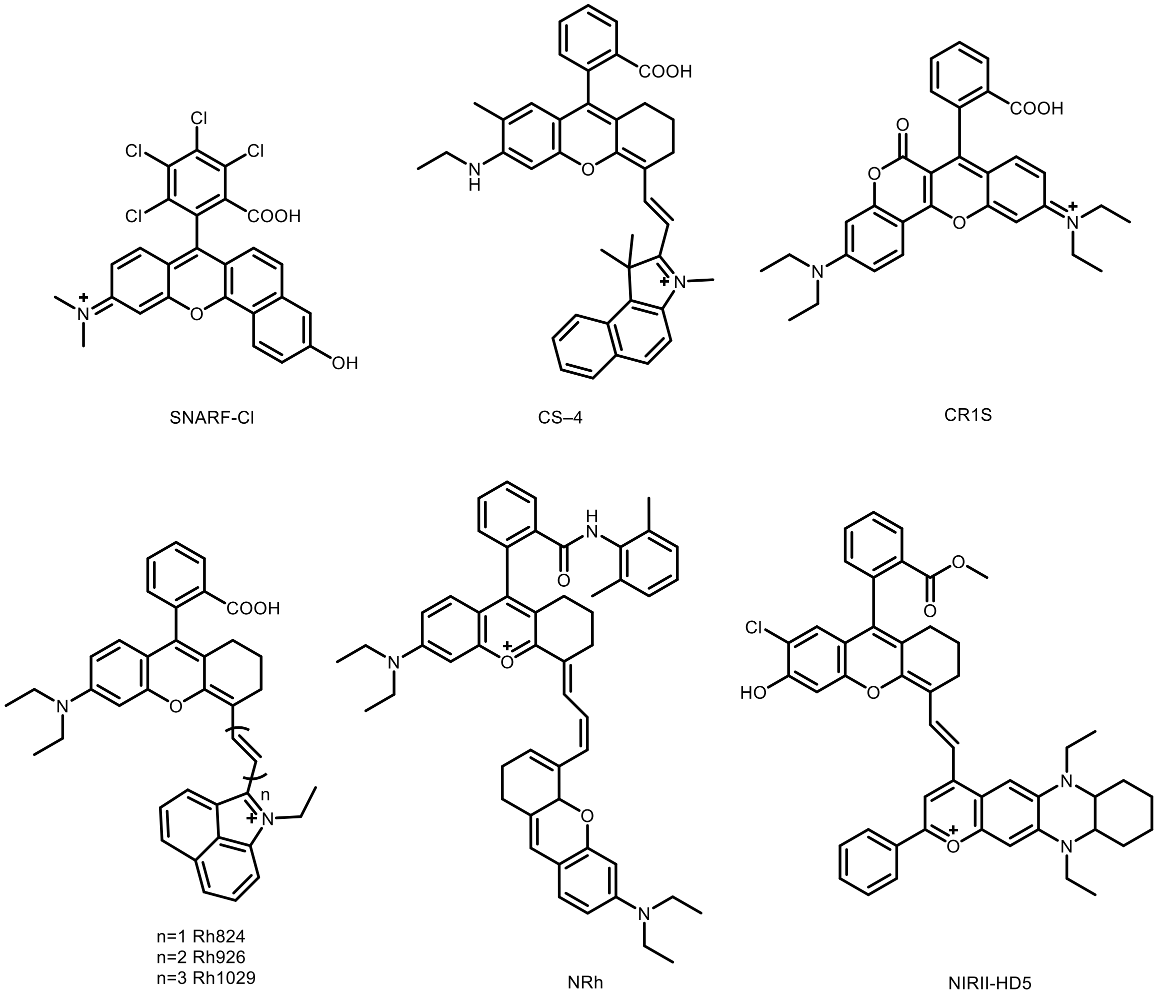

Fig. S4. Representative structures of cyanine–rhodamine hybrids. (*24, 42, 43, 76*)

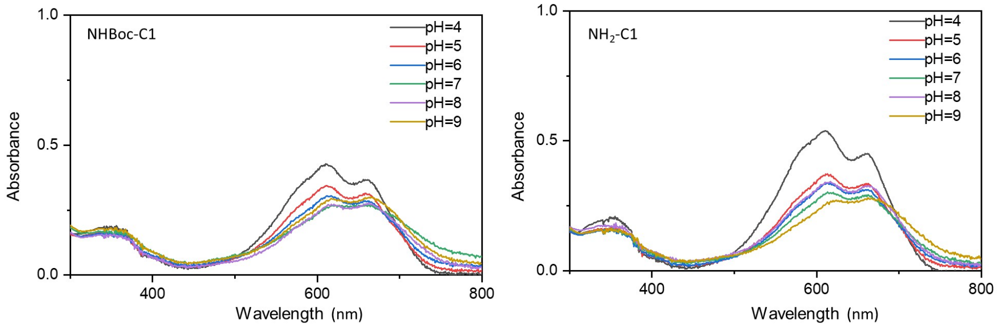

Fig. S5. Absorption spectra of NHBoc-C1 and NH$_2$-C1 (20 μM) in PBS of indicated pH.

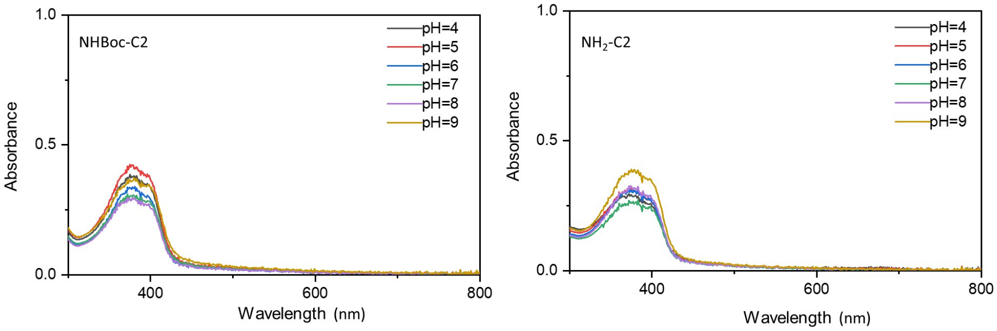

Fig. S6. Absorption spectra of NHBoc-C2 and NH$_2$-C2 (20 μM) in PBS of indicated pH.

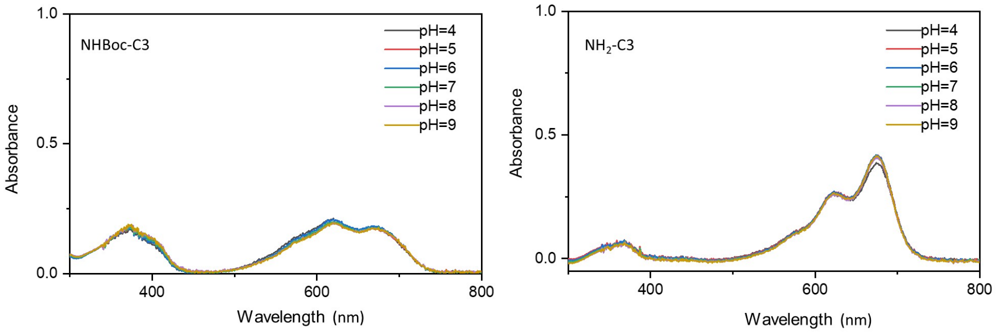

Fig. S7. Absorption spectra of NHBoc-C3 and NH$_2$-C3 (20 μM) in PBS of indicated pH.

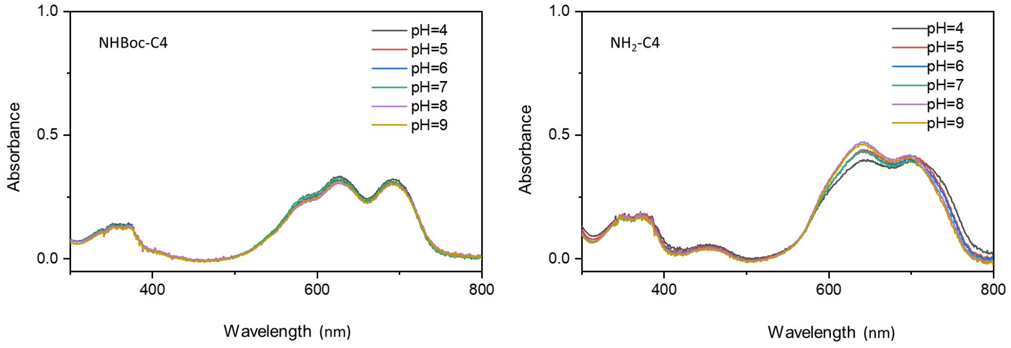

Fig. S8. Absorption spectra of NHBoc-C4 and NH_2-C4 (20 µM) in PBS of indicated pH.

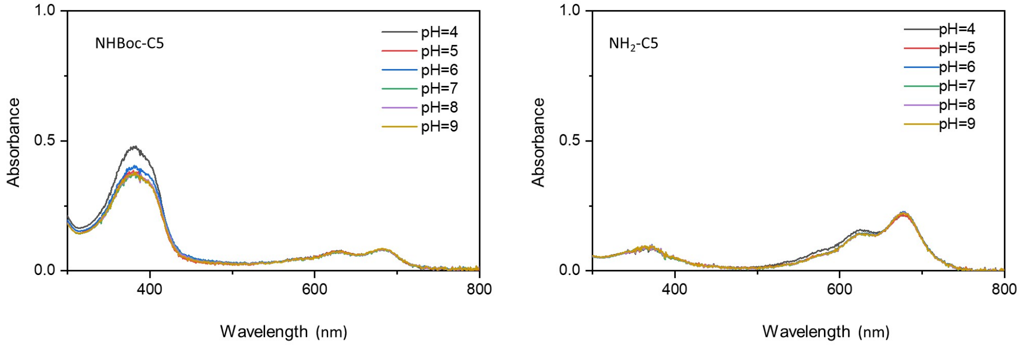

Fig. S9. Absorption spectra of NHBoc-C5 and NH_2-C5 (20 µM) in PBS of indicated pH.

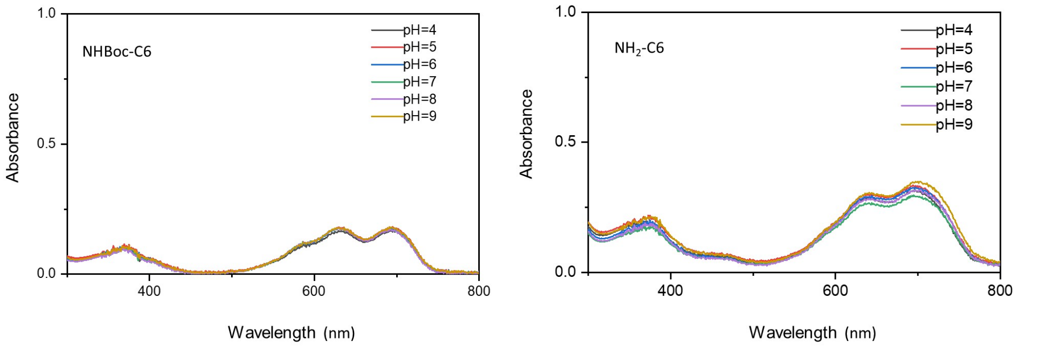

Fig. S10. Absorption spectra of NHBoc-C6 and NH_2-C6 (20 µM) in PBS of indicated pH.

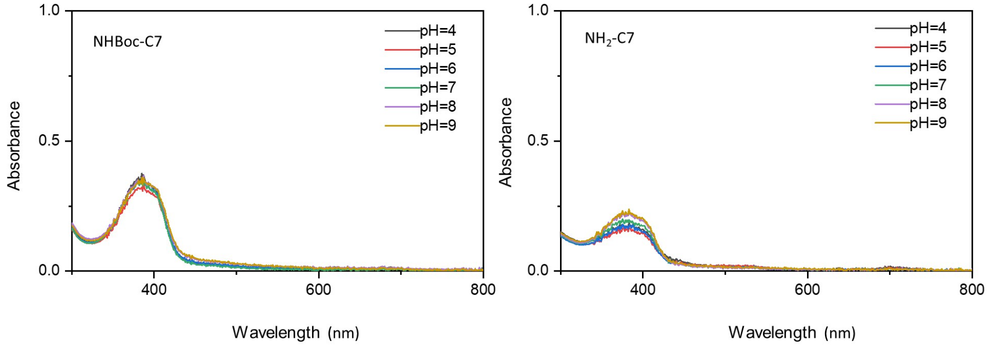

Fig. S11. Absorption spectra of NHBoc-C7 and NH$_2$-C7 (20 μM) in PBS of indicated pH.

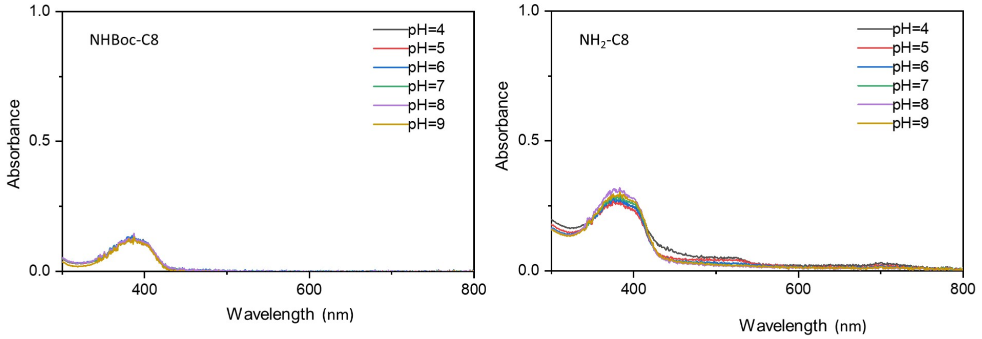

Fig. S12. Absorption spectra of NHBoc-C8 and NH$_2$-C8 (20 μM) in PBS of indicated pH.

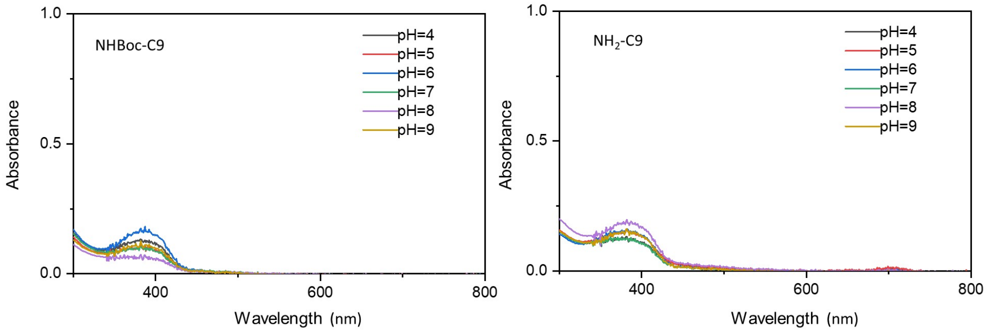

Fig. S13. Absorption spectra of NHBoc-C9 and NH$_2$-C9 (20 μM) in PBS of indicated pH.

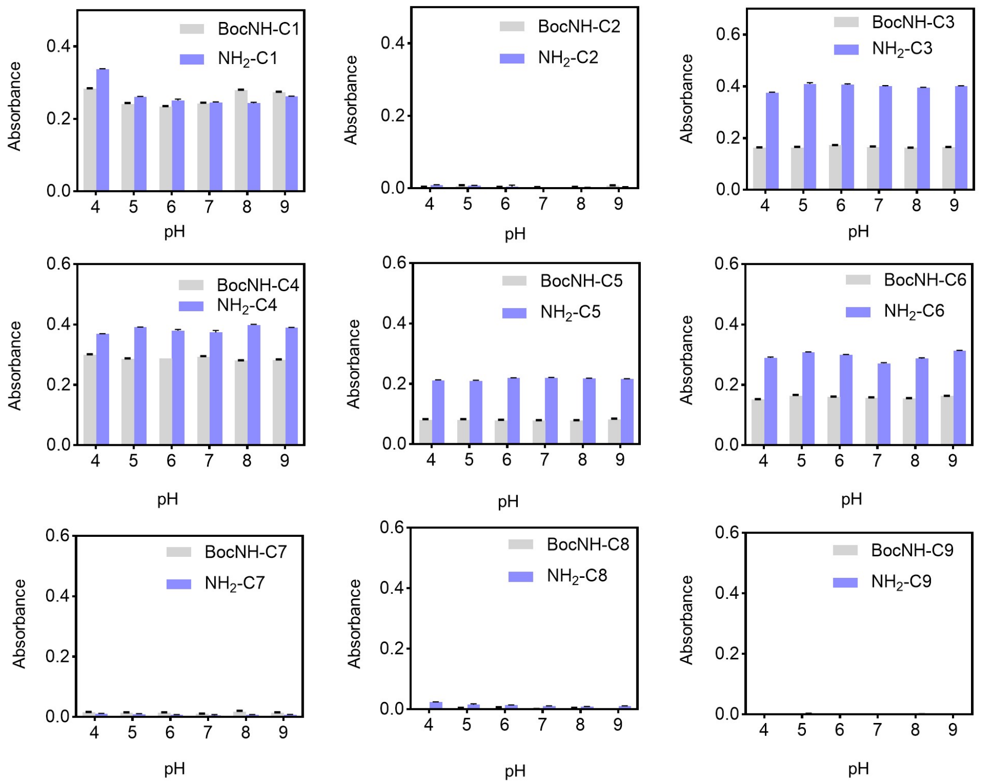

Fig. S14. Absorption intensity of C1 - C9 (20 μM) at 680 nm in PBS of indicated pH.

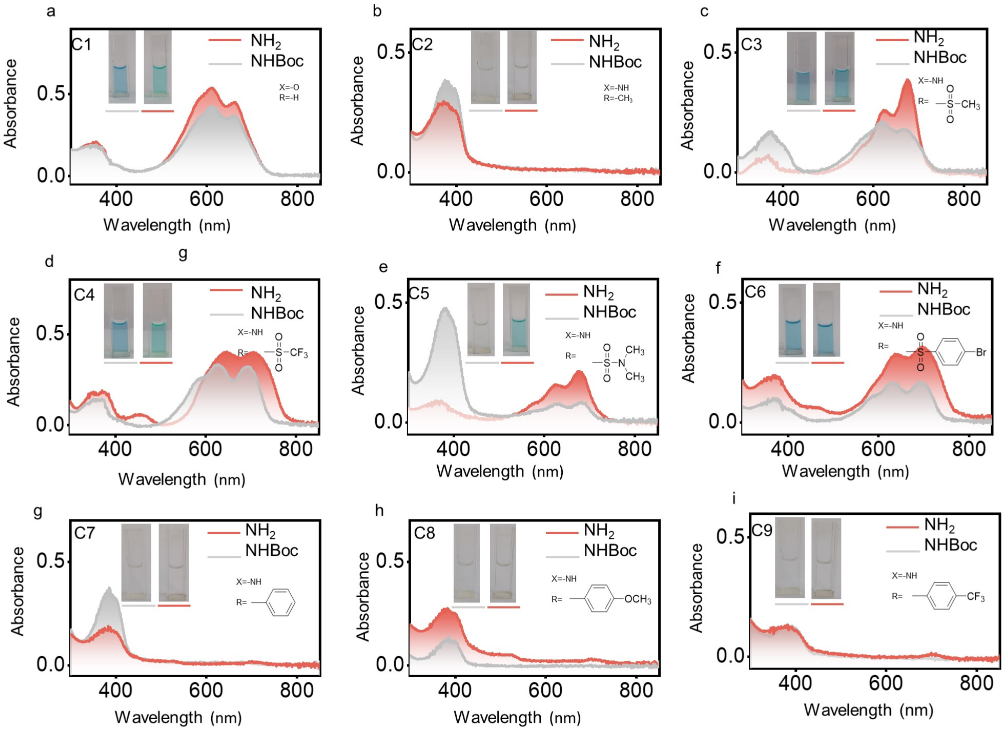

Fig. S15. Absorption spectra of C1 - C9 (20 μM) in PBS of pH 7.4.

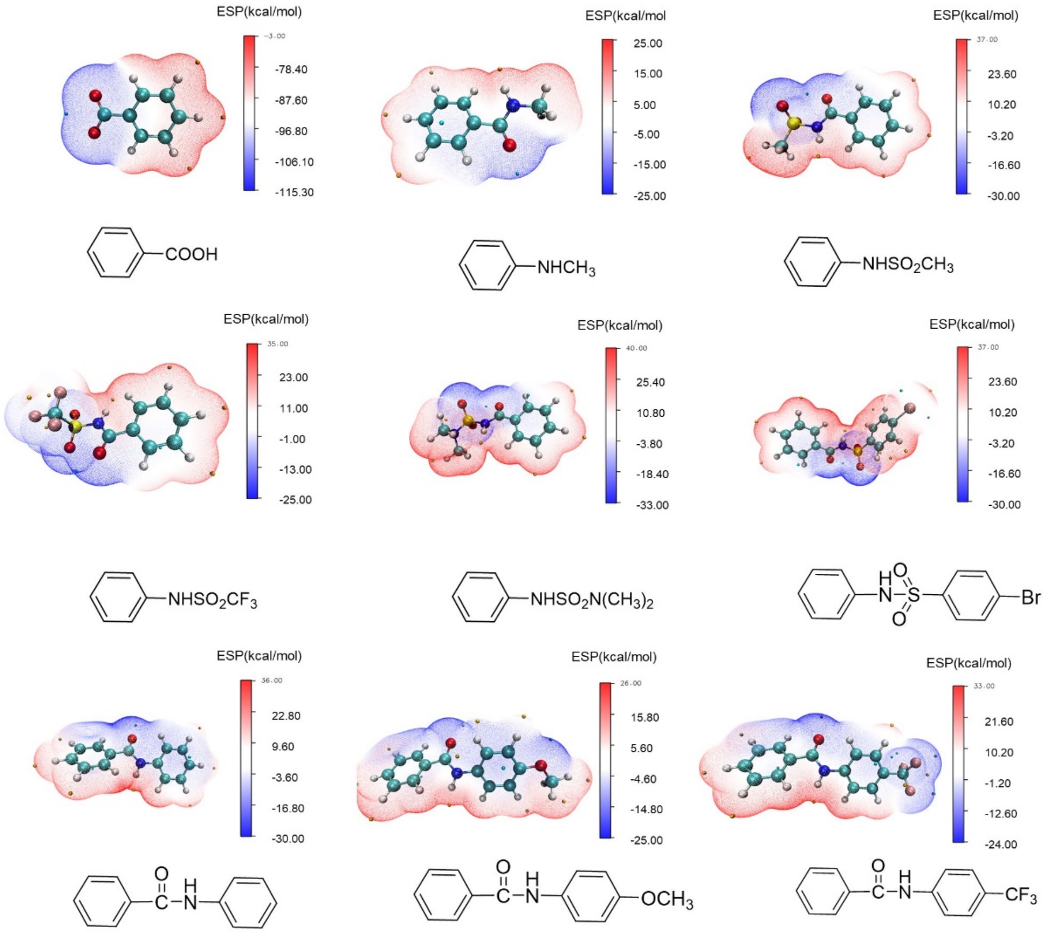

Fig. S16. Electronic density of the oxygen or nitrogen atoms at the *ortho-* carboxyl groups. Date were calculated *via* Gaussian computation.

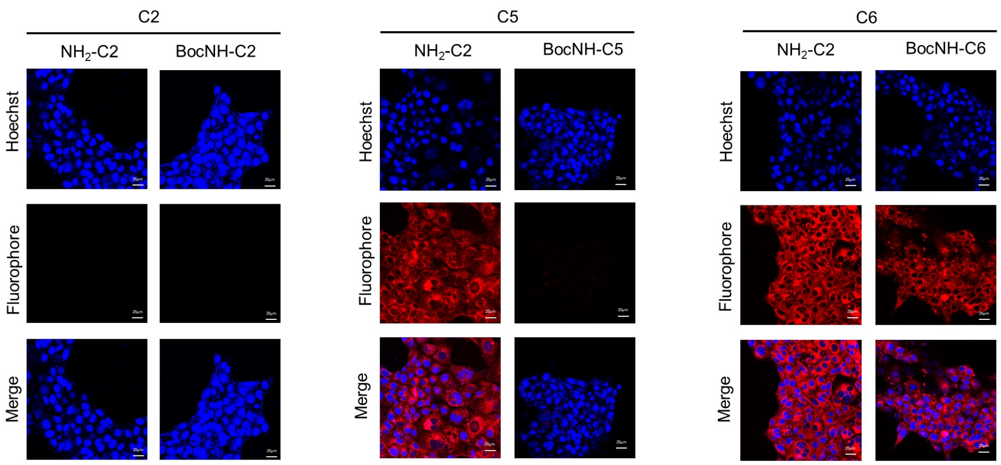

Fig. S17. Representative images of 4T1 cells after being stained with 5 μM C2-NH_2/-NHBoc, C5-NH_2/-NHBoc, C6-NH_2/-NHBoc for 90 min at 37 °C. Scale bar: 25 μm.

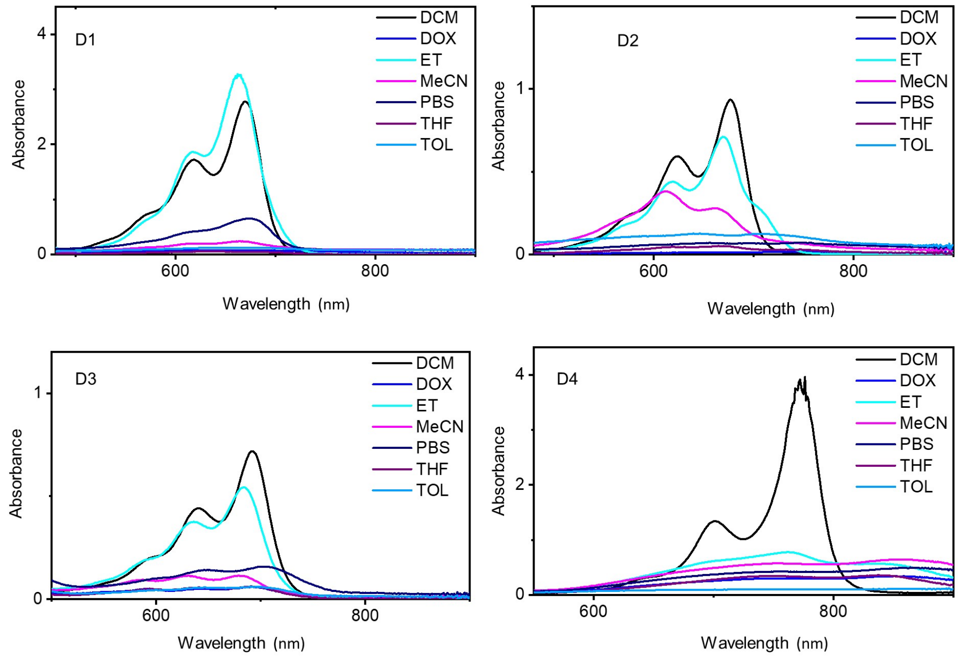

Fig. S18. Absorption spectra of D1-D4 in different solutions at 20 μM.

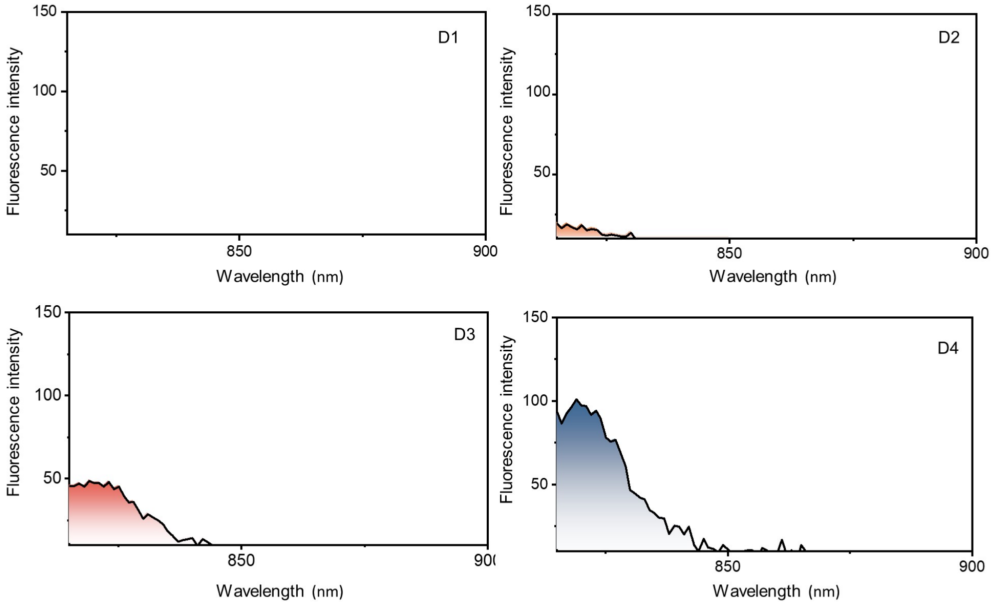

Fig. S19. Fluorescence spectra of D1-D4 (10 μM) in the NIR range in PBS of pH 7.4.

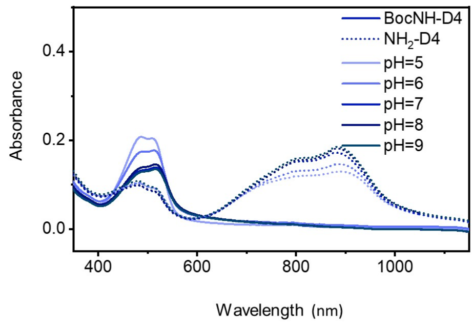

Fig. S20. Absorption spectra of BocNH-D4 and NH_2-D4 (20 μM) in PBS of indicated pH.

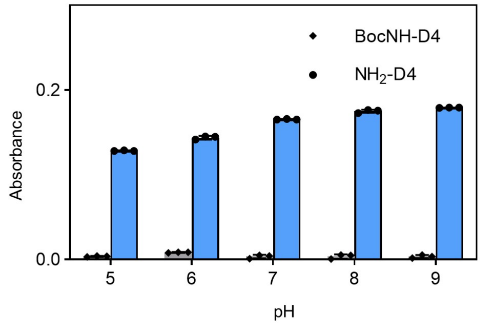

Fig. S21. Absorption intensity of BocNH-D4 and NH_2-D4 at 900 nm in PBS of indicated pH at 20 μM.

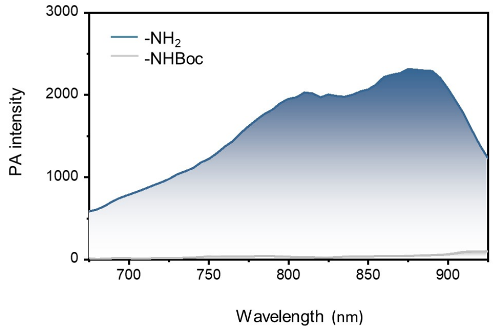

Fig. S22. PA spectra of BocNH-D4 and NH_2-D4 (20 μM) in PBS of pH 7.4.

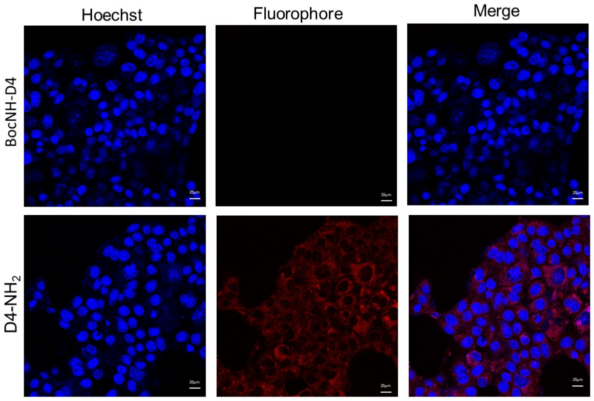

Fig. S23. Representative images of 4T1 cells after being stained with 5 μM BocNH-D4 or NH_2-D4 for 90 min at 37 °C. Scale bar: 25 μm.

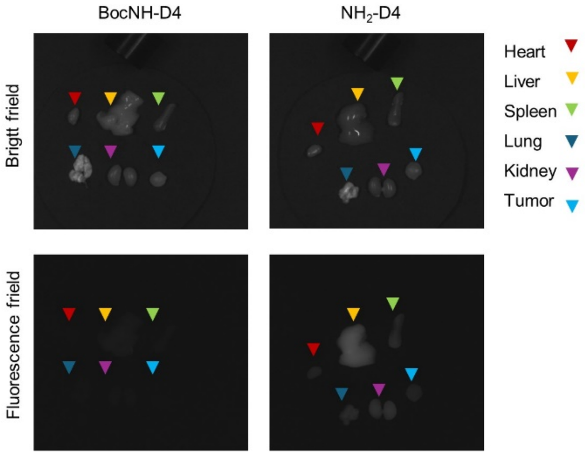

Fig. S24. Fluorescence imaging of organs post i.v. injection of either NHBoc-D4 or NH_2-D4.

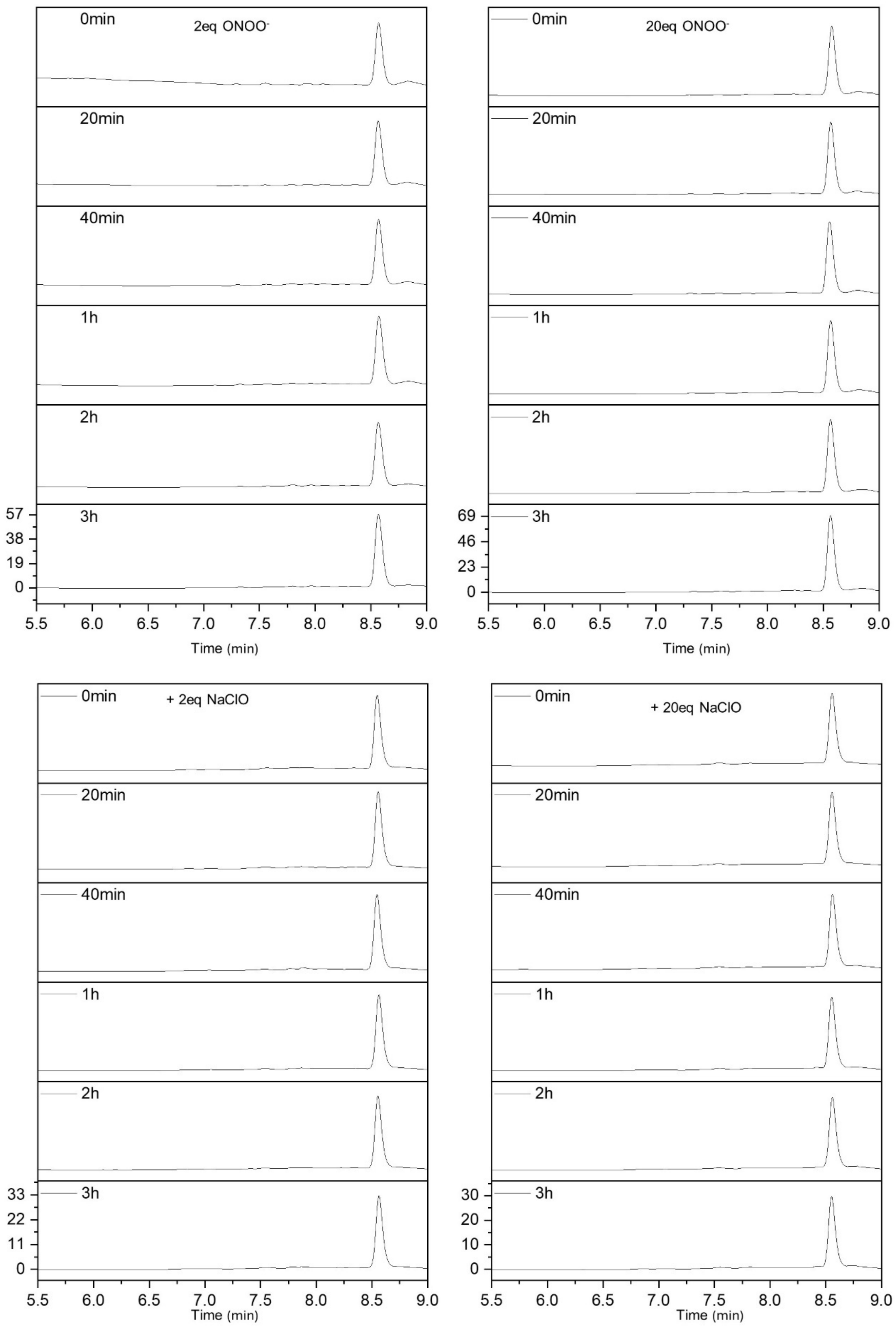

Fig. S25. HPLC traces of NH_2-D4 in PBS before and after the treatment of $ONOO^-$ or NaClO.

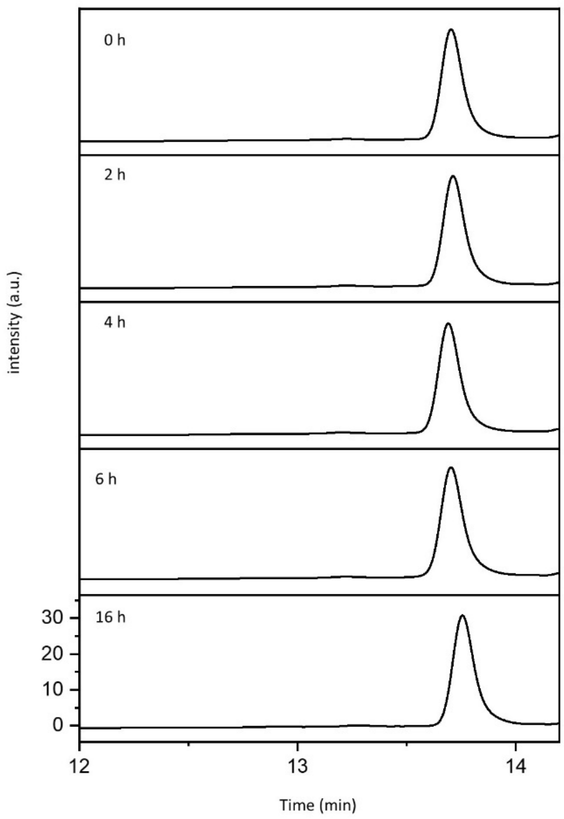

Fig. S26. Storage stability of H_2O_2-D4 in PBS of pH 7.4 as measured by HPLC.

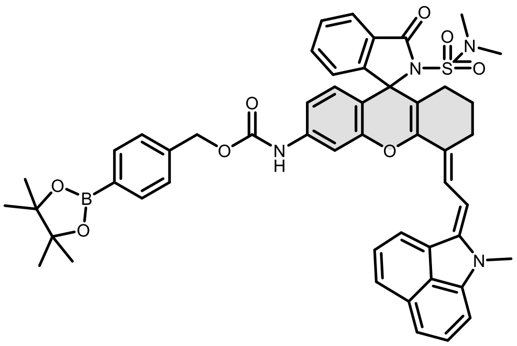

H_2O_2-D4

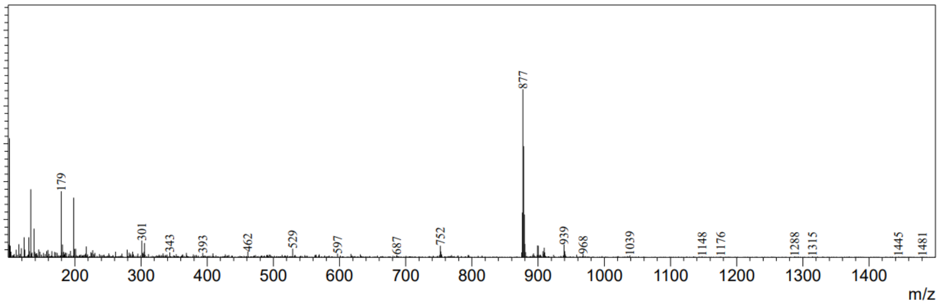

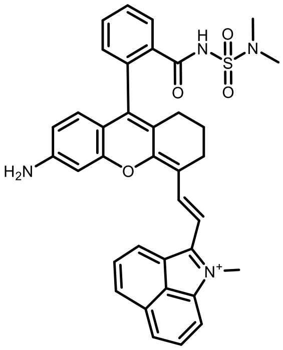

NH_2-D4

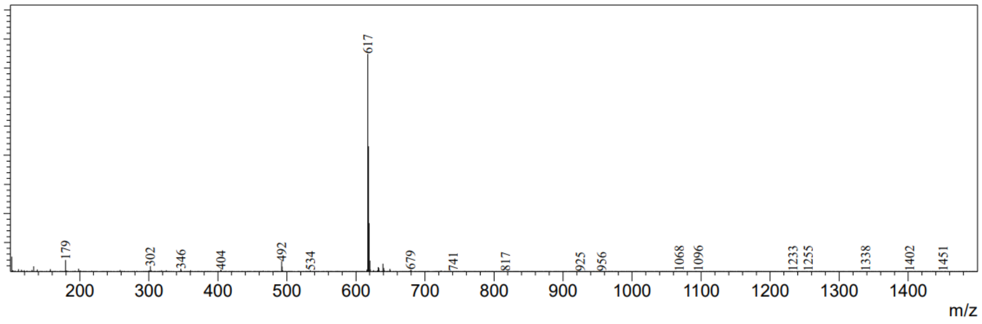

Fig. S27. MS data of H_2O_2-D4 before/after the treatment of H_2O_2.

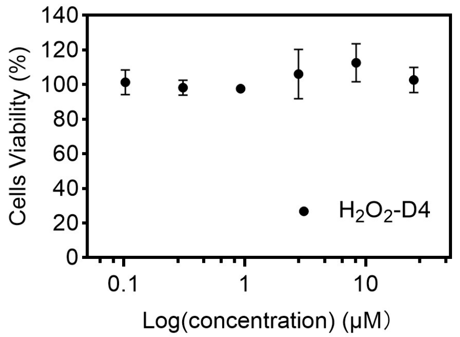

Fig. S28. Cytotoxicity of H_2O_2-D4.

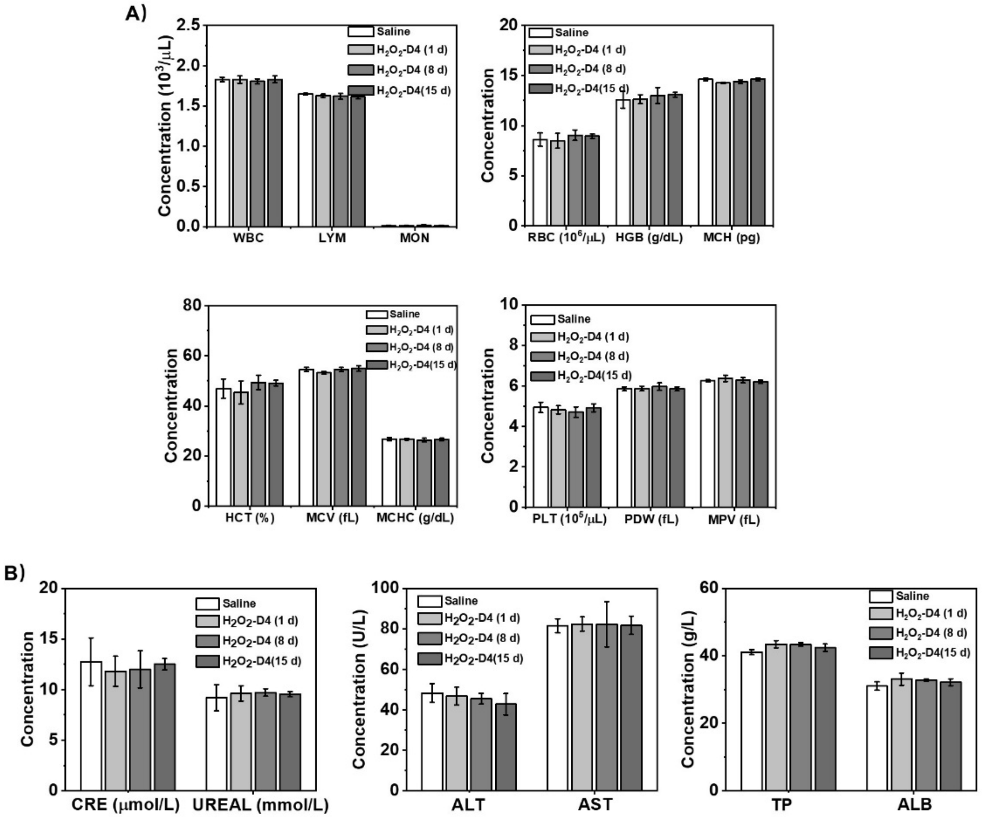

Fig. S29. Bio-safety assay results of H_2O_2-D4. Mice (n=5 per group) were treated with saline or **H_2O_2-D4**. (A) Blood routine analysis was performed after the indicated time (1, 8, and 15 days post-injection). Data are means ± SD. (B) Blood biochemical analysis was performed after the indicated time (1, 8, and 15 days post-injection). Data are means ± SD.

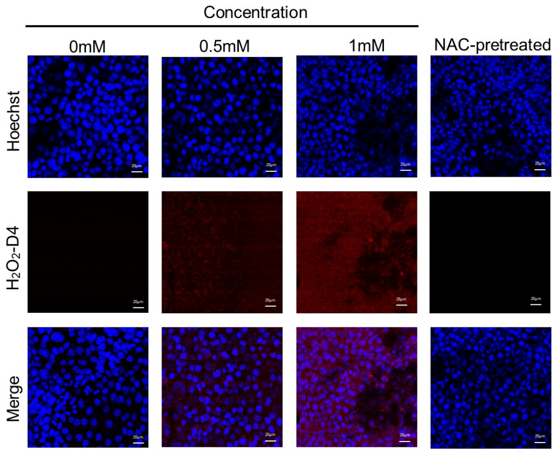

Fig. S30. Representative images of 4T1 cells first treated with different concentrations of H_2O_2 for 3 h and then stained with H_2O_2-D4 for 90 min at 37 °C. Scale bar: 25 μm.

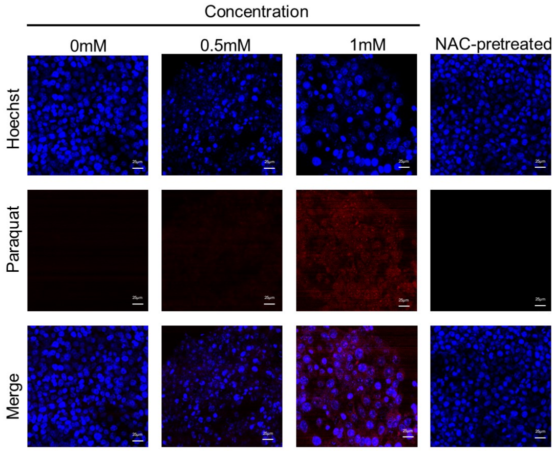

Fig. S31. Representative images of 4T1 cells first treated with different concentrations of paraquat for 3 h and then stained with H_2O_2-D4 for 90 min at 37 °C. Another group was pretreated with 100 μM NAC for 1h before being treated with paraquat (1 mM). Scale bar: 25 μm.

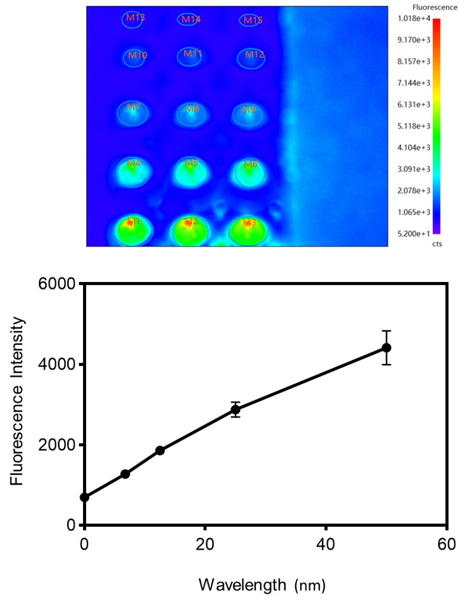

Fig. S32. Fluorescence intensity profile of NH_2 -D4 at different concentrations (0-50 µM) in PBS of pH 7.4. as measured on the BLT NIR-30F system with excitation at 808 nm.

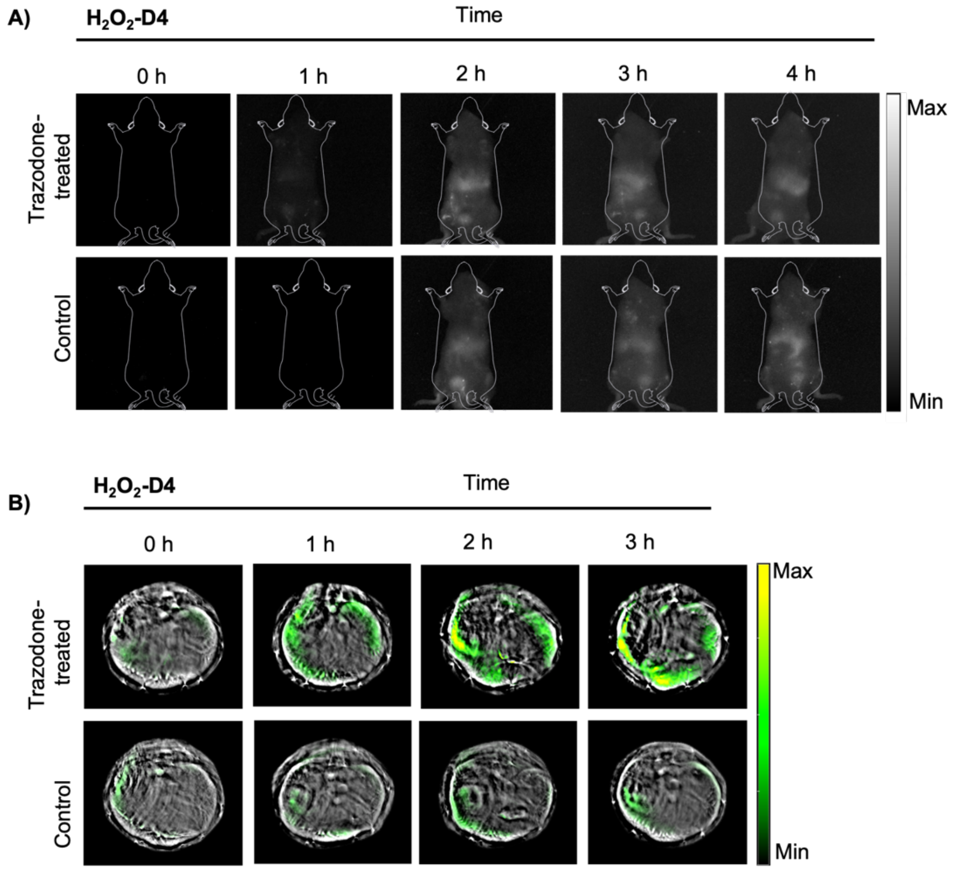

Fig. S33. Activatable imaging of H_2O_2 in mice with liver injury. (A) Whole-body fluorescence and (B) Liver cross-section PA imaging of mice with liver injury before and after I. V. injection of the probe.

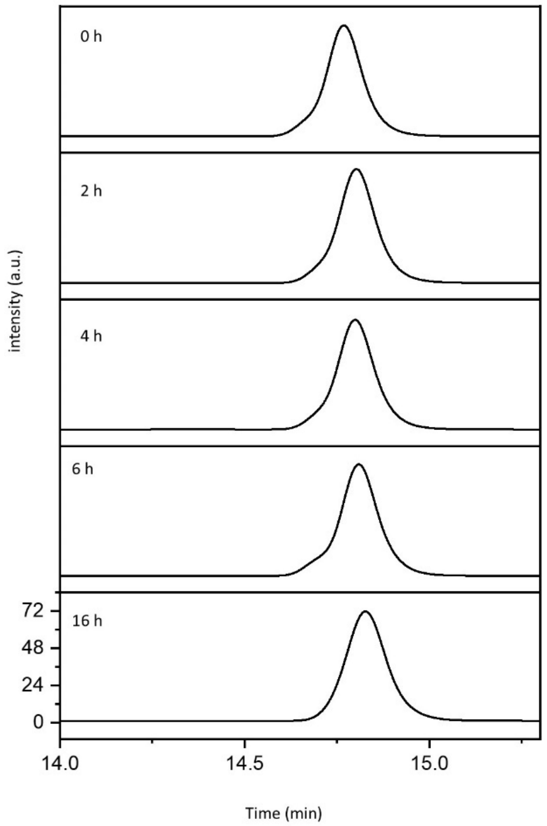

Fig. S34. Storage stability of H_2S-D4 in PBS of pH 7.4 as measured by HPLC.

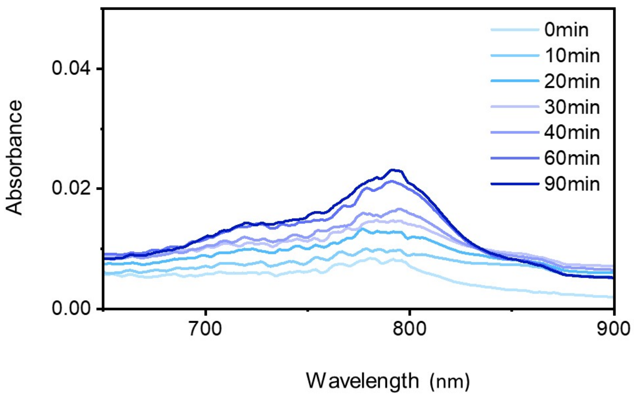

Fig. S35. Absorption spectra of H_2S-D4 (20 µM) after being treated with NaHS (50 µM) for the indicated time.

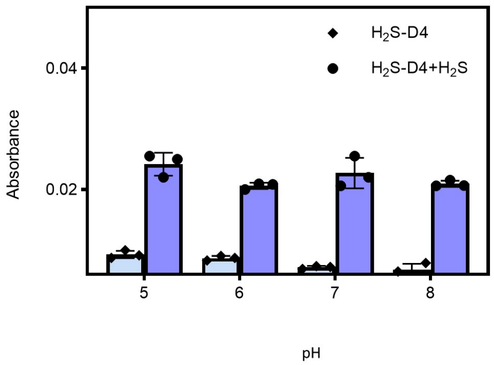

Fig. S36. The absorption intensity of H_2S-D4 at 900 nm before and 90 min after interacting with H_2S (50 µM) in PBS of indicated pH.

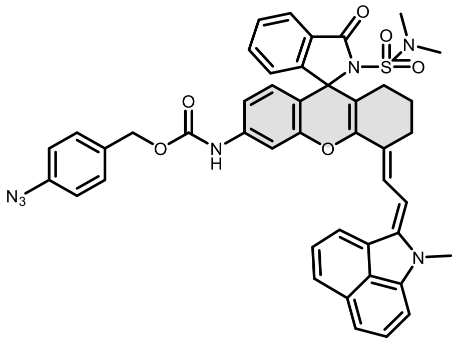

H_2S-D4

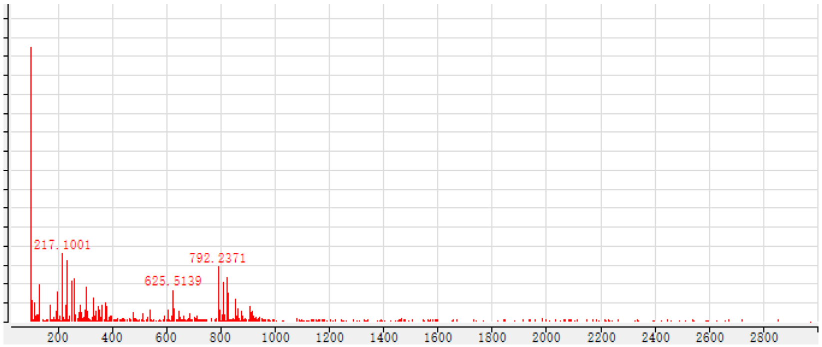

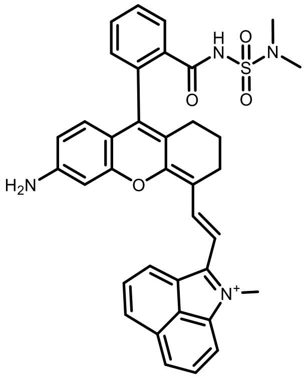

NH_2-D4

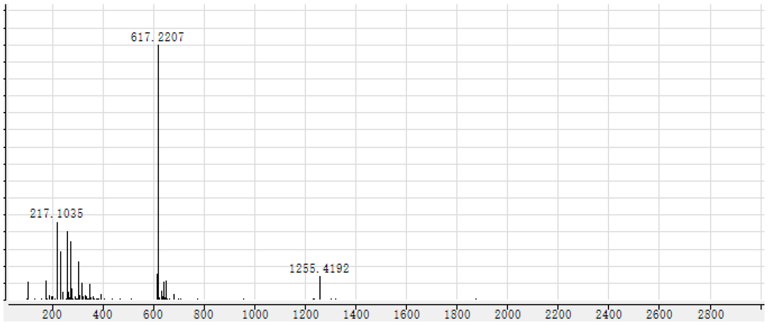

Fig. S37. HRMS data of H_2S-D4 before/after the treatment of NaHS.

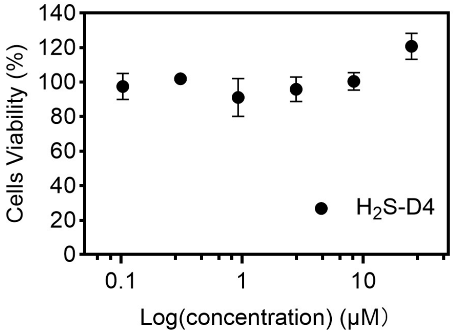

Fig. S38. Cytotoxicity H_2S-D4 as measured by the CCK8 assay.

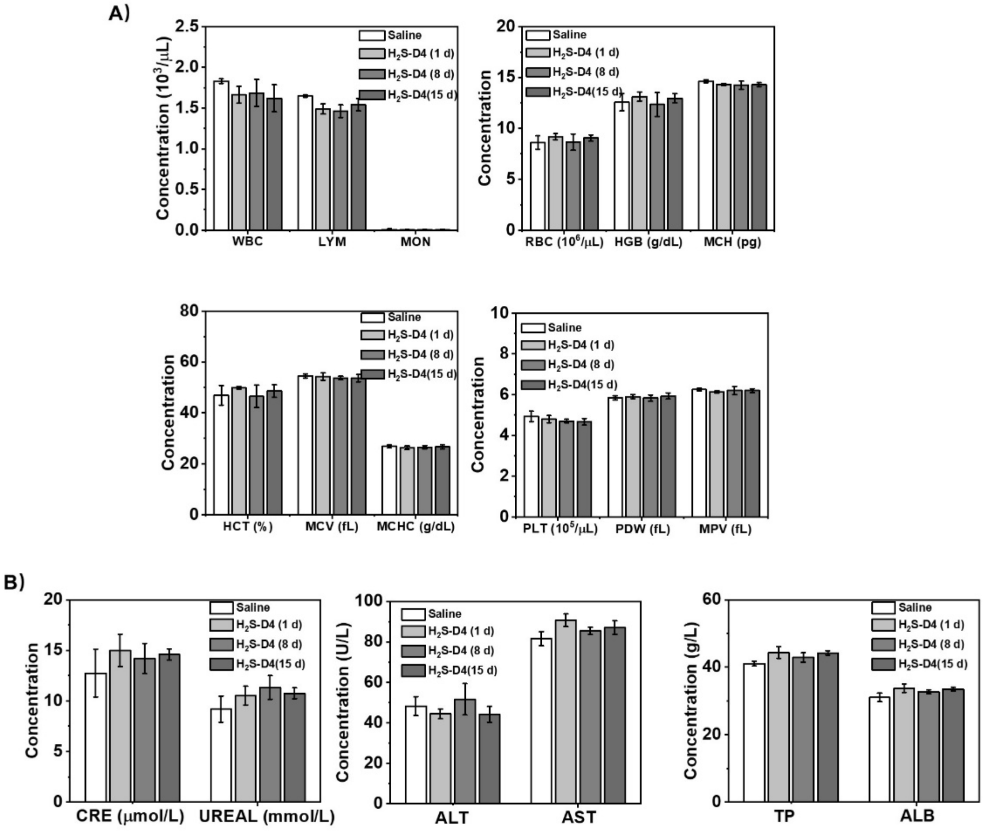

Fig. S39. Bio-safety assay results of H_2S-D4. Mice (n=5 per group) were treated with saline or **H_2S-D4**. (A) Blood routine analysis was performed after the indicated time (1, 8, and 15 days post-injection). Data are means ± SD. (B) Blood biochemical analysis was performed after the indicated time (1, 8, and 15 days post-injection). Data are means ± SD.

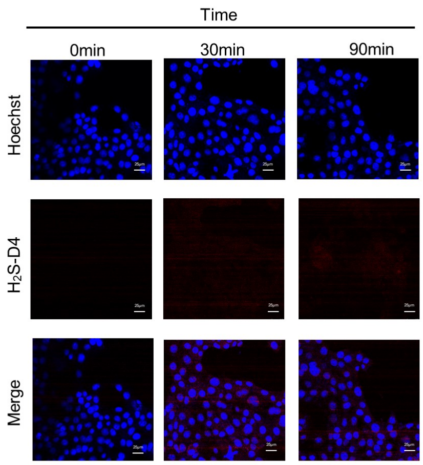

Fig. S40. Representative images of 4T1 cells first treated with 1 mM NaHS for 3 h and then stained with H_2S-D4 for different times at 37 ^oC. Scale bar: 25 μm.

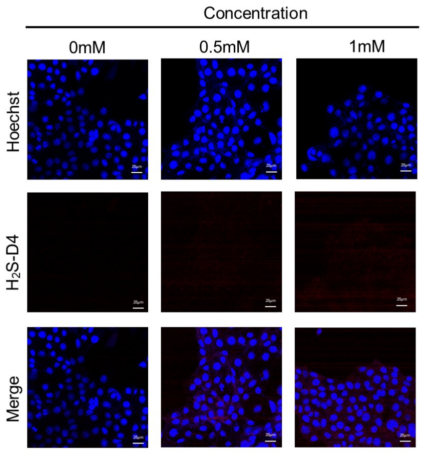

Fig. S41. Representative images of 4T1 cells first treated with different concentrations of H_2S for 3 h and then stained with H_2S-D4 for 90 min at 37 ^oC. Scale bar: 25 μm.

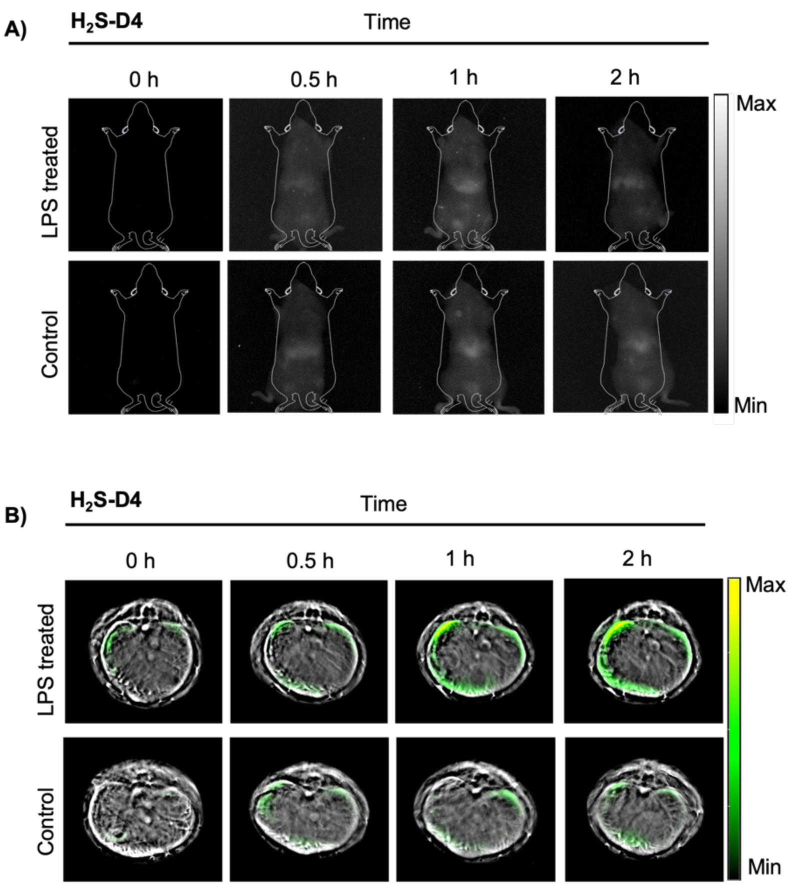

Fig. S42. Activatable imaging of H_2S in mice with LPS-induced acute liver inflammation. (A) Whole-body fluorescence and (B) Liver cross-section PA imaging of mice with LPS-induced acute liver inflammation before and after I. V. injection of the probe.

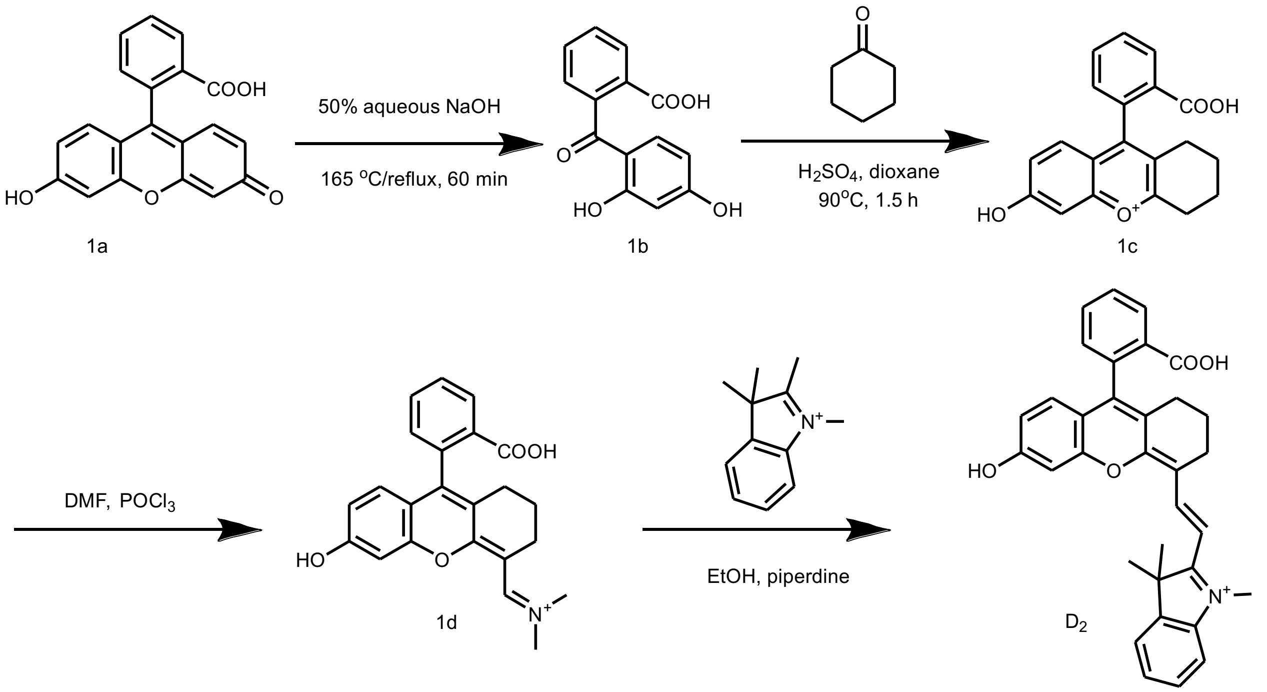

Fig. S43. Synthesis of compound D2.

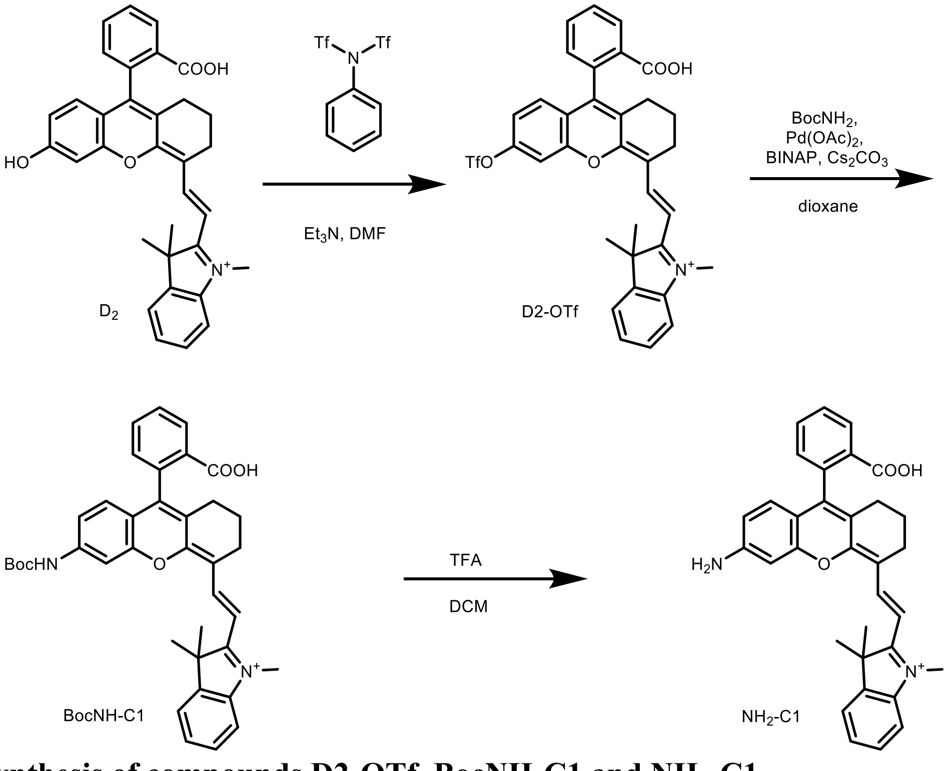

Fig. S44. Synthesis of compounds D2-OTf, BocNH-C1 and NH_2-C1.

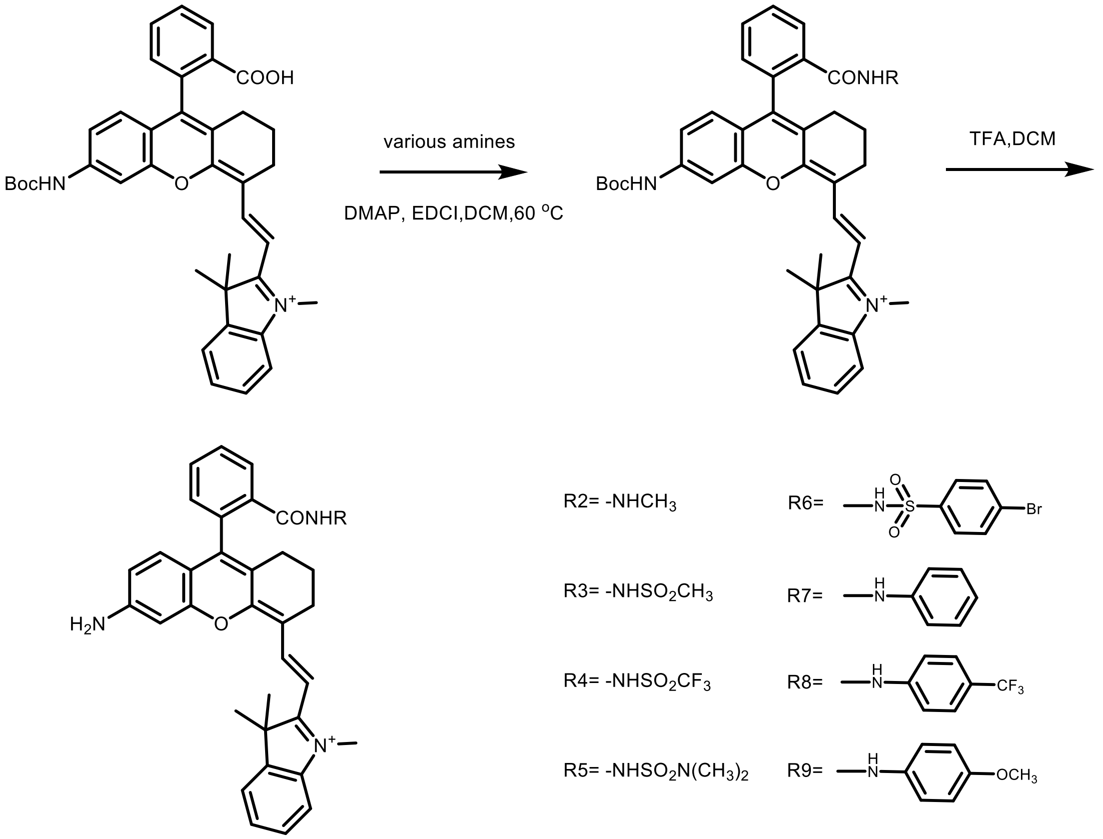

Fig. S45. Synthesis of compounds C2-C9.

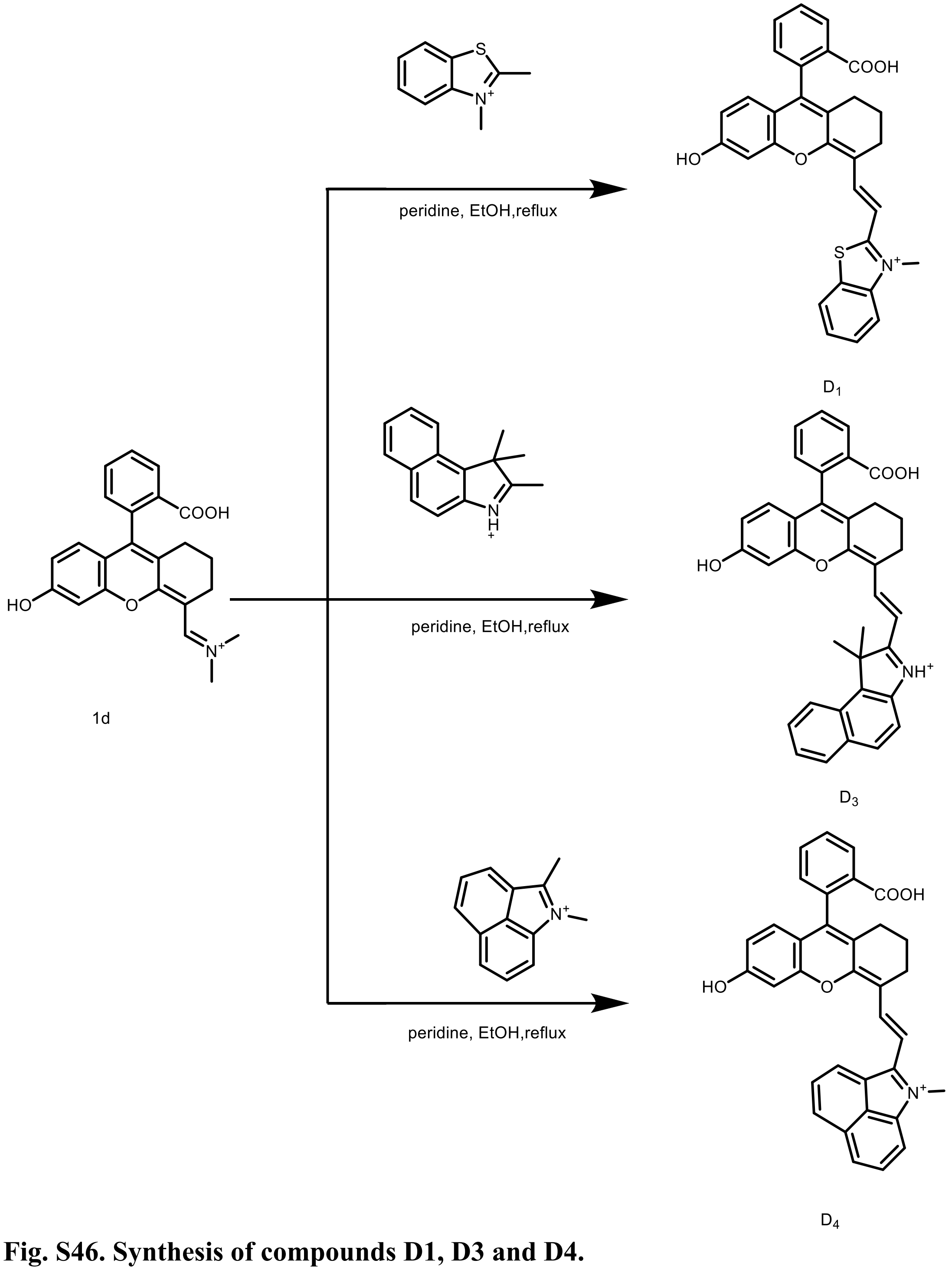

Fig. S46. Synthesis of compounds D1, D3 and D4.

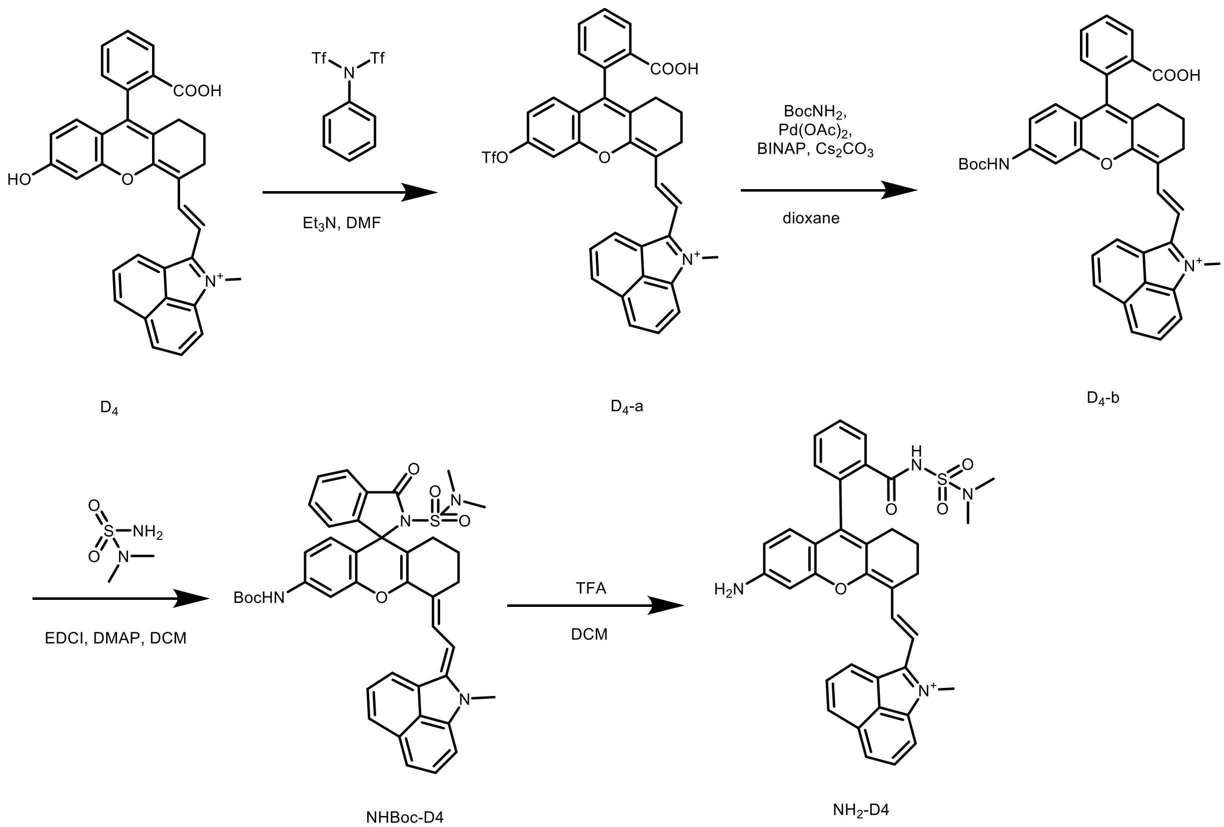

Fig. S47. Synthesis of compounds BocNH-D4 and NH_2-D4.

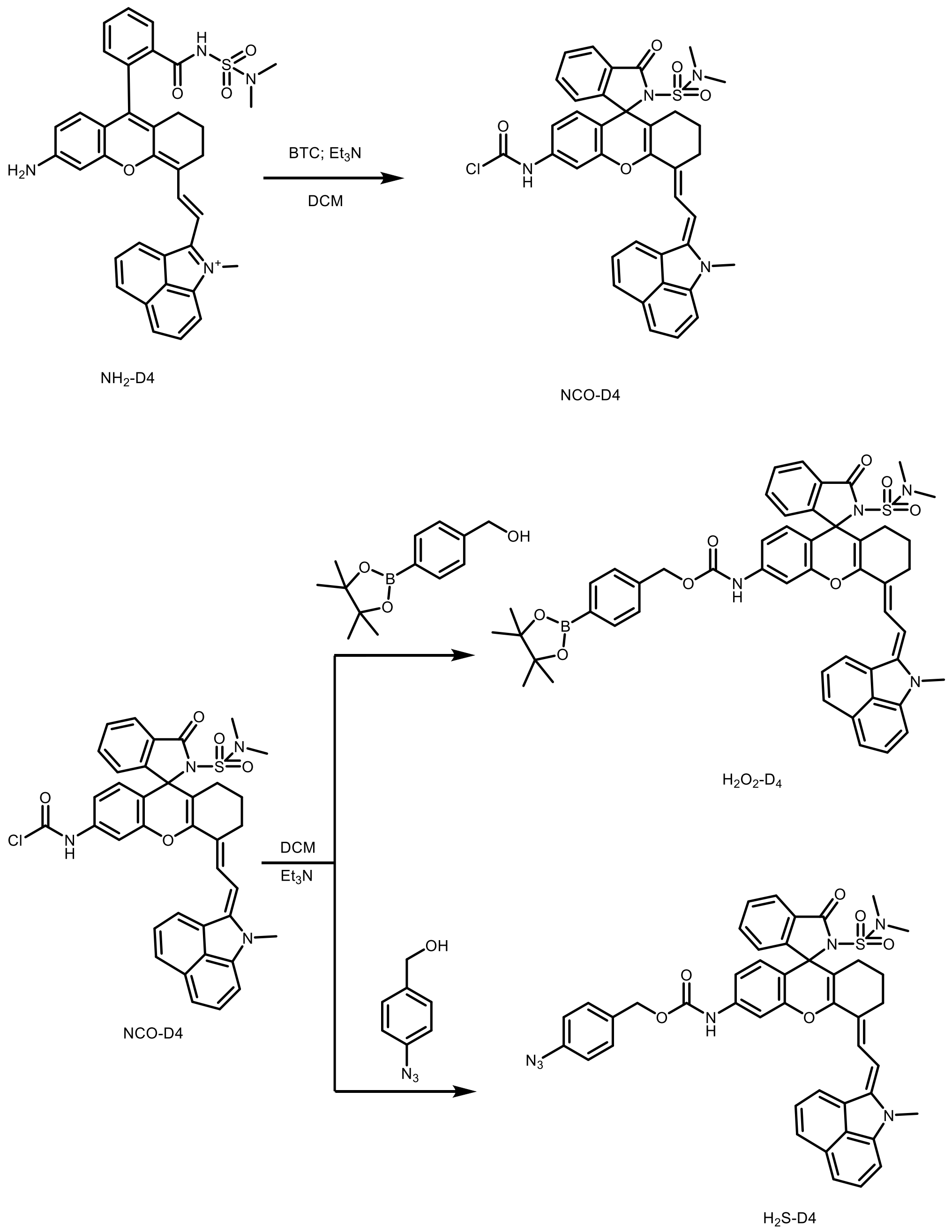

Fig. S48. Synthesis of compounds H_2O_2-D4 and H_2S-D4.

6. Synthetic procedures and structure characterization

Synthesis of compound D2

A solution of fluorescein (compound 1a) (10.0 g, 30.0 mmol) in 50 % NaOH (w/w) aqueous solution (300 mL) was heated at 165 °C for 1 h in a three-neck flask. The reaction mixture was allowed to cool to room temperature and carefully acidified with conc. HCl at 0 °C until a large amount of precipitate was formed as a pale-yellow solid (7.00 g), which is the crude product **1b**, and was used directly for the next step.

To a conc. H_2SO_4 (10 mL) under an ice bath, cyclohexanone (4 mL, 38.8 mmol) was added dropwise, and the mixture was stirred for 15 min at 0 °C, followed by the addition of dry dioxane (10 mL). The mixture was stirred for another 5 min. Compound 1b (5.00 g 19.4 mmol) was added in portions with vigorous stirring at 0 °C. The mixture was heated to 90 °C and stirred for 48 h. After cooling down to room temperature, the mixture was poured into ice water (200 mL) and stirred for 5 min. The mixture was filtrated, the solid was filtrated and washed with cold water (50 mL×3), and the filtrate was combined and extracted multiple times with DCM. The organic layer was dried over anhydrous Na_2SO_4, filtered, and then concentrated in vacuo. Crude compound **1c** was obtained as a golden-brown solid (3.00 g) and used directly for the next step.

To a round-bottom flask equipped with an ice-water bath was added DMF (3.0 mL, 38.0 mmol) and then phosphorous oxychloride (2.14 mL, 23.40 mmol) under a nitrogen atmosphere. The mixture was stirred for 30 min at 0 °C. To the above reaction mixture, a solution of compound **1c** (5.00 g, 15.6 mmol) in DMF (10.0 mL) was added at 0 °C under a nitrogen atmosphere. The resulting solution was stirred for 1 h at room temperature. The mixture was poured into ice water (200 mL) and stirred for 5 min. The mixture was filtrated and the solid was dried overnight. The crude product **1d** was obtained as a grass green solid (5.00 g) and used directly for the next step.

3-methyl-2-methylbenzo[d]thiazole-3-iumiodine (0.305 g, 1.76 mmol) and compound 1d (0.600 g, 1.59 mmol) were added to anhydrous EtOH (10 mL), then piperidine was added in a nitrogen atmosphere. The mixture was heated to reflux for 8 h. The reaction mixture was cooled to room temperature and then the solvent was extracted with DCM (10 mL×3). The extract was washed with water (10 mL×2) and brine (10 mL), The organic layer was dried over anhydrous Na_2SO_4, filtered, and then concentrated in vacuo. After purification by chromatography on a silica gel column (MeOH / DCM from 1/30 to 1/10), compound D2 was obtained as a blue solid (650 mg).
Yield: 80.9%
$\mathbf{R_f}$ = 0.13 (MeOH / DCM = 1: 10)
^{1}H NMR: (500 MHz, DMSO-d_6): δ 7.96 (d, J = 7.7 Hz, 1H), 7.76-7.72 (m, 1H), 7.64-7.61 (m, 2H), 7.36 – 7.32 (m, 1H), 7.24 (d, J = 7.6 Hz, 1H), 7.21-7.18 (m, 1H), 6.94 – 6.85 (m, 2H), 6.60 (s, 1H), 6.54 – 6.45 (m, 2H), 5.54 (d, J = 12.9 Hz, 1H), 3.25 (s, 3H), 3.16-3.14 (m, 1H), 2.59-2.57 (m, 1H), 2.46-2.43 (m, 1H), 1.97 – 1.88 (m, 2H), 1.69-1.67 (m, 1H), 1.63 (s, 6H).
ESI-MS (m/z): $[M]^+$ calc'd. for $C_{33}H_{30}NO_4^+$ 504.2169, found 504.2176

Synthesis of compound D2-OTf

N, N-phenylbistrifluoromethane-sulfonimide (0.778 g, 2.17 mmol), and triethylamine (300 μL, 2.97 mmol) were added to a solution of compound D2 (1.03 g, 1.98 mmol) in DMF (20 mL), and the mixture was stirred at room temperature for 3 h. After the addition of water, the mixture was extracted with AcOEt (20 mL×3). The extract was washed with water (20 mL×2) and brine (10 mL), The organic layer was dried over anhydrous Na_2SO_4, filtered, and then concentrated in vacuo. After purification by chromatography on a silica gel column (MeOH/ DCM from 1/30 to 1/10), compound **D2-OTf** was obtained as a purple solid (818 mg).

Yield: 65.0%

$\mathbf{R_f}$ = 0.30 (MeOH / DCM = 1: 10)

^{1}H NMR (500 MHz, Chloroform-*d*): *δ* 7.99 (d, *J* = 7.6 Hz, 1H), 7.70 – 7.65 (m, 1H), 7.60 (d, *J* = 7.6 Hz, 1H), 7.53 (s, 1H), 7.23 – 7.17 (m, 3H), 7.13 (d, *J* = 2.5 Hz, 1H), 6.91 – 6.87 (m, 2H), 6.87 – 6.83 (m, 1H), 6.80 (d, *J* = 8.8 Hz, 1H), 6.66 (d, *J* = 7.9 Hz, 1H), 3.21 (s, 3H), 2.66-2.63 (m, 1H), 2.45 (s, 1H), 2.09 – 2.00 (m, 4H), 1.70 (s, 6H).

^{13}C NMR: (126 MHz, Chloroform-*d*) δ 169.63, 151.63, 149.60, 134.94, 130.00, 129.80, 127.78, 125.32, 123.44, 121.69, 119.84, 116.21, 110.08, 106.02, 45.78, 29.71, 28.50, 24.99, 24.38, 23.04, 21.82.

ESI-MS (m/z): $[M]^+$ calc'd. for $C_{34}H_{29}F_3NO_6S^+$ 636.1663, found 636.1674

Synthesis of compound BocNH-C1

A mixture of compound **D2-OTf** (818 mg, 1.29 mmol), *tert*-butyl carbamate (256 mg, 2.19 mmol), palladium (II) acetate (30 mg, 0.089 mmol), (*S*)-(-)-2,2'-bis(diphenylphosphino)-1,1'-binaphthyl (160 mg, 0.257 mmol), Cs_2CO_3 (840 mg, 2.57 mmol) and dry dioxane (15 mL) was stirred at 90 °C for 12 h in a nitrogen atmosphere. After the addition of water to the reaction mixture, the mixture was extracted with AcOEt (20 mL×3). The extract was washed with water (20 mL×2) and brine (10 mL), The organic layer was dried over anhydrous Na_2SO_4, filtered, and then concentrated in vacuo. After purification by chromatography on a silica gel column (MeOH/ DCM from 1/30 to 1/10), compound BocNH-C1 was obtained as dark blue solid (671 mg).

Yield: 86.7%

$\mathbf{R_f}$ = 0.15 (MeOH / DCM = 1: 10)

^{1}H NMR: (500 MHz, Chloroform-*d*): *δ* 8.20 (d, *J* = 6.3 Hz, 1H), 8.05 (d, *J* = 13.4 Hz, 1H), 7.52 – 7.45 (m, 2H), 7.35 (d, *J* = 8.0 Hz, 2H), 7.30-2.28 (m, 2H), 7.13-7.09 (m, 1H), 6.99-6.97 (m, 1H), 6.93 (d, *J* = 8.0 Hz, 1H), 6.66 (d, *J* = 8.7 Hz, 1H), 5.69 (d, *J* = 13.5 Hz, 1H), 3.43 (s, 3H), 2.59 – 2.46 (m, 3H), 2.43-2.40 (m, 1H), 2.06-2.04 (m, 1H), 1.83 – 1.75 (m, 1H), 1.62(s, 3H), 1.60(s, 3H),1.49 (s, 9H).

^{13}C NMR: (126 MHz, Chloroform-*d*) δ 169.61, 153.00, 143.68, 143.36, 140.26, 131.24, 128.88, 128.29, 127.87, 122.33, 116.63, 116.11, 109.08, 105.11, 80.53, 48.44, 29.70, 28.51, 28.36, 28.02, 25.55, 24.16, 20.65.

ESI-MS (m/z): $[M]^+$ calc'd. for $C_{38}H_{39}N_2O_5^+$ 603.2854, found 603.2889

Synthesis of compound NH_2-C1

Compound **BocNH-C1** (671 mg, 1.11 mmol) mg was added in a solution of trifluoroacetic acid in DCM (15 mL), the mixture was stirred at room temperature for 1 h. After addition of water to the reaction mixture, the mixture was extracted with DCM (20 mL×3). The extract was washed with water (20 mL×2) and brine (10 mL), The organic layer was dried over anhydrous Na_2SO_4, filtered, and then concentrated in vacuo. Then Blue-green solid compound NH_2-C1 was afforded (550 mg) which was utilized in the next reaction without purification again.

Yield: 98.4%

$\mathbf{R_f}$ = 0.10 (MeOH / DCM = 1: 5)

^{1}H NMR: (500 MHz, Chloroform-*d*): δ 8.28-8.24(m, 1H), 8.19 (d, J = 13.4 Hz, 1H), 7.48 – 7.39 (m, 2H), 7.35 – 7.27 (m, 2H), 7.10-7.08 (m, 1H), 6.94 – 6.86 (m, 2H), 6.67 (d, J = 8.8 Hz, 1H), 6.55 (s, 1H), 6.31 (d, J = 8.8 Hz, 1H), 5.69 (d, J = 13.4 Hz, 1H), 3.40 (s, 3H), 2.97 – 2.86 (m, 1H), 2.65 – 2.45 (m, 3H), 2.24 – 2.15 (m, 1H), 1.85 – 1.73 (m, 1H), 1.66 (s, 3H) , 1.64 (s, 3H).

^{13}C NMR: (126 MHz, DMSO-d_6) δ 169.31, 162.47, 153.32, 152.81, 152.66, 144.82, 139.22, 133.40, 131.95, 129.64, 128.97, 128.45, 126.93, 126.78, 125.28, 122.31, 121.26, 118.01, 113.01, 108.08, 98.35, 94.29, 46.48, 30.09, 28.66, 28.60, 24.66, 24.42, 21.64.

ESI-MS (m/z): $[M]^+$ calc'd. for $C_{33}H_{31}N_2O_3^+$ 503.2330, found 503.2330

Synthesis of compound BocNH-C2

A mixture of compound **BocNH-C1** (50.0 mg, 0.830 mmol), methylamine (33.0 μL, 0.420 mmol), n-(3-dimethylaminopropyl)-n'-ethylcarbodiimide hydrochloride (64 mg, 0.33 mmol), 4-dimethylaminopyridine (41.0 mg, 0.330 mmol) and dry DCM (6 mL) was stirred at 60 °C for 12 h in a nitrogen atmosphere. After addition of water to the reaction mixture, the whole mixture was extracted with DCM (10 mL×3). The extract was washed with water (5 mL×2) and brine (5 mL), The organic layer was dried over anhydrous Na_2SO_4, filtered, and then concentrated in vacuo. After purification by chromatography on a silica gel column (PE / AcOEt = 2:1), compound **BocNH-C2** was obtained as pale-yellow solid (50 mg).

Yield: 87.3%

$\mathbf{R_f}$ **=** 0.500 (PE / AcOEt = 2:1)

^{1}H NMR (500 MHz, Chloroform-*d*): δ7.90 – 7.86 (m, 1H), 7.49 – 7.41 (m, 3H), 7.27 – 7.24 (m, 1H), 7.19 – 7.15 (m, 3H), 6.85-6.83 (m, 1H), 6.65 – 6.60 (m, 2H), 6.44 (d, J = 8.6 Hz, 1H), 5.36 (d, J = 12.6 Hz, 1H), 3.15 (s, 3H), 2.74 (s, 3H), 2.60 – 2.53 (m, 1H), 2.53 – 2.46 (m, 1H), 1.72 (s, 6H), 1.68 – 1.59 (m, 2H), 1.54 (s, 9H), 1.51-1.49 (m, 2H).

^{13}C NMR (126 MHz, Chloroform-*d*) δ 168.01, 158.03, 152.55, 152.07, 151.19, 147.74, 145.29, 139.22, 138.86, 132.12, 131.75, 128.34, 128.04, 127.70, 123.25, 122.92, 121.58, 119.84, 119.69, 119.36, 114.14, 112.70, 105.94, 105.72, 103.43, 91.85, 81.11, 66.02, 45.52, 29.14, 28.50, 28.35, 25.40, 24.89, 22.80, 22.15.

ESI-MS (m/z): $[M+H]^+$ calc'd. for $C_{39}H_{41}N_3O_4$ 616.3170, found 616.3149

Synthesis of compound NH_2-C2

Compound **BocNH-C2** (50.0 mg, 0.081 mmol) was added in a solution of trifluoroacetic acid in DCM (5 mL), the mixture was stirred at room temperature for 1 h. After addition of water to the reaction mixture, the whole mixture was extracted with DCM (10 mL×3). The extract was washed with water (10 mL×2) and brine (5 mL), The organic layer was dried over anhydrous Na_2SO_4, filtered, and then concentrated in vacuo. Then compound **NH_2-C2** was afforded (40.0 mg) as white solid.

Yield: 95.7%

R_f = 0.120 (PE / AcOEt = 2:1)

^{1}H NMR (500 MHz, Chloroform-*d*): *δ* 7.86 (d, *J* = 7.2 Hz, 1H), 7.52 – 7.39 (m, 3H), 7.18-7.15 (m, 3H), 6.86-6.83 (m, 1H), 6.61 (d, *J* = 7.9 Hz, 1H), 6.45 (d, *J* = 2.3 Hz, 1H), 6.34 – 6.19 (m, 2H), 5.37 (d, *J* = 12.6 Hz, 1H), 3.15 (s, 3H), 2.73 (s, 3H), 2.60 – 2.41 (m, 2H), 1.72 (s, 6H), 1.70 – 1.55 (m, 3H), 1.47 – 1.40 (m, 1H).

^{13}C NMR (126 MHz, Chloroform-*d*) δ 167.96, 157.83, 147.58, 147.33, 145.33, 138.81, 132.01, 131.91, 128.42, 128.15, 127.72, 123.23, 122.80, 121.55, 120.03, 119.48, 119.33, 111.53, 108.15, 105.70, 103.60, 101.43, 91.91, 66.21, 45.50, 29.14, 28.46, 25.36, 24.81, 22.80, 22.21.

ESI-MS (m/z): $[M+H]^+$ calc'd. for $C_{34}H_{33}N_3O_2$ 516.2646, found 516.2639

Synthesis of compound BocNH-C3

A mixture of compound **BocNH-C1** (75.0 mg, 0.124 mmol), methanesulfonamide (60.0 mg, 0.622 mmol), n-(3-dimethylaminopropyl)-n'-ethylcarbodiimide hydrochloride (95.4 mg, 0.500 mmol), 4-dimethylaminopyridine (60.8 mg, 0.500 mmol) and dry DCM (6 mL) was stirred at 60 °C for 12 h in a nitrogen atmosphere. After addition of water to the reaction mixture, the whole mixture was extracted with DCM (10 mL×3). The extract was washed with water (5 mL×2) and brine (5 mL), The organic layer was dried over anhydrous Na_2SO_4, filtered, and then concentrated in vacuo. After purification by chromatography on a silica gel column (MeOH / DCM from 1/30 to 1/10), compound **BocNH-C3** was obtained as blue solid (52.0 mg).

Yield: 61.9%

R_f = 0.500 (MeOH / DCM = 1: 10)

^{1}H NMR (500 MHz, Methanol-*d*$_4$) δ 8.38 – 8.35 (m, 1H), 7.94 – 7.89 (m, 2H), 7.89 – 7.84 (m, 2H), 7.71 (s, 1H), 7.67 – 7.59 (m, 2H), 7.47 – 7.43 (m, 1H), 7.40 – 7.31 (m, 2H), 7.29 – 7.21 (m, 1H), 7.07 – 7.00 (m, 1H), 3.70 – 3.64 (m, 2H), 3.31 (s, 3H), 3.01 – 2.78 (m, 2H), 2.72 – 2.42 (m, 2H), 2.07 (s, 6H), 1.87 (s, 9H).

ESI-MS (m/z): $[M]^+$ calc'd. for $C_{39}H_{42}N_3O_6S^+$ 680.2789, found 680.2794

Synthesis of compound NH_2-C3

Compound **BocNH-C3** (52.0 mg, 0.076 mmol) was added in a solution of trifluoroacetic acid in DCM (5 mL), the mixture was stirred at room temperature for 1 h. After addition of water to the reaction mixture, the whole mixture was extracted with DCM (10 mL×3). The extract was washed with water (10 mL×2) and brine (5 mL), The organic layer was dried over anhydrous Na_2SO_4, filtered, and then concentrated in vacuo. Then compound **NH_2-C3** was afforded (39.0 mg) as blue solid.

Yield: 87.9%

R_f = 0.500 (MeOH / DCM = 1: 10)

^{1}H NMR (500 MHz, DMSO-d_6): δ 7.92 (d, J = 7.6 Hz, 1H), 7.72 – 7.61 (m, 2H), 7.58-7.77 (m, 1H), 7.36 (d, J = 7.3 Hz, 1H), 7.21 (t, J = 7.8 Hz, 1H), 7.15 (d, J = 7.7 Hz, 1H), 6.96 – 6.89 (m, 2H), 6.47 – 6.38 (m, 2H), 6.36-6.31 (m, 1H), 5.57 (d, J = 13.0 Hz, 1H), 3.28 (s, 3H), 3.17-3.14 (m, 1H), 2.96 (s, 3H), 2.59-2.53 (m, 1H), 2.05 – 1.94 (m, 1H), 1.65 (s, 6H), 1.61 (m, 2H), 1.26-1.20 (m, 1H).
ESI-MS (m/z): $[M]^+$ calc'd. for $C_{34}H_{34}N_3O_4S^+$ 580.2265, found 580.2274

Synthesis of compound BocNH-C4

A mixture of compound **BocNH-C1** (50.0 mg, 0.830 mmol), trifluoromethanesulphonamide (52.0 mg, 0.420 mmol), n-(3-dimethylaminopropyl)-n'-ethylcarbodiimide hydrochloride (64.0 mg, 0.330 mmol), 4-dimethylaminopyridine (41.0 mg, 0.330 mmol) and dry DCM (6 mL) was stirred at 60 ºC for 12 h in a nitrogen atmosphere. After addition of water to the reaction mixture, the whole mixture was extracted with DCM (10 mL×3). The extract was washed with water (5 mL×2) and brine (5 mL), The organic layer was dried over anhydrous Na_2SO_4, filtered, and then concentrated in vacuo. After purification by chromatography on a silica gel column (MeOH / DCM from 1/20 to 1/10), compound **BocNH-C4** was obtained as blue solid (30.0 mg).
Yield: 49.3%
R_f = 0.500 (MeOH / DCM = 1:20)
^{1}H NMR (500 MHz, DMSO-d_6) δ 8.63 (d, J = 14.6 Hz, 1H), 8.09 – 8.02 (m, 1H), 7.82 – 7.74 (m, 2H), 7.63 – 7.55 (m, 3H), 7.52 – 7.47 (m, 1H), 7.43 – 7.36 (m, 1H), 7.23 (d, J = 8.8 Hz, 1H), 7.17 – 7.11 (m, 1H), 6.68 (d, J = 8.7 Hz, 1H), 6.45 (d, J = 14.8 Hz, 1H), 2.69 – 2.61 (m, 2H), 2.30 – 2.23 (m, 2H), 1.75 – 1.69 (m, 2H), 1.51 (s, 6H), 1.47 (s, 3H), 1.23 (s, 9H).
^{13}C NMR (126 MHz, DMSO-d_6) δ 176.60, 169.90, 161.87, 153.36, 152.88, 149.92, 144.39, 143.57, 142.94, 138.20, 134.17, 131.11, 130.50, 129.15, 127.54, 126.72, 123.83, 123.08, 118.14, 116.18, 114.61, 112.92, 103.85, 103.44, 80.62, 70.24, 50.19, 32.55, 29.49, 28.50, 27.95, 27.12, 23.99, 20.07.
ESI-MS (m/z): $[M]^+$ calc'd. for $C_{39}H_{39}F_3N_3O_6S^+$ 734.2507, found 734.2516

Synthesis of compound NH$_2$-C4

Compound **BocNH-C4** (30.0 mg, 0.041 mmol) was added in a solution of trifluoroacetic acid in DCM (5 mL), the mixture was stirred at room temperature for 1 h. After addition of water to the reaction mixture, the whole mixture was extracted with DCM (10 mL×3). The extract was washed with water (10 mL×2) and brine (5 mL), The organic layer was dried over anhydrous Na_2SO_4, filtered, and then concentrated in vacuo. Then compound **NH$_2$-C4** was afforded (21.0 mg) as blue solid.
Yield: 81.1%
R_f = 0.300 (MeOH / DCM = 1:10)
^{1}H NMR (500 MHz, DMSO-d_6) δ 8.46 (d, J = 14.0 Hz, 1H), 8.08 – 8.03 (m, 1H), 7.64 – 7.59 (m, 2H), 7.58 – 7.53 (m, 1H), 7.43 – 7.35 (m, 2H), 7.26 – 7.21 (m, 1H), 7.14 – 7.11 (m, 1H), 6.92 (s, 1H), 6.74 – 6.71 (m, 1H), 6.64 – 6.62 (m, 1H), 6.12 (d, J = 14.0 Hz, 1H), 3.62 (s, 3H), 3.17-3.15(m, 2H), 2.65 – 2.59 (m, 2H), 2.26 – 2.21 (m, 2H), 1.72 (s, 6H).
ESI-MS (*m/z*): $[M]^+$ calc'd. for $C_{34}H_{31}F_3N_3O_4S^+$ 634.1982, found 634.2002

Synthesis of compound BocNH-C5

A mixture of compound **BocNH-C1** (50.0 mg, 0.830 mmol), N, N-dimethylsulfamide (62.0 mg, 0.420 mmol), n-(3-dimethylaminopropyl)-n'-ethylcarbodiimide hydrochloride (64.0 mg, 0.330 mmol), 4-dimethylaminopyridine (41.0 mg, 0.330 mmol) and dry DCM (6 mL) was stirred at 60 °C for 12 h in a nitrogen atmosphere. After addition of water to the reaction mixture, the whole mixture was extracted with DCM (10 mL×3). The extract was washed with water (5 mL×2) and brine (5 mL), The organic layer was dried over anhydrous Na_2SO_4, filtered, and then concentrated in vacuo. After purification by chromatography on a silica gel column (PE / AcOEt from 5/1 to 2/1), compound **BocNH-C5** was obtained as pale-yellow solid (43.0 mg).

Yield: 72.8%

R_f = 0.500 (PE / AcOEt = 1:1)

^{1}H NMR (500 MHz, Chloroform-*d*) δ 7.89 (d, *J* = 7.6 Hz, 1H), 7.62 – 7.55 (m, 1H), 7.52 – 7.46 (m, 2H), 7.18 – 7.12 (m, 3H), 7.07 (s, 1H), 6.87 – 6.81 (m, 1H), 6.63 – 6.56 (m, 2H), 6.55 (d, *J* = 8.7 Hz, 1H), 5.36 (d, *J* = 12.6 Hz, 1H), 3.14 (s, 3H), 2.82 (s, 6H), 2.72 – 2.62 (m, 1H), 2.45 – 2.37 (m, 1H), 2.07 – 2.00 (m, 1H), 1.79 – 1.70 (m, 3H), 1.71 (s, 3H), 1.69 (s, 3H), 1.53 (s, 9H).

^{13}C NMR (126 MHz, Chloroform-*d*) δ 166.37, 156.77, 151.45, 151.36, 150.94, 146.94, 144.30, 138.36, 137.88, 133.46, 127.91, 127.65, 127.26, 126.61, 123.15, 122.75, 120.50, 119.09, 119.02, 118.21, 112.98, 112.07, 104.63, 101.60, 91.14, 80.02, 69.86, 44.44, 36.93, 28.09, 27.51, 27.40, 27.30, 24.29, 22.46, 20.94.

ESI-MS (*m/z*): $[M+H]^+$ calc'd. for $C_{40}H_{44}N_4O_6S$ 709.3055, found 709.3083

Synthesis of compound NH_2-C5

Compound **BocNH-C5** (43.0 mg, 0.061 mmol) was added in a solution of trifluoroacetic acid in DCM (5 mL), the mixture was stirred at room temperature for 1 h. After addition of water to the reaction mixture, the whole mixture was extracted with DCM (10 mL×3). The extract was washed with water (10 mL×2) and brine (5 mL), The organic layer was dried over anhydrous Na_2SO_4, filtered, and then concentrated in vacuo. Then compound **NH_2-C5** was afforded (35.0 mg) as green solid.

Yield: 94.9%

R_f = 0.200 (PE / AcOEt = 1:1)

^{1}H NMR (500 MHz, Chloroform-*d*) δ 7.87 (d, *J* = 7.7 Hz, 1H), 7.62 – 7.55 (m, 1H), 7.52 – 7.45 (m, 2H), 7.21 – 7.10 (m, 3H), 6.84 (t, *J* = 7.4 Hz, 1H), 6.61 (d, *J* = 7.8 Hz, 1H), 6.48 (d, *J* = 2.3 Hz, 1H), 6.41 (d, *J* = 8.4 Hz, 1H), 6.26-6.24 (m, 1H), 5.37 (d, *J* = 12.7 Hz, 1H), 3.14 (s, 3H), 2.83 (s, 6H), 2.71 – 2.62 (m, 1H), 2.45 – 2.35 (m, 1H), 2.06 – 1.97 (m, 1H), 1.70 (s, 6H), 1.55 – 1.45 (m, 3H).

^{13}C NMR (126 MHz, Chloroform-*d*) δ 166.40, 152.01, 137.92, 133.30, 127.87, 127.72, 127.57, 126.62, 123.20, 122.69, 120.50, 118.21, 109.58, 104.63, 100.55, 44.47, 36.92, 28.68, 28.11, 27.51, 24.28, 22.54, 21.01.

ESI-MS (*m/z*): $[M]^+$ calc'd. for $C_{35}H_{37}N_4O_4S^+$ 609.2531, found 609.2540

Synthesis of compound BocNH-C6

A mixture of compound **BocNH-C1** (50.0 mg, 0.830 mmol), 4-bromobenzenesulfonamide (98.0 mg, 0.420 mmol), n-(3-dimethylaminopropyl)-n'-ethylcarbodiimide hydrochloride (64.0 mg, 0.330 mmol), 4-dimethylaminopyridine (41.0 mg, 0.330 mmol) and dry DCM (6 mL) was stirred at 60 °C for 12 h in a nitrogen atmosphere. After addition of water to the reaction mixture, the whole mixture was extracted with DCM (10 mL×3). The extract was washed with water (5 mL×2) and brine (5 mL), The organic layer was dried over anhydrous Na_2SO_4, filtered, and then concentrated in vacuo. After purification by chromatography on a silica gel column (MeOH / DCM from 1/30 to 1/10), compound **BocNH-C6** was obtained as blue solid (60.0 mg).

Yield: 88.2%

R_f = 0.500 (MeOH / DCM = 1: 30)

^{1}H NMR (500 MHz, Chloroform-*d*): δ 7.85 (d, J = 7.7 Hz, 1H), 7.60 – 7.55 (m, 4H), 7.52 – 7.50 (m, 2H), 7.48 – 7.44 (m, 1H), 7.21 – 7.16 (m, 2H), 7.13 (d, J = 7.8 Hz, 1H), 6.87-6.85 (m, 2H), 6.68 – 6.60 (m, 2H), 6.26 (d, J = 8.6 Hz, 1H), 5.40 (d, J = 12.6 Hz, 1H), 3.17 (s, 3H), 2.67 – 2.60 (m, 1H), 2.52 – 2.45 (m, 1H), 2.05-2.00 (m, 1H), 1.75 (s, 3H), 1.74 (s, 3H), 1.63 (m, 3H),1.56 (s, 9H).

^{13}C NMR (126 MHz, Chloroform-*d*) δ 165.47, 151.27, 138.67, 133.86, 130.56, 129.13, 128.07, 127.37, 126.66, 122.91, 120.57, 112.44, 112.08, 104.90, 91.16, 80.07, 44.59, 28.68, 28.14, 27.57, 27.33, 24.23, 22.52, 20.90.

ESI-MS (*m/z*): $[M]^+$ calc'd. for $C_{44}H_{43}BrN_3O_6S^+$ 820.2051 and 822.2030, found 820.2053 and 822.2046

Synthesis of compound NH_2-C6

Compound **BocNH-C6** (60.0 mg, 0.073 mmol) was added in a solution of trifluoroacetic acid in DCM (5 mL), the mixture was stirred at room temperature for 1 h. After addition of water to the reaction mixture, the whole mixture was extracted with DCM (10 mL×3). The extract was washed with water (10 mL×2) and brine (5 mL), The organic layer was dried over anhydrous Na_2SO_4, filtered, and then concentrated in vacuo. Then compound **NH_2-C6** was afforded (52.0 mg) as purple solid.

Yield: 98.7%

R_f = 0.420 (MeOH / DCM = 1: 30)

^{1}H NMR (500 MHz, DMSO-d_6): δ 8.29 (d, J = 13.7 Hz, 1H), 7.98 – 7.90 (m, 1H), 7.56 – 7.50 (m, 1H), 7.43 (d, J = 8.2 Hz, 2H), 7.40 – 7.34 (m, 3H), 7.30 (d, J = 7.9 Hz, 1H), 7.19-7.17 (m, 1H), 7.05 (d, J = 7.1 Hz, 1H), 6.79 (s, 2H), 6.68 – 6.63 (m, 1H), 6.59 – 6.51 (m, 2H), 6.02 (d, J = 13.9 Hz, 1H), 3.56 (s, 3H), 2.06-2.03 (m, 2H), 1.75(s, 3H), 1.72 (s, 3H), 1.62 – 1.52 (m, 2H), 1.26-1.20 (m, 2H).

^{13}C NMR (126 MHz, DMSO-d_6) δ 170.09, 169.51, 160.74, 155.54, 155.04, 144.50, 140.47, 135.90, 130.97, 130.60, 129.31, 128.14, 124.27, 123.96, 122.67, 118.47, 115.83, 114.97, 113.24, 110.49, 98.13, 97.47, 48.34, 31.18, 28.65, 28.58, 26.24, 24.22, 20.59.

ESI-MS (*m/z*): $[M]^+$ calc'd. for $C_{39}H_{35}BrN_3O_4S^+$ 720.1527 and 722.1506, found 720.1535 and 722.1527

Synthesis of compound BocNH-C7

A mixture of compound **BocNH-C1** (50.0 mg, 0.830 mmol), aniline (37.0 uL, 0.420 mmol), n-(3-dimethylaminopropyl)-n'-ethylcarbodiimide hydrochloride (64.0 mg, 0.330 mmol), 4-dimethylaminopyridine (41.0 mg, 0.330 mmol) and dry DCM (6 mL) was stirred at 60 °C for 12 h in a nitrogen atmosphere. After addition of water to the reaction mixture, the whole mixture was extracted with DCM (10 mL×3). The extract was washed with water (5 mL×2) and brine (5 mL), The organic layer was dried over anhydrous Na_2SO_4, filtered, and then concentrated in vacuo. After purification by chromatography on a silica gel column (MeOH / DCM from 1/30 to 1/10), compound **BocNH-C7** was obtained as white solid (50.0 mg).

Yield: 89.3%

R_f = 0.500 (MeOH / DCM = 1:10)

^{1}H NMR ^{1}H NMR (500 MHz, Chloroform-*d*) δ 8.00 – 7.95 (m, 1H), 7.56 – 7.47 (m, 2H), 7.36 (d, *J* = 12.6 Hz, 1H), 7.24 – 7.19 (m, 3H), 7.18 – 7.16 (m, 2H), 7.16 – 7.11 (m, 4H), 6.88 – 6.81 (m, 1H), 6.65 (d, *J* = 8.6 Hz, 1H), 6.63 – 6.57 (m, 2H), 5.32 (d, *J* = 12.6 Hz, 1H), 3.13 (s, 3H), 2.60 – 2.49 (m, 1H), 2.47 – 2.35 (m, 1H), 1.98 – 1.89 (m, 1H), 1.69 (s, 3H), 1.67 (s, 3H), 1.65 – 1.56 (m, 3H), 1.53 (s, 9H).

^{13}C NMR (126 MHz, Chloroform-*d*): *δ* 167.66, 157.00, 151.53, 150.78, 150.26, 146.59, 144.19, 138.25, 137.79, 137.06, 135.62, 131.96, 130.04, 127.86, 127.56, 126.64, 125.65, 125.13, 123.10, 122.50, 118.89, 118.33, 112.93, 104.7, 102.74, 90.72, 79.90, 68.11, 44.45, 28.06, 27.39, 27.28, 24.22, 23.43, 22.29, 20.91.

ESI-MS (*m/z*): $[M+H]^+$ calc'd. for $C_{44}H_{43}N_3O_4$ 678.3327, found 678.3331

Synthesis of compound NH_2-C7

Compound **BocNH-C7** (50.0 mg, 0.074 mmol) was added in a solution of trifluoroacetic acid in DCM (5 mL), the mixture was stirred at room temperature for 1h. After addition of water to the reaction mixture, the whole mixture was extracted with DCM (10 mL×3). The extract was washed with water (10 mL×2) and brine (5 mL), The organic layer was dried over anhydrous Na_2SO_4, filtered, and then concentrated in vacuo. Then compound **NH_2-C7** was afforded (42.0 mg) as white solid.

Yield: 98.4%

R_f = 0.350 (MeOH / DCM = 1:10)

^{1}H NMR (500 MHz, Chloroform-*d*) δ 7.99 – 7.93 (m, 1H), 7.56 – 7.51 (m, 1H), 7.51 – 7.44 (m, 1H), 7.35 (d, *J* = 12.7 Hz, 1H), 7.26 – 7.18 (m, 4H), 7.17 – 7.12 (m, 4H), 6.85-6.83 (m, 1H), 6.60 (d, *J* = 7.8 Hz, 1H), 6.53 (d, *J* = 8.4 Hz, 1H), 6.36 (d, *J* = 2.3 Hz, 1H), 6.28-6.26 (m, 1H), 5.32 (d, *J* = 12.6 Hz, 1H), 3.13 (s, 3H), 2.59 – 2.48 (m, 1H), 2.48 – 2.33 (m, 1H), 2.00 – 1.86 (m, 1H), 1.69 (s, 3H), 1.67 (s, 3H), 1.66 – 1.57 (m, 3H).

^{13}C NMR (126 MHz, Chloroform-*d*) δ 168.07, 157.77, 152.25, 151.48, 147.24, 145.29, 137.02, 132.75, 131.30, 128.86, 128.66, 128.32, 127.69, 126.35, 126.03, 123.47, 123.44, 121.53, 119.36, 119.31, 111.54, 109.57, 105.69, 104.27, 101.53, 91.84, 69.17, 45.48, 29.11, 28.43, 28.38, 25.24, 23.30, 22.01.

ESI-MS (*m/z*): $[M+H]^+$ calc'd. for $C_{39}H_{35}N_3O_2$ 578.2803, found 578.2904

Synthesis of compound BocNH-C8

A mixture of compound **BocNH-C1** (50.0 mg, 0.830 mmol), 4-aminobenzotrifluoride (52.0 µL, 0.420 mmol), n-(3-dimethylaminopropyl)-n'-ethylcarbodiimide hydrochloride (64.0 mg, 0.330 mmol), 4-dimethylaminopyridine (41.0 mg, 0.330 mmol) and dry DCM (6 mL) was stirred at 60 °C for 12 h in a nitrogen atmosphere. After addition of water to the reaction mixture, the whole mixture was extracted with DCM (10 mL×3). The extract was washed with water (5 mL×2) and brine (5 mL), The organic layer was dried over anhydrous Na_2SO_4, filtered, and then concentrated in vacuo. After purification by chromatography on a silica gel column (PE / AcOEt = 8:1), compound **BocNH-C8** was obtained as white solid (55.0 mg).

Yield: 88.7%

R_f = 0.600 (PE / AcOEt = 8:1)

^{1}H NMR ^{1}H NMR (500 MHz, Chloroform-*d*) δ 7.99 – 7.95 (m, 1H), 7.56 – 7.45 (m, 2H), 7.36 – 7.32 (m, 1H), 7.24 – 7.17 (m, 1H), 7.17 – 7.12 (m, 3H), 6.96 – 6.89 (m, 2H), 6.87 – 6.80 (m, 1H), 6.76 – 6.71 (m, 2H), 6.66 – 6.54 (m, 3H), 5.32 (d, *J* = 12.6 Hz, 1H), 3.72 (s, 3H), 3.13 (s, 3H), 2.61 – 2.53 (m, 1H), 2.45 – 2.34 (m, 1H), 1.99 – 1.87 (m, 1H), 1.71(s, 3H), 1.64 (s, 3H), 1.65 – 1.56 (m, 3H), 1.53 (s, 9H).

^{13}C NMR (126 MHz, Chloroform-*d*) δ 168.06, 58.05, 157.96, 152.49, 151.95, 145.26, 139.17, 138.86, 132.70, 131.48, 129.27, 128.59, 128.48, 127.84, 127.67, 123.55, 123.50, 121.56, 119.89, 119.57, 119.35, 114.12, 113.79, 105.74, 105.71, 103.84, 91.81, 80.74, 68.93, 55.24, 45.49, 29.11, 28.50, 28.41, 28.35, 25.31, 23.39, 21.99.

ESI-MS (*m/z*): $[M+H]^+$ calc'd. for $C_{45}H_{42}F_3N_3O_4$ 746.3201, found 746.3201

Synthesis of compound NH_2-C8

Compound **BocNH-C8** (55.0 mg, 0.074 mmol) was added in a solution of trifluoroacetic acid in DCM (5 mL), the mixture was stirred at room temperature for 1 h. After addition of water to the reaction mixture, the whole mixture was extracted with DCM (10 mL×3). The extract was washed with water (10 mL×2) and brine (5 mL), The organic layer was dried over anhydrous Na_2SO_4, filtered, and then concentrated in vacuo. Then compound **NH_2-C8** was afforded (45.0 mg) as white solid.

Yield: 94.5%

R_f = 0.300 (PE / AcOEt = 8:1)

^{1}H NMR ^{1}H NMR (500 MHz, Chloroform-*d*) δ 7.99 – 7.93 (m, 1H), 7.56 – 7.44 (m, 2H), 7.32 (d, *J* = 12.8 Hz, 1H), 7.25 – 7.19 (m, 1H), 7.19 – 7.12 (m, 2H), 6.97 – 6.91 (m, 2H), 6.84 (d, *J* = 1.0 Hz, 1H), 6.77 – 6.72 (m, 2H), 6.60 (d, *J* = 7.8 Hz, 1H), 6.51 (d, *J* = 8.4 Hz, 1H), 6.35 (d, *J* = 2.3 Hz, 1H), 6.31 – 6.27 (m, 1H), 5.32 (d, *J* = 12.6 Hz, 1H), 3.72 (s, 3H), 3.13 (s, 3H), 2.97 – 2.86 (m, 1H), 2.42 – 2.34 (m, 1H), 1.95 – 1.88 (m, 1H), 1.68(s, 3H), 1.66(s, 3H) 1.64 – 1.57 (m, 3H).

^{13}C NMR (126 MHz, Chloroform-*d*) δ 168.07, 158.01, 157.75, 151.30, 147.24, 145.29, 138.80, 132.59, 131.62, 128.98, 128.30, 127.98, 127.70, 123.50, 123.45, 121.54, 120.08, 119.31, 114.05, 111.48, 109.40, 105.69, 103.94, 101.49, 91.83, 69.19, 55.25, 45.47, 29.11, 28.45, 28.37, 25.27, 23.39, 22.03.

ESI-MS (*m/z*): $[M+H]^+$ calc'd. for $C_{40}H_{34}F_3N_3O_2$ 646.2676, found 646.2744

Synthesis of compound BocNH-C9

A mixture of compound **BocNH-C1** (50.0 mg, 0.830 mmol), p-anisidine (51.0 mg, 0.420 mmol), n-(3-dimethylaminopropyl)-n'-ethylcarbodiimide hydrochloride (64.0 mg, 0.330 mmol), 4-dimethylaminopyridine (41.0 mg, 0.330 mmol) and dry DCM (6 mL) was stirred at 60 °C for 12 h in a nitrogen atmosphere. After addition of water to the reaction mixture, the whole mixture was extracted with DCM (10 mL×3). The extract was washed with water (5 mL×2) and brine (5 mL), The organic layer was dried over anhydrous Na_2SO_4, filtered, and then concentrated in vacuo. After purification by chromatography on a silica gel column (MeOH / DCM = 1:10), compound **BocNH-C9** was obtained as white solid (44.0 mg).

Yield: 74.9%

$\mathbf{R_f}$ **=** 0.350 (MeOH / DCM = 1:10)

^{1}H NMR (500 MHz, Chloroform-*d*) δ 7.97 (d, *J* = 7.5 Hz, 1H), 7.59 – 7.51 (m, 1H), 7.52 – 7.39 (m, 6H), 7.24 – 7.21 (m, 1H), 7.20 – 7.15 (m, 2H), 6.91 – 6.82 (m, 2H), 6.67 – 6.59 (m, 2H), 6.57 (s, 1H), 5.34 (d, *J* = 12.6 Hz, 1H), 3.14 (s, 3H), 2.56 – 2.47 (m, 1H), 2.49 – 2.40 (m, 1H), 1.88 – 1.82 (m, 1H), 1.72 (s, 3H), 1.70 (s, 3H), 1.64 – 1.56 (m, 3H), 1.53 (s, 9H).

^{13}C NMR (126 MHz, Chloroform-*d*) δ 168.41, 158.32, 152.43, 151.51, 151.33, 147.44, 145.22, 139.45, 138.85, 133.41, 130.23, 128.72, 128.03, 127.71, 125.86, 125.83, 124.30, 123.65, 123.40, 121.59, 120.11, 119.46, 119.43, 114.16, 113.67, 105.94, 105.79, 104.03, 91.73, 81.07, 68.89, 45.57, 29.13, 28.5, 28.32, 25.20, 23.10, 21.94.

ESI-MS (*m/z*): $[M+H]^+$ calc'd. for $C_{45}H_{45}N_3O_5$ 708.3432, found 708.3420

Synthesis of compound NH_2-C9

Compound **BocNH-C9** (44.0 mg, 0.062 mmol) was added in a solution of trifluoroacetic acid in DCM (5 mL), the mixture was stirred at room temperature for 1 h. After addition of water to the reaction mixture, the whole mixture was extracted with DCM (10 mL×3). The extract was washed with water (10 mL×2) and brine (5 mL), The organic layer was dried over anhydrous Na_2SO_4, filtered, and then concentrated in vacuo. Then compound **NH_2-C9** was afforded (32.0 mg) as white solid.

Yield: 86.5%

$\mathbf{R_f}$ **=** 0.120 (MeOH / DCM = 1:10)

^{1}H NMR (500 MHz, Chloroform-*d*) δ 8.00 – 7.94 (m, 1H), 7.57 – 7.52 (m, 1H), 7.51 – 7.39 (m, 6H), 7.24 – 7.21 (m, 1H), 7.20 – 7.15 (m, 2H), 6.88 – 6.82 (m, 1H), 6.62 (d, *J* = 7.8 Hz, 1H), 6.50 (d, *J* = 8.5 Hz, 1H), 6.41 (d, *J* = 2.3 Hz, 1H), 6.27-6.25 (m, 1H), 5.33 (d, *J* = 12.7 Hz, 1H), 3.14 (s, 3H), 2.55 – 2.47 (m, 1H), 2.47 – 2.39 (m, 1H), 1.88 – 1.80 (m, 1H), 1.72 (s, 3H), 1.70 (s, 3H), 1.63 – 1.54 (m, 3H).

^{13}C NMR (126 MHz, Chloroform-*d*): *δ* 168.32, 158.11, 151.98, 151.55, 147.50, 147.35, 145.25, 140.73, 138.80, 133.29, 130.41, 128.52, 128.43, 127.73, 125.77, 125.74, 125.16, 124.44, 123.54, 123.40, 123.00, 121.55, 119.87, 119.64, 119.42, 111.75, 109.23, 105.77, 104.09, 101.61, 91.77, 69.21, 45.54, 29.12, 28.46, 28.43, 26.93, 25.16, 23.11, 21.99.

ESI-MS (*m/z*): $[M+H]^+$ calc'd. for $C_{40}H_{37}N_3O_3$ 608.2908, found 608.2929

Synthesis of compound D1

2,3-dimethylbenzothiazolium iodide (0.096 g, 0.580 mmol) and compound **1d** (0.200 g, 0.540 mmol) were added to anhydrous EtOH (10 mL), then piperdine was added in a nitrogen atmosphere. The mixture was heated to reflux for 8 h. The mixture was cooled to room temperature. After addition of water to the reaction mixture, the whole mixture was extracted with DCM (15 mL×3). The extract was washed with water (10 mL×2) and brine (5 mL), The organic layer was dried over anhydrous Na_2SO_4, filtered, and then concentrated in vacuo. After purification by chromatography on a silica gel column (MeOH / DCM = 1:30), compound **D1** was obtained as a blue solid (200 mg).

Yield: 76.3%

$\mathbf{R_f}$ **=** 0.150 (MeOH / DCM = 1:30)

^{1}H NMR (500 MHz, DMSO-d_6) δ 8.05 – 8.00 (m, 1H), 7.71 (d, J = 7.8 Hz, 1H), 7.49 – 7.43 (m, 2H), 7.36 (d, J = 3.1 Hz, 2H), 7.17 – 7.11 (m, 1H), 7.03 – 6.98 (m, 1H), 6.53 (d, J = 9.3 Hz, 1H), 6.25 (d, J = 9.3 Hz, 1H), 6.20 (s, 1H), 5.90 (d, J = 12.9 Hz, 1H), 5.55-5.53 (m, 1H), 3.15 (s, 3H), 2.56 – 2.53 (m, 2H), 2.29 – 2.20 (m, 1H), 2.11 – 2.01 (m, 1H), 1.70 – 1.59 (m, 2H).

ESI-MS (*m/z*): $[M]^+$ calc'd. for $C_{30}H_{24}NO_4S^+$ 494.1421, found 494.1421

Synthesis of compound D3

1,1,2-trimethyl-1H-benzeindole (0.047 g, 0.580 mmol) and compound **1d** (0.200 g, 0.530 mmol) were added to anhydrous EtOH (10 mL), then piperdine was added in a nitrogen atmosphere. The mixture was heated to reflux for 8 h. The mixture was cooled to room temperature. After addition of water to the reaction mixture, the whole mixture was extracted with DCM (15 mL×3). The extract was washed with water (10 mL×2) and brine (5 mL), The organic layer was dried over anhydrous Na_2SO_4, filtered, and then concentrated in vacuo. After purification by chromatography on a silica gel column (DCM / MeOH = 30:1), compound **D3** was obtained as a purple solid (200 mg).

Yield: 76.9%

$\mathbf{R_f}$ **=** 0.220 (MeOH / DCM = 1:30)

^{1}H NMR (500 MHz, Methanol-d_4) δ 8.49 (d, J = 15.8 Hz, 1H), 8.12 (d, J = 8.4 Hz, 1H), 7.94 (d, J = 8.2 Hz, 1H), 7.88 (d, J = 8.5 Hz, 1H), 7.80 – 7.76 (m, 1H), 7.70 (d, J = 8.5 Hz, 1H), 7.58 – 7.53 (m, 1H), 7.45 – 7.39 (m, 3H), 7.10 – 7.07 (m, 1H), 6.64 (d, J = 2.3 Hz, 1H), 6.55 (d, J = 8.5 Hz, 1H), 6.39 – 6.34 (m, 2H), 2.55 – 2.50 (m, 2H), 2.45 – 2.38 (m, 1H), 2.26 – 2.18 (m, 1H), 1.84 – 1.71 (m, 2H), 1.69 (s, 3H), 1.71 (s, 3H).

^{13}C NMR (126 MHz, Methanol- d_4): δ 174.93, 153.77, 152.59, 150.30, 140.87, 138.28, 134.44, 134.22, 132.27, 129.34, 129.29, 128.86, 128.67, 128.42, 128.22, 127.29, 126.95, 126.19, 123.96, 122.55, 122.33, 118.35, 115.94, 111.10, 110.48, 110.02, 101.21, 53.76, 31.68, 29.39, 27.18, 24.08, 22.90, 20.61.

ESI-MS (*m/z*): $[M]^+$ calc'd. for $C_{36}H_{30}NO_4^+$ 540.2170, found 540.2178

Synthesis of compound D4

N-Methyl-2-methylbenzindolium-iodid (0.200 g, 0.583 mmol) and compound **1d** (0.200 g, 0.530 mmol) were added to anhydrous EtOH (10 mL), then piperdine was added in a nitrogen atmosphere. The mixture was heated to reflux for 8 h. The reaction mixture was cooled to room temperature. After addition of water to the reaction mixture, the whole mixture was extracted with DCM (15 mL×3). The extract was washed with water (10 mL×2) and brine (5 mL), The organic layer was dried over anhydrous Na_2SO_4, filtered, and then concentrated in vacuo. After purification by chromatography on a silica gel column (MeOH / DCM = 1:1), compound **D4** was obtained as a dark blue solid (186 mg).

Yield: 88.0%

R_f = 0.330 (MeOH / DCM = 1:1)

^{1}H NM (500 MHz, DMSO-d_6) δ 8.05 (d, J = 6.7 Hz, 2H), 8.00 (d, J = 7.4 Hz, 1H), 7.82 (d, J = 7.9 Hz, 2H), 7.54 (s, 2H), 7.41 (d, J = 7.6 Hz, 1H), 7.27 (d, J = 8.2 Hz, 1H), 7.16 – 7.08 (m, 1H), 6.87 (d, J = 7.3 Hz, 1H), 6.60 – 6.51 (m, 2H), 6.37 (s, 1H), 6.26 (d, J = 12.9 Hz, 1H), 3.51 (s, 3H), 2.79 – 2.67 (m, 2H), 1.99 – 1.90 (m, 1H), 1.83 – 1.77 (m, 1H), 1.72 – 1.66 (m, 2H).

^{13}C NMR (126 MHz, DMSO-d_6) δ 159.03, 154.84, 143.77, 131.37, 130.86, 129.89, 129.42, 128.69, 128.19, 126.83, 126.15, 123.84, 122.56, 117.86, 116.91, 115.78, 102.92, 102.52, 102.00, 49.01, 29.93, 25.41, 21.42.

ESI-MS (*m/z*): $[M]^+$ calc'd. for $C_{34}H_{26}NO_4^+$ 512.1857, found 512.1878

Synthesis of compound D4-a

N, N-phenylbistrifluoromethane-sulfonimide (0.614 g, 1.72 mmol) and triethylamine (326 μL, 2.34 mmol) were added to a solution of compound **D4** (1.00 g, 1.56 mmol) in DMF (10 mL), and the whole mixture was stirred at room temperature for 3 h. After addition of water to the reaction mixture, the whole mixture was extracted with AcOEt (25 mL×3). The extract was washed with water (20 mL×2) and brine (5 mL), The organic layer was dried over anhydrous Na_2SO_4, filtered, and then concentrated in vacuo. After purification by chromatography on a silica gel column (MeOH / DCM = 1:20), compound **D4-a** was obtained as a dark blue solid (880 mg).

Yield: 73.0%

R_f = 0.550 (MeOH / DCM = 1:20)

^{1}H NMR (500 MHz, Chloroform-*d*) δ 8.03 – 7.97 (m, 2H), 7.90 (d, J = 12.5 Hz, 1H), 7.75 – 7.63 (m, 3H), 7.65 – 7.58 (m, 1H), 7.41 – 7.34 (m, 1H), 7.27 (d, J = 2.5 Hz, 1H), 7.27 – 7.21 (m, 1H), 7.20 (d, J = 8.3 Hz, 1H), 6.97 – 6.91 (m, 1H), 6.83 (d, J = 8.8 Hz, 1H), 6.56 (d, J = 7.3 Hz, 1H), 6.01 (d, J = 12.4 Hz, 1H), 3.66-3.64 (m, 1H), 3.44 (s, 3H), 2.80-7.78 (m, 1H), 2.62-2.60 (m, 1H), 2.12-2.10 (m, 1H), 1.81 – 1.71 (m, 2H).

^{13}C NMR (126 MHz, Chloroform-*d*) δ 168.47, 151.18, 150.47, 148.70, 146.59, 143.74, 143.34, 134.08, 131.04, 128.98, 128.90, 127.91, 127.63, 126.40, 125.74, 124.45, 124.29, 123.06, 122.32, 120.56, 120.08, 117.83, 115.44, 114.46, 109.07, 104.33, 98.81, 98.58, 83.01, 28.68, 24.40, 22.01, 20.81.

ESI-MS (*m/z*): $[M]^+$ calc'd. for $C_{35}H_{25}F_3NO_6S^+$ 644.1350, found 644.1365

Synthesis of compound D4-b

A mixture of compound **D4-a** (500 mg, 0.730 mmol), tert-butyl carbamate (103 mg, 0.880 mmol), Palladium (II) Acetate (16.5 mg, 0.074 mmol), (S)-(-)-2,2'-Bis(diphenylphosphino)-1,1'-binaphthyl (91.5 mg, 0.174 mmol), Cs_2CO_3 (264 mg, 0.810 mmol) and dry dioxane (10 mL) was stirred at 90 °C for 12 h in a nitrogen atmosphere. The reaction mixture was cooled to room temperature. After addition of water to the reaction mixture, the whole mixture was extracted with AcOEt (15 mL×3). The extract was washed with water (10 mL×2) and brine (5 mL), The organic layer was dried over anhydrous Na_2SO_4, filtered, and then concentrated in vacuo. After purification by chromatography on a silica gel column (MeOH / DCM = 1:1), compound **D4-b** was obtained as dark green solid (191 mg).

Yield: 40.0%

$\mathbf{R_f}$ = 0.250 (MeOH / DCM = 1:1)

^{1}H NMR (500 MHz, DMSO-d_6): δ 7.96 (d, J = 7.7 Hz, 2H), 7.85 (d, J = 12.7 Hz, 1H), 7.80-7.77 (m, 2H), 7.73 – 7.66 (m, 2H), 7.58 (d, J = 2.2 Hz, 1H), 7.42 – 7.36 (m, 1H), 7.33 (d, J = 7.7 Hz, 1H), 7.22-7.20 (m, 2H), 6.78-6.76 (m, 1H), 6.59 (d, J = 8.7 Hz, 1H), 6.21 (d, J = 12.6 Hz, 1H), 3.45 (s, 3H), 2.82-2.79 (m, 1H), 2.60-2.58 (m, 1H), 1.93 – 1.85 (m, 1H), 1.70 – 1.57 (m, 3H), 1.50 (s, 9H).

^{13}C NMR (126 MHz, DMSO-d_6): δ 169.47, 153.11, 152.18, 150.84, 147.34, 144.47, 144.15, 141.98, 135.96, 132.07, 131.17, 130.52, 129.87, 129.15, 128.52, 127.18, 126.80, 125.79, 125.34, 125.20, 124.00, 120.95, 120.79, 115.39, 114.85, 111.93, 105.94, 105.32, 101.17, 100.71, 85.33, 80.17, 63.25, 29.72, 28.55, 25.19, 23.19, 22.01.

ESI-MS (*m/z*): $[M]^+$ calc'd. for $C_{39}H_{35}N_2O_5^+$ 611.2541, found 611.2652

Synthesis of compound BocNH-D4

A mixture of compound **D4-b** (467 mg, 0.720 mmol), N, N-dimethylsulfamide (448 mg, 3.61 mmol), n-(3-dimethylaminopropyl)-n'-ethylcarbodiimide hydrochloride (553 mg, 2.89 mmol), 4-dimethylaminopyridine (353 mg, 2.89 mmol) and dry DCM (10 mL) was stirred at 60 °C for 12 h in a nitrogen atmosphere. The reaction mixture was cooled to room temperature. After addition of water to the reaction mixture, the whole mixture was extracted with AcOEt (15 mL×3). The extract was washed with water (10 mL×2) and brine (5 mL), The organic layer was dried over anhydrous Na_2SO_4, filtered, and then concentrated in vacuo. After purification by chromatography on a silica gel column (PE / AcOEt = 5:1), compound **BocNH-D4** was obtained as orange solid (200 mg).

Yield: 40.0%

$\mathbf{R_f}$ = 0.500 (PE / AcOEt = 5:1)

^{1}H NMR (500 MHz, DMSO-d_6) δ 9.68 (s, 1H), 7.96 (d, J = 7.7 Hz, 2H), 7.85 (d, J = 12.7 Hz, 1H), 7.83 – 7.76 (m, 1H), 7.74 – 7.65 (m, 2H), 7.60 – 7.55 (m, 1H), 7.41 – 7.37 (m, 1H), 7.33 (d, J = 7.7 Hz, 1H), 7.26 – 7.17 (m, 2H), 6.80 – 6.74 (m, 1H), 6.59 (d, J = 8.7 Hz, 1H), 6.21 (d, J = 12.6 Hz, 1H), 3.45 (s, 3H), 2.85 – 2.77 (m, 1H), 2.63 – 2.54 (m, 1H), 1.94 – 1.85 (m, 1H), 1.70 – 1.58 (m, 3H), 1.50 (s, 9H).

^{13}C NMR (126 MHz, Chloroform-*d*) δ 166.32, 151.41, 150.73, 146.74, 143.53, 142.87, 138.52, 133.56, 131.20, 128.04, 127.80, 127.68, 127.66, 127.24, 124.49, 123.83, 123.12, 122.84, 120.02, 119.35, 113.90, 112.23, 104.78, 104.26, 99.56, 98.03, 79.91, 69.53, 36.93, 28.63, 27.31, 24.66, 22.58, 20.99.

ESI-MS (*m/z*): $[M+H]^+$ calc'd. for $C_{41}H_{40}N_4O_6S$ 717.2742, found 717.2748

Synthesis of compound NH_2-D4

Compound **BocNH-D4** (200 mg, 0.324 mmol) was added in a solution of trifluoroacetic acid in DCM (10 mL), the mixture was stirred at room temperature for 1 h. After addition of water to the reaction mixture, the whole mixture was extracted with AcOEt (15 mL×3). The extract was washed with water (10 mL×2) and brine (5 mL), The organic layer was dried over anhydrous Na_2SO_4, filtered, and then concentrated in vacuo. Then dark green solid compound **NH_2-D4** was afforded (160 mg) which was utilized in the next reaction without purification again.

Yield: 93.0%

R_f = 0.100 (MeOH / DCM = 1:1)

^{1}H NMR (500 MHz, Chloroform-*d*) δ 8.02 (d, *J* = 6.7 Hz, 1H), 7.93 – 7.85 (m, 2H), 7.68 – 7.58 (m, 3H), 7.55 – 7.48 (m, 1H), 7.37 – 7.30 (m, 1H), 7.19 – 7.11 (m, 2H), 7.10 – 7.04 (m, 1H), 6.63 (s, 1H), 6.57 (d, *J* = 8.6 Hz, 1H), 6.50 (d, *J* = 7.3 Hz, 1H), 6.00 (d, *J* = 12.5 Hz, 1H), 3.39 (s, 3H), 2.83 (s, 6H), 2.59 – 2.51 (m, 1H), 2.15 – 2.06 (m, 1H), 1.86 – 1.76 (m, 1H), 1.76 – 1.67 (m, 3H), 1.54 (s, 9H).

ESI-MS (*m/z*): $[M]^+$ calc'd. for $C_{36}H_{33}N_4O_4S^+$ 617.2218, found 617.2217

Synthesis of compound H_2O_2-D4

Compound **NH_2-D4** (50.0 mg, 0.081 mmol) and triphosgene (8.02 mg, 0.027 mmol) and triethylamine (7.50 µL, 0.041mmol) were added sequentially to dry DCM (2 mL) and stirred on ice bath for 30 min then the reaction was allowed to warm to room temperature by removing the ice bath. The solvent was removed under vacuum condition, and the mixture compound **NCO-D4** was utilized in the next reaction without purification. And then, Compound **NCO-D4** and 4-(4,4,5,5-Tetramethyl -1,3,2- dioxaborolane -2-yl) benzyl carbonochloridate (94.8 mg, 0.400 mmol) were added sequentially to dry DCM (2 mL) and stirred on ice bath for 30 min. Triethylamine (11.0 µL, 0.800 mmol) was added dropwise, and the solution was allowed to warm to room temperature and stirred for 1 h. After addition of water to the reaction mixture, the whole mixture was extracted with DCM (5 mL×3). The extract was washed with water (2 mL×2) and brine (3 mL), The organic layer was dried over anhydrous Na_2SO_4, filtered, and then concentrated in vacuo. Then the solvent was removed under vacuum condition. The mixture was purified by column chromatography (PE / AcOEt = 5:1) to give compound **H_2O_2-D4** as an orange product (35.0 mg).

Yield: 49.0%

R_f = 0.350 (PE / AcOEt = 5:1)

^{1}H NMR (500 MHz, Methanol-*d_4*): *δ* 7.96 (d, *J* = 6.8 Hz, 1H), 7.84 (d, *J* = 7.9 Hz, 2H), 7.74 (d, *J* = 7.6 Hz, 2H), 7.57 (m, 3H), 7.48-7.46 (m, 1H), 7.35 (d, *J* = 7.6 Hz, 1H), 7.28 (m, 1H), 7.12 (m, 2H), 7.06 (m, 2H), 6.73 (d, *J* = 8.5 Hz, 1H), 6.51 (d, *J* = 8.6 Hz, 1H), 6.45 (s, 1H), 5.95 (d, *J* = 12.5 Hz, 1H), 5.16 – 5.24 (m, 2H), 3.26 (s, 6H), 2.75 (s, 3H), 2.61-2.54 (m, 1H), 2.64 – 2.44 (m, 1H), 2.07 – 1.95 (m, 1H), 1.76 – 1.62 (m, 2H), 1.55-1.46 (m, 1H), 1.28 (s, 12H).

^{13}C NMR (126 MHz, Chloroform-*d*) δ 167.31, 152.33, 147.79, 144.57, 144.00, 135.10, 135.06, 134.63, 131.05, 129.11, 128.80, 128.42, 127.37, 126.07, 124.93, 124.17, 123.90, 121.11, 120.46, 115.01, 114.48, 105.28, 100.52, 99.06, 83.91, 83.82, 67.07, 65.32, 37.9, 29.71, 24.87, 23.61, 22.01.

ESI-MS (*m/z*): $[M+H]^+$ calc'd. for $C_{50}H_{49}BN_4O_8S$ 877.3437, found 877.3445

Synthesis of compound H_2S-D4

Compound **NCO-D4** and 4-azidobenzyl alcohol (59.6 mg, 0.400 mmol) were added sequentially to dry DCM (2 mL) and stirred on ice for 30 min. Triethylamine (11.0 μL, 0.800 mmol) was added dropwise, and the solution was allowed to warm to room temperature and stirred for 4 h. After addition of water to the reaction mixture, the whole mixture was extracted with DCM (5 mL×3). The extract was washed with water (2 mL×2) and brine (3 mL), The organic layer was dried over anhydrous Na_2SO_4, filtered, and then concentrated in vacuo. The mixture was purified by column chromatography (PE / AcOEt = 5:1) to give compound **H_2S-D4** as an orange product (35.0 mg).

Yield: 62.5%

R_f = 0.330 (PE / AcOEt = 5:1)

^{1}H NMR (500 MHz, Chloroform-*d*): *δ* 8.03 (d, *J* = 7.0 Hz, 1H), 7.95 – 7.87 (m, 2H), 7.70 – 7.59 (m, 3H), 7.55 – 7.50 (m, 1H), 7.45 – 7.38 (m, 2H), 7.38 – 7.32 (m, 1H), 7.30 (d, *J* = 2.2 Hz, 1H), 7.20 – 7.13 (m, 2H), 7.04 (d, *J* = 8.2 Hz, 2H), 6.76 (s, 1H), 6.60 (d, *J* = 8.6 Hz, 1H), 6.53-6.51 (m, 1H), 6.02 (d, *J* = 12.5 Hz, 1H), 5.31 – 5.15 (m, 2H), 3.42 (s, 3H), 2.84 (s, 6H), 2.83 – 2.80 (m, 1H), 2.61-2.48 (m, 1H), 2.15 – 2.06 (m, 1H), 1.86 – 1.75 (m, 1H), 1.76 – 1.66 (m, 1H), 1.54 – 1.51(m, 1H).

^{13}C NMR (126 MHz, Chloroform-*d*) δ 167.31, 167.22, 152.35, 151.46, 147.81, 144.20, 136.10, 134.77, 132.14, 131.05, 130.08, 129.33, 129.13, 128.70, 125.39, 125.08, 124.91, 124.11, 124.05, 123.92, 121.12, 119.23, 117.49, 115.16, 115.00, 108.36, 105.27, 105.13, 100.52, 100.32, 99.20, 99.10, 70.00, 66.57, 37.96, 29.70, 25.65, 23.60, 21.94.

ESI-MS (*m/z*): $[M+H]^+$ calc'd. for $C_{44}H_{37}N_7O_6S$ 792.2599, found 792.2371

7. NMR trace

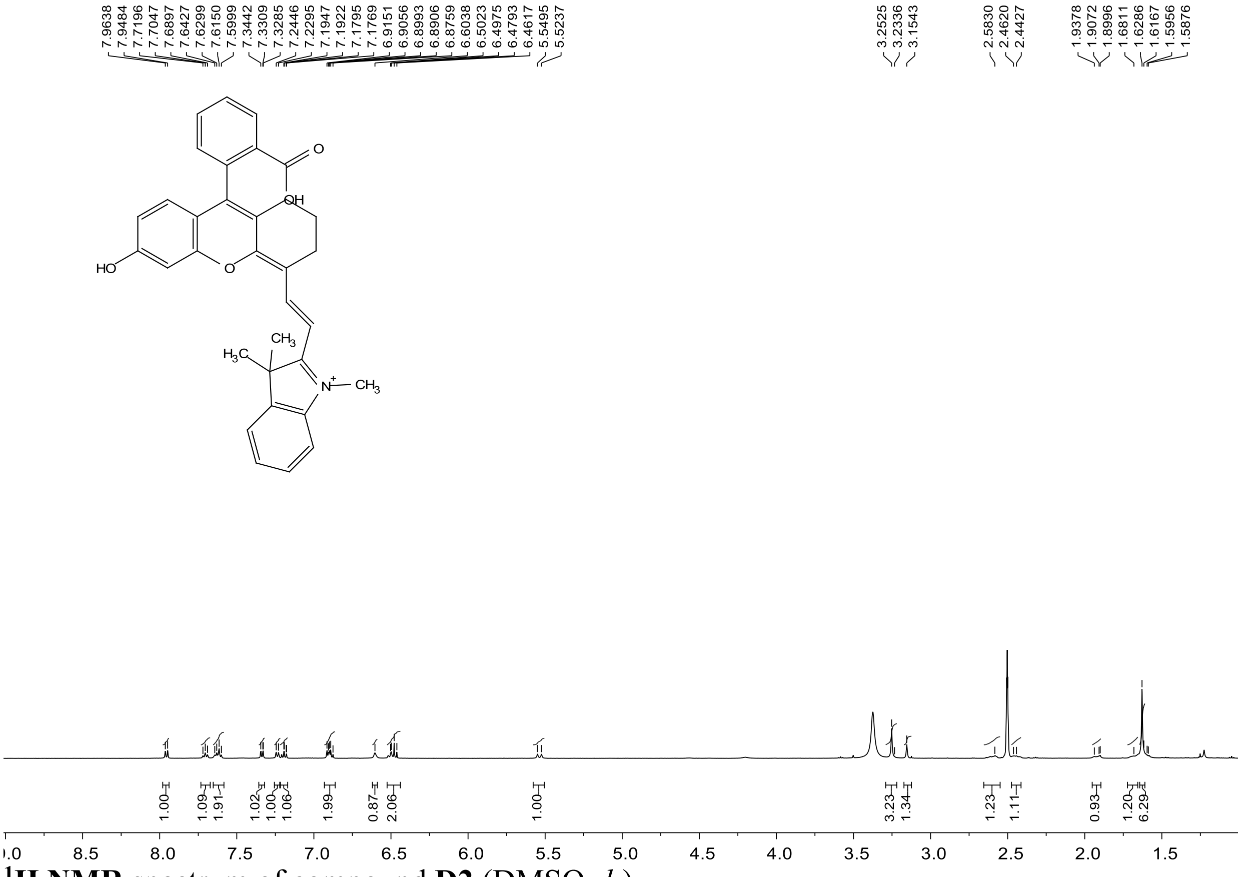

^{1}H NMR spectrum of compound **D2** (DMSO-d_6).

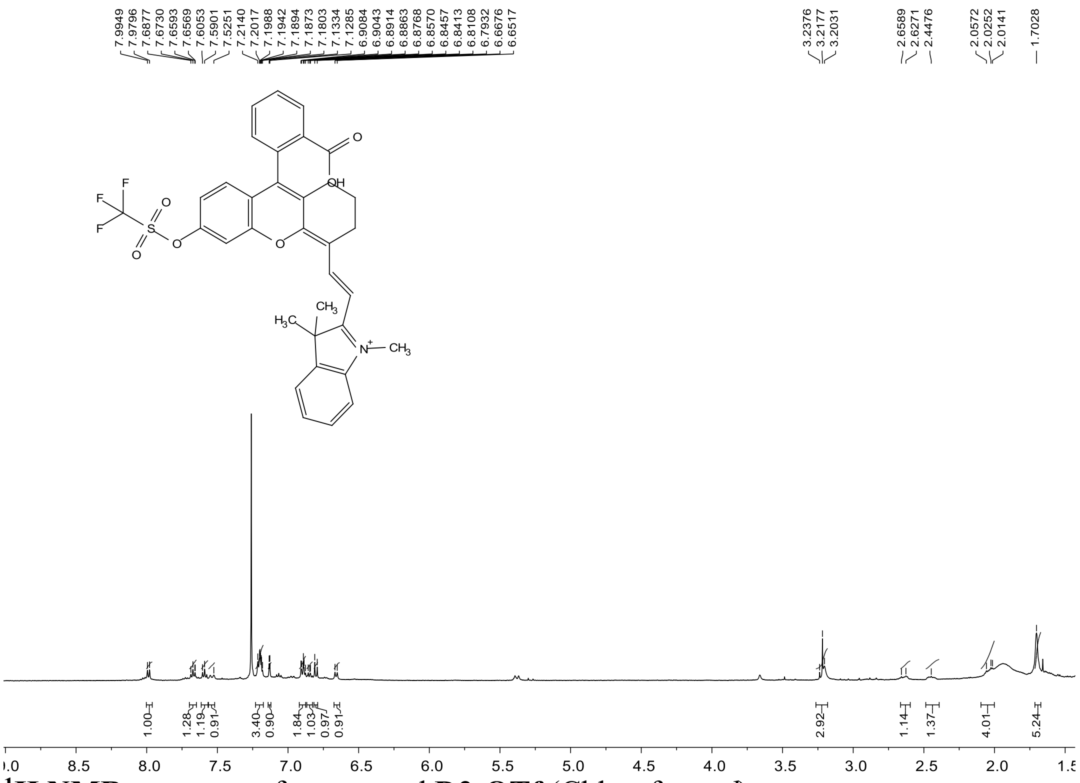

^{1}H NMR spectrum of compound **D2-OTf** (Chloroform-*d*).

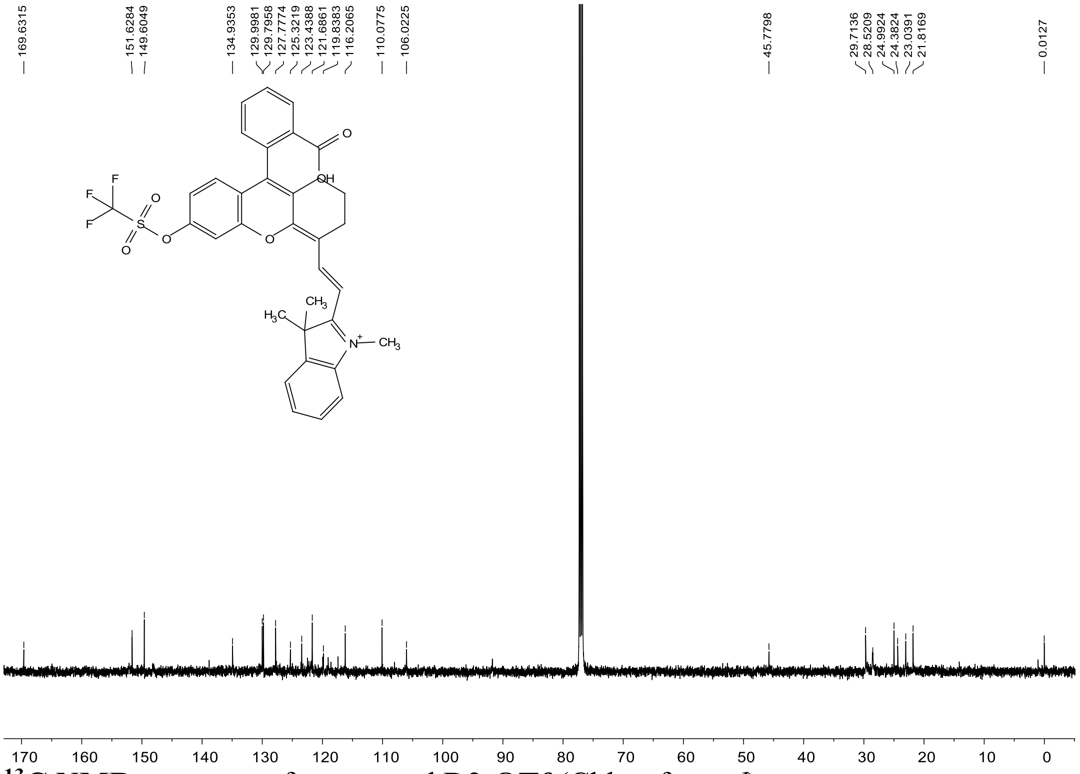

^{13}C NMR spectrum of compound **D2-OTf** (Chloroform-*d*).

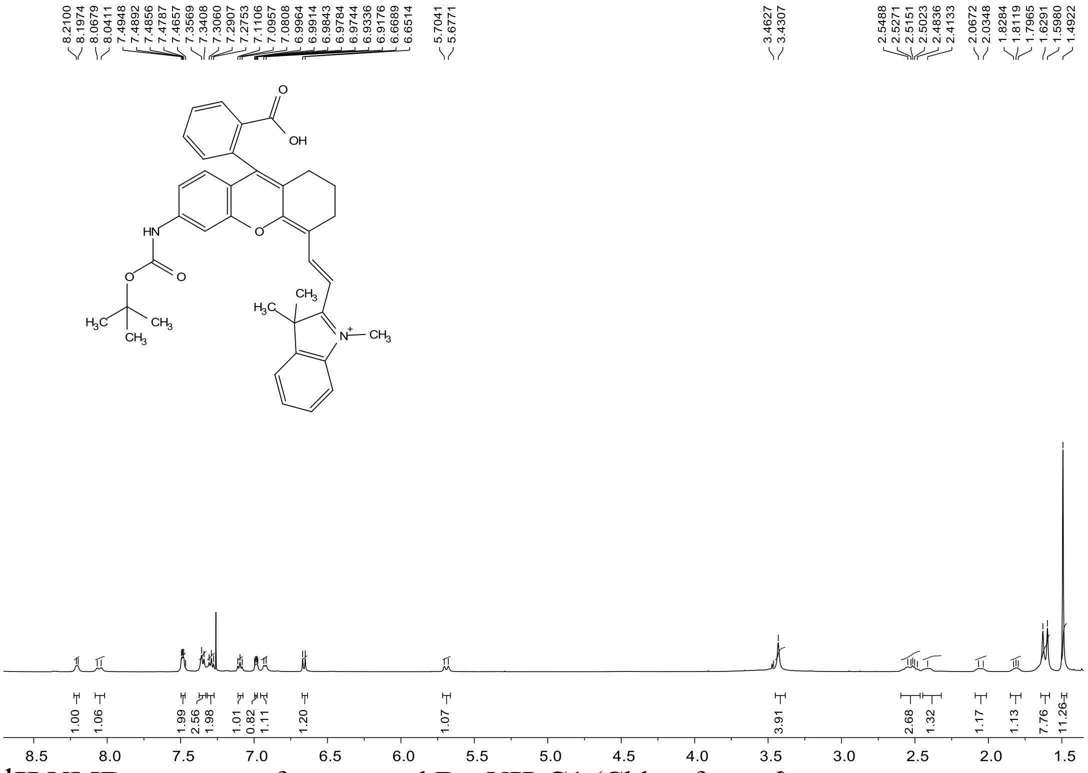

^{1}**H NMR** spectrum of compound **BocNH-C1** (Chloroform-*d*).

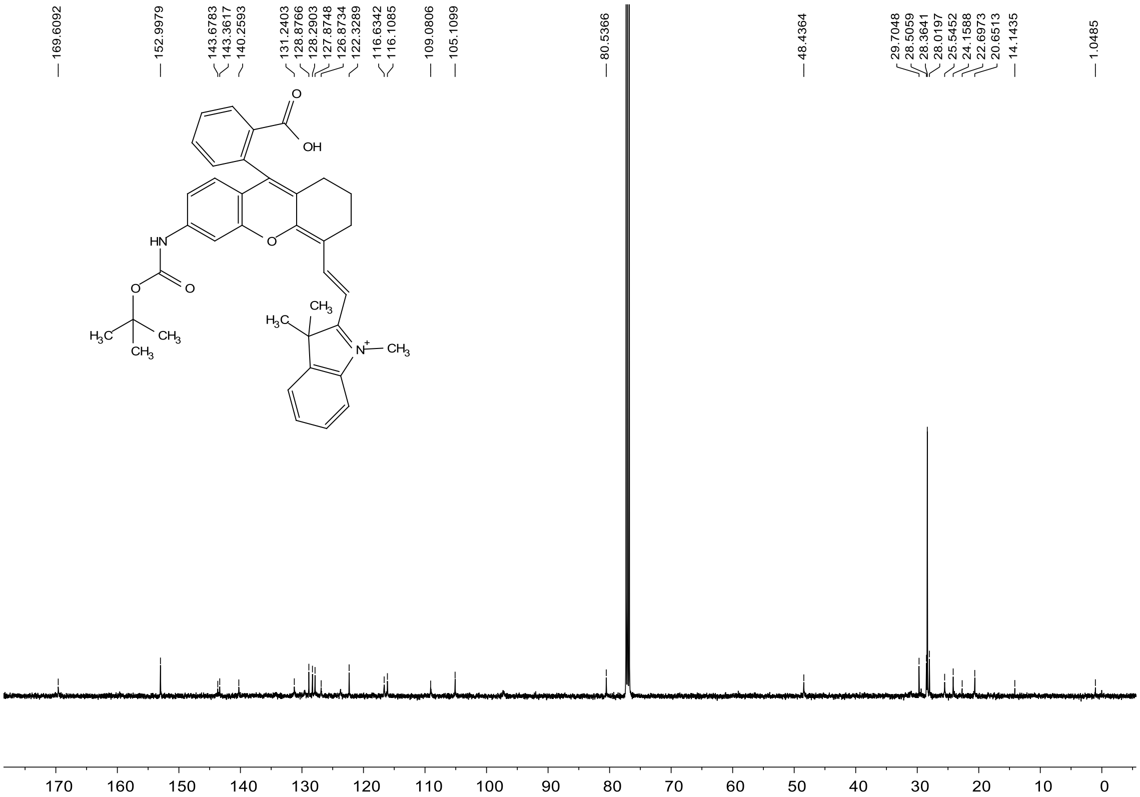

^{13}**C NMR** spectrum of compound **BocNH-C1** (Chloroform-*d*).

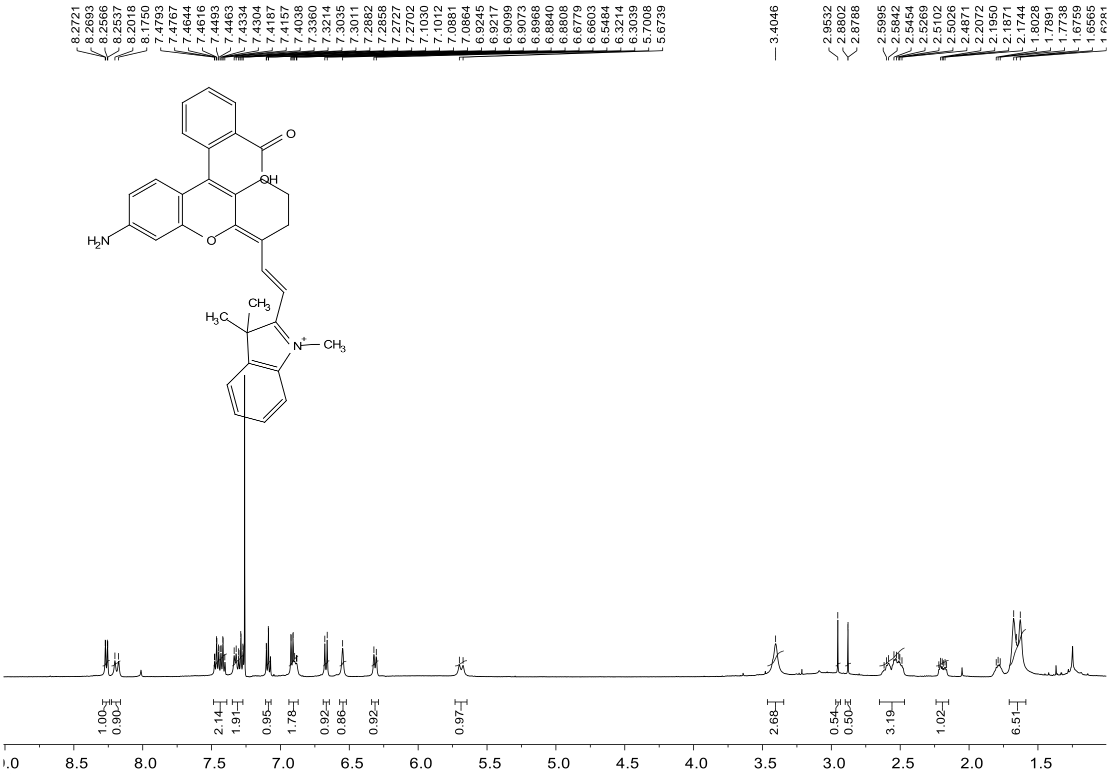

^{1}**H NMR** spectrum of compound **NH$_2$-C1** (Chloroform-*d*).

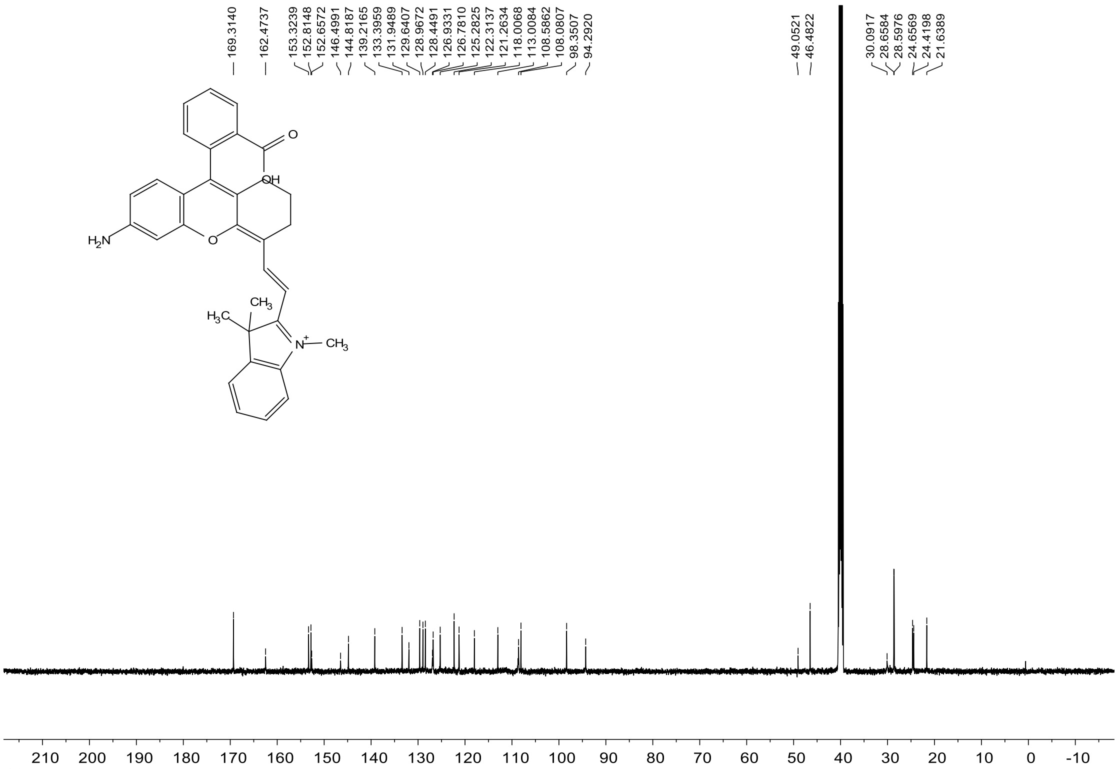

^{13}**C NMR** spectrum of compound **NH$_2$-C1** (DMSO-*d*$_6$).

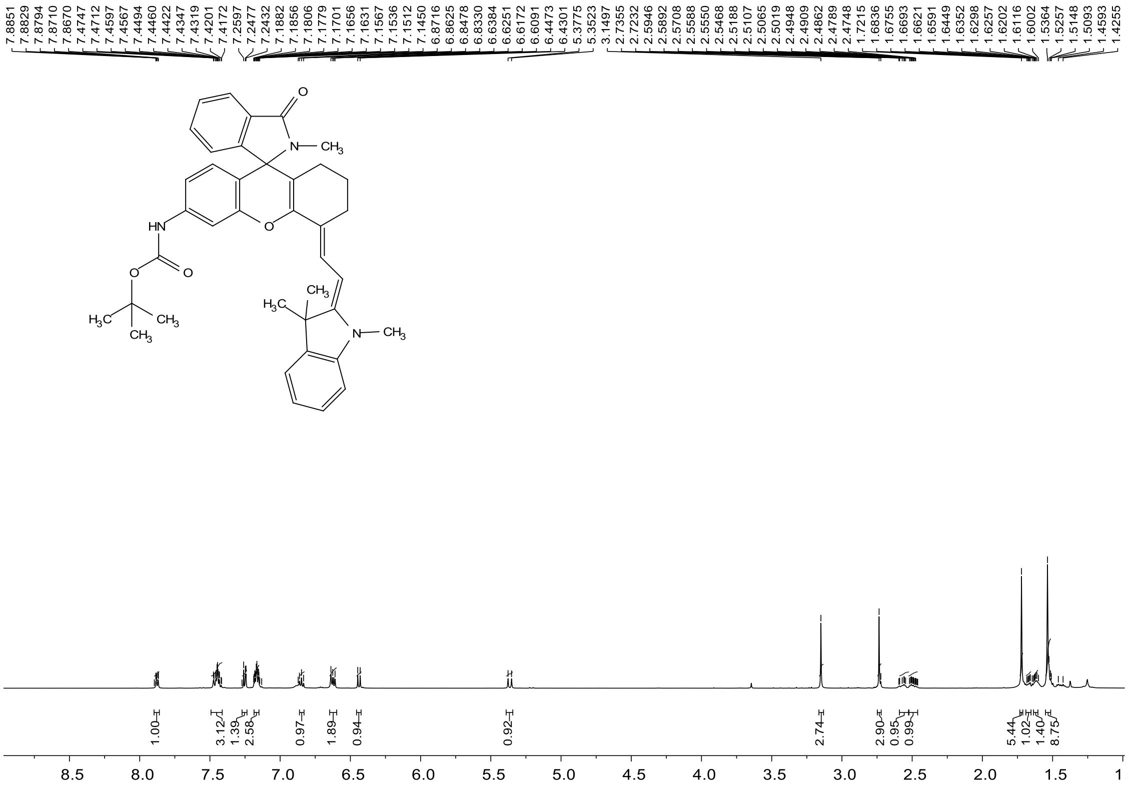

^{1}H NMR spectrum of compound **BocNH-C2** (Chloroform-*d*).

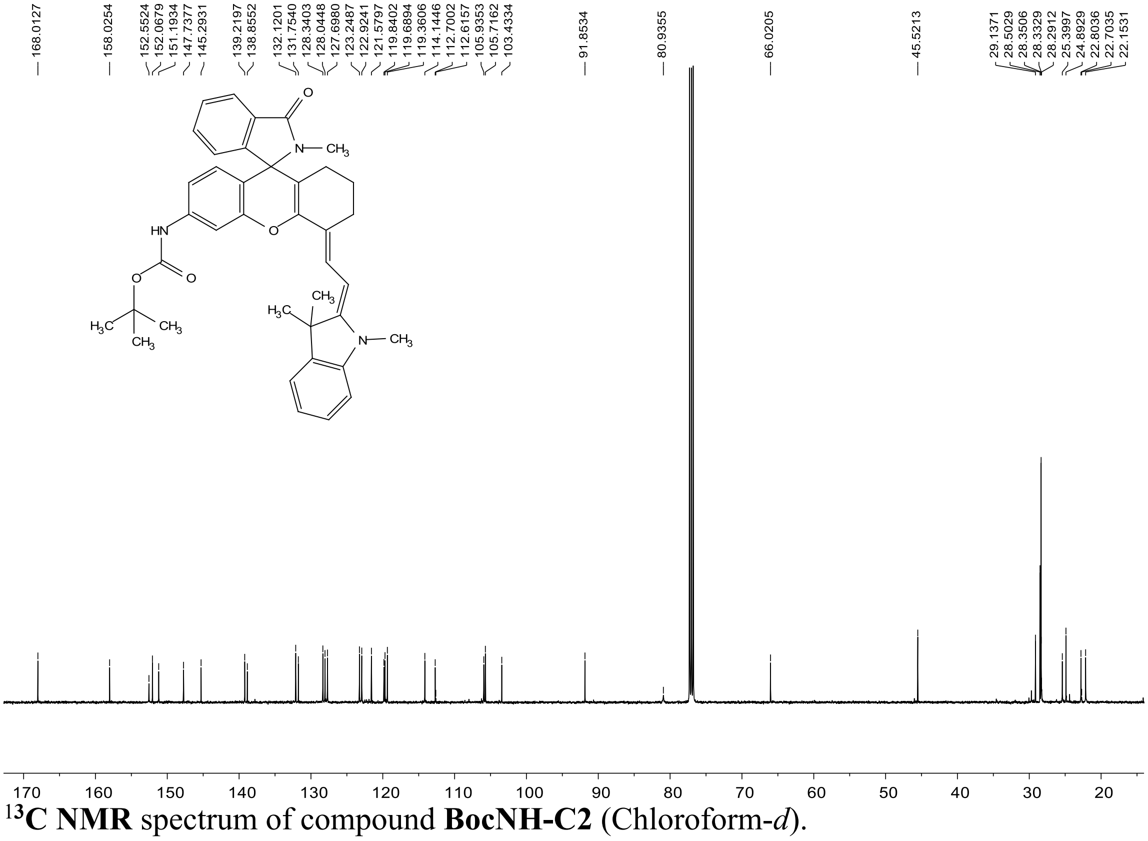

^{13}C NMR spectrum of compound **BocNH-C2** (Chloroform-*d*).

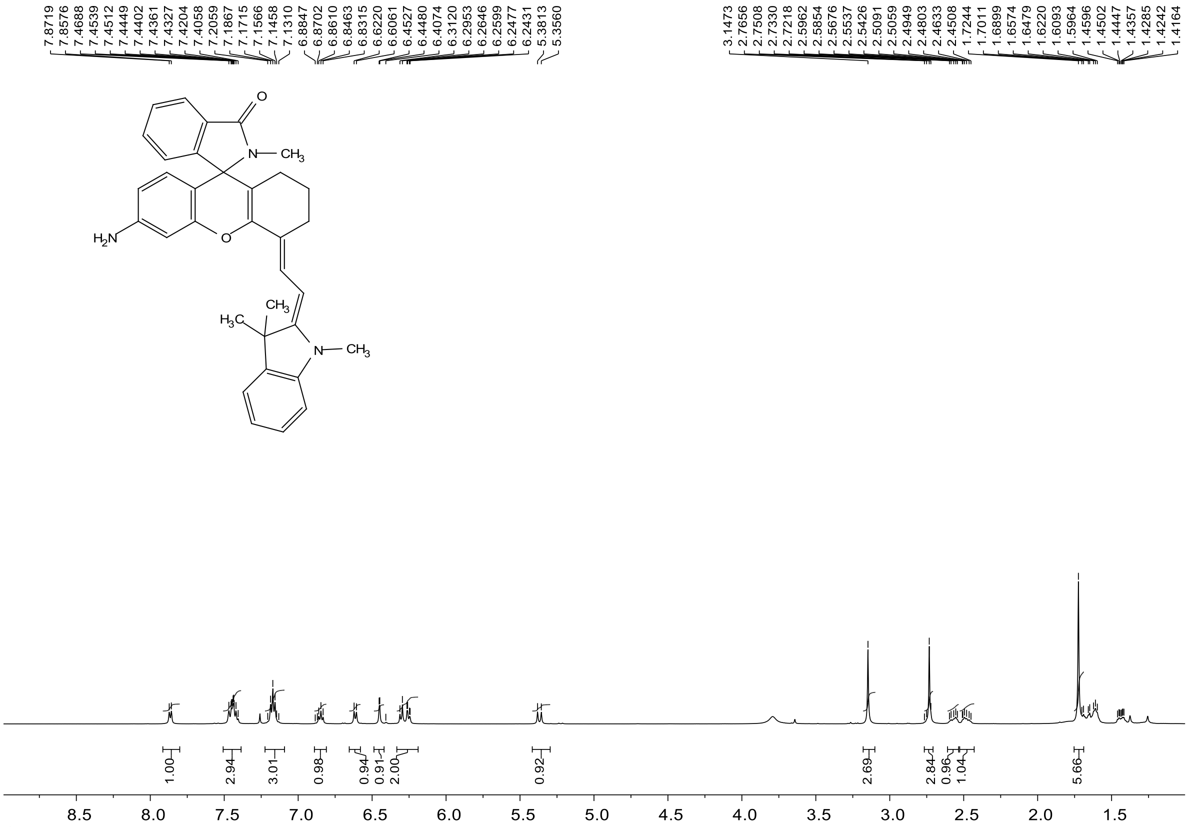

^{1}H NMR spectrum of compound **NH$_2$-C2** (Chloroform-*d*).

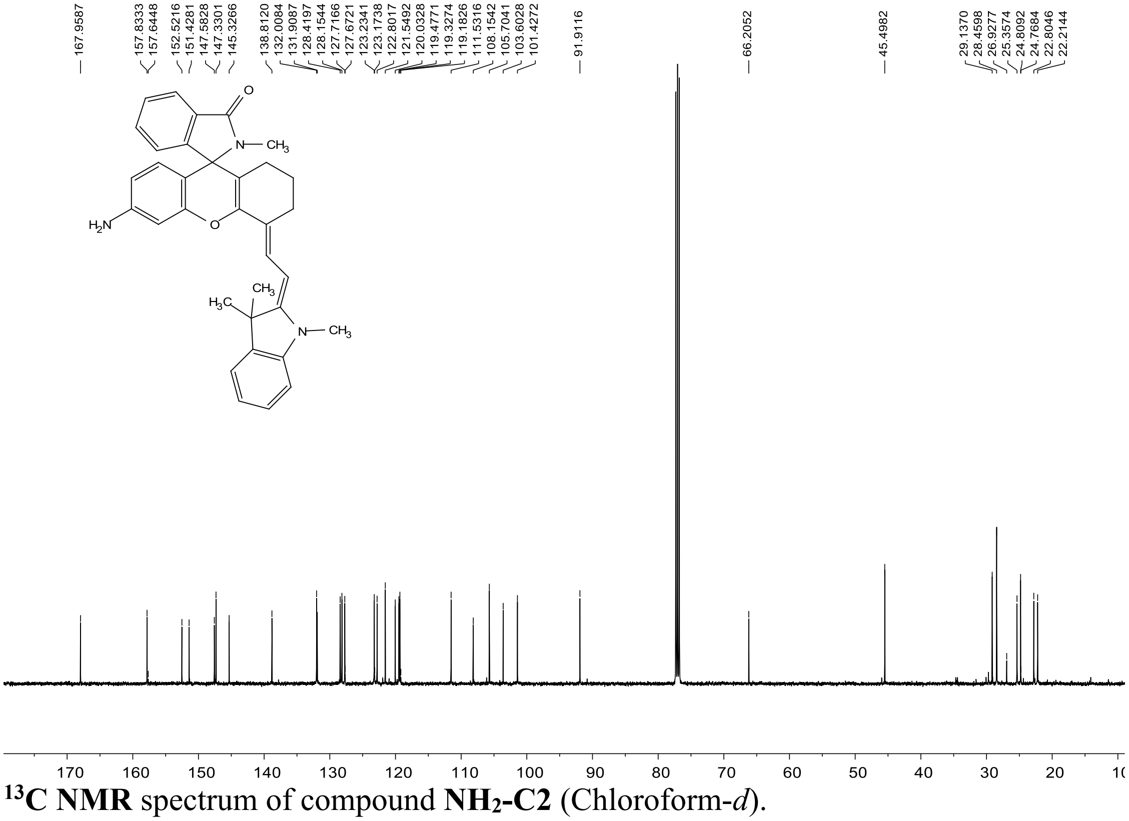

^{13}C NMR spectrum of compound **NH$_2$-C2** (Chloroform-*d*).

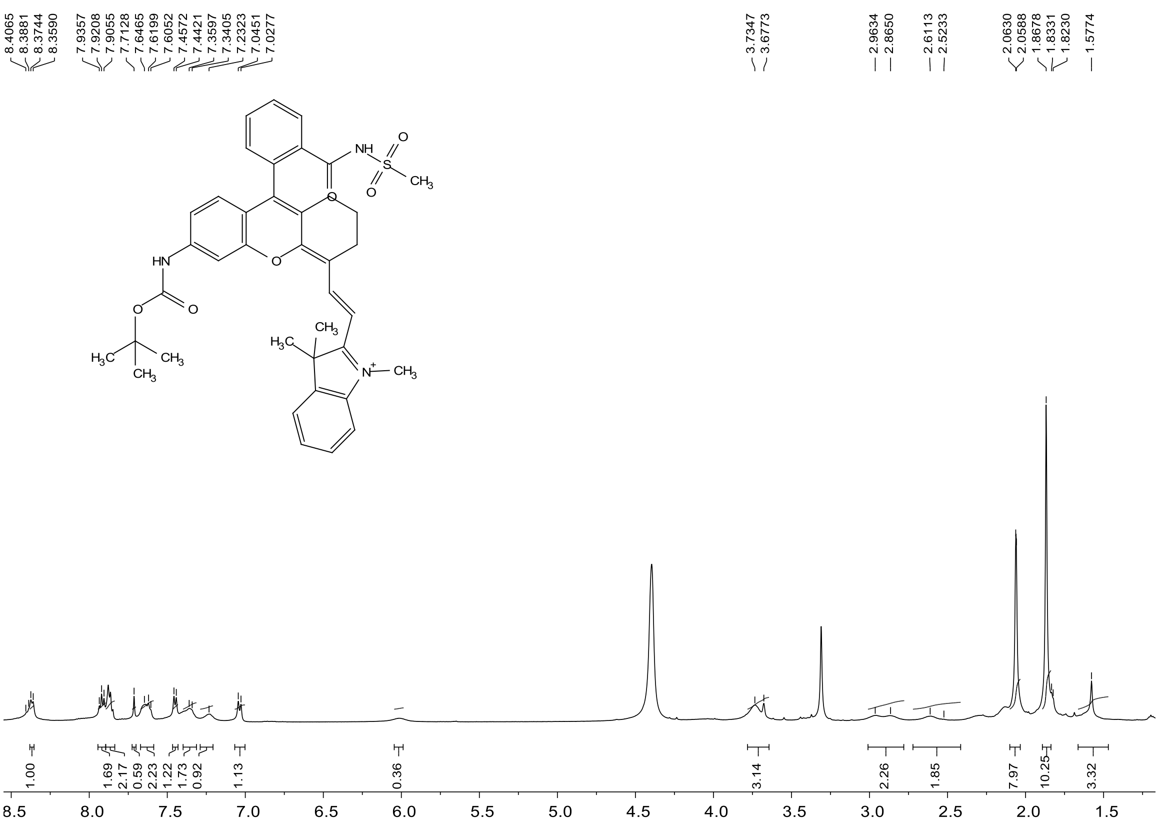

^{1}H NMR spectrum of compound **BocNH-C3** (Methanol-d_4).

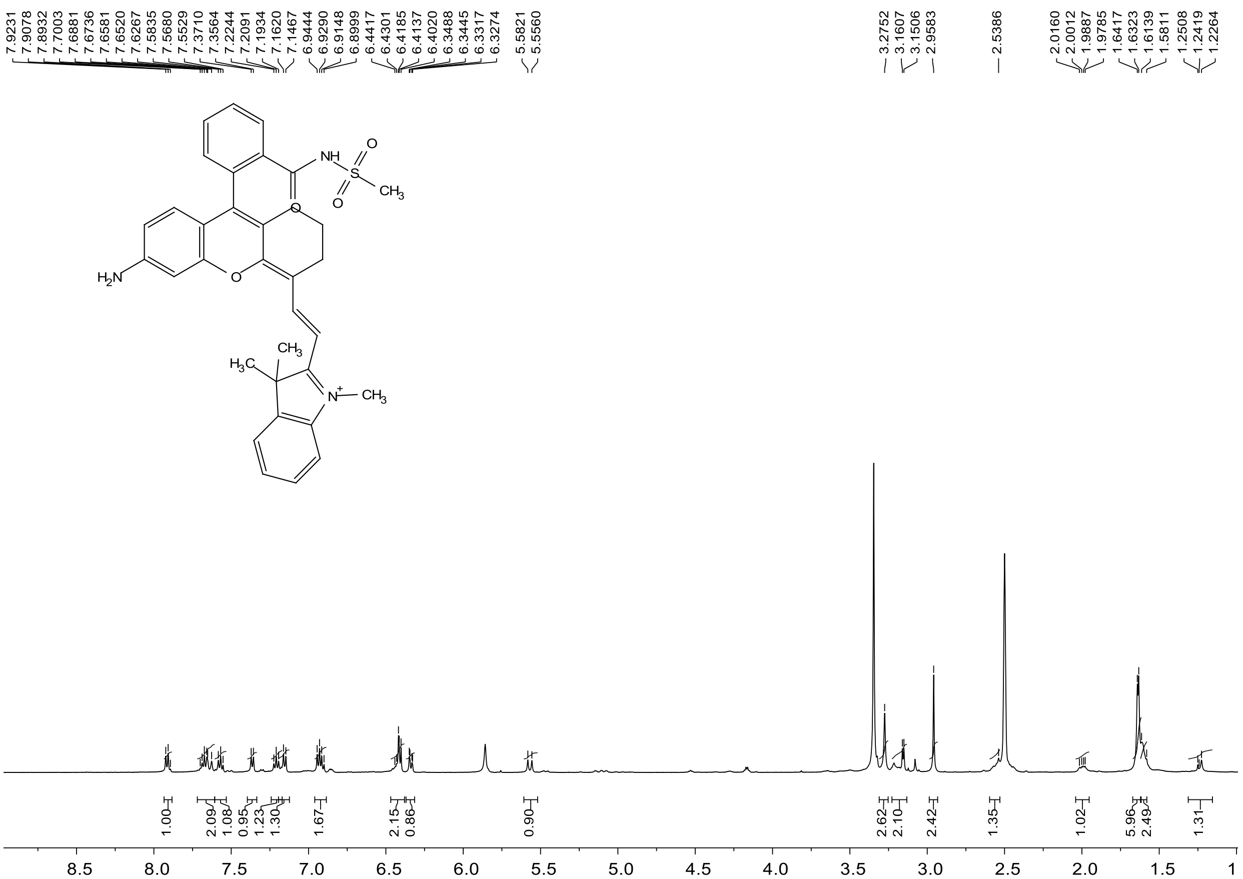

^{1}H NMR spectrum of compound **NH_2-C3** (DMSO-d_6).

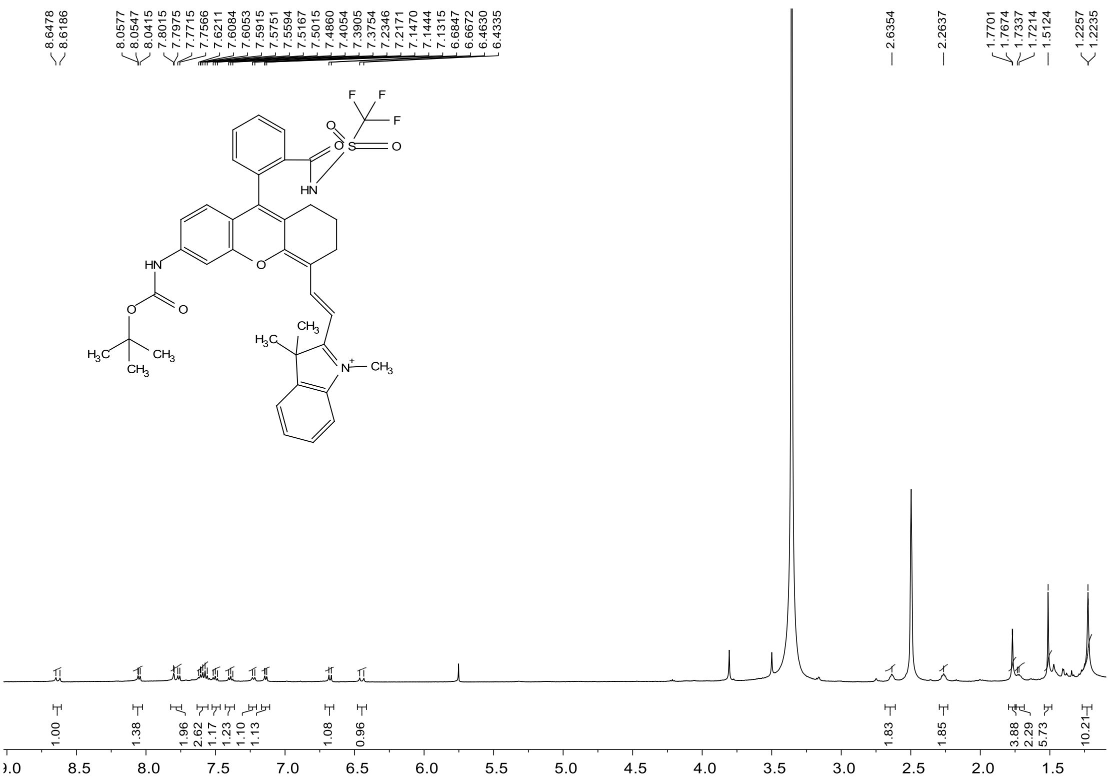

¹H NMR spectrum of compound **BocNH-C4** (DMSO-d_6)

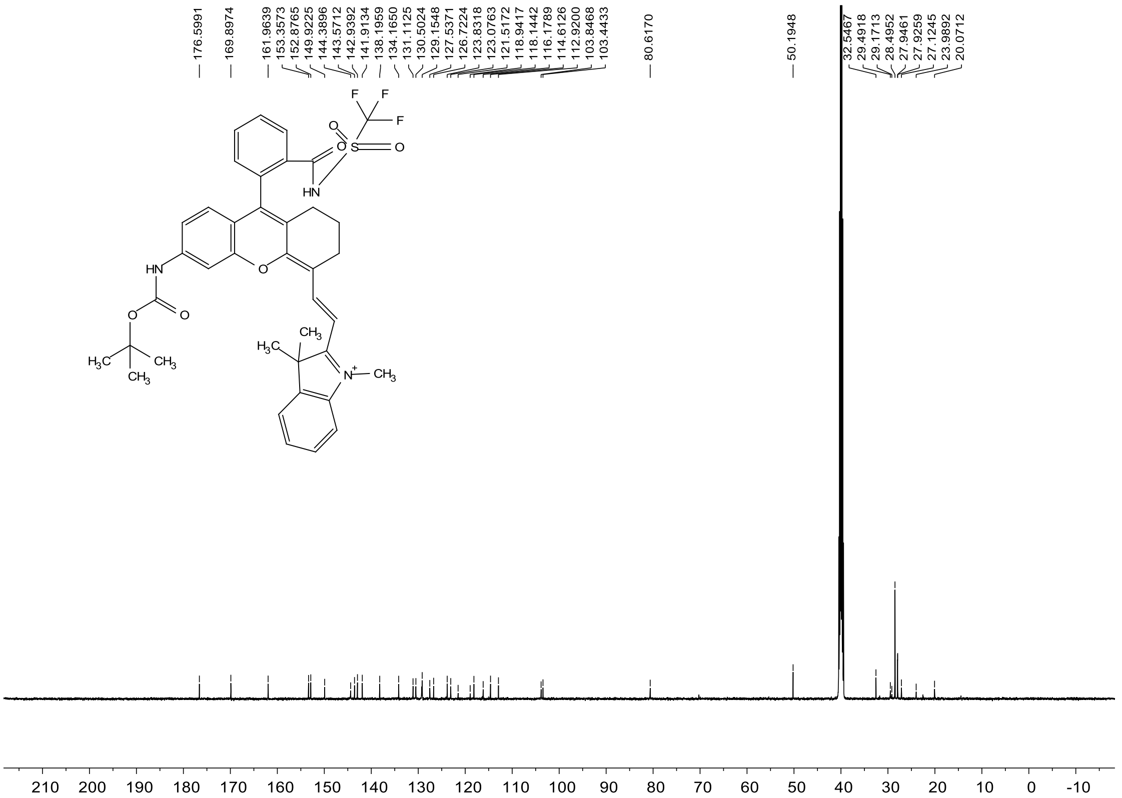

¹³C NMR spectrum of compound **BocNH-C4** (DMSO-d_6).

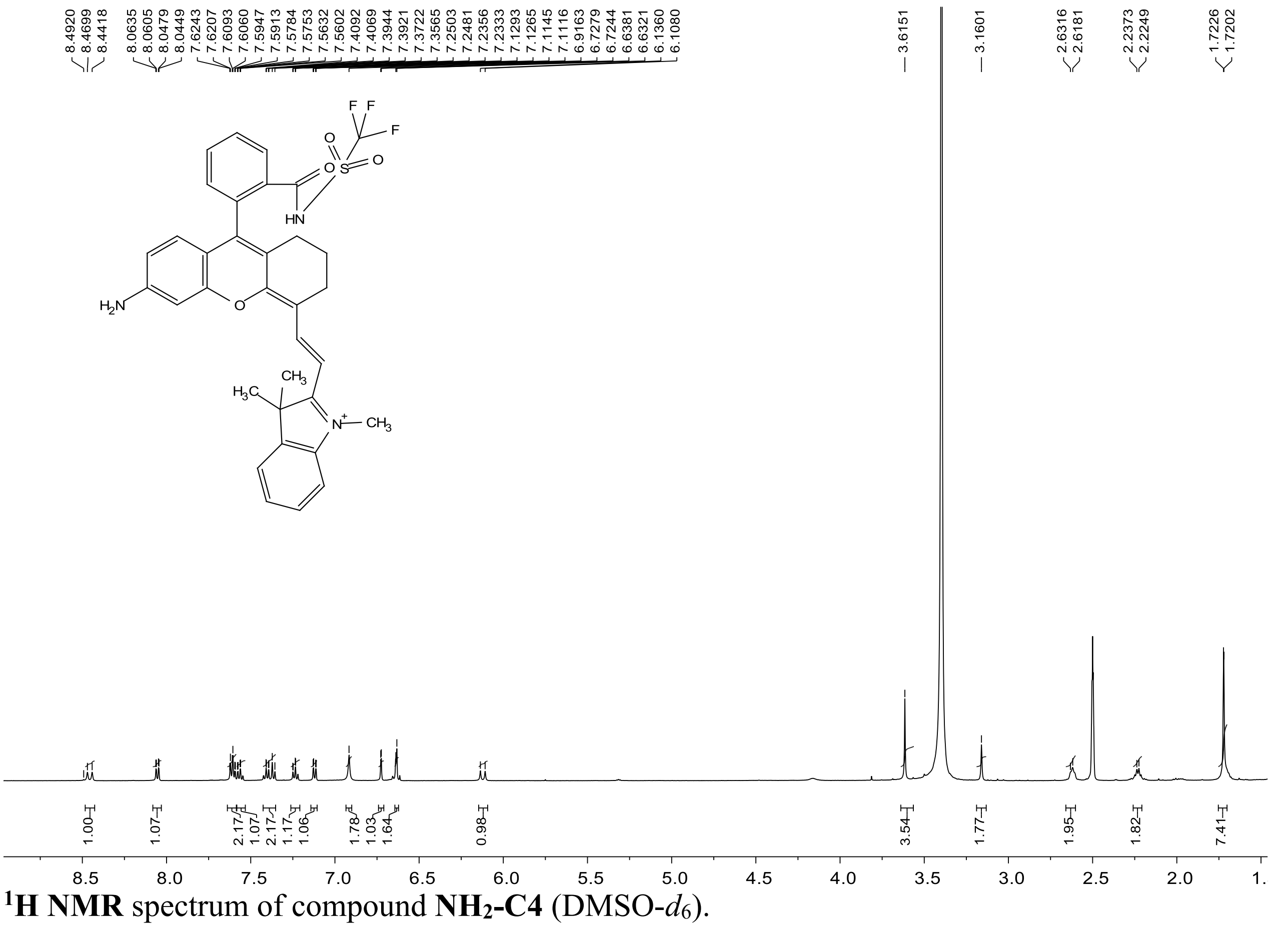

^{1}H NMR spectrum of compound **NH_2-C4** (DMSO-d_6).

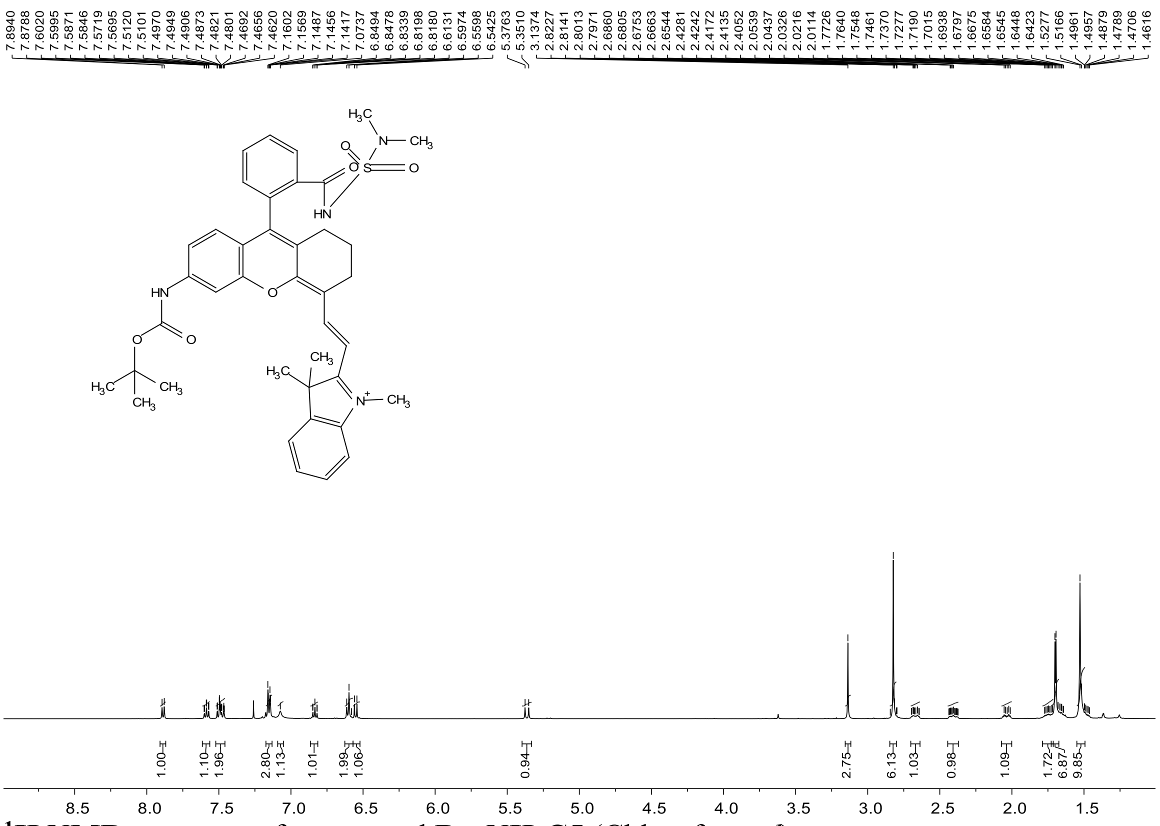

^{1}H NMR spectrum of compound **BocNH-C5** (Chloroform-*d*).

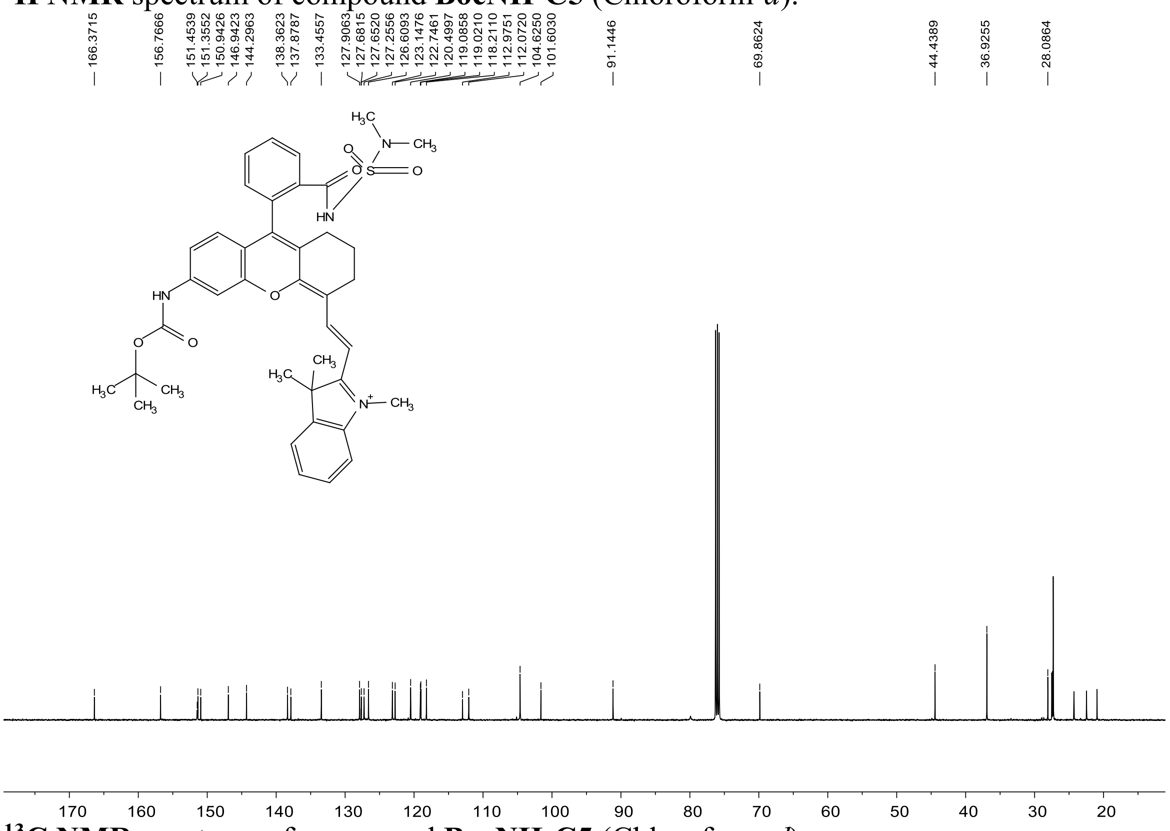

^{13}C NMR spectrum of compound **BocNH-C5** (Chloroform-*d*).

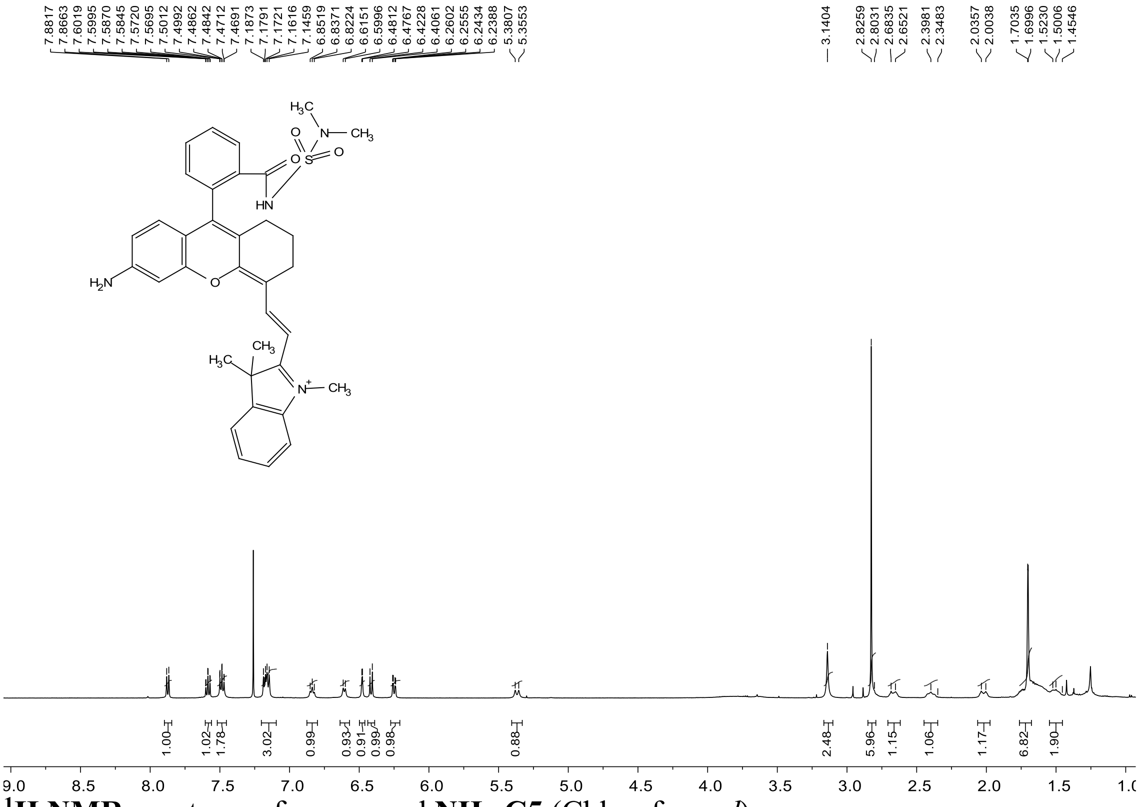

^{1}H NMR spectrum of compound **NH_2-C5** (Chloroform-*d*)

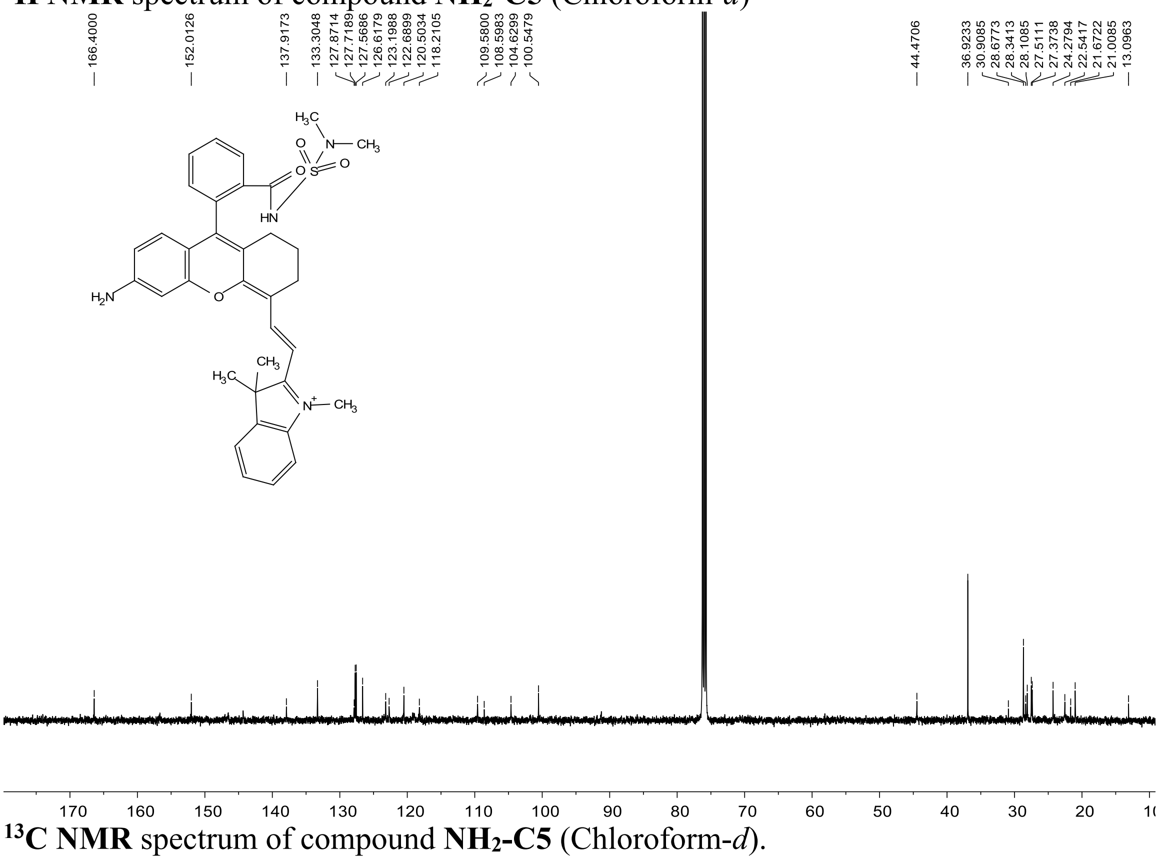

^{13}C NMR spectrum of compound **NH_2-C5** (Chloroform-*d*).

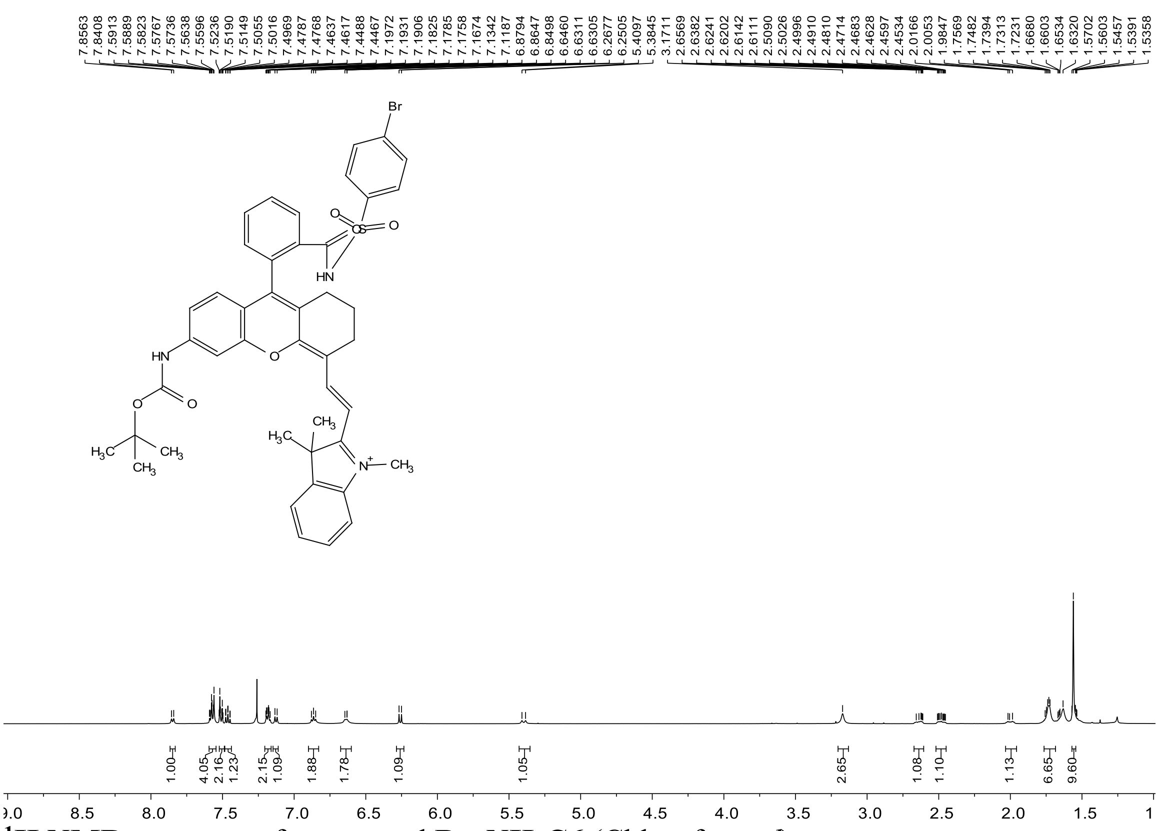

^{1}H NMR spectrum of compound **BocNH-C6** (Chloroform-*d*).

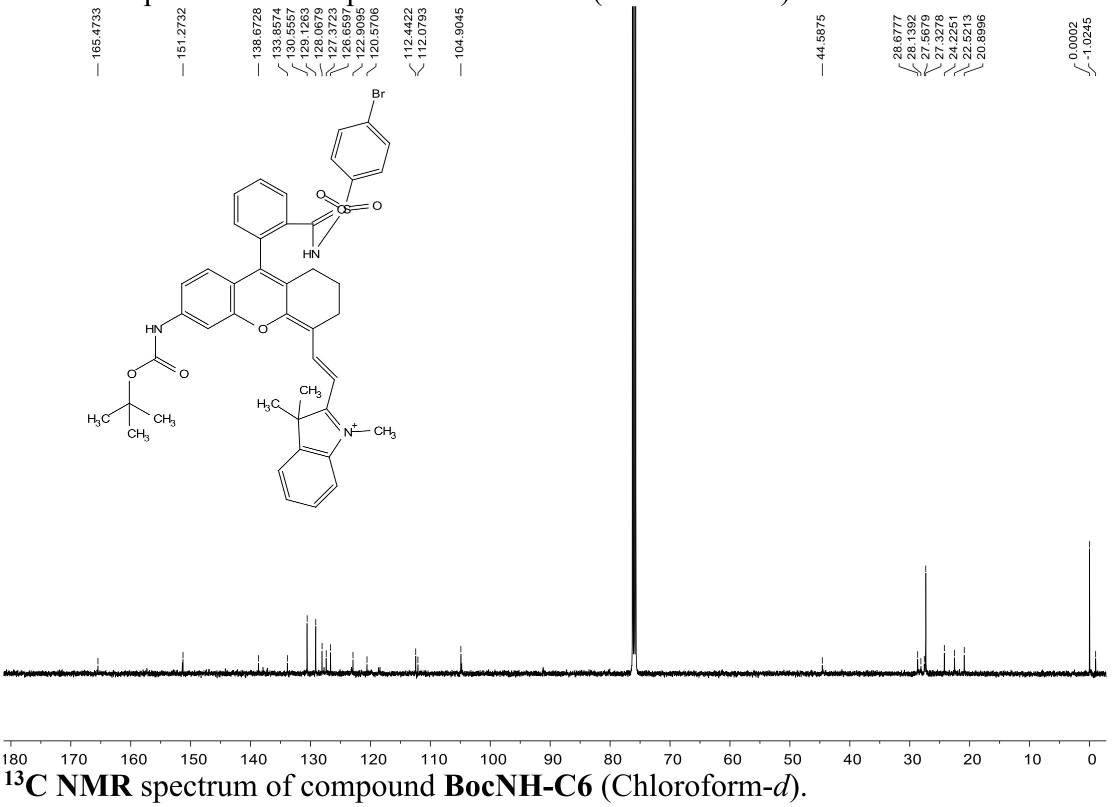

^{13}C NMR spectrum of compound **BocNH-C6** (Chloroform-*d*).

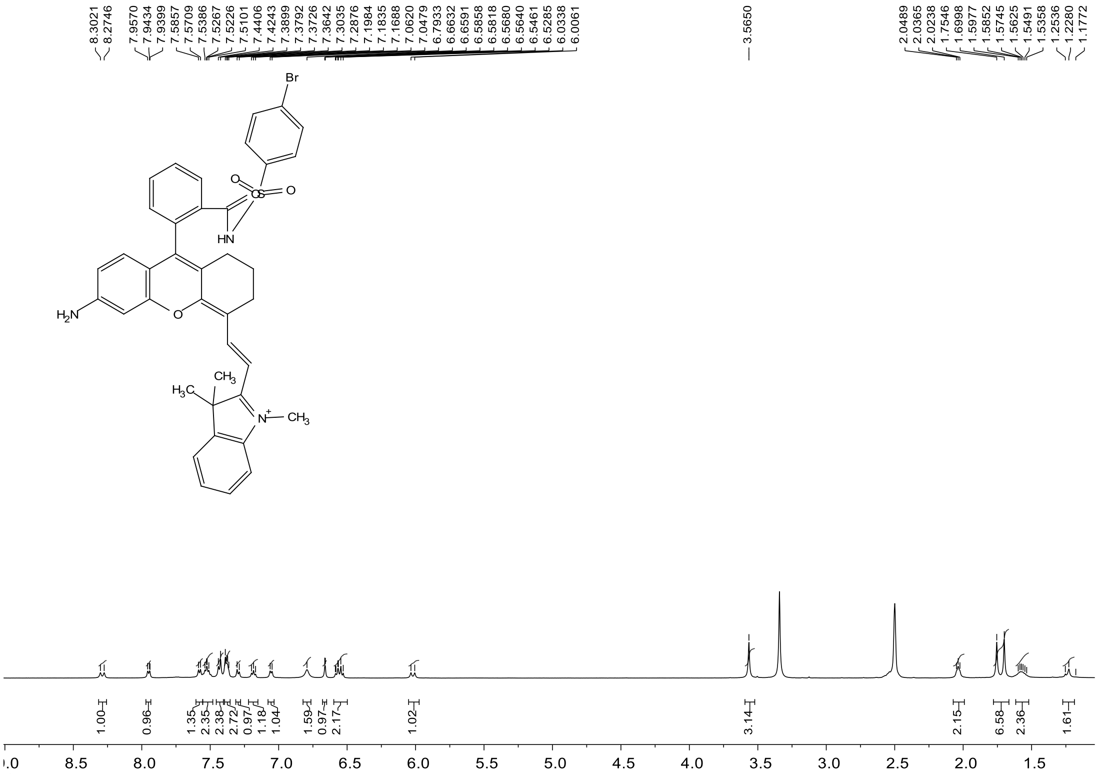

^{1}H NMR spectrum of compound **NH_2-C6** (DMSO-d_6).

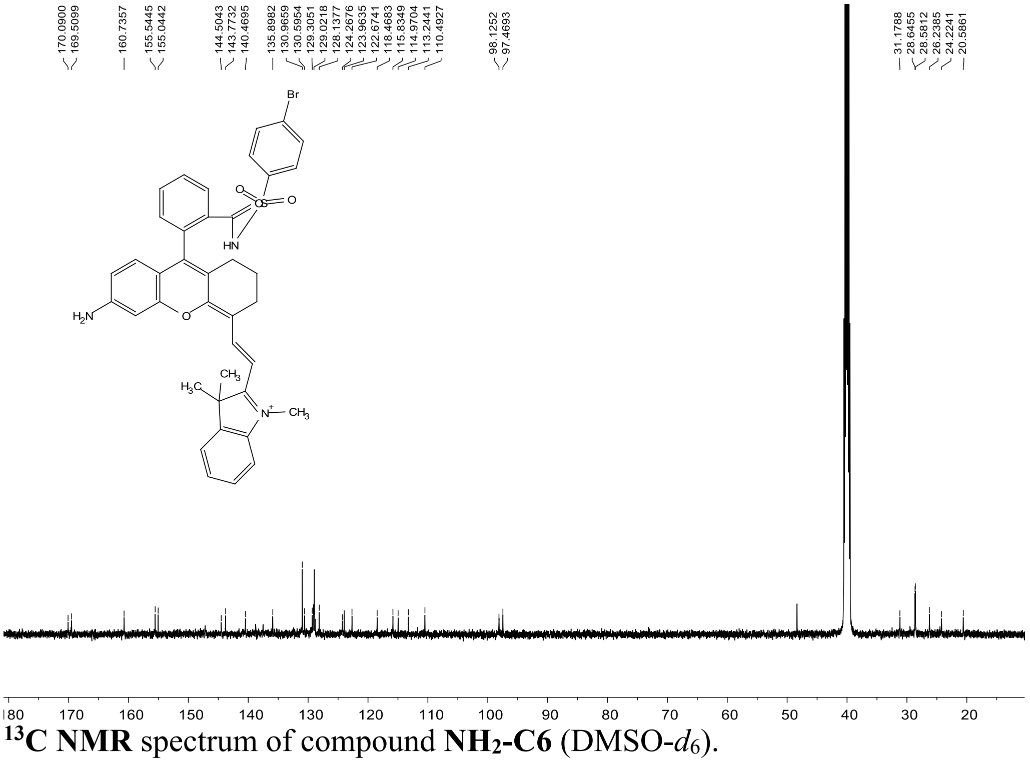

^{13}C NMR spectrum of compound **NH_2-C6** (DMSO-d_6).

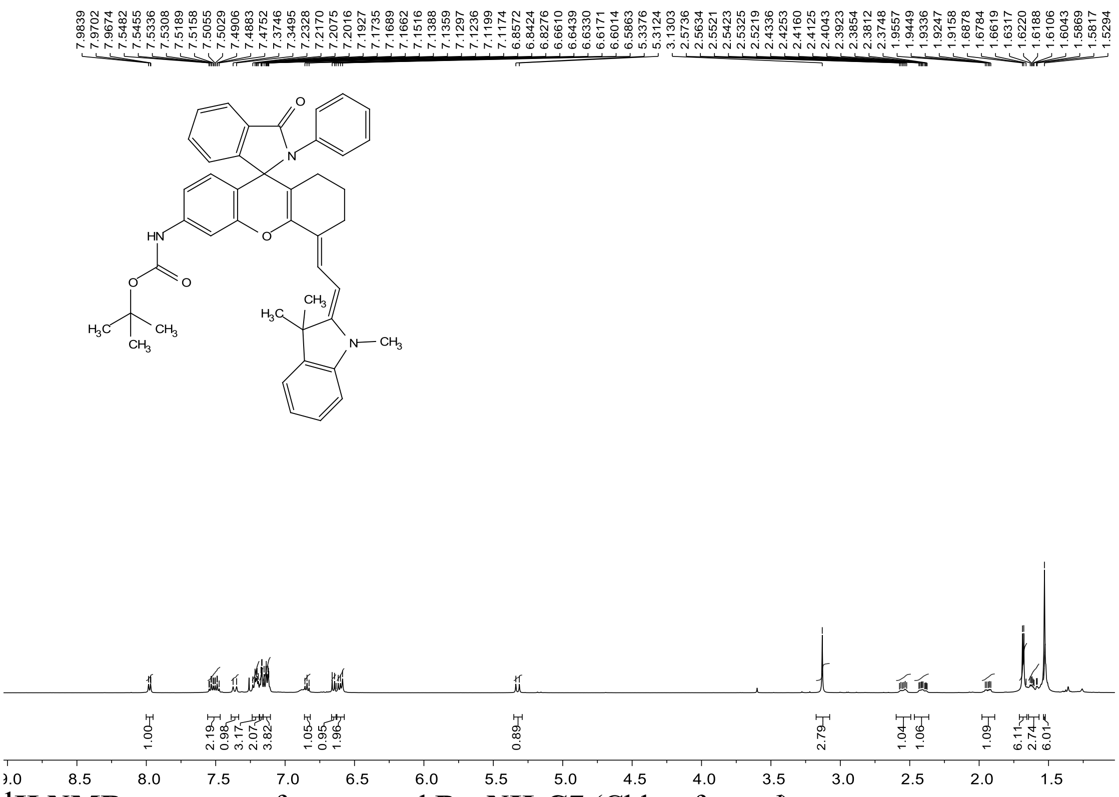

^{1}H NMR spectrum of compound **BocNH-C7** (Chloroform-*d*).

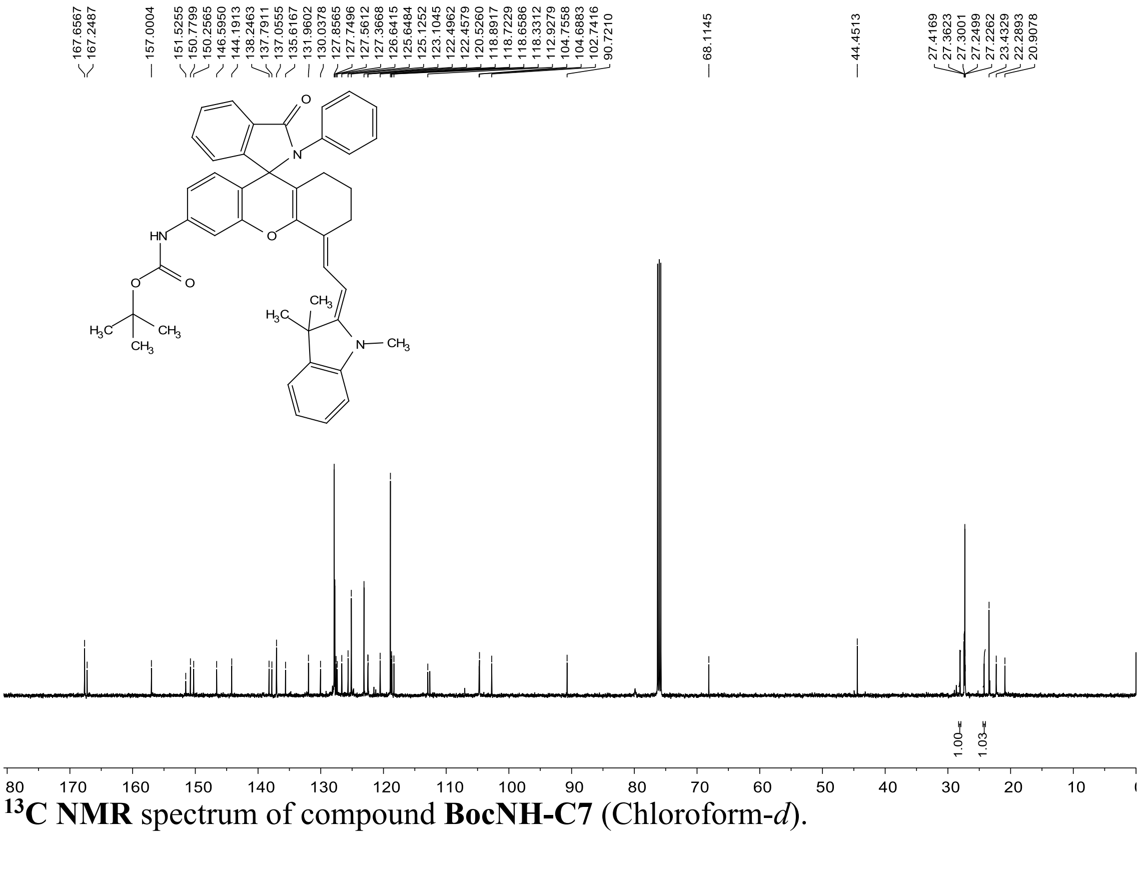

^{13}C NMR spectrum of compound **BocNH-C7** (Chloroform-*d*).

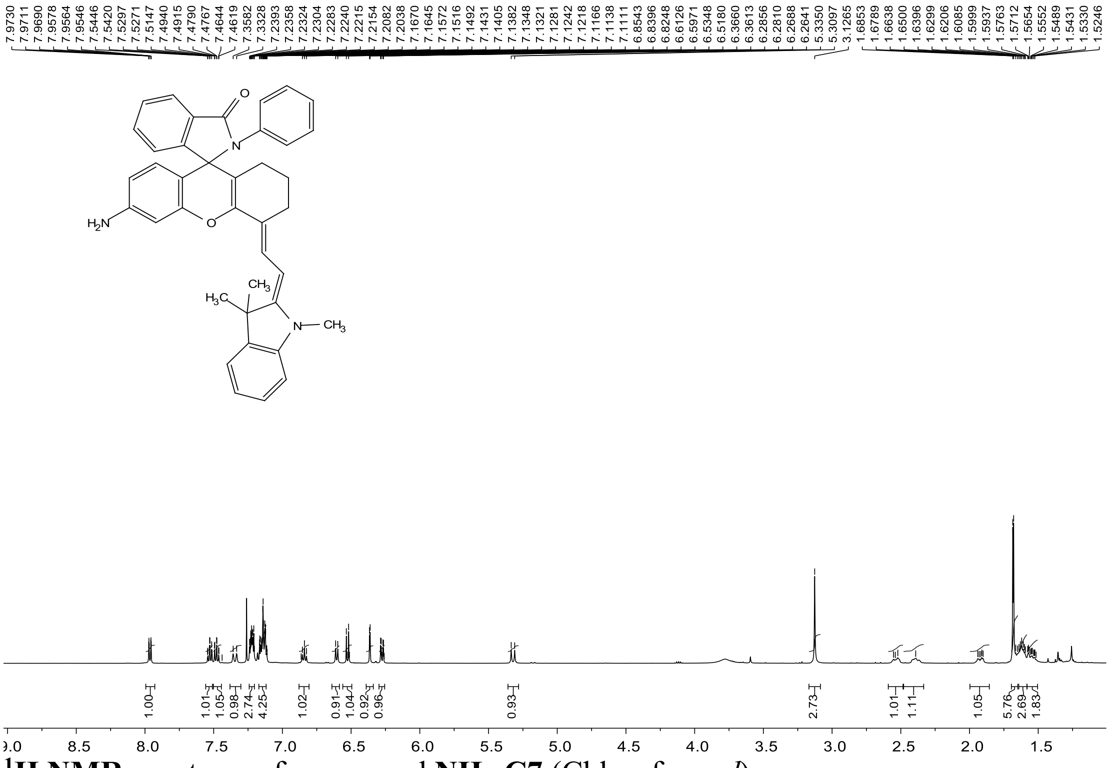

^{1}H NMR spectrum of compound **NH_2-C7** (Chloroform-*d*).

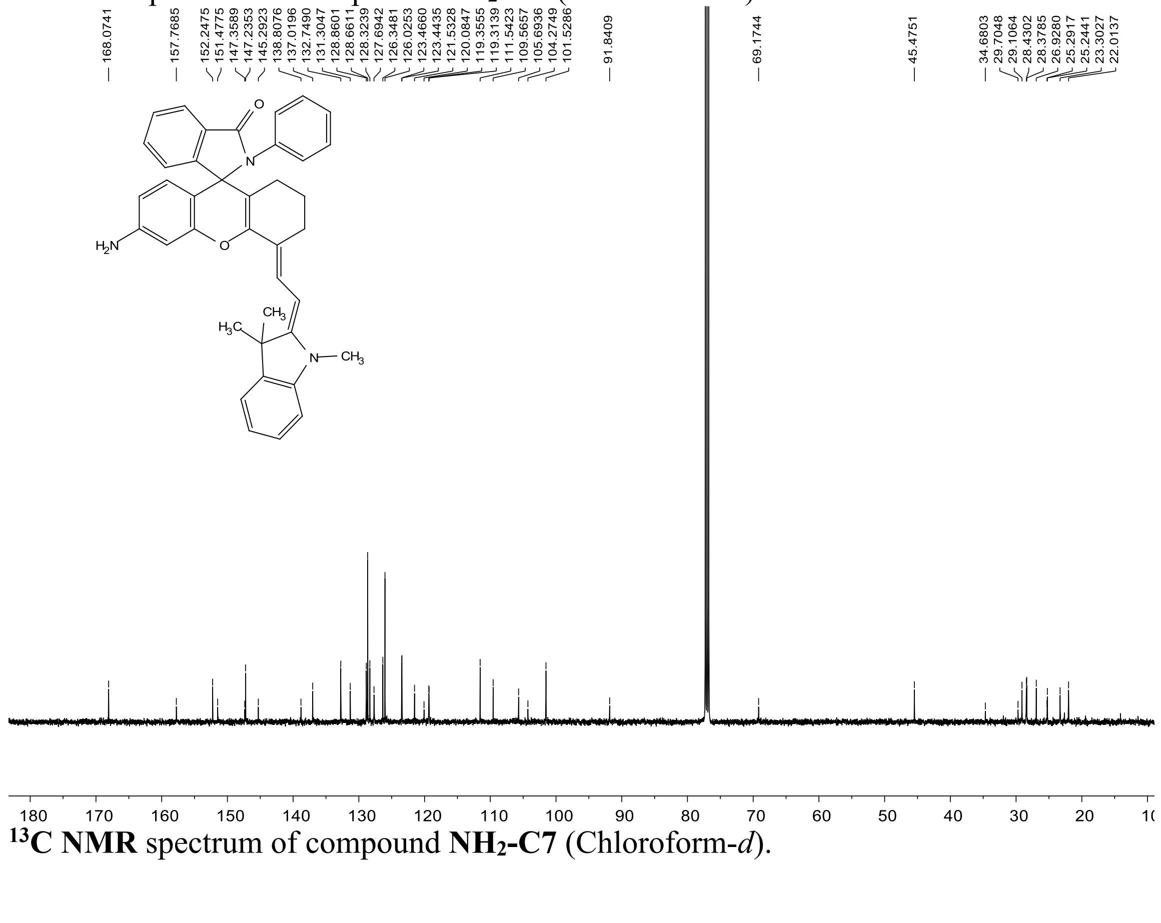

^{13}C NMR spectrum of compound **NH_2-C7** (Chloroform-*d*).

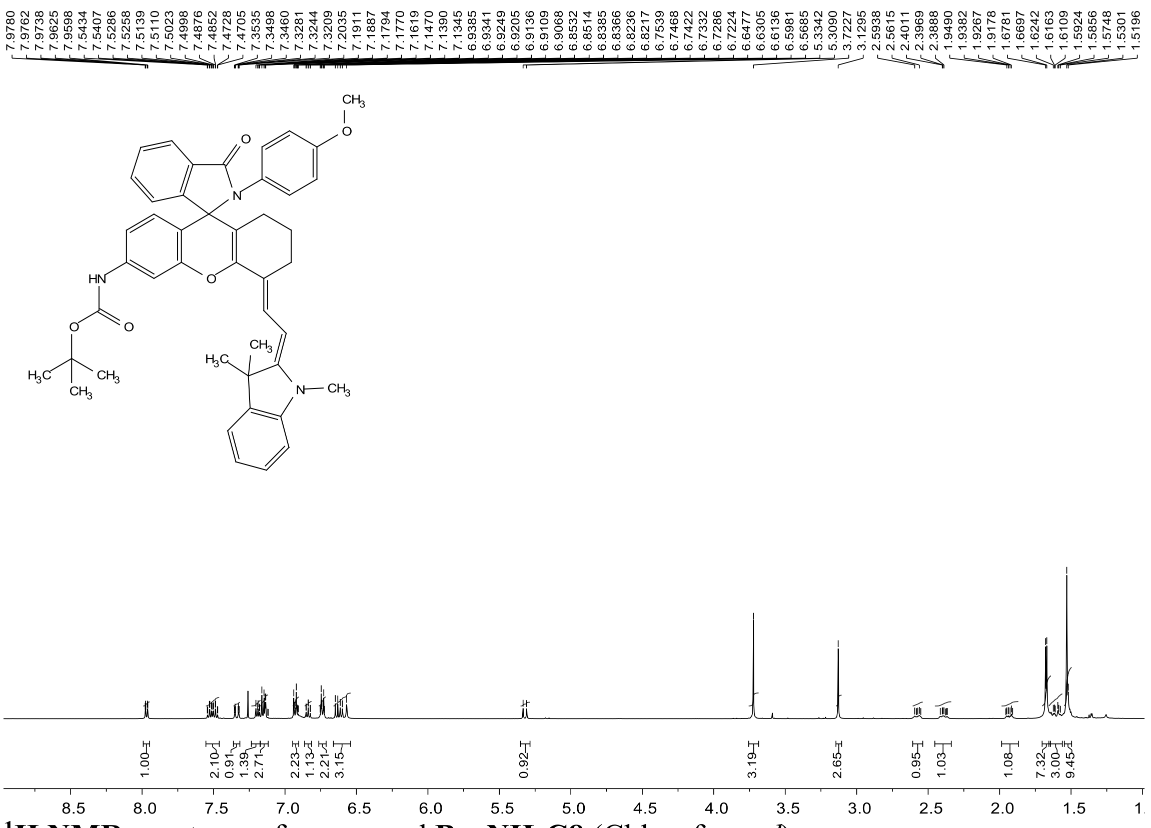

^{1}H NMR spectrum of compound **BocNH-C8** (Chloroform-*d*).

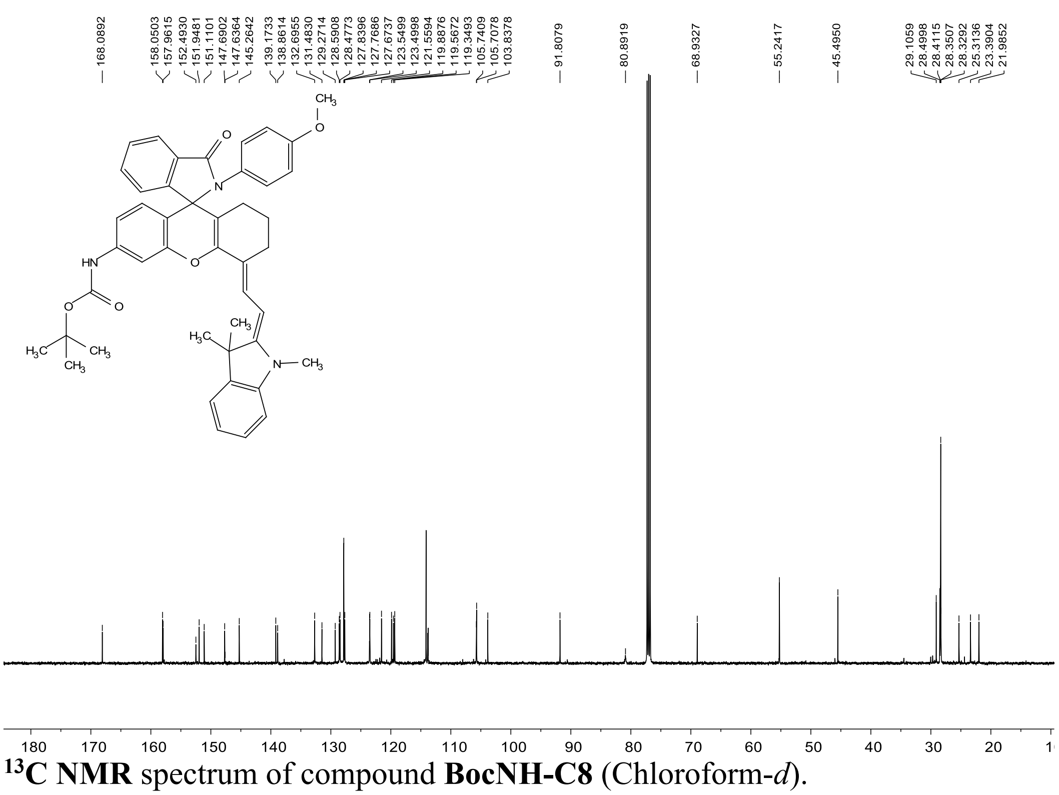

^{13}C NMR spectrum of compound **BocNH-C8** (Chloroform-*d*).

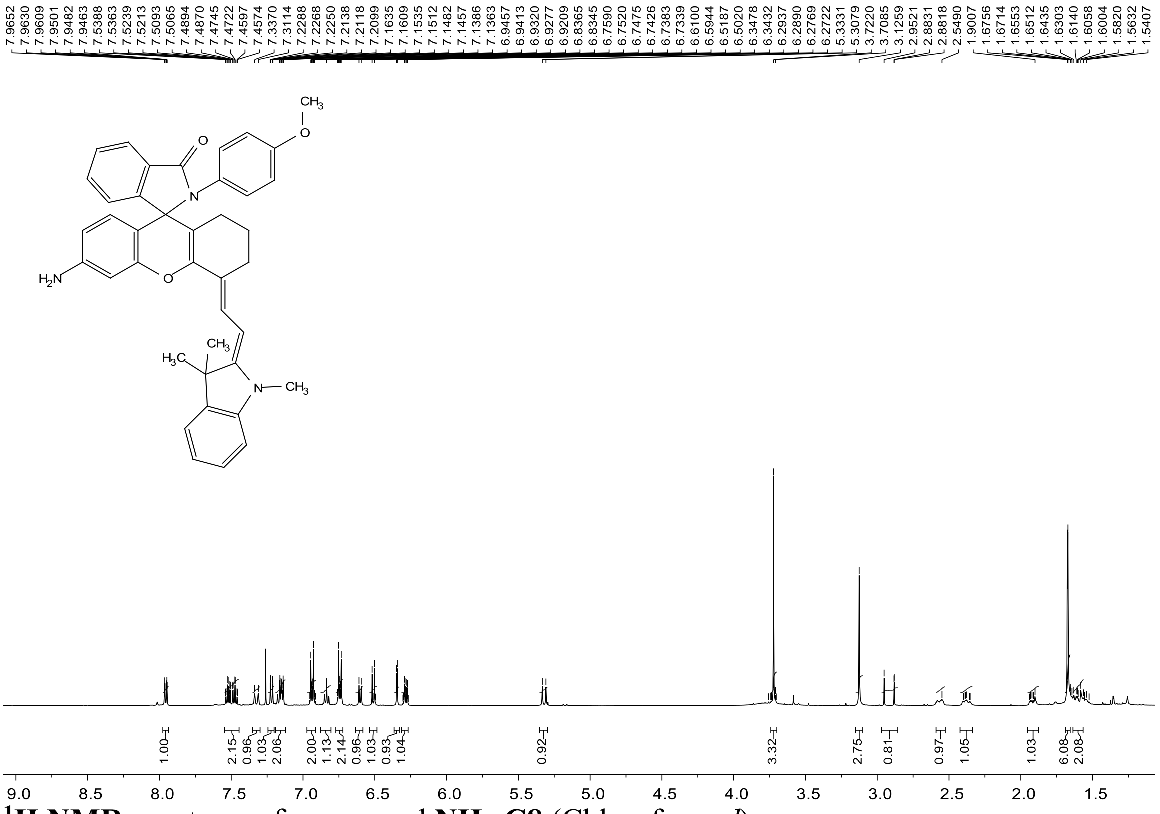

^{1}H NMR spectrum of compound **NH_2-C8** (Chloroform-*d*).

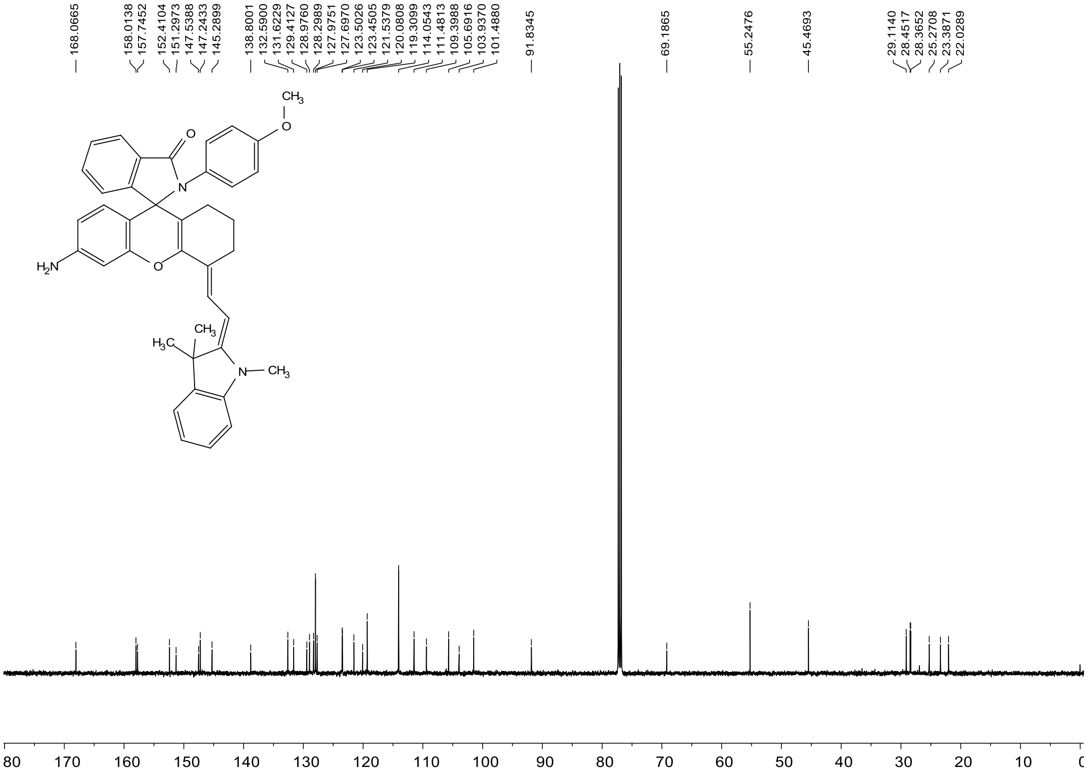

^{13}C NMR spectrum of compound **NH_2-C8** (Chloroform-*d*).

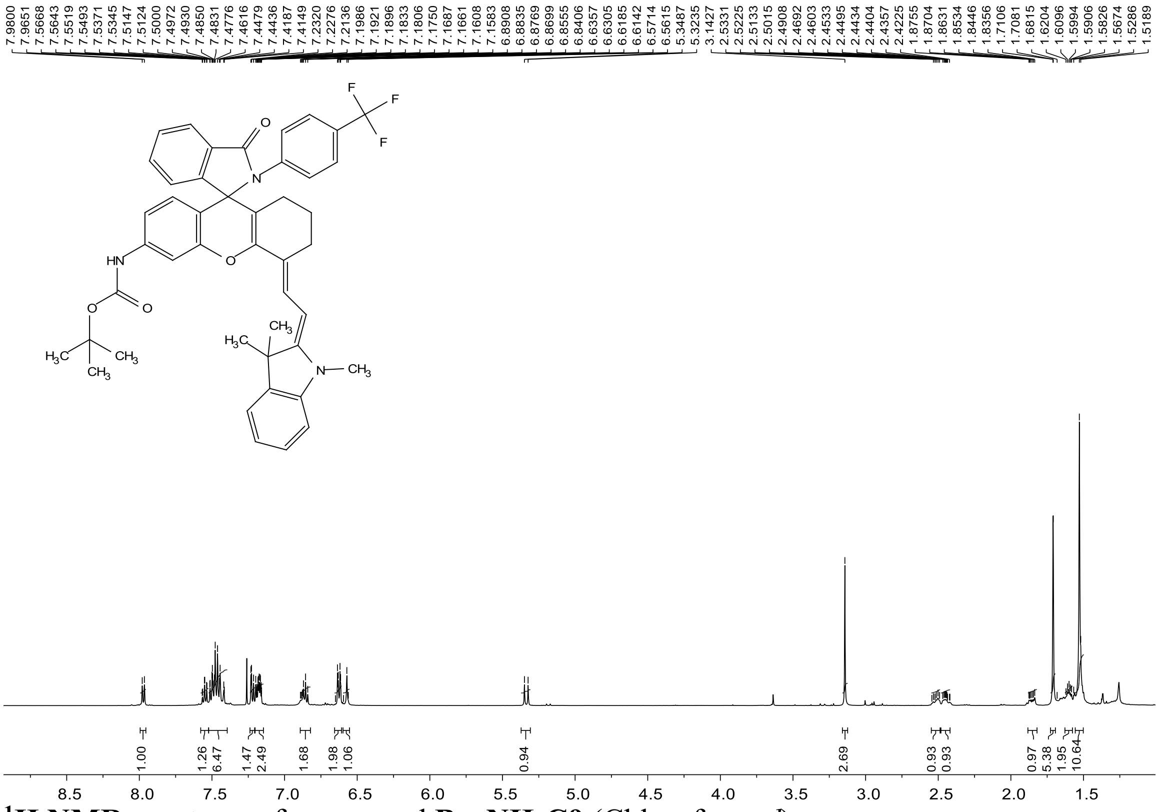

^{1}H NMR spectrum of compound **BocNH-C9** (Chloroform-*d*).

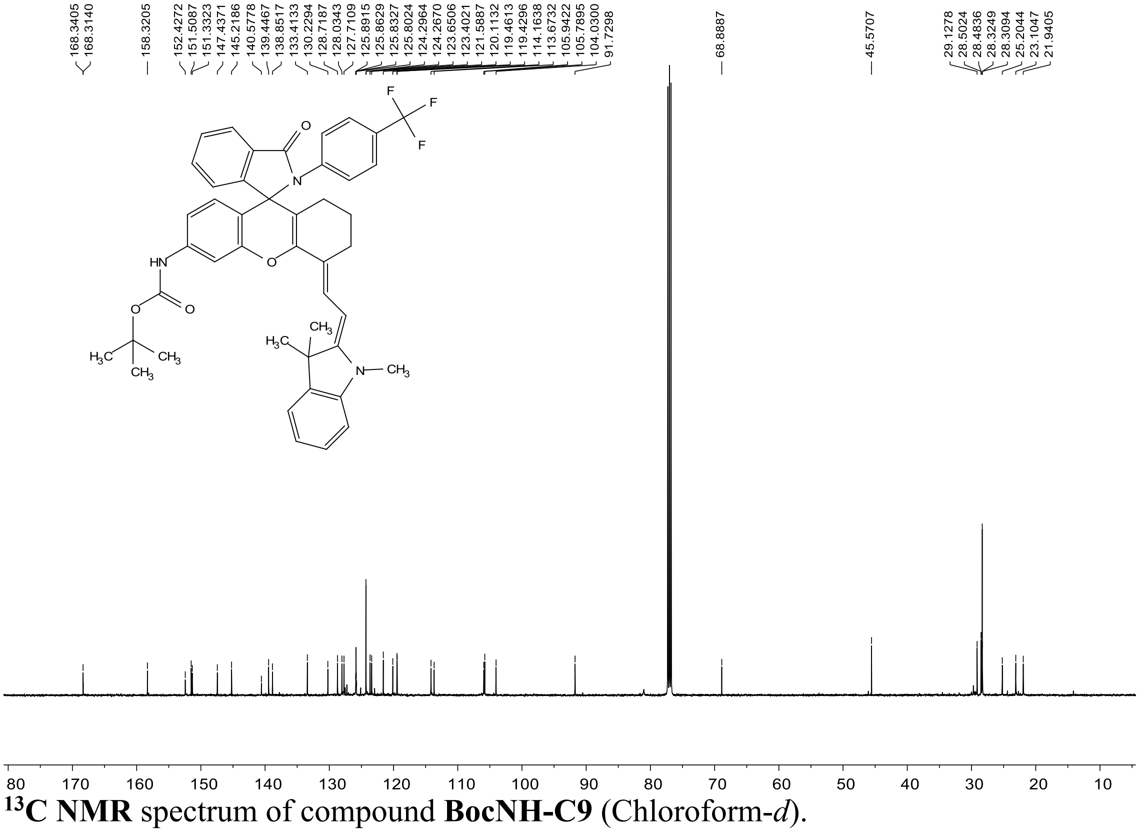

^{13}C NMR spectrum of compound **BocNH-C9** (Chloroform-*d*).

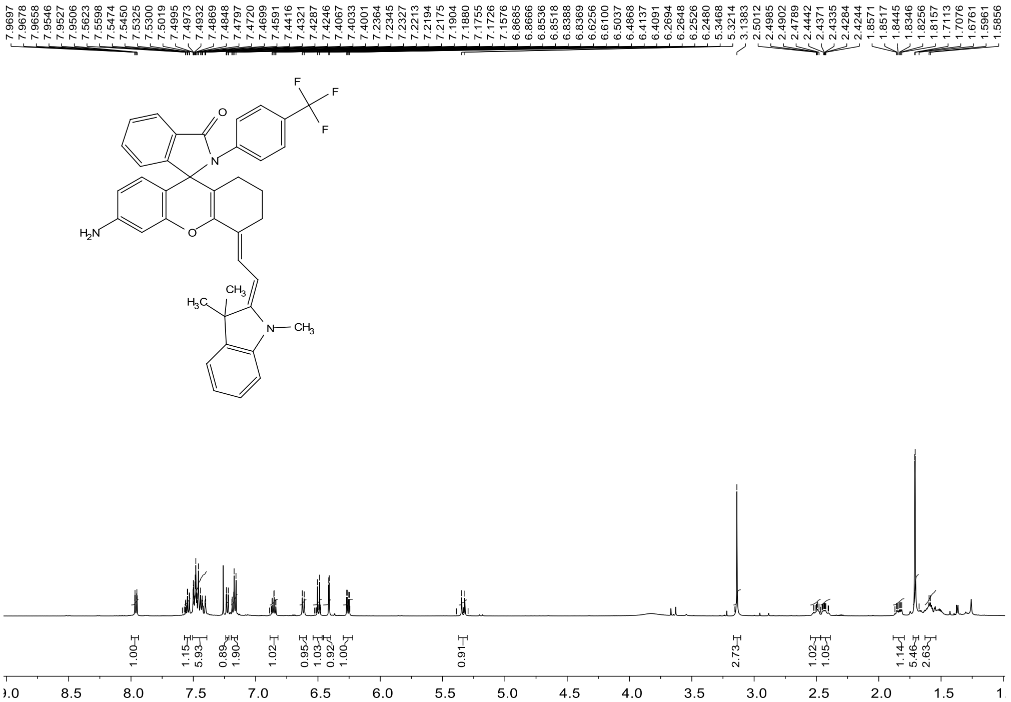

^{1}H NMR spectrum of compound **NH_2-C9** (Chloroform-*d*).

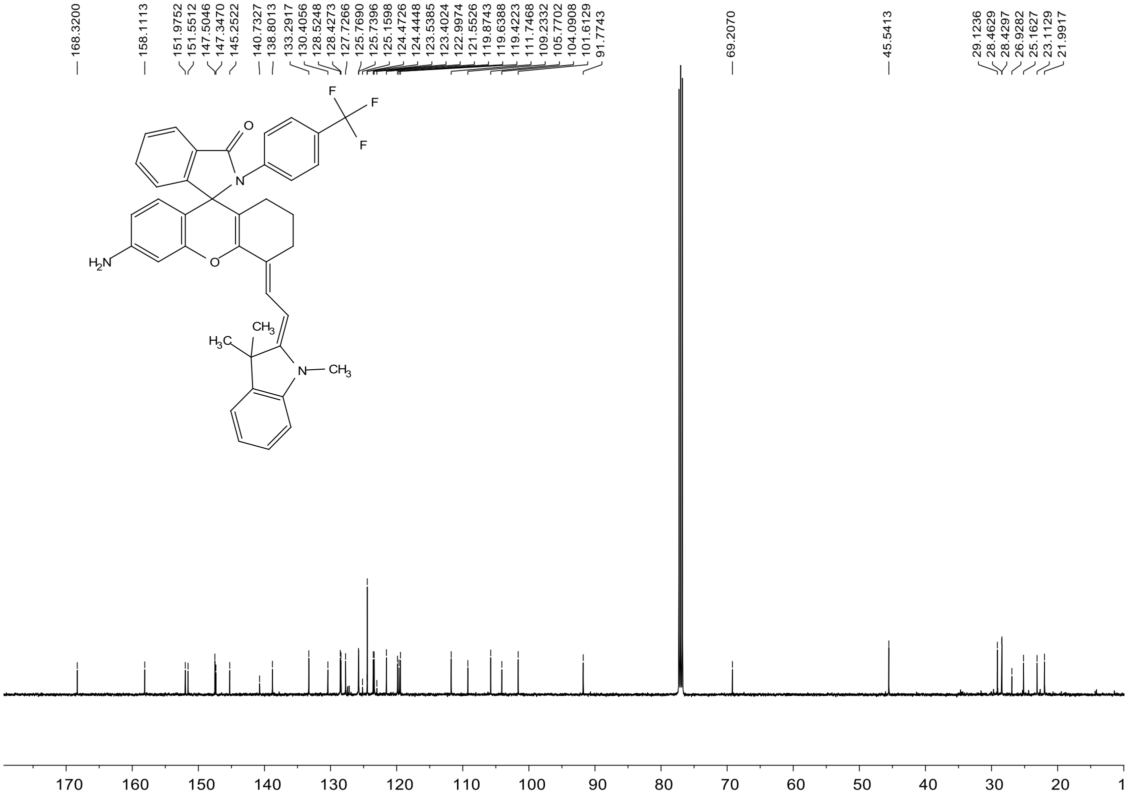

^{13}C NMR spectrum of compound **NH_2-C9** (Chloroform-*d*).

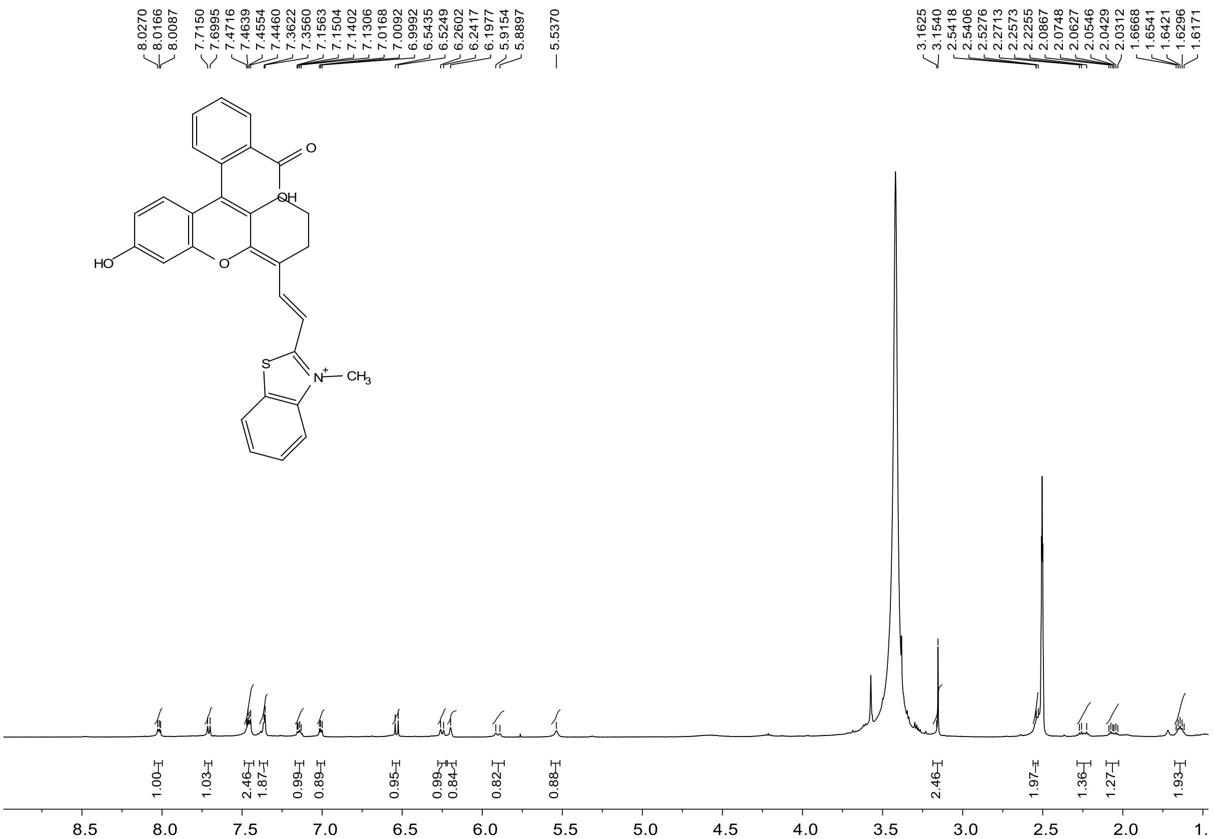

^{1}H NMR spectrum of compound **D1** (DMSO-d_6).

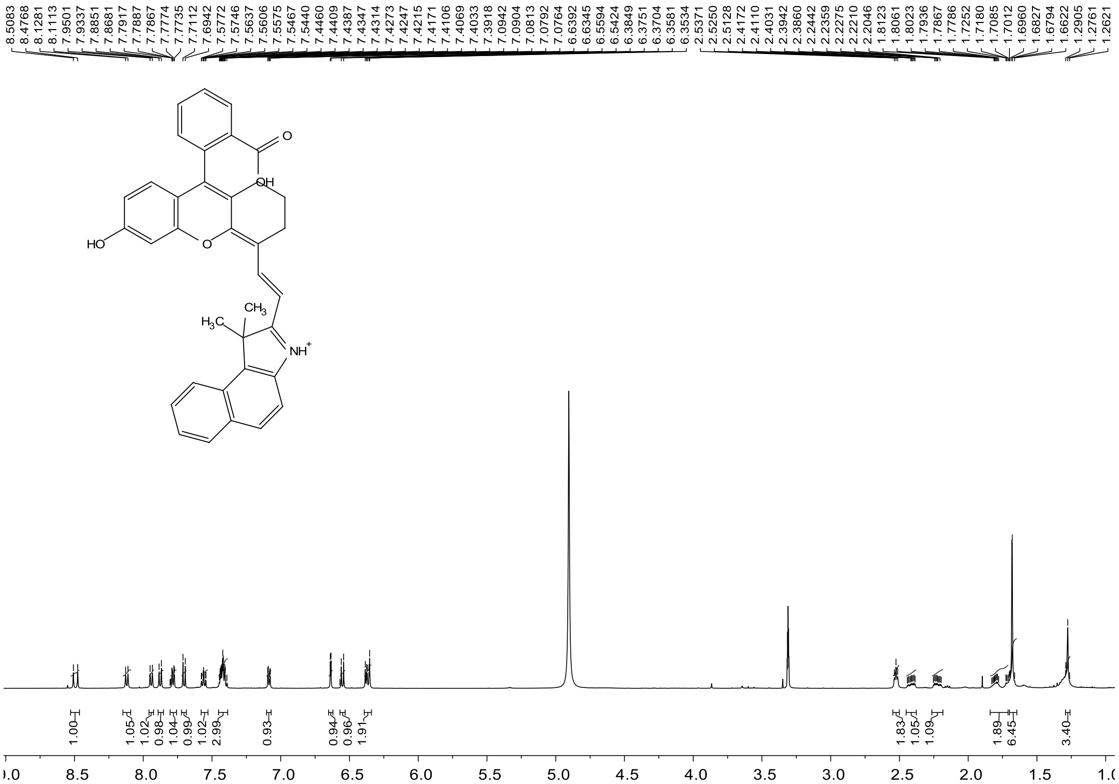

^{1}H NMR spectrum of compound **D3** (Methanol-d_4).

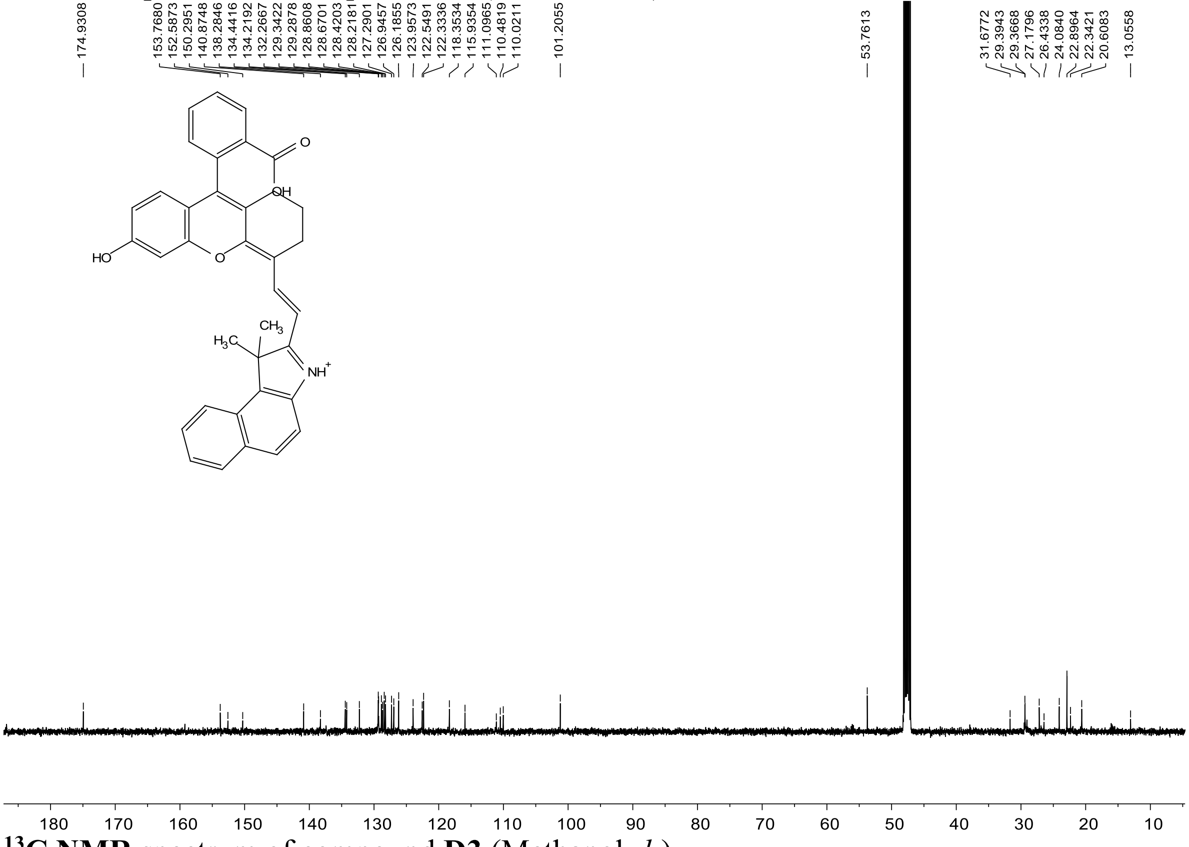

^{13}C NMR spectrum of compound **D3** (Methanol-d_4)

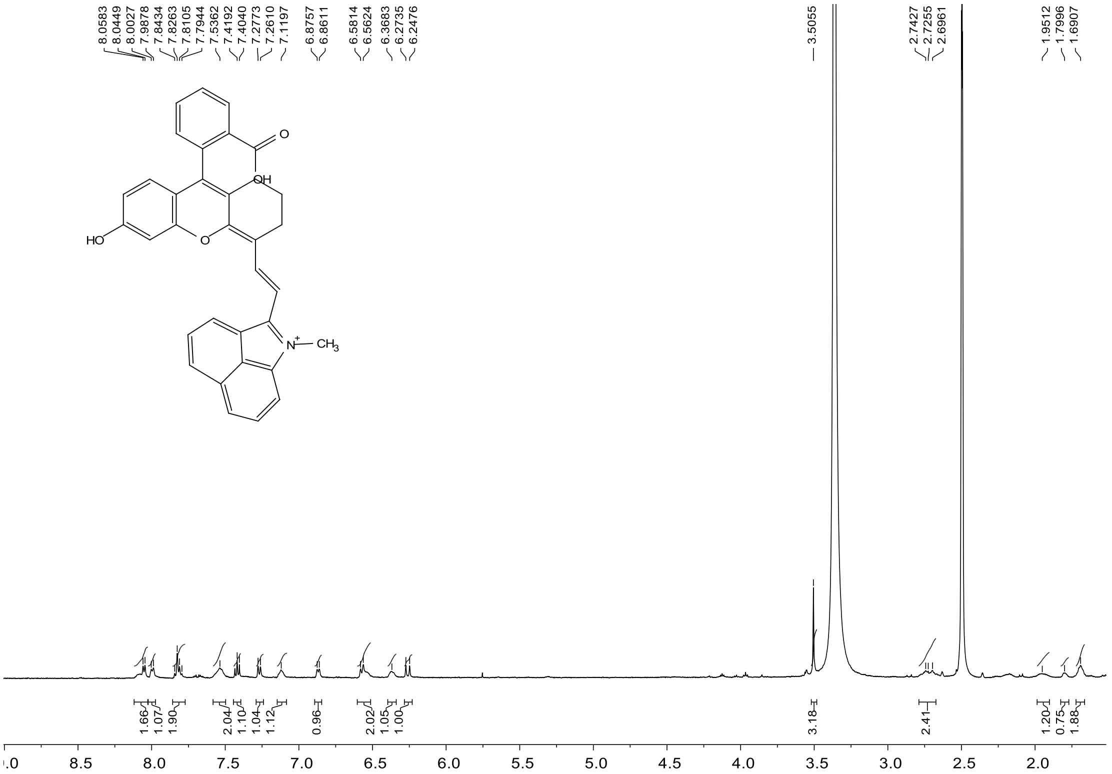

^{1}H NMR spectrum of compound **D4** (DMSO-d_6).

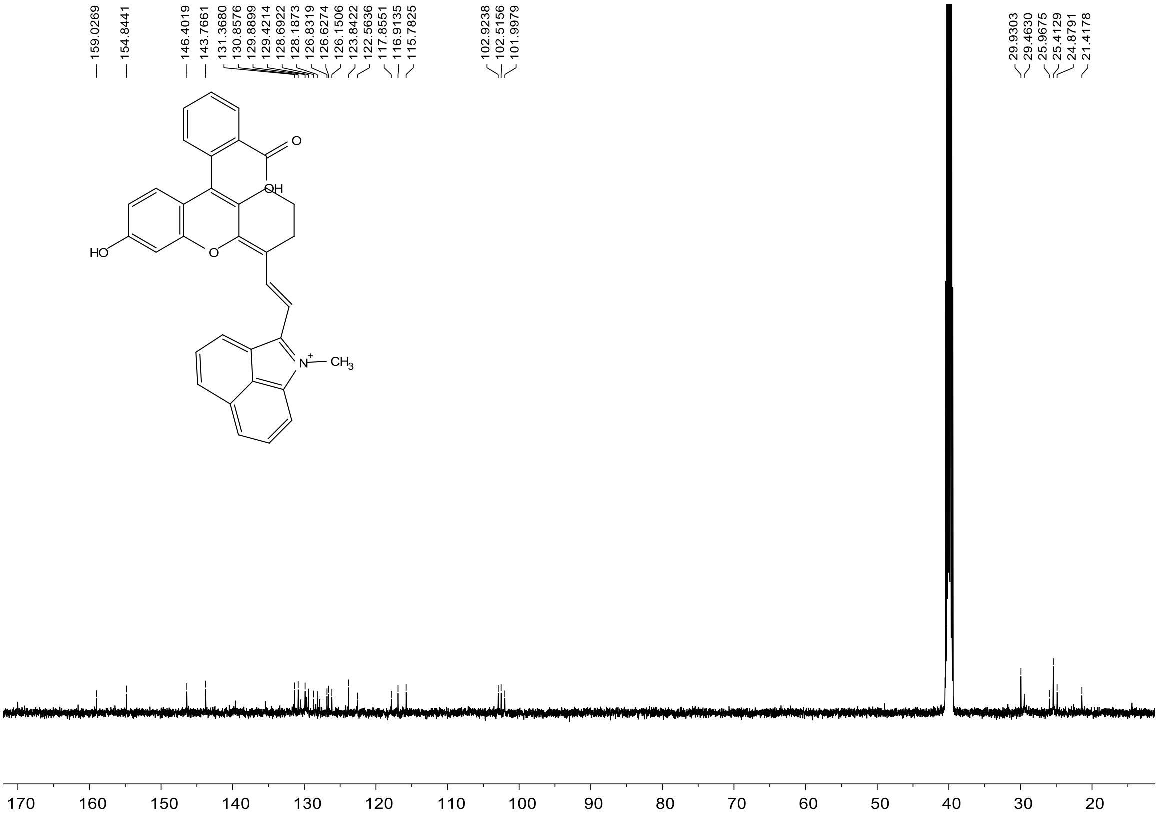

^{13}C NMR spectrum of compound **D4** (DMSO-d_6).

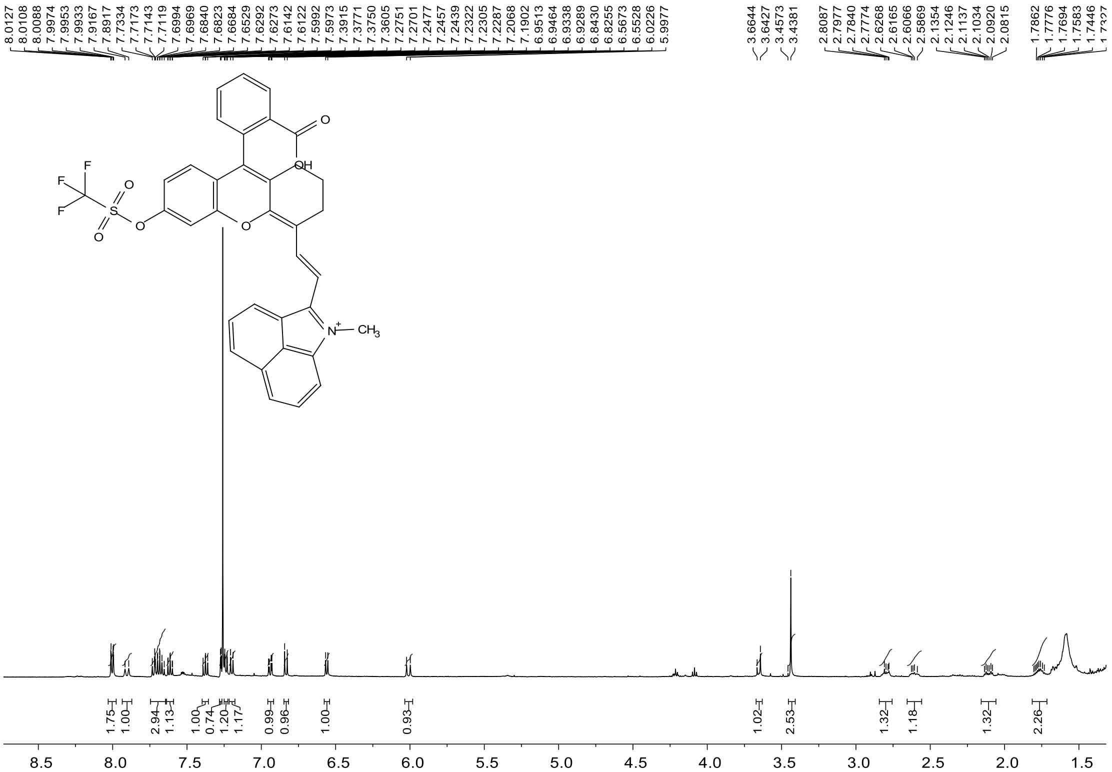

^{1}H NMR spectrum of compound **D4-a** (Chloroform-*d*).

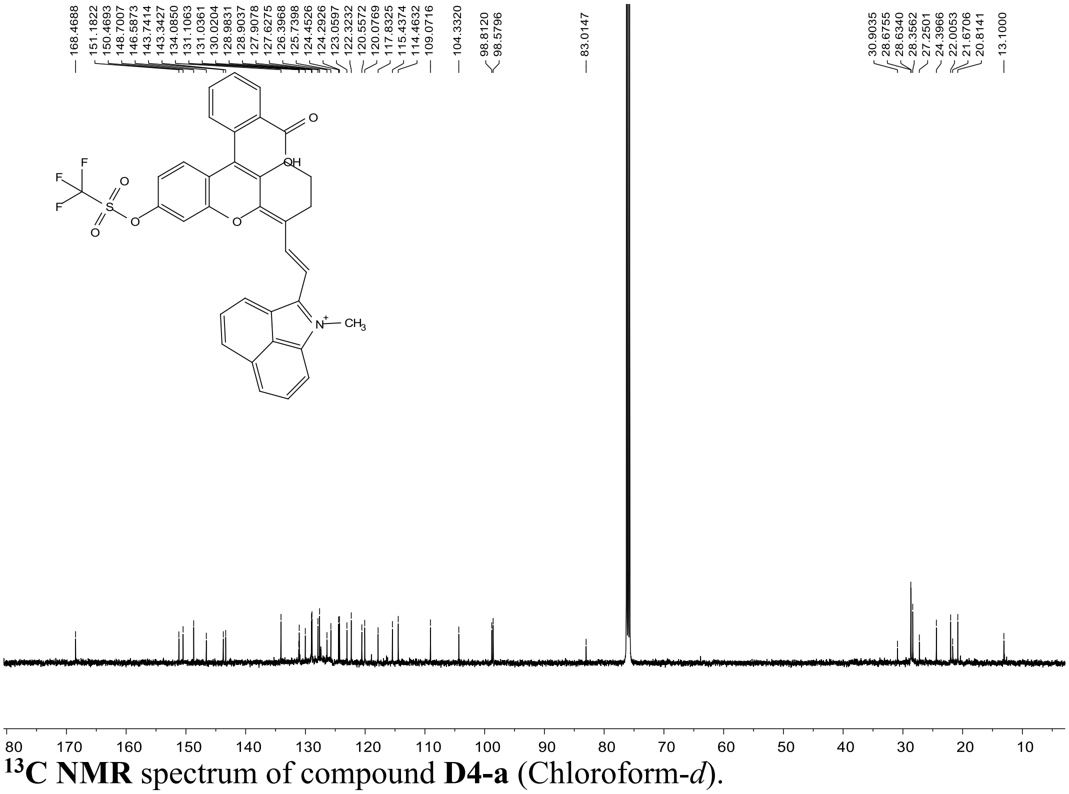

^{13}C NMR spectrum of compound **D4-a** (Chloroform-*d*).

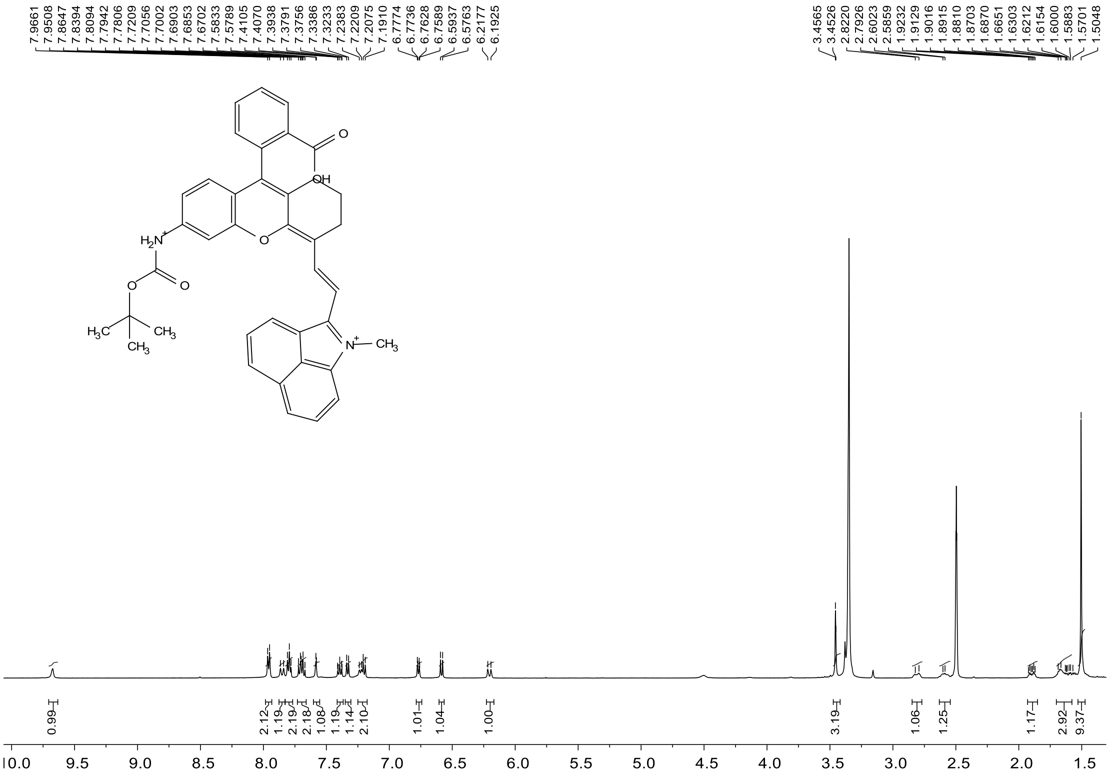

^{1}**H NMR** spectrum of compound **D4-b** (DMSO-d_6).

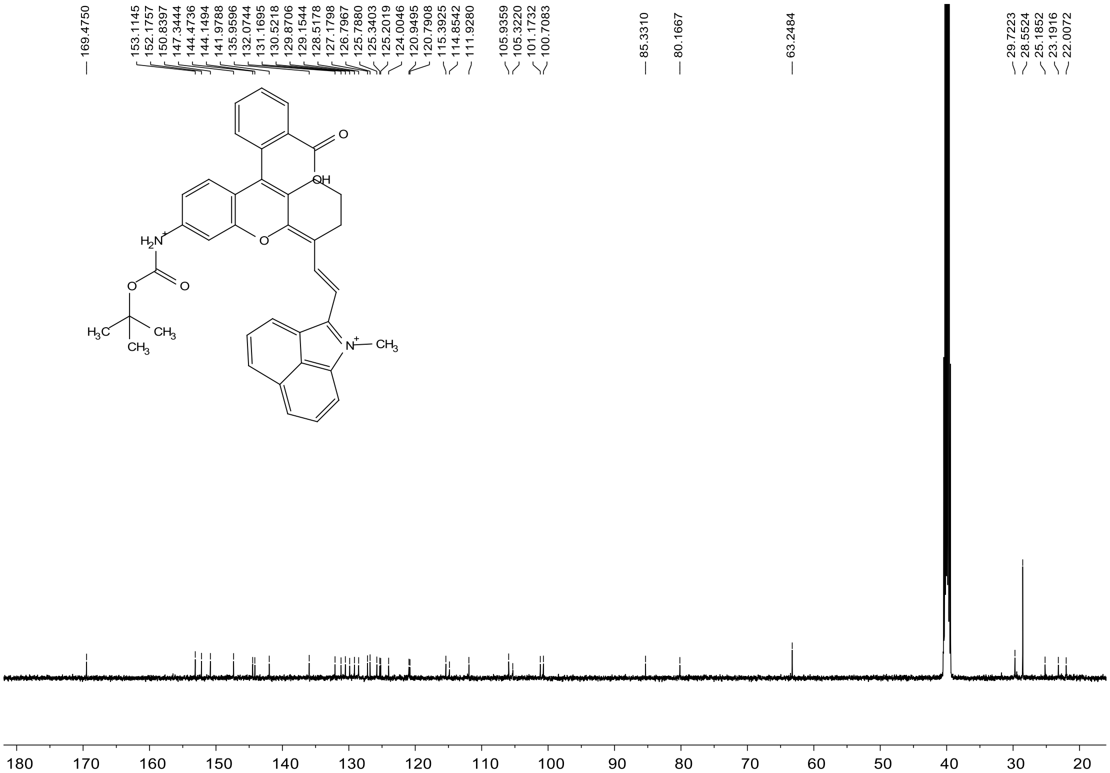

^{13}**C NMR** spectrum of compound **D4-b** (DMSO-d_6).

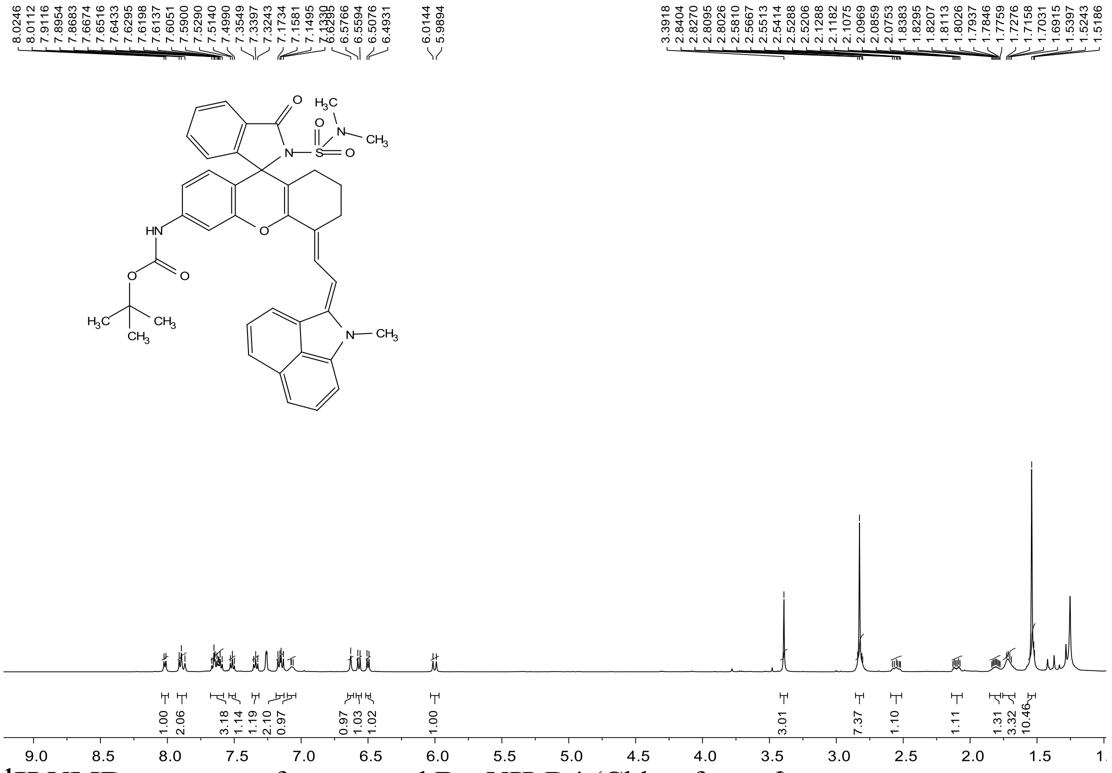

¹H NMR spectrum of compound **BocNH-D4** (Chloroform-*d*).

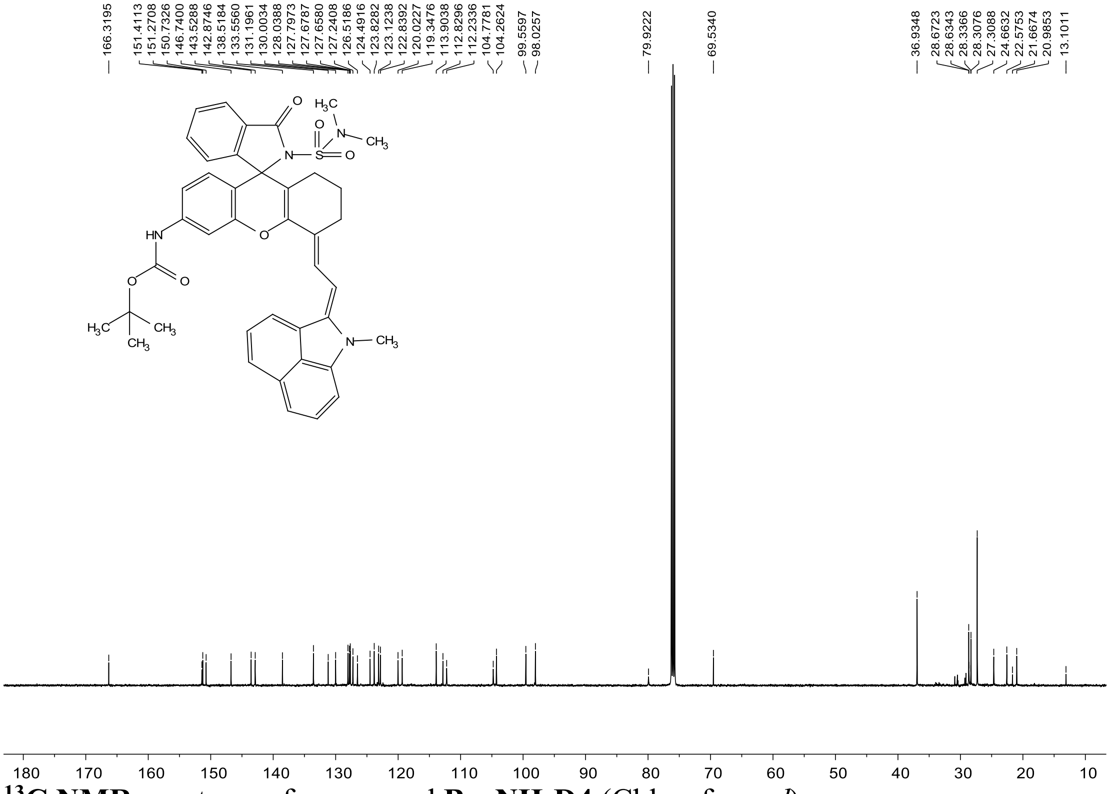

¹³C NMR spectrum of compound **BocNH-D4** (Chloroform-*d*).

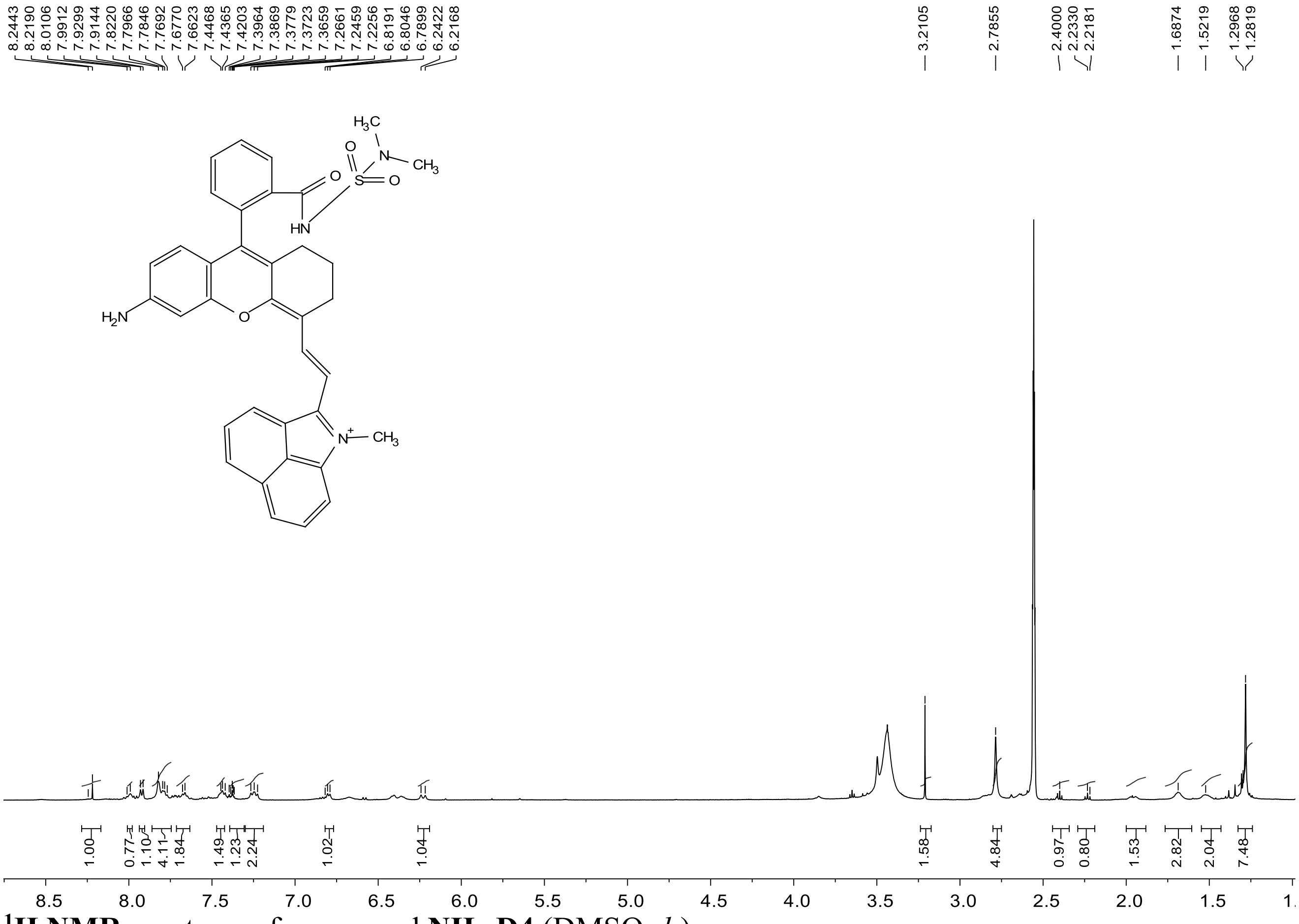

^{1}H NMR spectrum of compound **NH_2-D4** (DMSO-d_6).

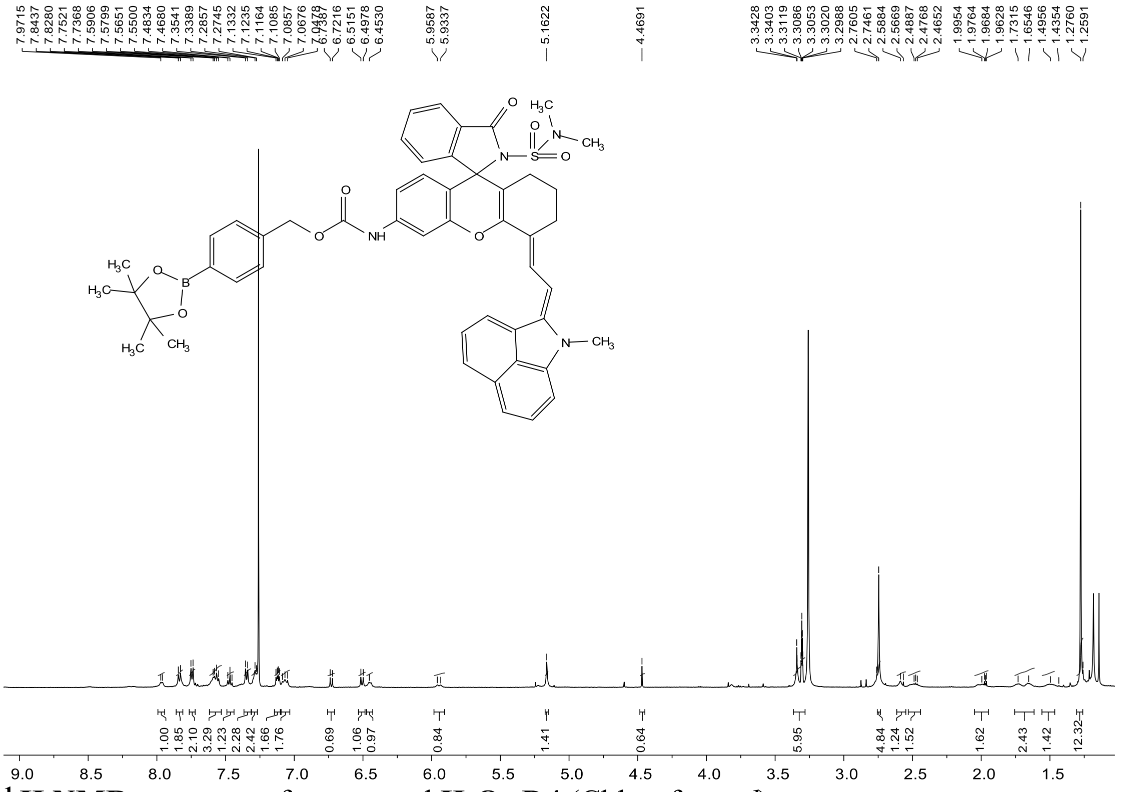

^{1}**H NMR** spectrum of compound **H_2O_2-D4** (Chloroform-*d*).

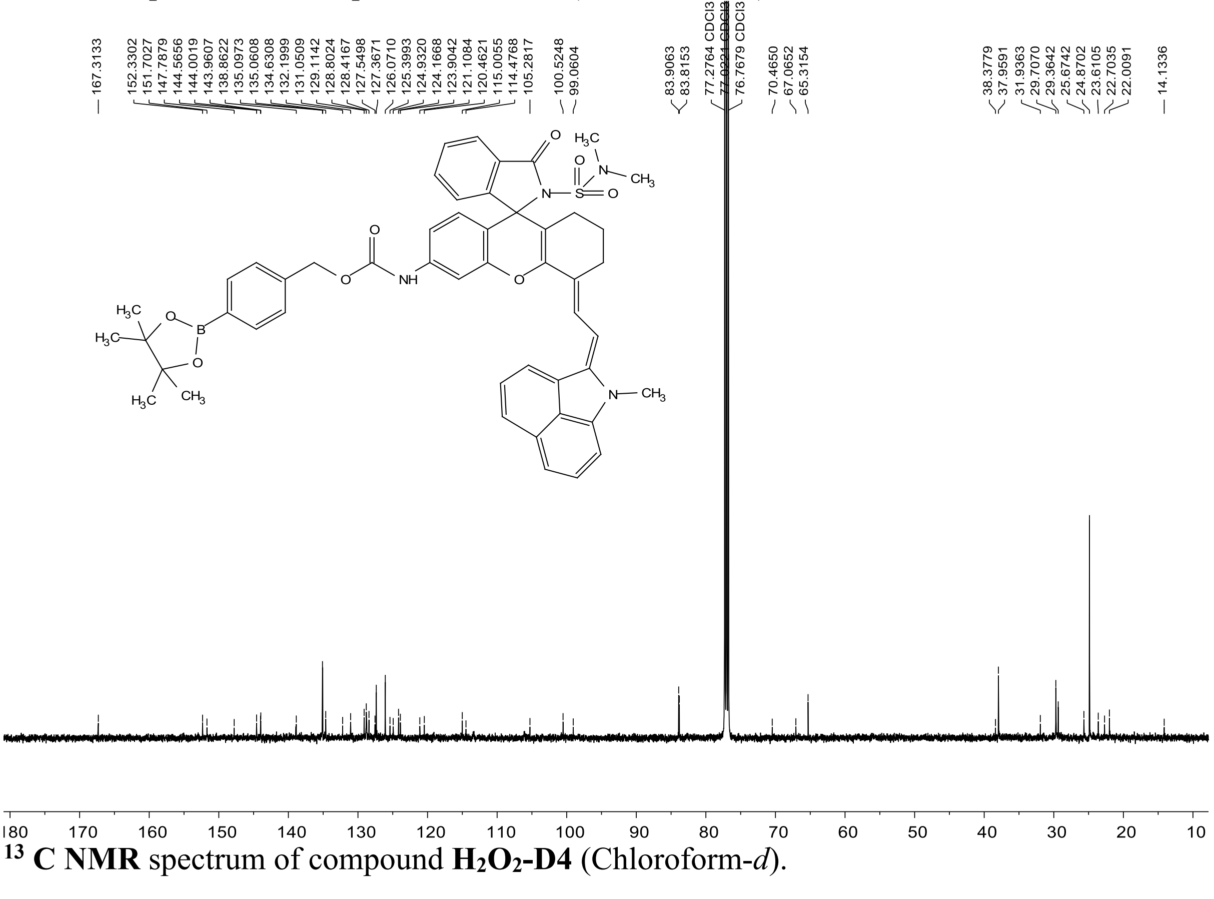

^{13}**C NMR** spectrum of compound **H_2O_2-D4** (Chloroform-*d*).

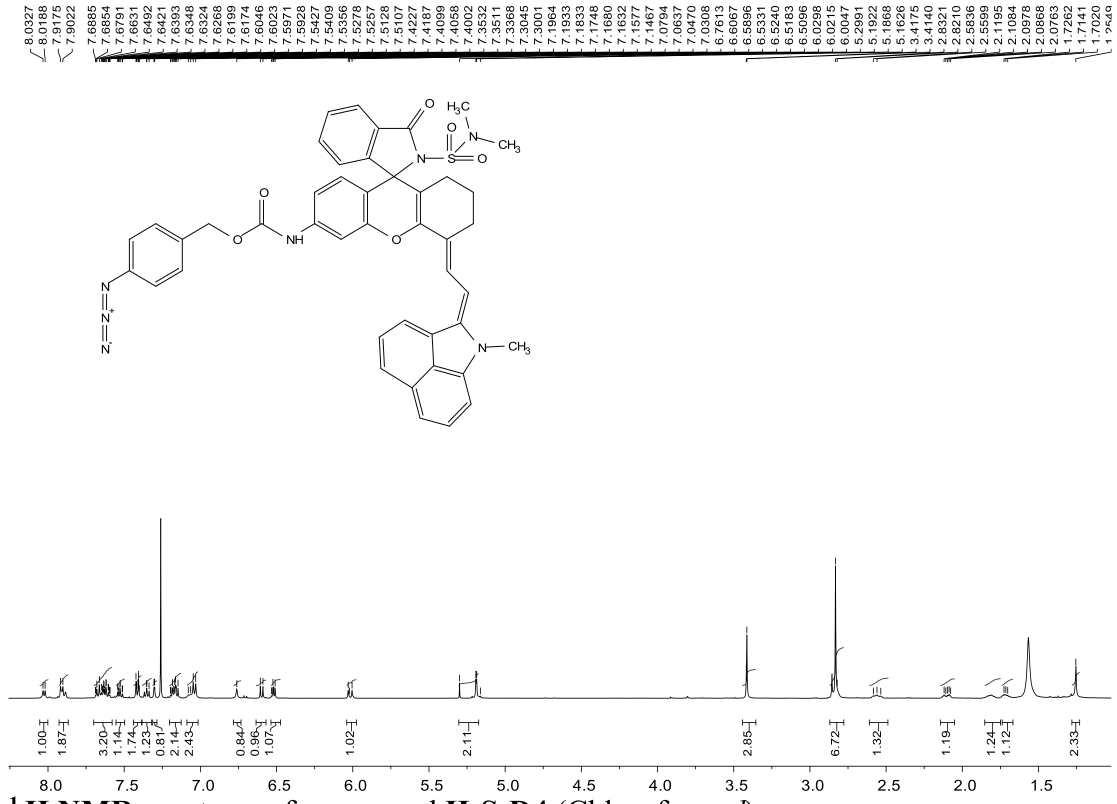

[1] H NMR spectrum of compound **H$_2$S-D4** (Chloroform-*d*).

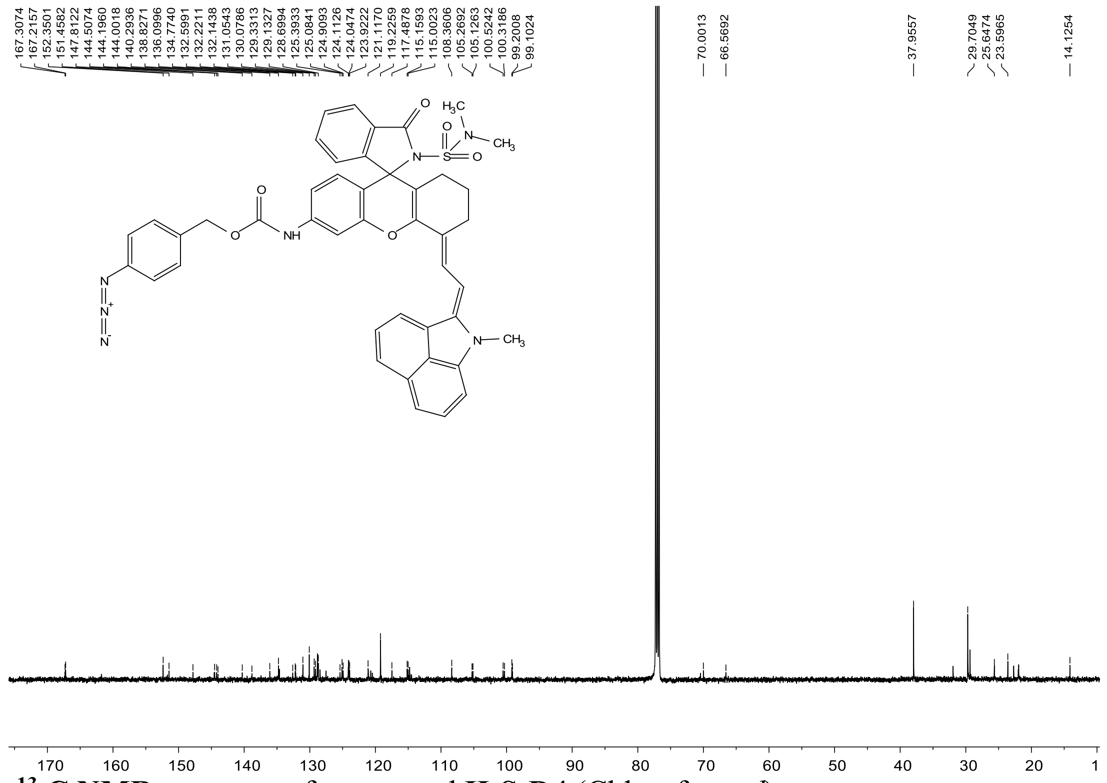

[13] C NMR spectrum of compound **H$_2$S-D4** (Chloroform-*d*).

8. HRMS spectrum traces of compounds

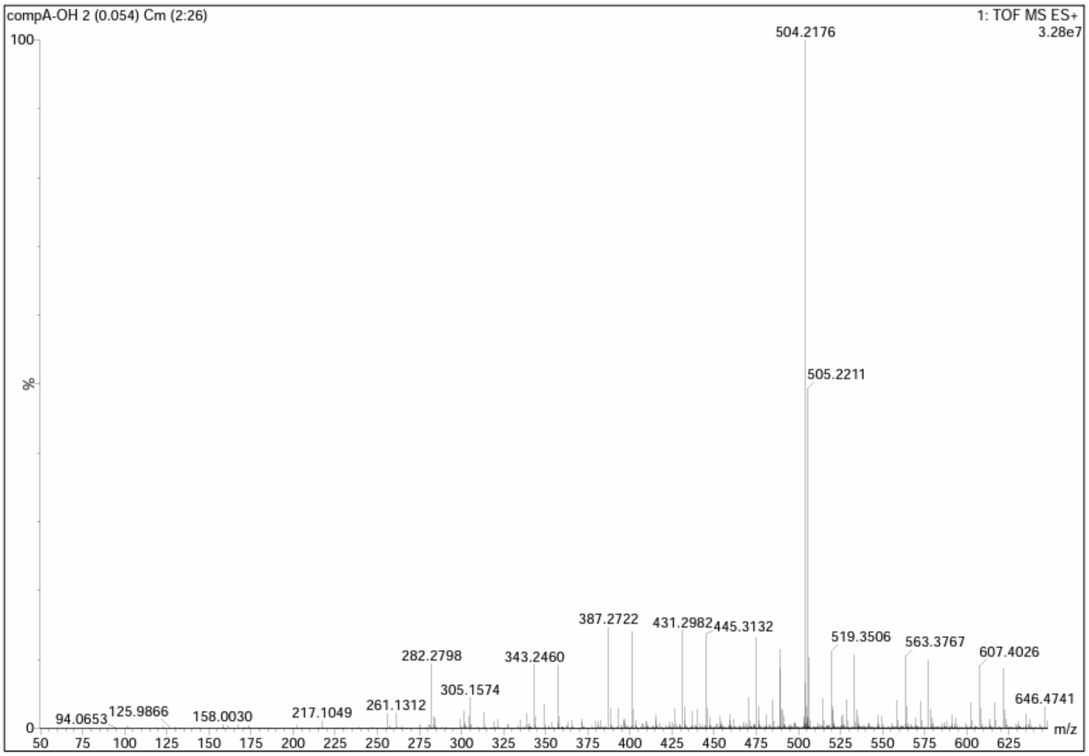

HRMS spectrum of compound **D2**

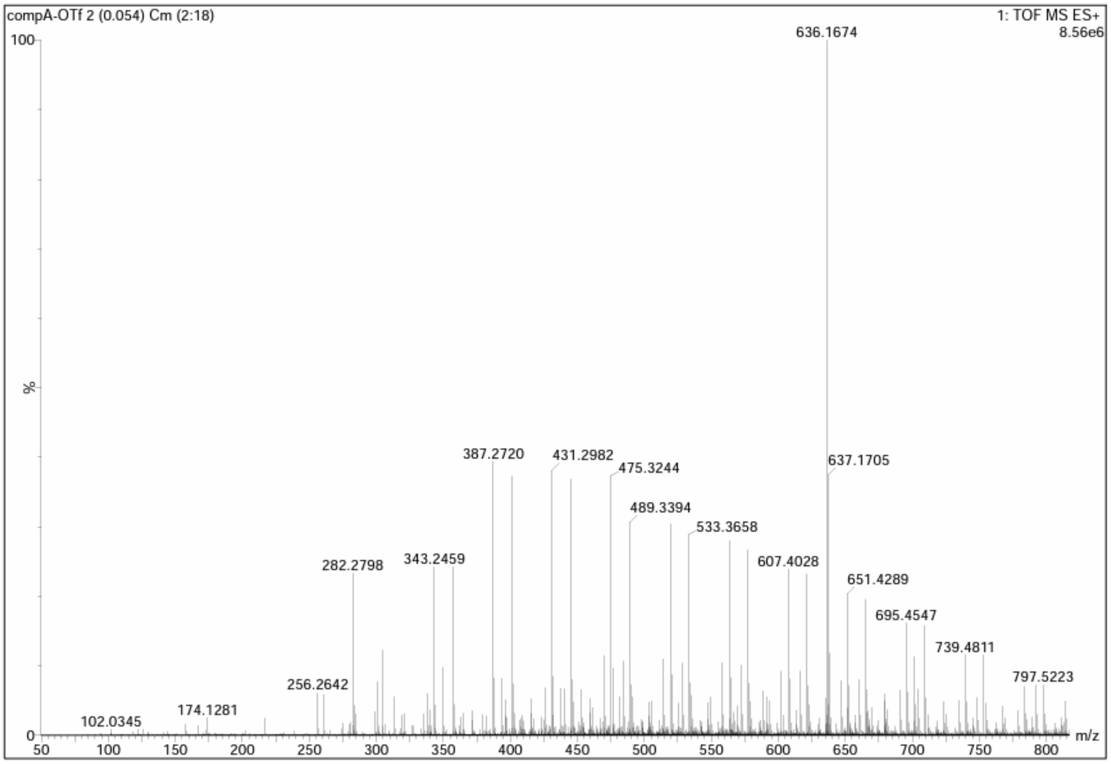

HRMS spectrum of compound **D2-OTf**

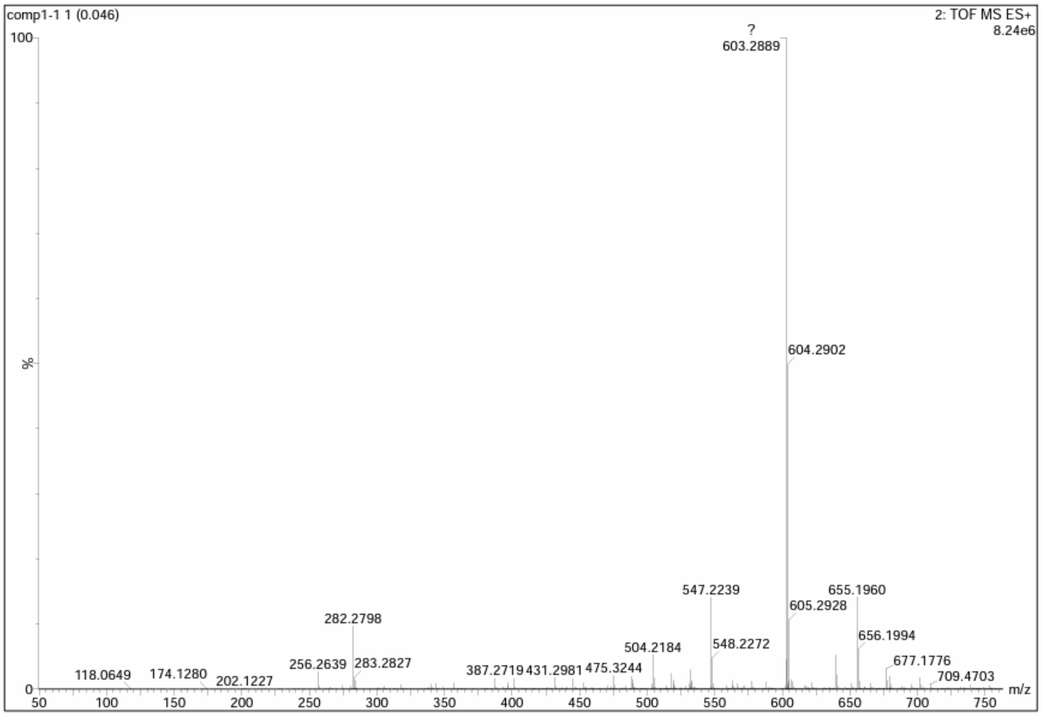

HRMS spectrum of compound **BocNH-C1**

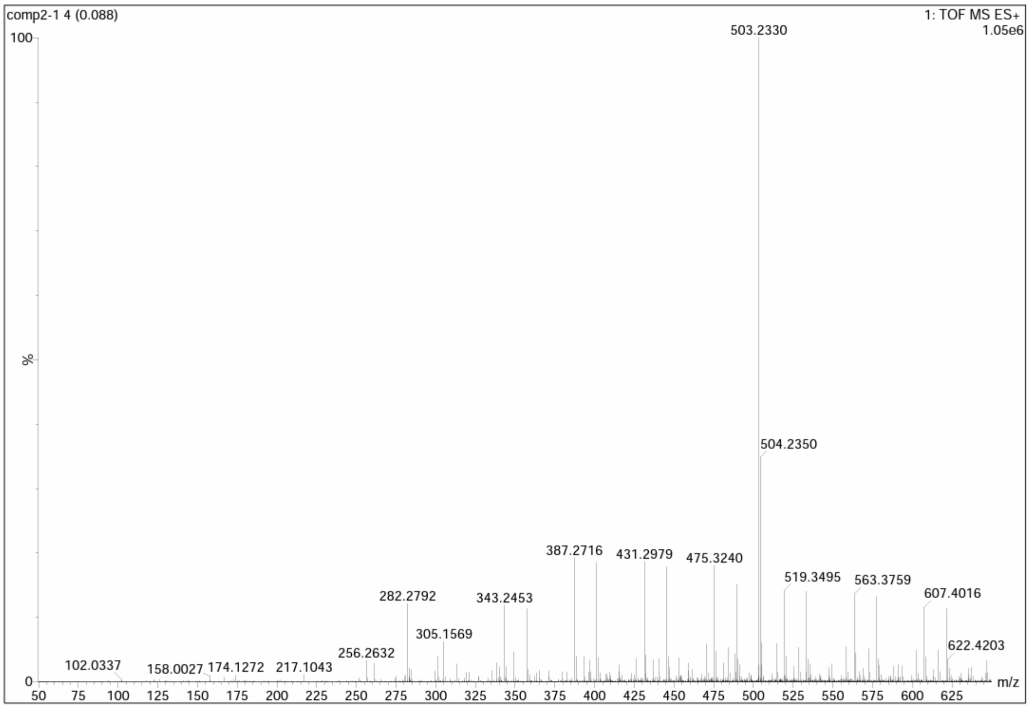

HRMS spectrum of compound **NH_2-C1**

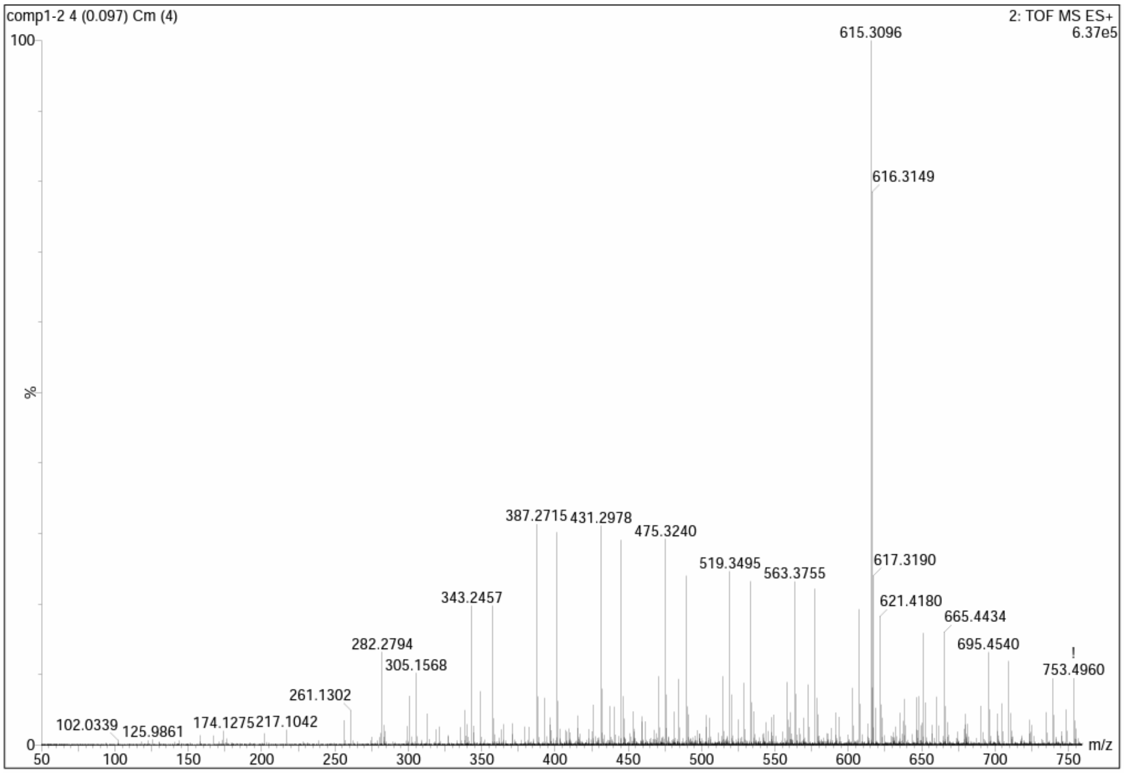

HRMS spectrum of compound **BocNH-C2**

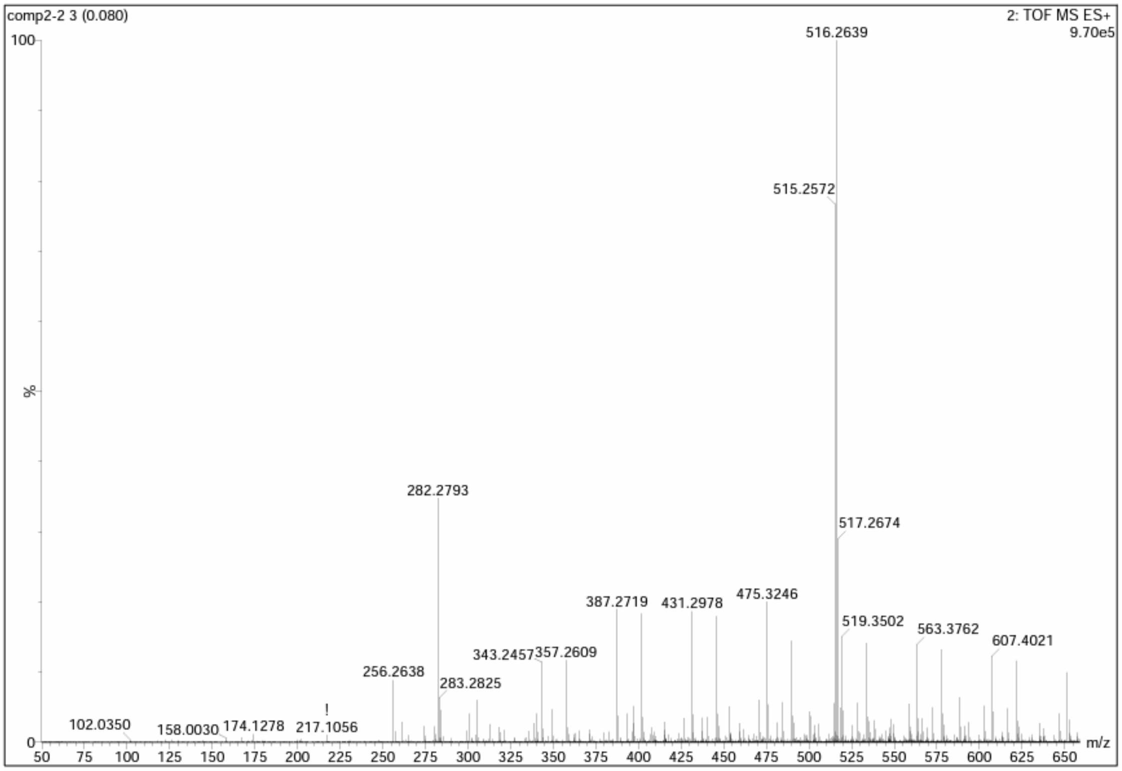

HRMS spectrum of compound **NH_2-C2**

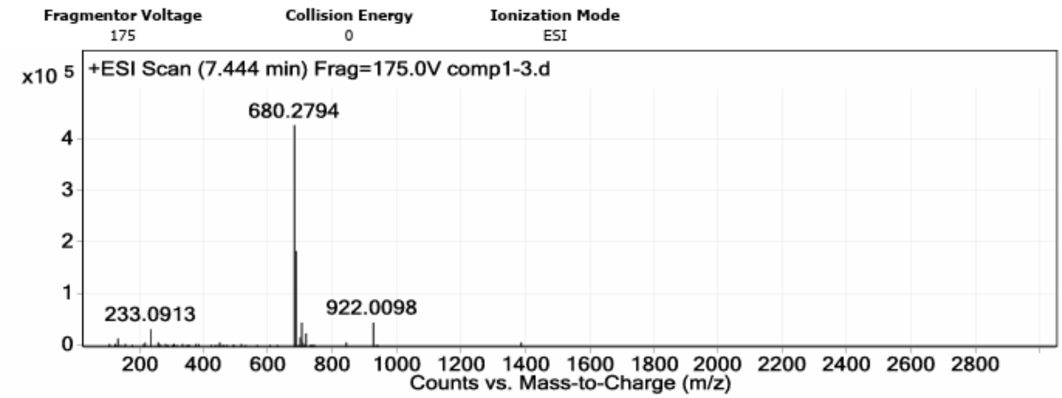

HRMS spectrum of compound **BocNH-C3**

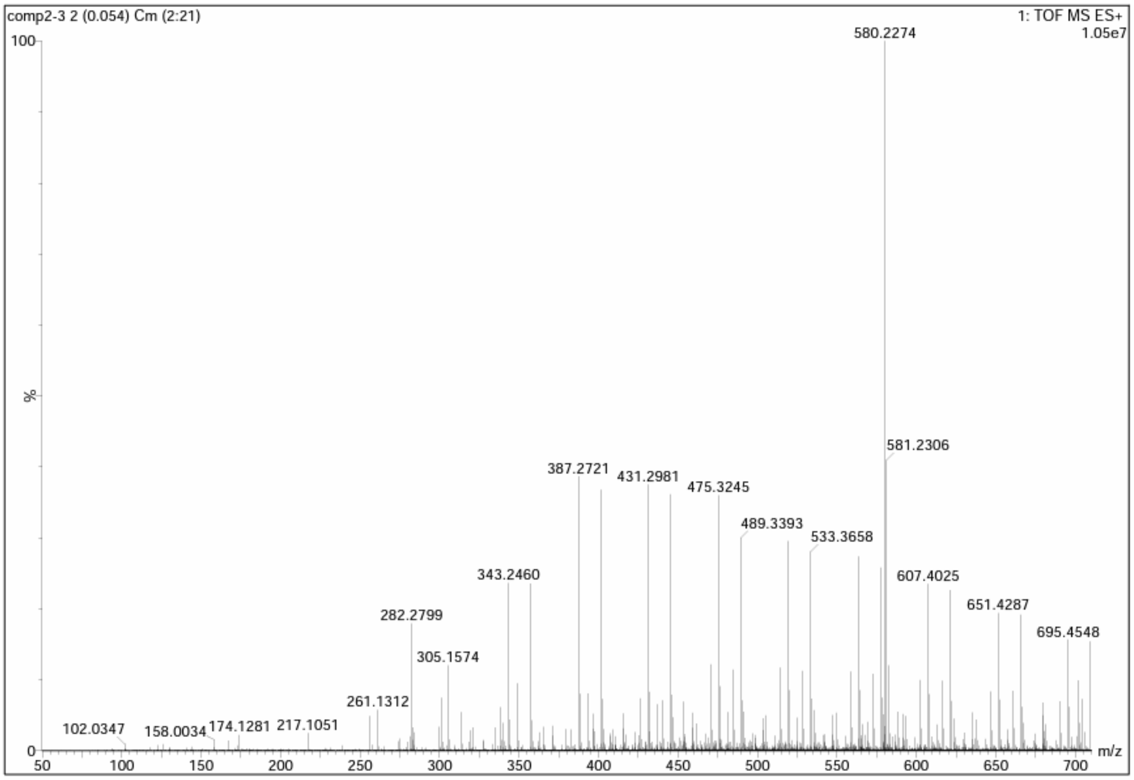

HRMS spectrum of compound **NH_2-C3**

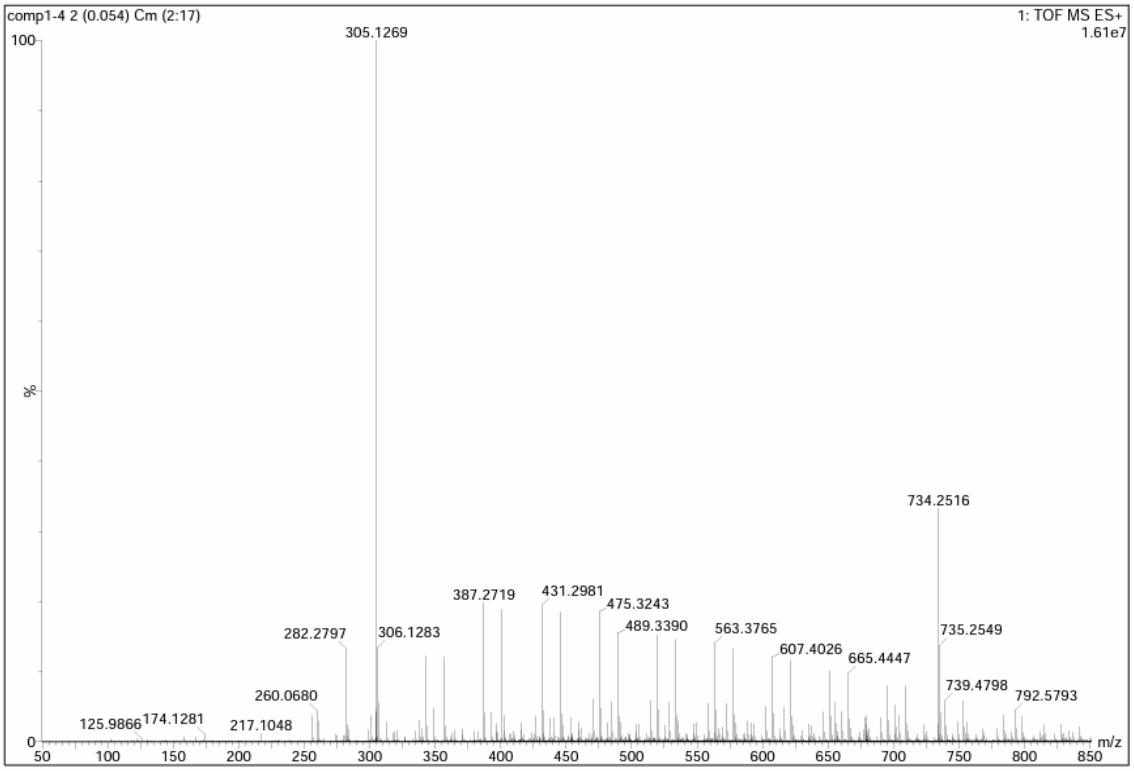

HRMS spectrum of compound **BocNH-C4**

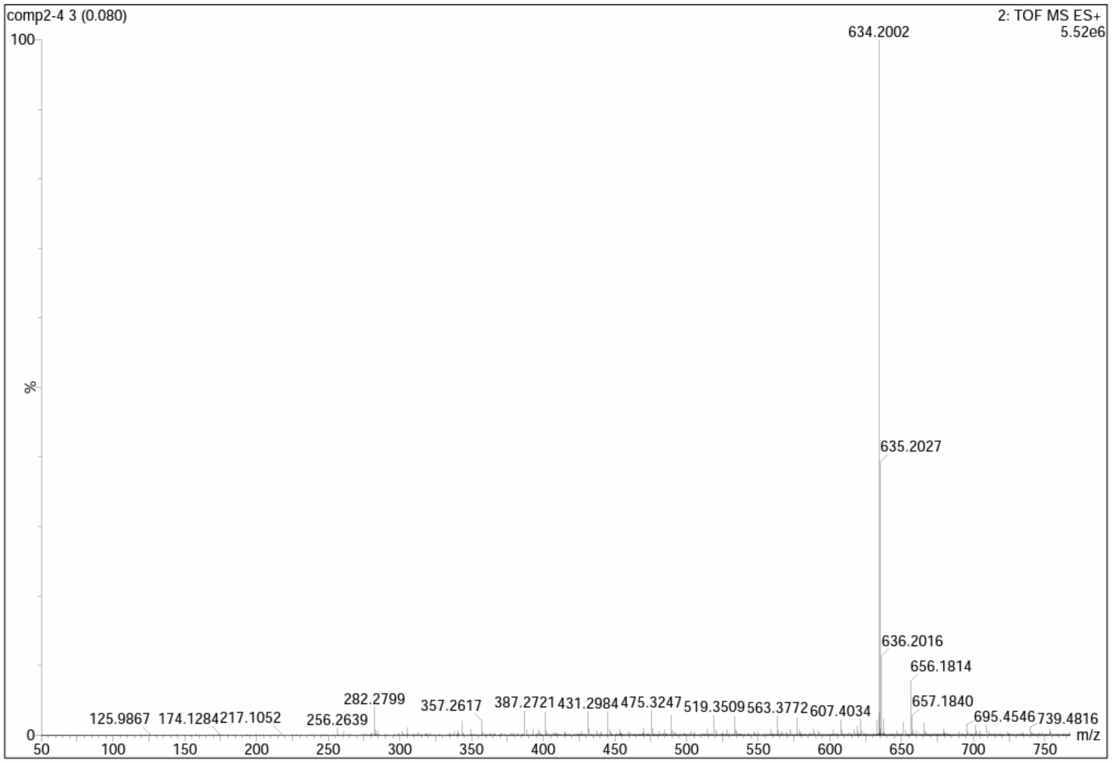

HRMS spectrum of compound **NH_2-C4**

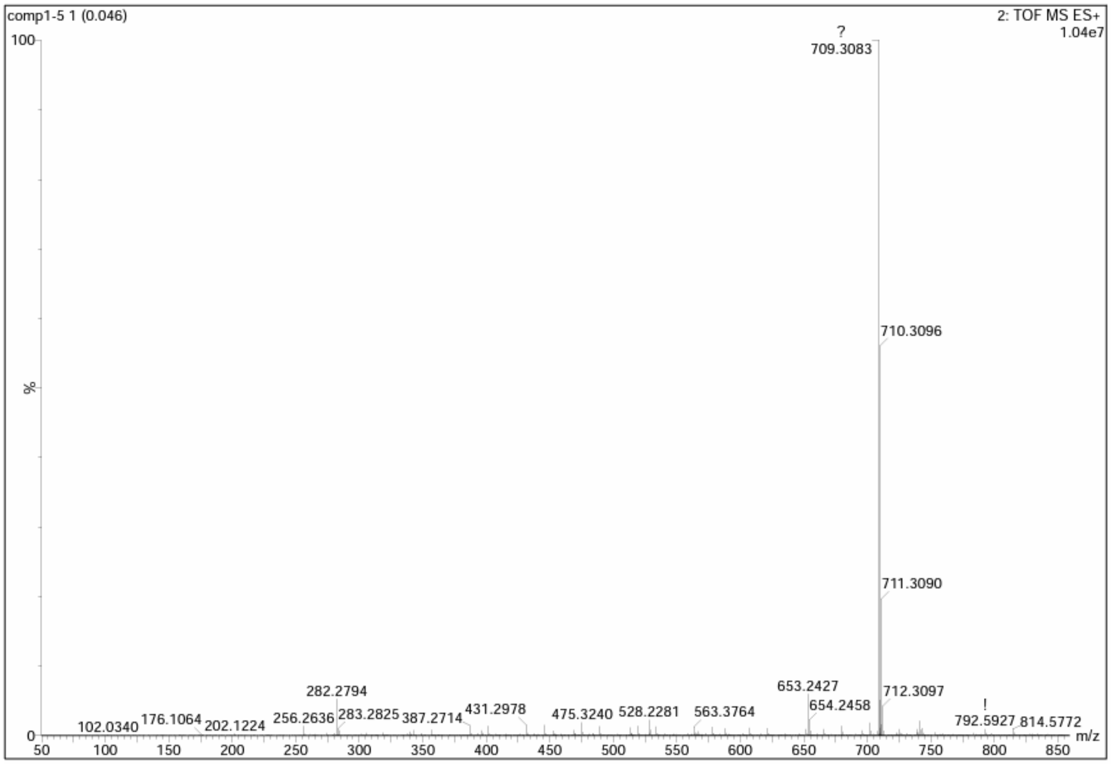

HRMS spectrum of compound **BocNH-C5**

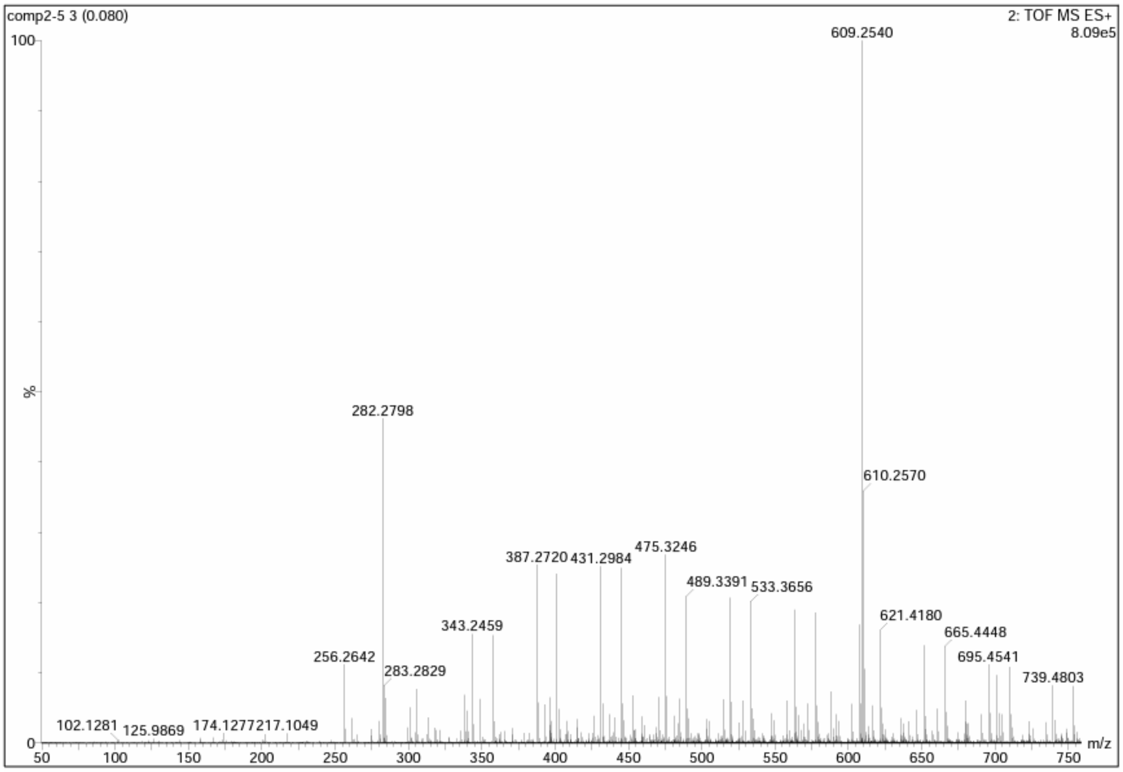

HRMS spectrum of compound **NH_2-C5**

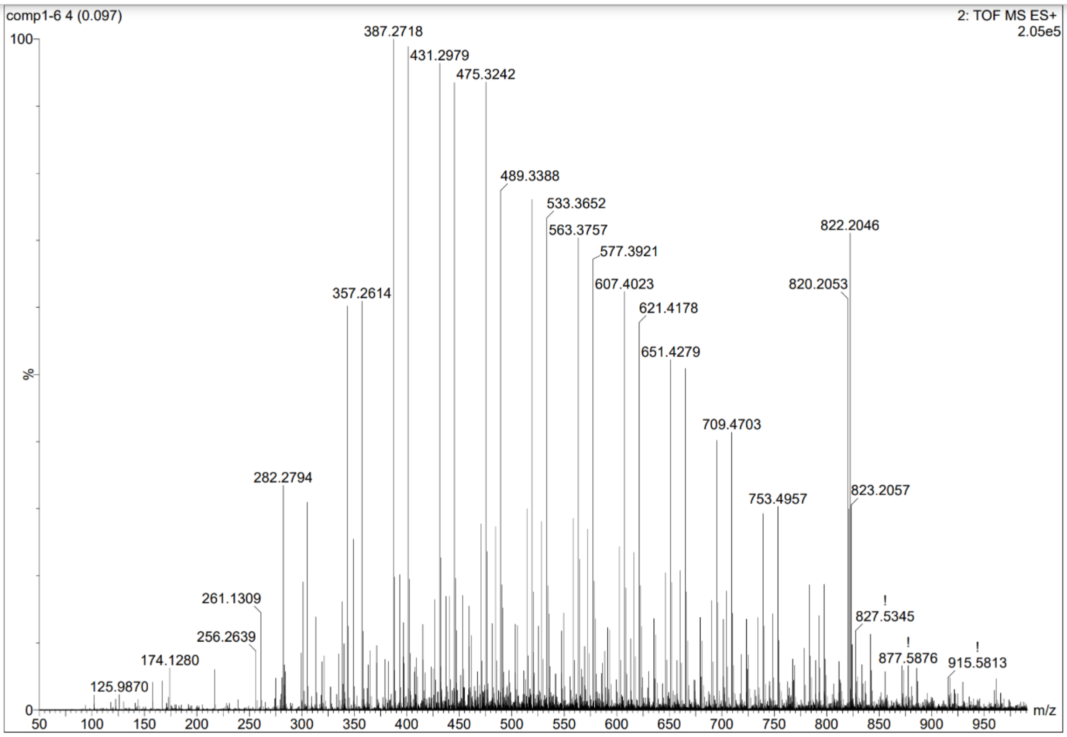

HRMS spectrum of compound **BocNH-C6**

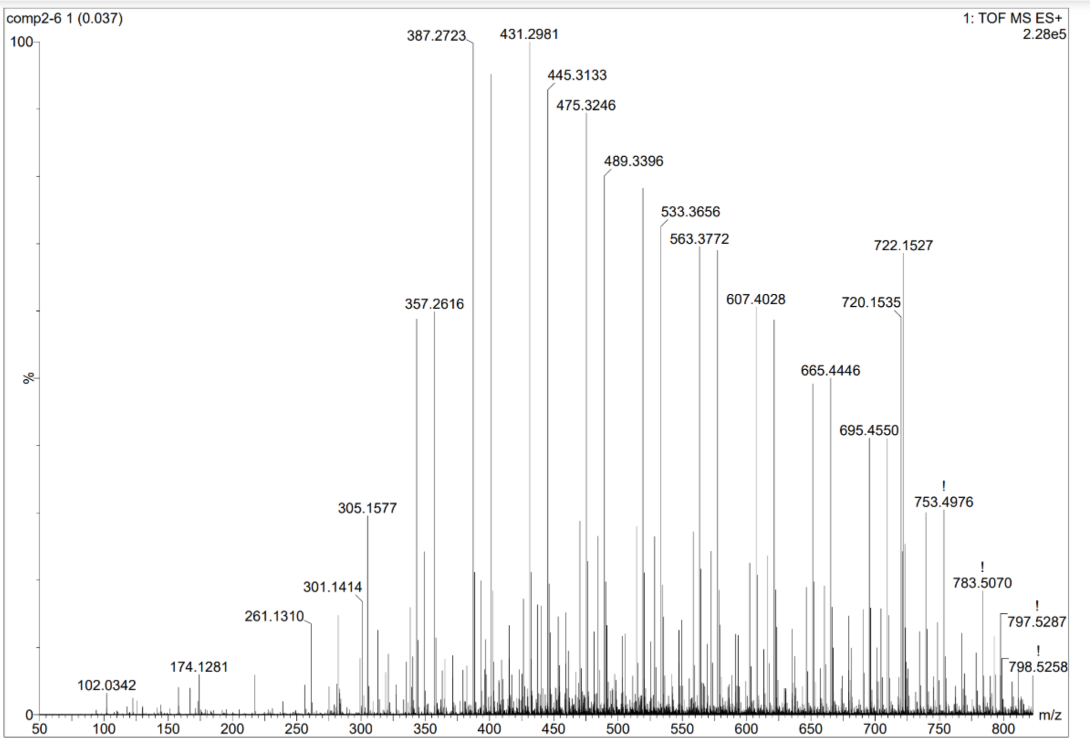

HRMS spectrum of compound **NH_2-C6**

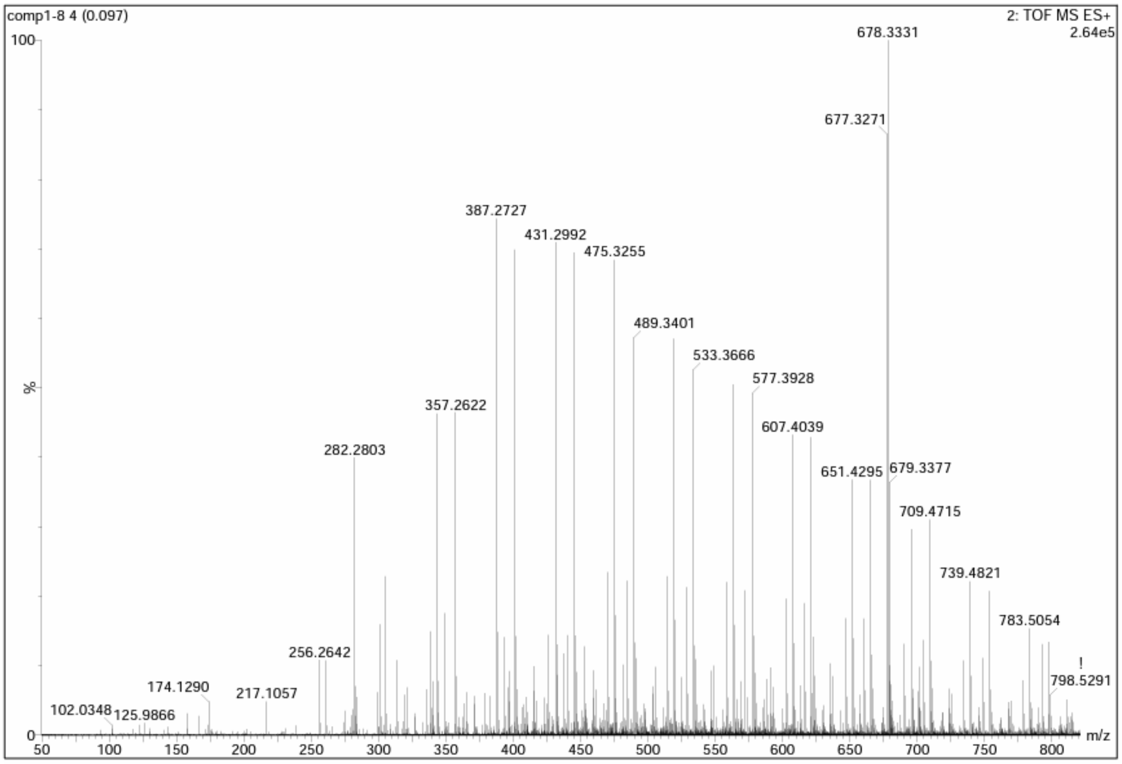

HRMS spectrum of compound **BocNH-C7**

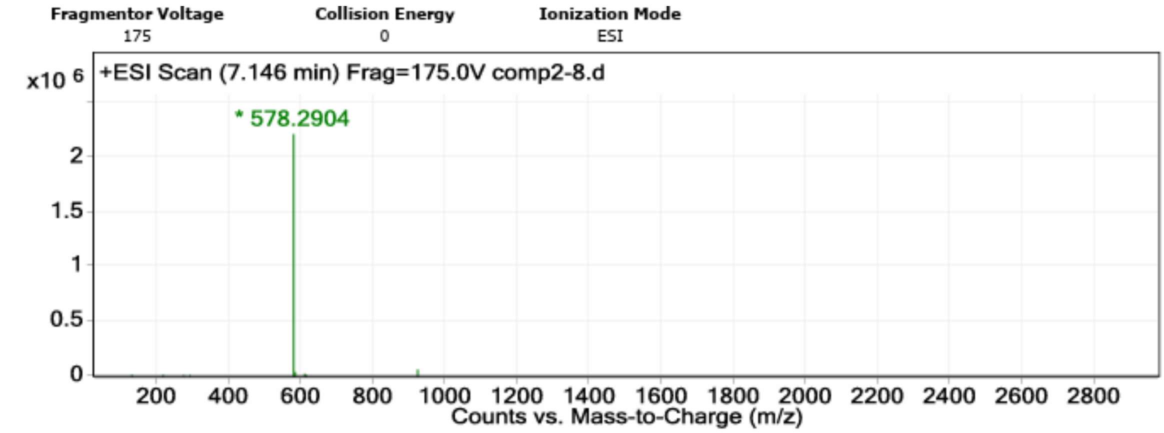

HRMS spectrum of compound **NH_2-C7**

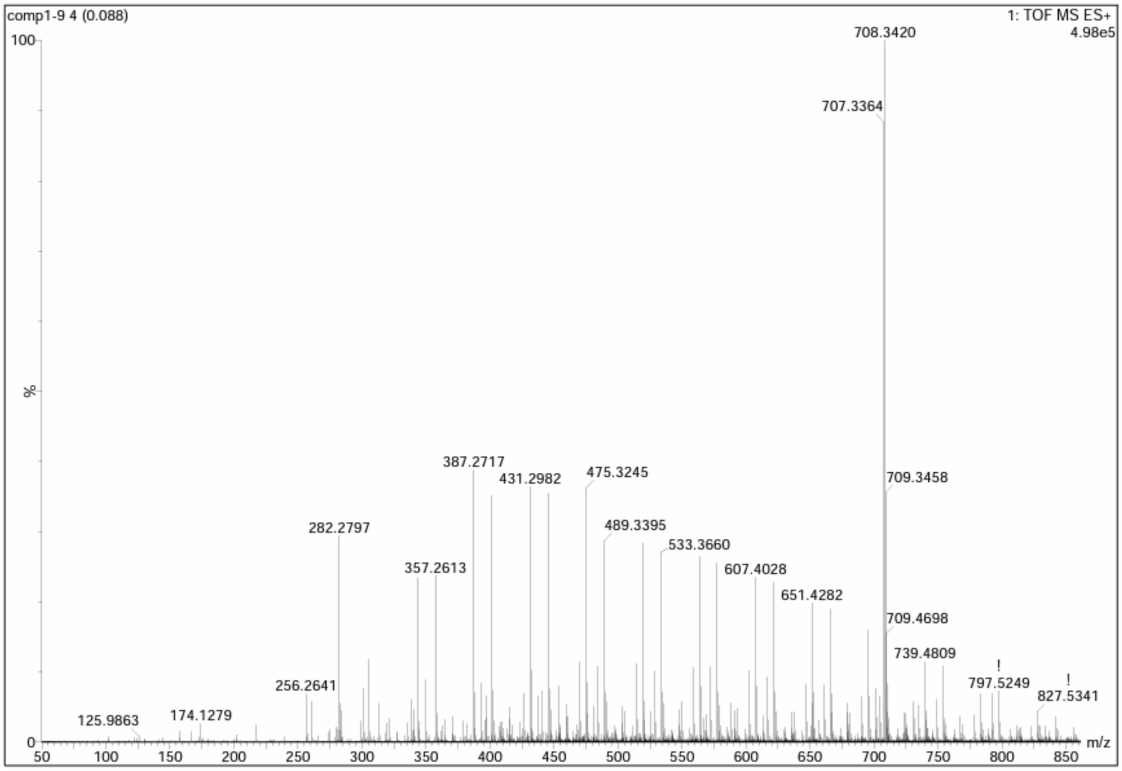

HRMS spectrum of compound **BocNH-C8**

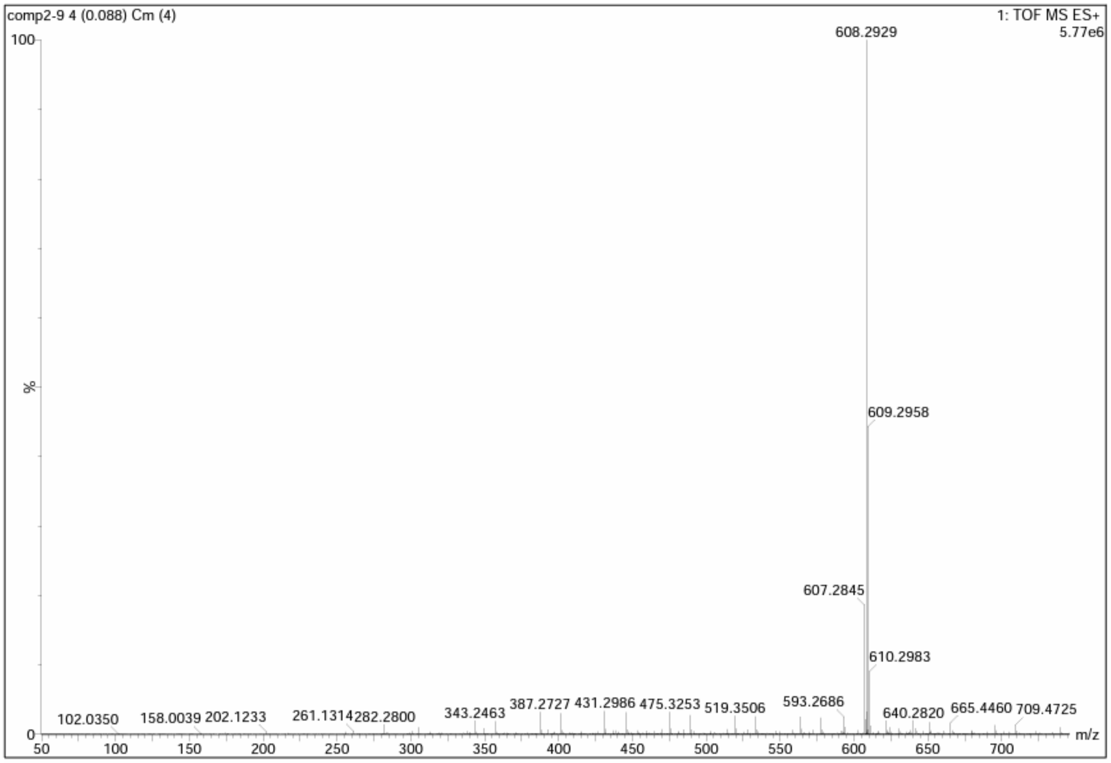

HRMS spectrum of compound **NH_2-C8**

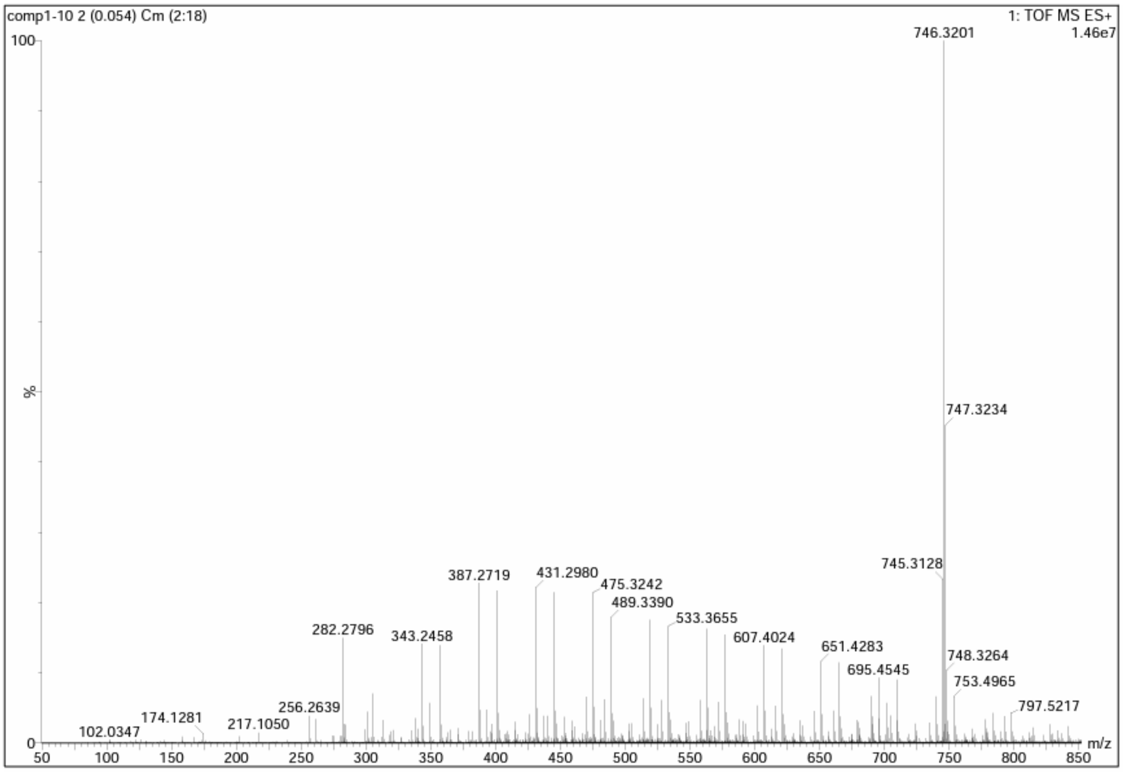

HRMS spectrum of compound **BocNH-C9**

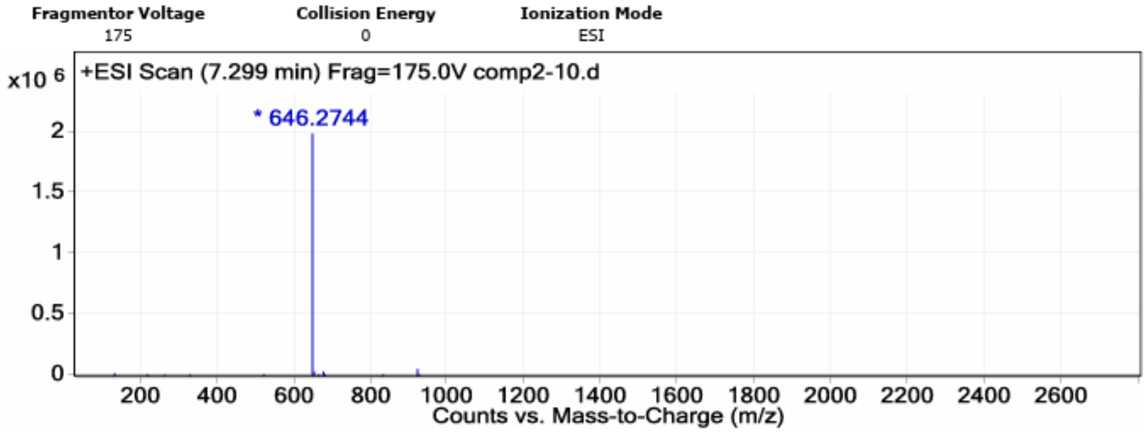

HRMS spectrum of compound **NH_2-C9**

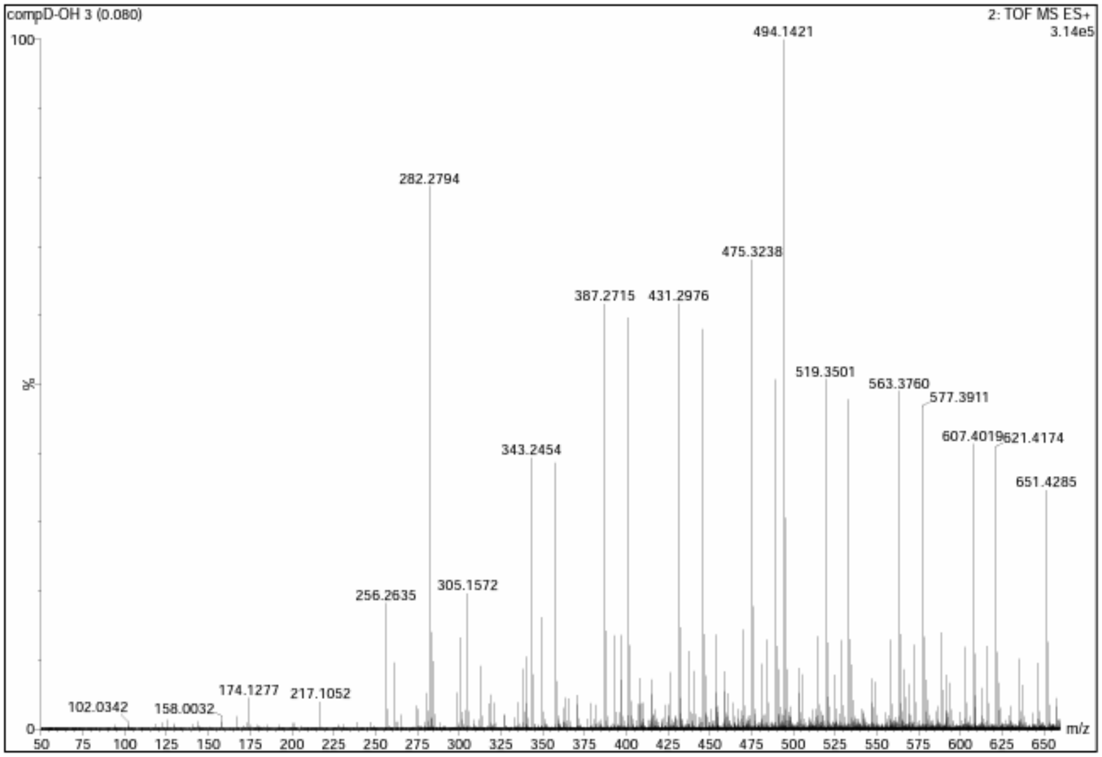

HRMS spectrum of compound **D1**

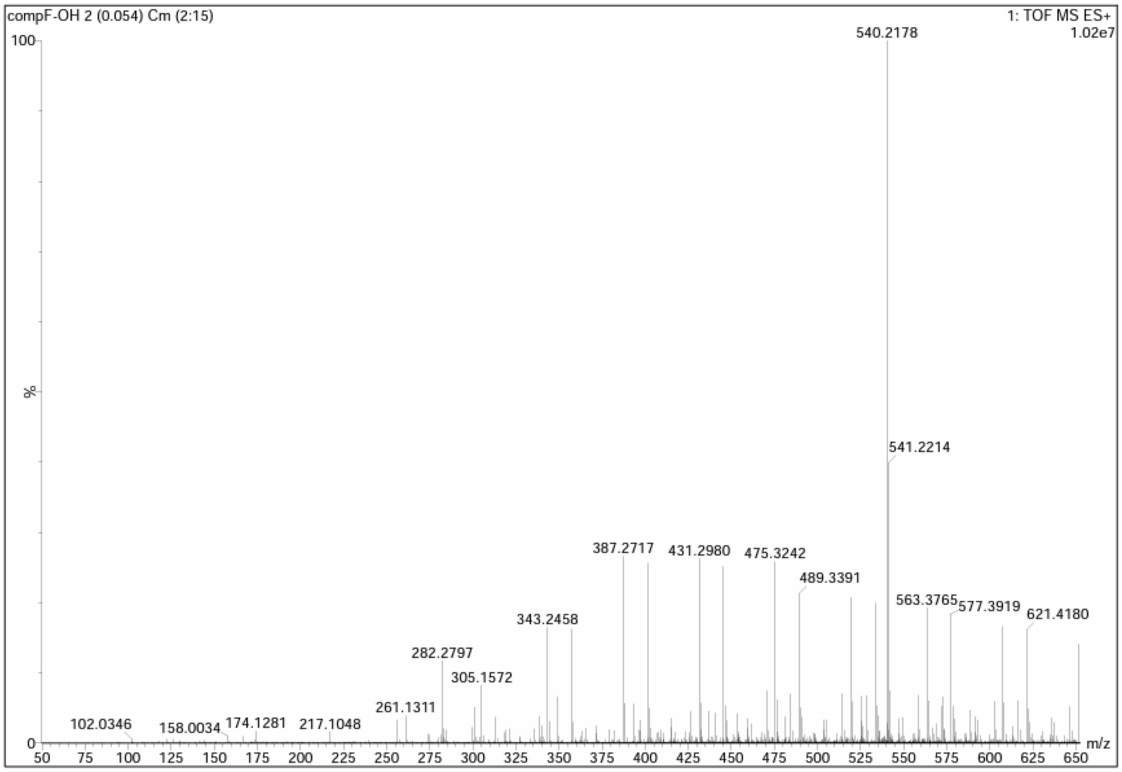

HRMS spectrum of compound **D3**

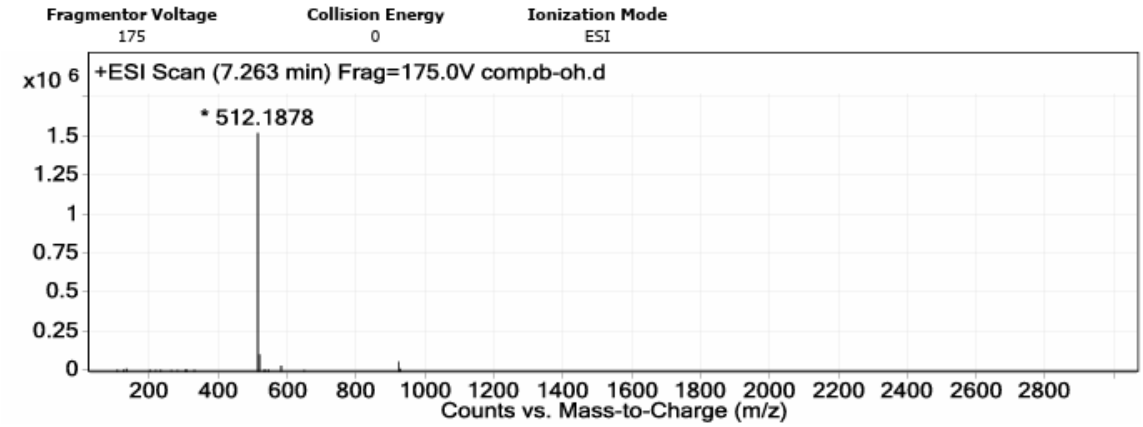

HRMS spectrum of compound **D4**

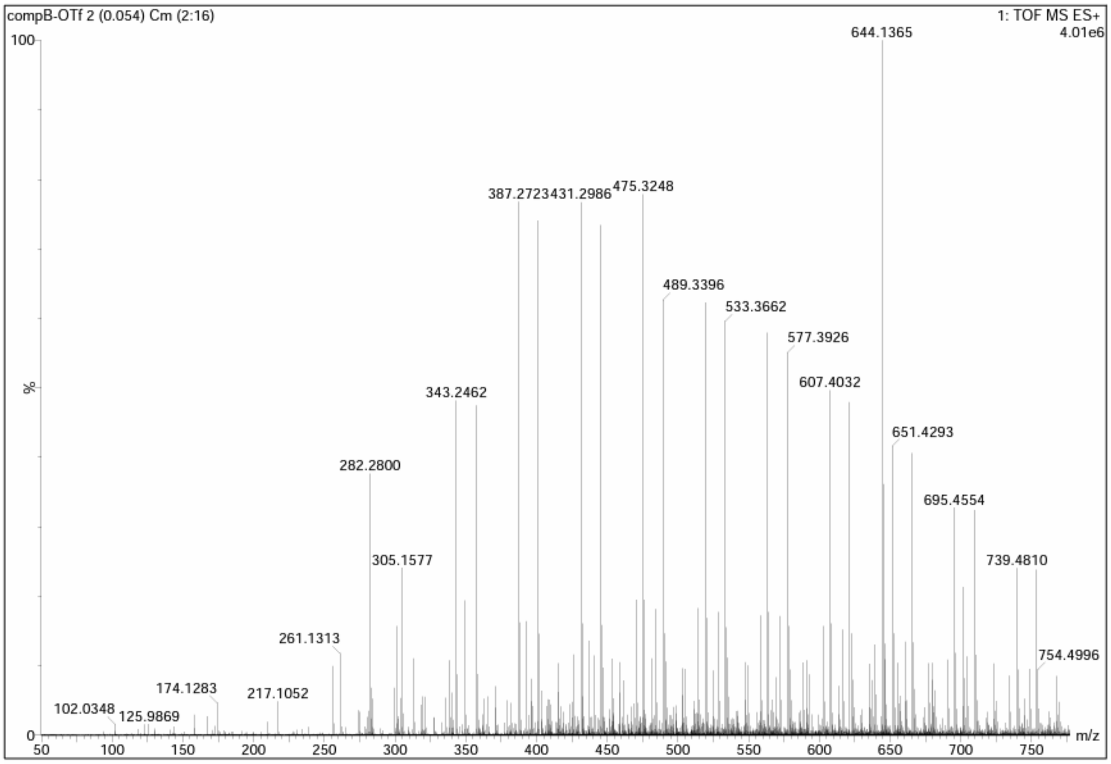

HRMS spectrum of compound **D4-a**

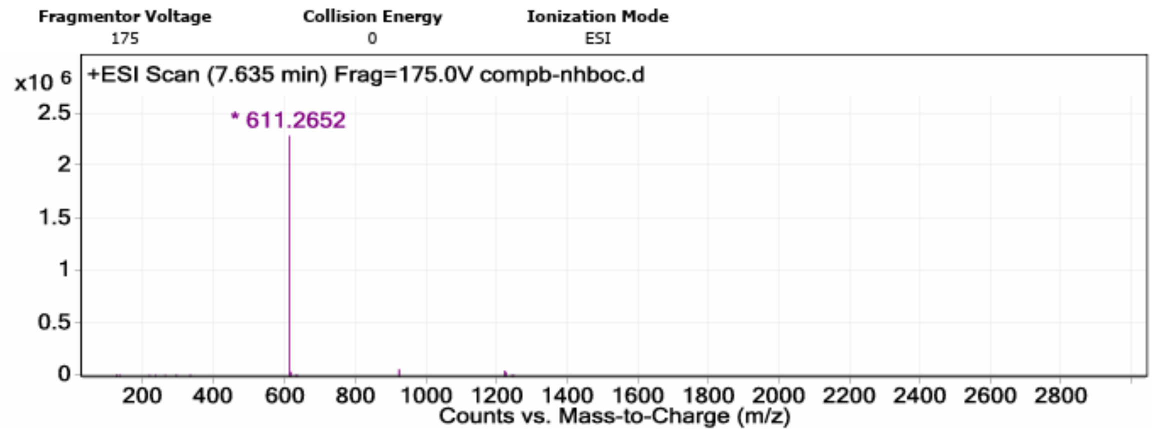

HRMS spectrum of compound **D4-b**

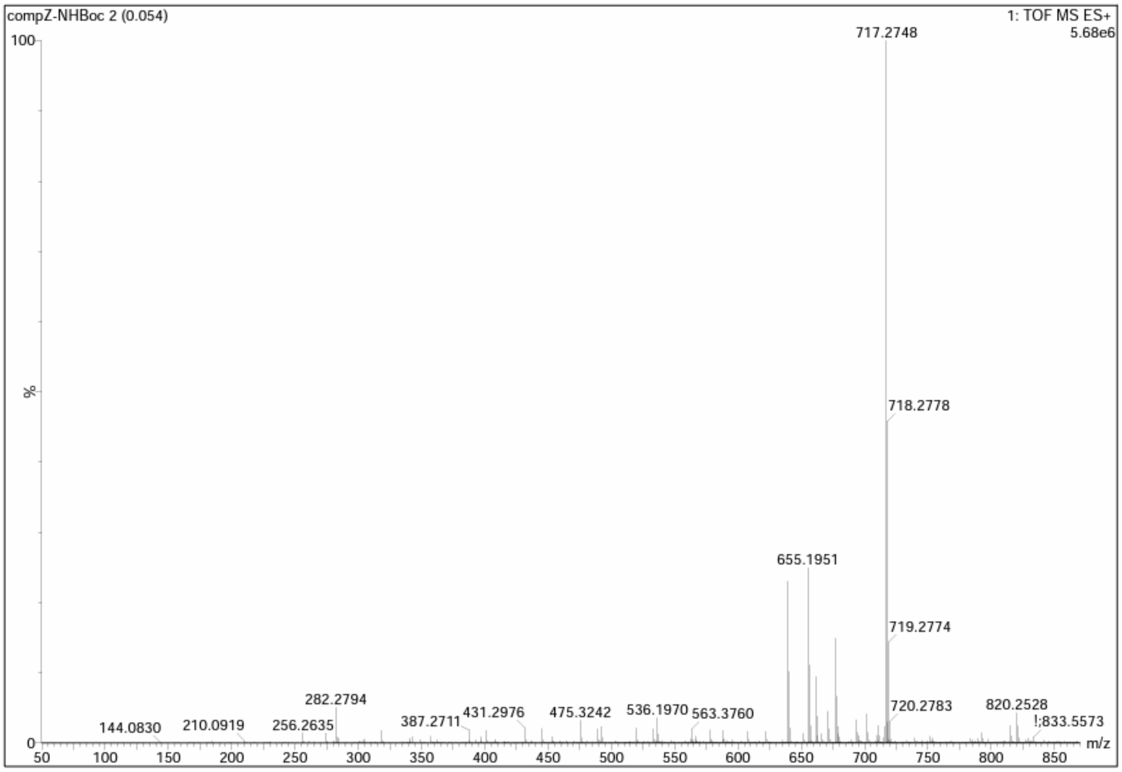

HRMS spectrum of compound **BocNH-D4**

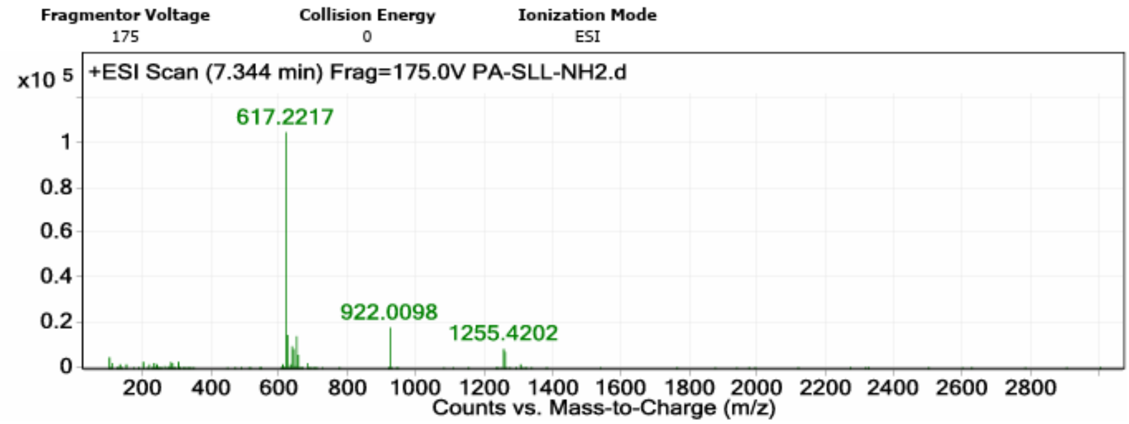

HRMS spectrum of compound **NH_2-D4**

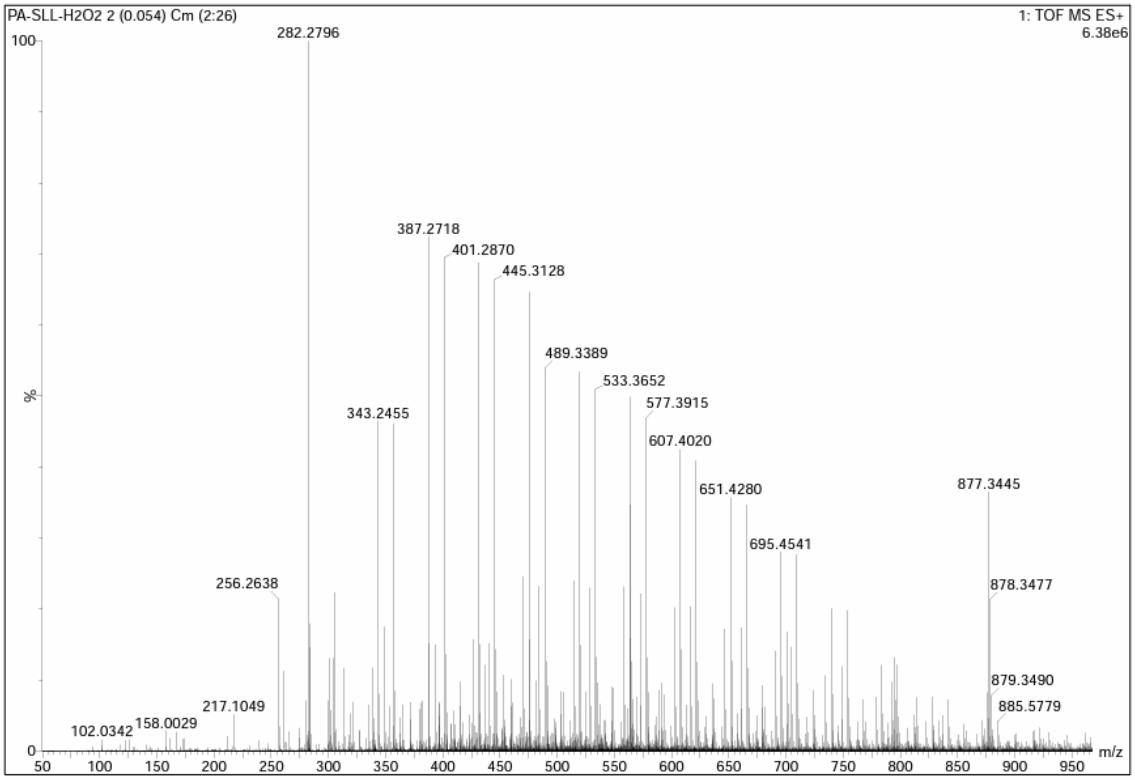

HRMS spectrum of compound **H_2O_2-D4**

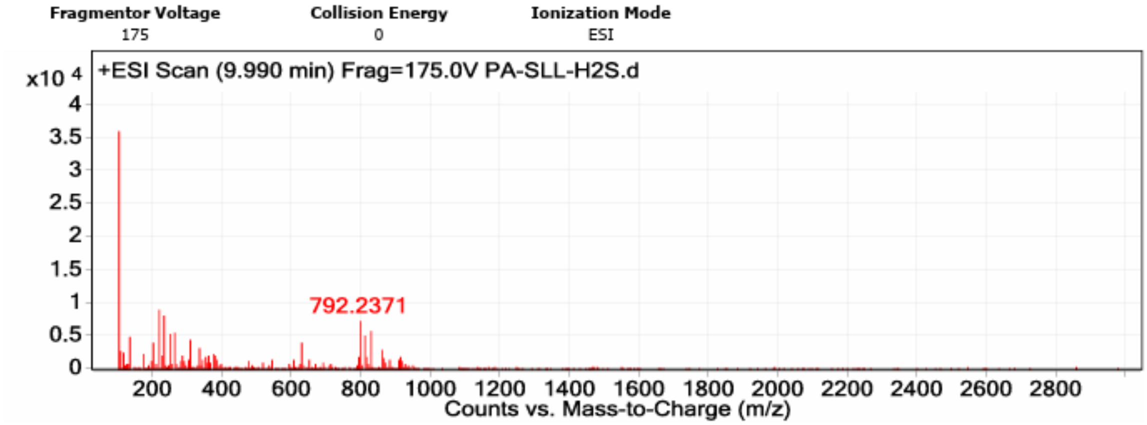

HRMS spectrum of compound **H_2S-D4**
